# Author Correction: The Anglo-Saxon migration and the formation of the early English gene pool

**DOI:** 10.1038/s41586-022-05429-y

**Published:** 2022-10-17

**Authors:** Joscha Gretzinger, Duncan Sayer, Pierre Justeau, Eveline Altena, Maria Pala, Katharina Dulias, Ceiridwen J. Edwards, Susanne Jodoin, Laura Lacher, Susanna Sabin, Åshild J. Vågene, Wolfgang Haak, S. Sunna Ebenesersdóttir, Kristjan H. S. Moore, Rita Radzeviciute, Kara Schmidt, Selina Brace, Martina Abenhus Bager, Nick Patterson, Luka Papac, Nasreen Broomandkhoshbacht, Kimberly Callan, Éadaoin Harney, Lora Iliev, Ann Marie Lawson, Megan Michel, Kristin Stewardson, Fatma Zalzala, Nadin Rohland, Stefanie Kappelhoff-Beckmann, Frank Both, Daniel Winger, Daniel Neumann, Lars Saalow, Stefan Krabath, Sophie Beckett, Melanie Van Twest, Neil Faulkner, Chris Read, Tabatha Barton, Joanna Caruth, John Hines, Ben Krause-Kyora, Ursula Warnke, Verena J. Schuenemann, Ian Barnes, Hanna Dahlström, Jane Jark Clausen, Andrew Richardson, Elizabeth Popescu, Natasha Dodwell, Stuart Ladd, Tom Phillips, Richard Mortimer, Faye Sayer, Diana Swales, Allison Stewart, Dominic Powlesland, Robert Kenyon, Lilian Ladle, Christina Peek, Silke Grefen-Peters, Paola Ponce, Robin Daniels, Cecily Spall, Jennifer Woolcock, Andy M. Jones, Amy V. Roberts, Robert Symmons, Anooshka C. Rawden, Alan Cooper, Kirsten I. Bos, Tom Booth, Hannes Schroeder, Mark G. Thomas, Agnar Helgason, Martin B. Richards, David Reich, Johannes Krause, Stephan Schiffels

**Affiliations:** 1 Max Planck Institute for Evolutionary Anthropology, Leipzig, Germany; 2 University of Central Lancashire, Preston, UK; 3 University of Huddersfield, Huddersfield, UK; 4 Leiden University, Leiden, Netherlands; 5 University of Oxford, Oxford, UK; 6 University of Tübingen, Tübingen, Germany; 7 Center for Evolution and Medicine, Arizona State University, Tempe, AZ USA; 8 Globe Institute, Faculty of Health and Medical Sciences, University of Copenhagen, Copenhagen, Denmark; 9 deCODE Genetics/AMGEN Inc., Reykjavík, Iceland; 10 Department of Anthropology, School of Social Sciences, University of Iceland, Reykjavík, Iceland; 11 University of Münster, Münster, Germany; 12 Department of Earth Sciences, Natural History Museum, London, UK; 13 Department of Genetics, Harvard Medical School, Boston, MA USA; 14 Broad Institute of Harvard and MIT, Cambridge, MA USA; 15 Howard Hughes Medical Institute, Harvard Medical School, Boston, MA USA; 16 Landesmuseum Natur und Mensch, Oldenburg, Germany; 17 University of Rostock, Rostock, Germany; 18 Lower Saxony State Museum, Hanover, Germany; 19 Landesamt für Kultur und Denkmalpflege Mecklenburg-Vorpommern, Schwerin, Germany; 20 Institute for Historical Coastal Research (NIhK), Wilhelmshaven, Germany; 21 Sedgeford Historical and Archaeological Research Project, Sedgeford, UK; 22 Cranfield Forensic Institute, Cranfield Defence and Security, Cranfield University, Cranfield, UK; 23 Melbourne Dental School, University of Melbourne, Melbourne, Victoria Australia; 24 The Atlantic Technological University, Sligo, Ireland; 25 Milton Keynes Museum, Milton Keyes, UK; 26 Cotswold Archaeology, Needham Market, UK; 27 Cardiff University, Cardiff, UK; 28 University of Kiel, Kiel, Germany; 29 University of Zurich, Zurich, Switzerland; 30 Department of Evolutionary Anthropology, University of Vienna, Vienna, Austria; 31 Human Evolution and Archaeological Sciences, University of Vienna, Vienna, Austria; 32 Museum of Copenhagen, Copenhagen, Denmark; 33 Canterbury Archaeological Trust, Canterbury, UK; 34 Isle Heritage CIC, Sandgate, UK; 35 Oxford Archaeology East, Cambridge, UK; 36 University of Birmingham, Birmingham, UK; 37 Centre for Anatomy and Human Identification (CAHID), University of Dundee, Dundee, UK; 38 The Landscape Research Centre Ltd, Yedingham, UK; 39 East Dorset Antiquarian Society (EDAS), West Bexington, UK; 40 Department of Archaeology and Anthropology, Bournemouth University, Poole, UK; 41 Ossatura–Wilhelm-Börker, Braunschweig, Germany; 42 University of York, York, UK; 43 Tees Archaeology, Hartlepool, UK; 44 FAS Heritage, York, UK; 45 Royal Cornwall Museum, Truro, UK; 46 Cornwall Archaeological Unit, Truro, UK; 47 The Novium Museum, Chichester, UK; 48 Fishbourne Roman Palace, Fishbourne, UK; 49 South Downs Centre, Midhurst, UK; 50 BlueSkyGenetics, Adelaide, South Australia Australia; 51 Natural History Museum, London, UK; 52 University College London, London, UK; 53 Department of Human Evolutionary Biology, Harvard University, Cambridge, MA USA; 54 Present Address: Institute of Geosystems and Bioindication, Technische Universität Braunschweig, Braunschweig, Germany; 55 Present Address: Cotswold Archaeology, Needham Market, UK

**Keywords:** Population genetics, Archaeology, Evolutionary genetics, Cultural evolution

Correction to: *Nature* 10.1038/s41586-022-05247-2 Published online 21 September 2022

In the version of this article initially published, the affiliation listed for Alan Cooper was incorrect (South Australian Museum, Adelaide, South Australia, Australia). It instead should have read BlueSkyGenetics, Adelaide, South Australia, Australia, and has been corrected in the HTML and PDF versions of the article

