# Supplementary material for: The Anglo-Saxon migration and the formation of the early English gene pool
Source: Nature. 2022 Sep 21;610(7930):112–9. doi: 10.1038/s41586-022-05247-2 (PMC9534755; doi:10.1038/s41586-022-05247-2)
Supplement: Supplementary file 1 — This file contains Supplementary Notes 1-7. The file also includes Supplementary Figs. 1.1–7.1, Supplementary Tables 2.2–2.7 and additional references. [file 41586_2022_5247_MOESM1_ESM.docx]

**Supplementary Information**

The Anglo-Saxon migration and the formation of the Early English gene pool

Supplementary Information Guide

[Supplementary Note 1 *Site descriptions* 3](#_heading=h.gjdgxs)

[England 3](#_heading=h.30j0zll)

[Apple Down, Chichester, West Sussex 3](#_heading=h.1fob9te)

[Dover Buckland, Dover, Kent 4](#_heading=h.2et92p0)

[Widemouth Bay, Bude, Cornwall 6](#_heading=h.tyjcwt)

[Fox Holes Cave, Clapdale, North Yorkshire 6](#_heading=h.3dy6vkm)

[Eastry Updown, Dover District, Kent 6](#_heading=h.1t3h5sf)

[Ely, East Cambridgeshire, Cambridgeshire 7](#_heading=h.4d34og8)

[Hartlepool, Olive Street, Unitary Authority Hartlepool 8](#_heading=h.2s8eyo1)

[Dover Hill, Folkestone, Kent 9](#_heading=h.17dp8vu)

[Hatherdene Close, Cherry Hinton, Cambridgeshire 9](#_heading=h.3rdcrjn)

[RAF Lakenheath, Eriswell parish, Suffolk 10](#_heading=h.26in1rg)

[Lincoln Castle, Lincoln, Lincolnshire 11](#_heading=h.lnxbz9)

[Newquay, Crantock, Cornwall 12](#_heading=h.35nkun2)

[Norton, Bishopsmill, Stockton on Tees 12](#_heading=h.1ksv4uv)

[Norton, East Mill, Stockton on Tees 12](#_heading=h.44sinio)

[Oakington, South Cambridgeshire, Cambridgeshire 13](#_heading=h.2jxsxqh)

[Polhill, Maidstone District, Kent 14](#_heading=h.z337ya)

[Rookery Hill, Bishopstone, East Sussex 15](#_heading=h.3j2qqm3)

[Sedgeford, King's Lynn and West Norfolk, Norfolk 16](#_heading=h.1y810tw)

[West Heslerton, Ryedale District, North Yorkshire 17](#_heading=h.4i7ojhp)

[Wolverton, Radcliffe School Mid-Saxon Cemetery, Borough of Milton Keynes 18](#_heading=h.2xcytpi)

[Worth Matravers, Football Field, Dorset 19](#_heading=h.1ci93xb)

[Ireland 19](#_heading=h.3whwml4)

[Kilteasheen, The Bishop’s Seat, County Roscommon 19](#_heading=h.2bn6wsx)

[Netherlands 20](#_heading=h.qsh70q)

[Groningen, Groningen 20](#_heading=h.3as4poj)

[Midlum, Friesland 20](#_heading=h.1pxezwc)

[Denmark 21](#_heading=h.49x2ik5)

[Copenhagen, Sct. Clements, KBM3621, Denmark 21](#_heading=h.2p2csry)

[Germany 22](#_heading=h.147n2zr)

[Alt-Inden, North Rhine-Westphalia 22](#_heading=h.3o7alnk)

[Hannover-Anderten, Hannover, Lower Saxony 22](#_heading=h.ihv636)

[Dunum, Wittmund, Lower Saxony 22](#_heading=h.32hioqz)

[Drantum, Cloppenburg, Lower Saxony 23](#_heading=h.1hmsyys)

[Häven, district of Kloster Tempzin, Ludwigslust-Parchim, Mecklenburg-Western Pomerania 24](#_heading=h.41mghml)

[Hiddestorf, Hannover, Lower Saxony 24](#_heading=h.2grqrue)

[Issendorf, district of Stade, Lower Saxony 25](#_heading=h.vx1227)

[Liebenau, Nienburg/Weser, Lower Saxony 26](#_heading=h.3fwokq0)

[Schleswig, Rathausmarkt, Schleswig-Flensburg, Schleswig-Holstein 26](#_heading=h.1v1yuxt)

[Schortens, Friesland, Lower Saxony 27](#_heading=h.2u6wntf)

[Zetel, Friesland, Lower Saxony 27](#_heading=h.19c6y18)

[Supplementary Note 2 *Reference data*, *kinship, uniparental markers, and potential sex bias in early medieval England* 28](#_heading=h.3tbugp1)

[Reference data 28](#_heading=h.28h4qwu)

[Kinship 31](#_heading=h.nmf14n)

[Mitochondrial DNA 32](#_heading=h.37m2jsg)

[Y-chromosome 43](#_heading=h.3l18frh)

[Sex bias 48](#_heading=h.1egqt2p)

[Descriptive statistics 48](#_heading=h.3ygebqi)

[ADMIXTURE analysis of the X-chromosome 50](#_heading=h.2dlolyb)

[Estimating sex-biased ancestry from uniparental markers in the presence of variable admixture proportions 52](#_heading=h.sqyw64)

[Y-chromosomes 53](#_heading=h.4bvk7pj)

[Mitochondrial haplogroups 57](#_heading=h.1664s55)

[Supplementary Note 3 *Continental affinities in early medieval samples from England* 60](#_heading=h.kgcv8k)

[General population genetic affinities 60](#_heading=h.34g0dwd)

[Estimating continental admixture 65](#_heading=h.1jlao46)

[Specific population genetic affinities 66](#_heading=h.43ky6rz)

[Supplementary Note 4 *Genetic structure of continental medieval samples* 72](#_heading=h.xvir7l)

[General population genetic affinities 72](#_heading=h.3hv69ve)

[Identifying outliers 74](#_heading=h.4h042r0)

[Estimating admixture 75](#_heading=h.2w5ecyt)

[Supplementary Note 5 *Continental west European ancestry in ancient and modern England* 79](#_heading=h.3vac5uf)

[Modelling early medieval England 79](#_heading=h.2afmg28)

[Identifying outliers 79](#_heading=h.pkwqa1)

[Modelling early medieval England 83](#_heading=h.39kk8xu)

[Modelling present-day England 88](#_heading=h.1opuj5n)

[Identification of southern European-reated ancestry in England 91](#_heading=h.2nusc19)

[Quantifying the impact on present-day England on a regional level 100](#_heading=h.184mhaj)

[Supplementary Note 6 *Scandinavian Peninsula ancestry in ancient and modern Britain* 111](#_heading=h.2pta16n)

[Early medieval England 111](#_heading=h.14ykbeg)

[Identifying outliers 111](#_heading=h.3oy7u29)

[Estimating admixture from the Scandinavian Peninsula 113](#_heading=h.338fx5o)

[Modelling present-day Britain and Ireland 115](#_heading=h.1idq7dh)

[qpAdm 115](#_heading=h.42ddq1a)

[Supervised ADMIXTURE 115](#_heading=h.2hio093)

[Supplementary Note 7 *Association of CNE ancestry with elements of material culture* 117](#_heading=h.3gnlt4p)

[Fisher’s exact test 117](#_heading=h.1vsw3ci)

[For England as a whole 117](#_heading=h.4fsjm0b)

[Regional Patterns 118](#_heading=h.2uxtw84)

[Cemetery Patterns 118](#_heading=h.1a346fx)

[Wilcoxon Rank Sum test 119](#_heading=h.3u2rp3q)

[References 121](#_heading=h.2981zbj)

# Supplementary Note 1 *Site descriptions*

## England

#### Apple Down, Chichester, West Sussex

The early Anglo-Saxon cemetery at Apple Down, West Sussex, was discovered in 1981 and excavated between 1982 and 1987 by the Chichester Research Committee. The Apple Down cemetery was located north of the old Roman Civitas capital *Noviomagus Reginorum*. It was a mixed-rite site containing 121 inhumation and 64 cremation burials dating from the fifth to the seventh century (Supp. Fig. 1.1)[^1^](https://paperpile.com/c/hqnLqQ/ZybRd). Apple Down is an interesting site because there were three distinct ways to treat the dead: i) cremation, ii) east west inhumation or iii) north south inhumation. These different disposal methods seem to have been contemporary and show different patterns in skeletal trauma, infant burial and grave good use. Consequently, this spatial patterning suggests the co-existence of different groups or communities who shared the same cemetery space but used it in different ways[^2^](https://paperpile.com/c/hqnLqQ/G8IaM).

Provenance note: Samples from Apple Down were provided by the Novium Museum, Chichester. Permission to analyse was granted to coauthor Ceiridwen Edwards in 2012.


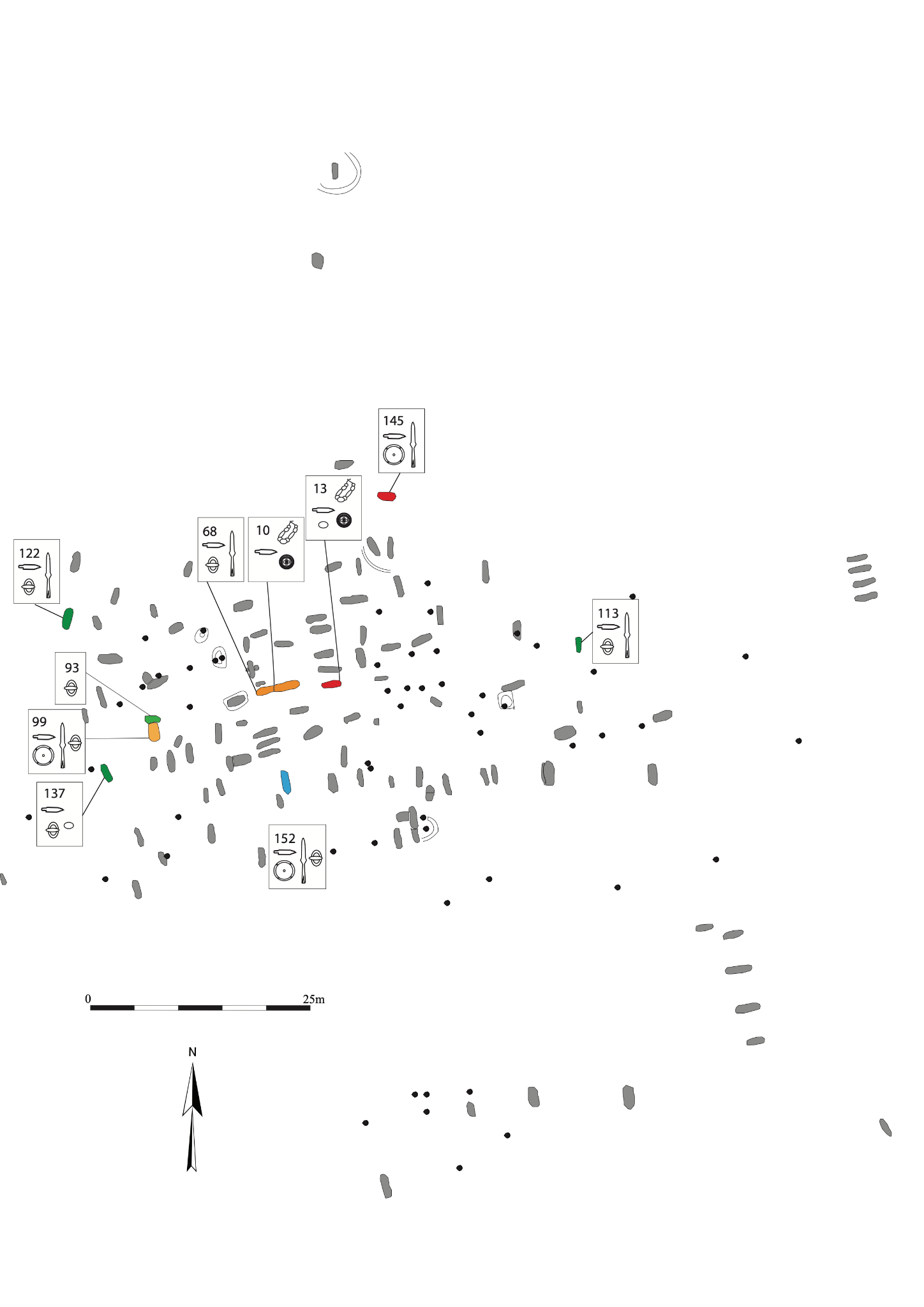


***Supplementary Figure 1.1, Site plan of Apple Down.*** *Shown are the locations of the graves, major artifacts in them, the DNA attribution of each grave (red is CNE, yellow 2/3 CNE, green is WBI, blue 2/3 WBI).*

#### Dover Buckland, Dover, Kent

The early Anglo-Saxon cemetery at Dover Buckland in East Kent was first excavated in 1951 by Vera Everson, following the discovery of artifacts when building the Buckland Estate housing development[^3^](https://paperpile.com/c/hqnLqQ/Gu1L). Further housing development in 1994 saw the second phase of excavation by the Canterbury Archaeological Trust. The sites of these two excavations were separated by an earlier railway line, which destroyed a limited number of graves[^4^](https://paperpile.com/c/hqnLqQ/1dxS). Together, the two elements comprised about 507 inhumation graves, but no cremations. Fascinatingly, these two elements (north and south) were organised in very different ways: The south-westernmost part of the cemetery, excavated in 1994, consisted of seven plots of graves, all neatly separated and consistently clustered at 2m. From this part of the cemetery, samples for ancient DNA analysis were taken (Supp. Fig. 1.2).

Dover Buckland has been established as an important Anglo Saxon site by Everson, who placed a considerable interpretive weight on what she learned from this excavation. She compared Buckland favourably with Merovingian cemeteries and, in particular, their chronologies. She developed Knife and shield boss typologies, and performed a rudimentary investigation of cemetery organisation by comparing the site to others recently excavated. Her impression of an early medieval male martial elite came from her analysis of this site. Subsequent analysis of Dover Buckland has identified several important aspects of female dress costume - including burials which appeared to be buried with objects incongruent with their biological sex[^5,6^](https://paperpile.com/c/hqnLqQ/PzhH+rmlm).

This project sampled from the remains exacted in 1994 and investigated 74 individuals. The sample was selected based on petrous bone preservation and includes individuals with a diverse range of grave goods, both full weapon burials and brooch burials as well as individuals with no artifacts at all. Grave 281 has previously been identified as a male based on skeletal assessment but is genetically female. Her grave goods included disc brooches, beads, and two small square-headed brooches.

Provenance Note: ​​Samples from Dover Buckland were provided by the Canterbury Archaeological Trust. Permission to analyse was granted to coauthor Duncan Sayer in 2019.


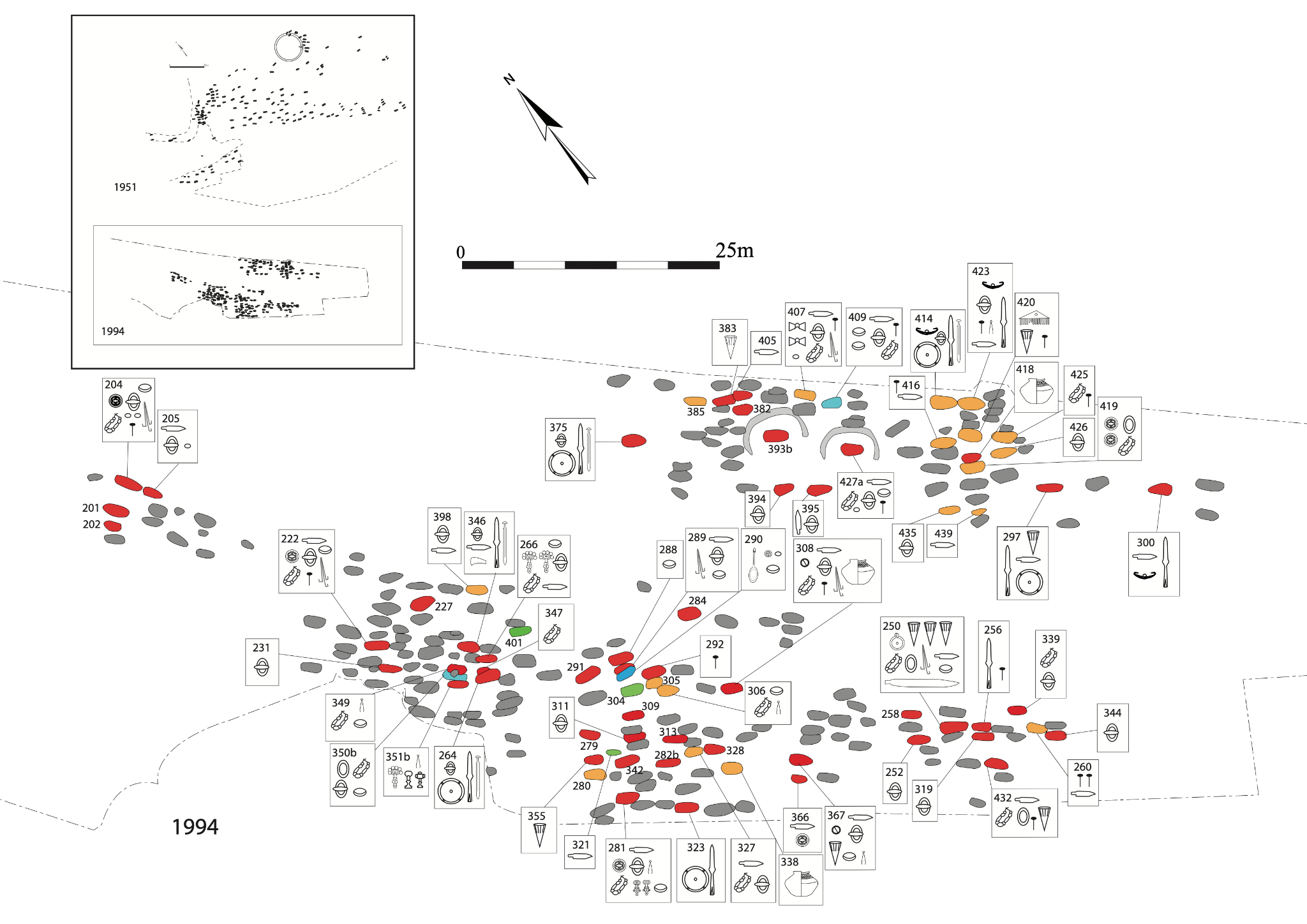


***Supplementary Figure 1.2, Site plan of Dover Buckland.*** *Shown are the locations of the graves, major artifacts in them, the DNA attribution of each grave (red is CNE, yellow 2/3 CNE, green is WBI, blue 2/3 WBI).*

#### Widemouth Bay, Bude, Cornwall

Widemouth Bay is a long, open coastline on the Atlantic coast of Cornwall, located close to the town of Bude. A Mr. J. North undertook some excavations on the Bay in 1968 and 1972. Very little detailed information is available regarding why these excavations took place and what was found. The excavations uncovered various finds including human and animal bone, a spindle whorl, a slate disc, a whetstone, some pottery, flint, shell, charcoal and an iron nail. The finds are stored at the Royal Cornwall Museum. A sample of the petrous portion of a temporal bone found at Widemouth Bay is included in this study.

Provenance note: Samples from Widemouth Bay were provided by the Royal Cornwall Museum. Permission to analyse was granted to coauthor Tom Booth in 2019.

#### Fox Holes Cave, Clapdale, North Yorkshire

Fox Holes Cave is a rock shelter near Trow Gill, close to the village of Clapdale in the North Yorkshire Dales[^7,8^](https://paperpile.com/c/hqnLqQ/erQAB+IJ2Jc). Excavations at Fox Holes Cave in the 1920s uncovered human bones representing the remains of at least two people (Individual 1 and 2), as well as faunal remains, Neolithic pottery, Neolithic and Bronze Age arrowheads, stone tools and a bone pin. The burials were partial but semi-articulated, suggesting whole bodies had been interred in the cave originally. The human remains are kept by Tom Lord in his collection at Lower Winskill Farm. Human remains dating to the Early Neolithic have been recovered from other caves in the area which, combined with the prehistoric finds, suggested that the human remains from Fox Holes Cave were likely to be prehistoric, and Early Neolithic specifically. However, radiocarbon dating of the temporal bone of Individual 1 produced a surprising date of 655-774 cal. CE (1295 ± 36 bp, UBA-30799), in the early medieval period. A sample from the petrous portion of the temporal bone of Individual 2, which was assumed to date to around the same time as Individual 1, is included in this study.

Provenance note: Samples from Fox Holes Cave were provided by the Tom Lord’s collection at Lower Winskill Farm. Permission to analyse was granted to coauthor Tom Booth in 2015.

#### Eastry Updown, Dover District, Kent

The early Anglo-Saxon cemetery at Updown, Eastry (or Eastry III) was excavated in 1976 by S.C. Hawkes in front of a water pipe and 1989 by Kent Rescue Unit. There are at least four cemeteries in Eastry and it has been suggested that it was an important centre within early medieval Kent. Eastry is part of a wealthy group of cemeteries in East Kent. The two phases of work on this site have identified a total of 78 inhumation graves dating to the seventh century and at least 19 small mounds (Supp. Fig. 1.3). There are a range of grave goods from Eastry including jewellery items and weapons, but notably the cemetery includes imported objects from the Frankish continent and even a Byzantine buckle and Amethyst Beads[^9^](https://paperpile.com/c/hqnLqQ/bwBUZ).

Provenance note: Samples from Eastry Updown were provided by the University of Central Lancashire. Permission to analyse was granted to coauthor Duncan Sayer in 2017.


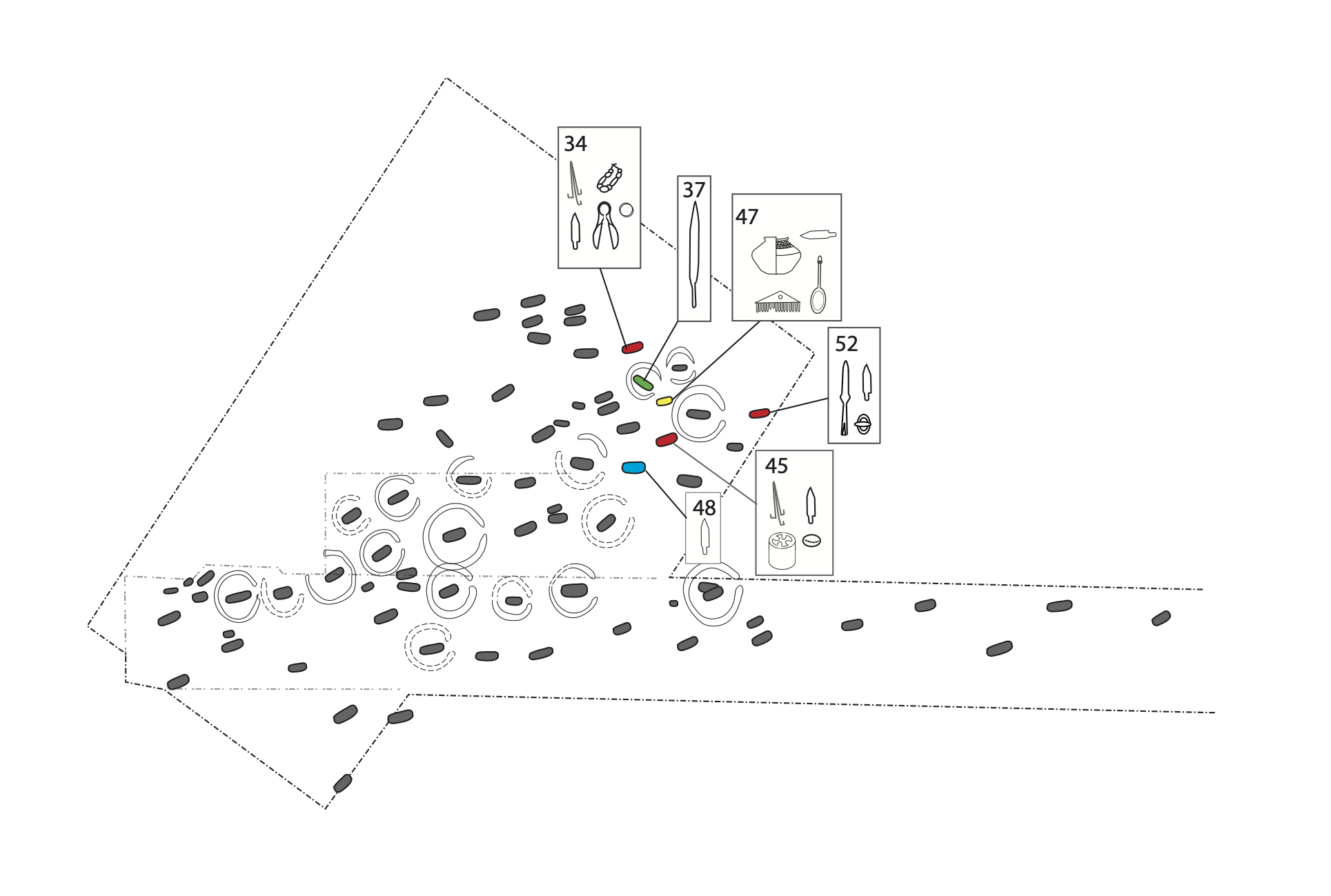


***Supplementary Figure 1.3, Site plan of Eastry Updown.*** *Shown are the locations of the graves, major artifacts in them, the DNA attribution of each grave (red is CNE, yellow 2/3 CNE, green is WBI, blue 2/3 WBI).*

#### Ely, East Cambridgeshire, Cambridgeshire

There are four Early Anglo-Saxon cemeteries from Ely, Cambridgeshire. The one sampled for this project was excavated by Oxford Archaeology East in 2017, and was located in Field D North West Ely. It was a mixed rite site consisting of 28 burials and two cremations. Twenty of the burials were sixth century in date and along with the cremations they were found in the northern group aligned alongside a Roman track (Supp. Fig 1.4). To the south there were eight burials within the limits of a small Roman enclosure. These eight may have been a little later in date and finds dated them to the later 6th and seventh centuries.

Provenance note: Samples from Ely were provided by Oxford Archaeology East. Permission to analyse was granted to coauthor Duncan Sayer in 2017.


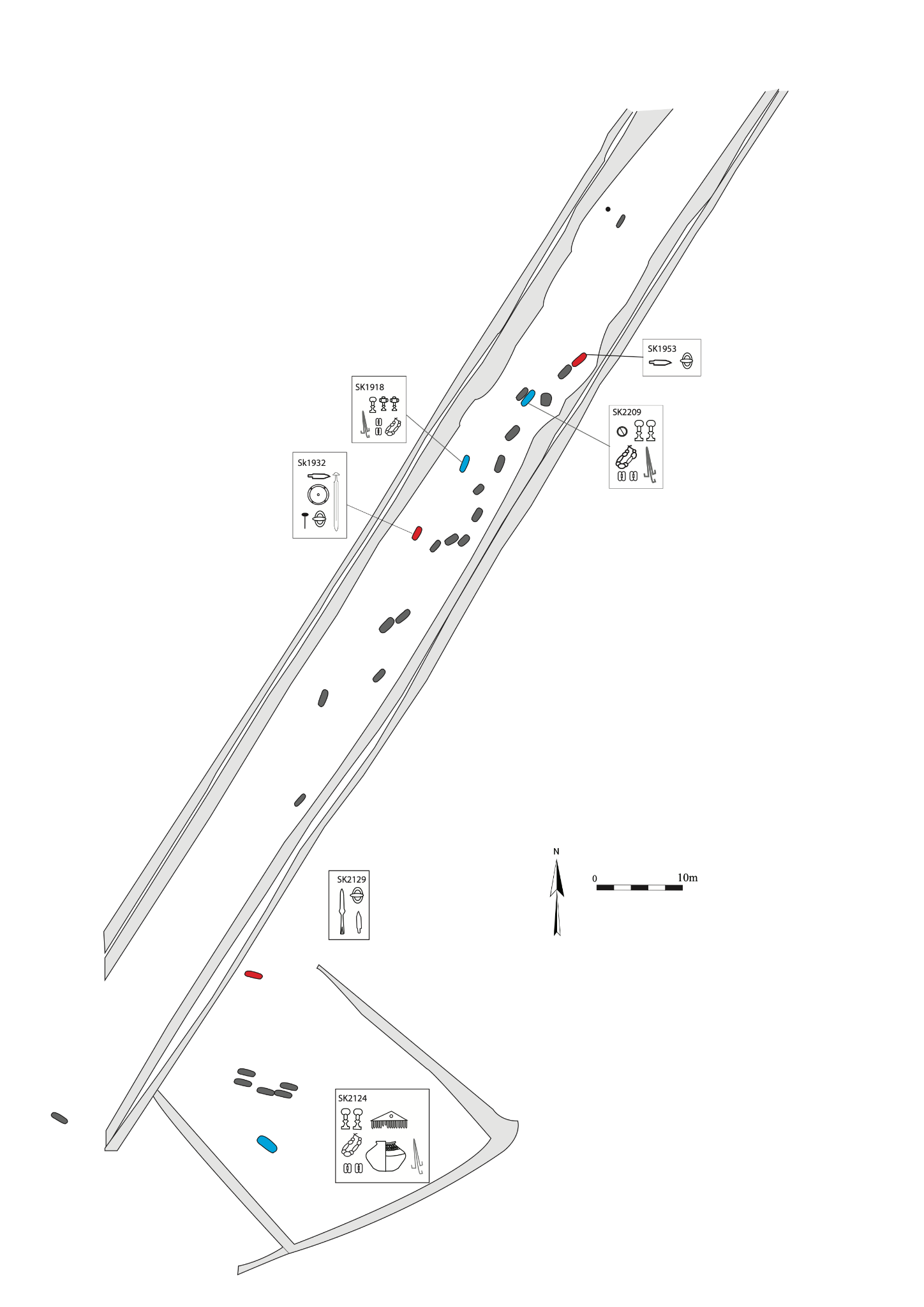


***Supplementary Figure 1.4, Site plan of Ely.*** *Shown are the locations of the graves, major artifacts in them, the DNA attribution of each grave (red is CNE, yellow 2/3 CNE, green is WBI, blue 2/3 WBI).*

#### Hartlepool, Olive Street, Unitary Authority Hartlepool

The site at Olive Street, Hartlepool, England was an accidental discovery. During repair work to a stone wall, human remains were found and Tees Archaeology were asked to investigate. In total six bodies were excavated and a small portion of a stone structure. There had been extensive inter-cutting of graves. Three of the bodies were radiocarbon-dated and these all provided dates between the 7^th^ and 10^th^ centuries. This is thought to be part of a large cemetery, part of which was excavated in 1964 and again yielded pre-conquest radiocarbon dates. The archive is held by Tees Archaeology and the site is forthcoming in the Durham Archaeological Journal under the title “Anglo-Saxon remains from Back Gladstone Street, Hartlepool” by Stephen J Sherlock.

Provenance note: Samples from Hartlepool were provided by Tees Archaeology. Permission to analyse was granted to coauthor Tom Booth in 2019.

#### Dover Hill, Folkestone, Kent

Digging associated with road building on Dover Hill near Folkestone in Kent through the late 19^th^ and early 20^th^ century resulted in repeated encounters with the remains of an early medieval ‘Pagan Saxon’ cemetery. Road widening works in 1907 led A.G. Nichols to excavate 35 of the burials from this Dover Hill cemetery. The graves had been cut into the soft chalk bedrock, and the heads of the skeletons were often supported by chalk pillows. Burials were accompanied by various grave goods; double-edged swords, spearheads, shield bosses, knives, a dagger, a crystal ball, several buckles, various amber and glass beads and one garnet-inlaid brooch. Based on the finds, the cemetery is thought to date to between 450-600 CE. In most cases only the skulls of the burials were retained and deposited at the Natural History Museum in London. However, one skeleton, Burial 4, a 30-40-year-old female was lifted in full and deposited at Folkestone Museum. Burial 4 was found with a brooch, a knife, a belt buckle, an amber bead necklace and bead bracelets The skeleton, known as the Folkestone Woman, is still on display at Folkestone Museum and has been nicknamed ‘Aefre’. A sample of the petrous portion of the temporal bone of ‘Aefre’ is included in this study.

Provenance note: Samples from Dover Hill were provided by the Folkestone Museum. Permission to analyse was granted to coauthor Tom Booth in 2016.

#### Hatherdene Close, Cherry Hinton, Cambridgeshire

Hatherdene Close is an early Anglo-Saxon cemetery located in Cherry Hinton, Cambridgeshire and was excavated by Oxford Archaeology East in 2007 and 2016[^10^](https://paperpile.com/c/hqnLqQ/s5oNd). The cemetery was an inhumation site and consisted of 126 individuals dating to the early Anglo-Saxon period, 5^th^ to 6^th^ century. The majority of graves lay to the northeast of a Roman funerary enclosure and Iron Age square barrow, with eleven graves within the earlier features (Supp. Fig. 1.5). Hatherdeane is noteworthy because there were a number of ‘stacked’ burials where the same grave was used for multiple internments of individuals, one on top of the other as opposed to a more usual side by side interment seen in multiple inhumation graves.

Provenance note: Samples from Hatherdene Close were provided by Oxford Archaeology East. Permission to analyse was granted to coauthor Duncan Sayer in 2017.


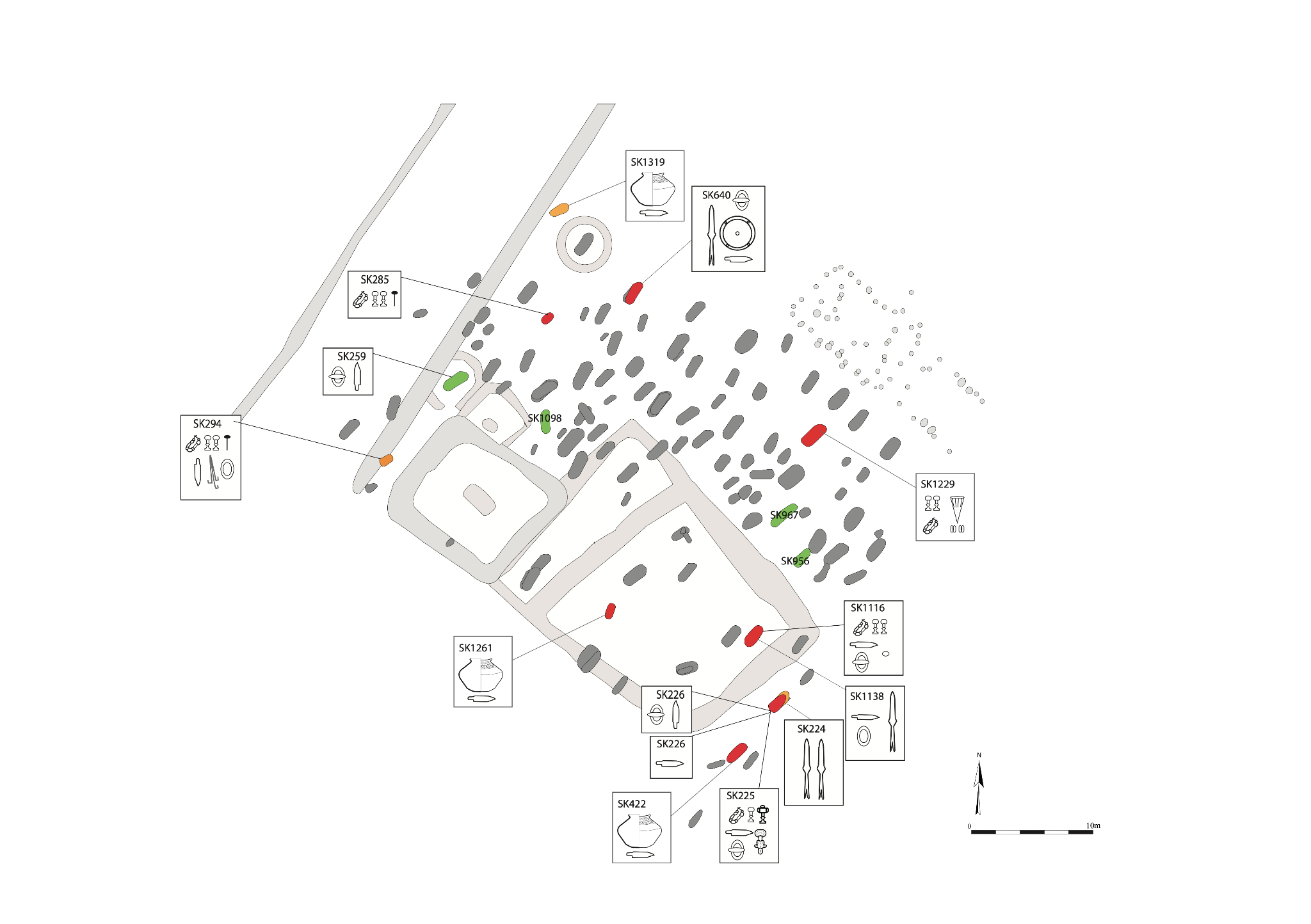


***Supplementary Figure 1.5, Site plan of Hatherdene Close.*** *Shown are the locations of the graves, major artifacts in them, the DNA attribution of each grave (red is CNE, yellow 2/3 CNE, green is WBI, blue 2/3 WBI).*

#### RAF Lakenheath, Eriswell parish, Suffolk

Early Anglo-Saxon burials within the perimeter of the military airbase RAF Lakenheath and also within the bounds of the parish of Eriswell were first discovered in 1957 as a result of building work. Excavation in 1959 was led by Grace, Lady Briscoe, in 1959, with the finds from 33 graves published as the cemetery of Little Eriswell by Patricia Hutchinson in 1966 (ERL 008). Close to this site, three more inhumation graves were found in a pipe-trench in May 1979 (ERL 046). From 1997, larger scale surveys and excavations were carried out funded by the Ministry of Defence Estates in advance of extensive new building.

This fieldwork identified what appear to be three discrete burial grounds, which were closely spaced and in contemporary use. They are referred to in the forthcoming publication and study of the site as the East (ERL 008 and ERL 114), Central (ERL 046) and West (ERL 104 and ERL 161) burial grounds. There is vestigial evidence of cremation burial from around or before the middle of the 5^th^ century CE in the west site, with inhumation introduced there by the final quarter of that century and perhaps as early as c. 460 CE. Inhumation in the central and east burial grounds appears to have commenced at much the same time as in the west. Although the limits of excavation mean that one cannot be certain that the East and Central burial grounds do not join up, the layout of graves differs between the three areas, with burial in the East surrounding a large Bronze-age barrow, burial in the central burial ground in quite regular rows running south-north, and a more complex polyfocal grouping in the west. Burial took place in all three burial grounds through to the third quarter of the 6th century, after which it continued in the west alone. The cessation of burial in the west site appears to have been correlated with the start of burial beside another Bronze-age barrow at the Lord’s Walk site some 300 metres to the south.

The burial grounds are of different sizes: the burials of 62 individuals have been excavated in the central site, 99 (of the Early Anglo-Saxon Period, plus three of the Bronze Age) in Little Eriswell/the east site, and 273 who were inhumed, plus a minimum of 8 cremations, in the West. There are relatively few double or multiple internments in one grave. The chronology of the burials indicates a substantial drop in the burying population between Stage 1, when all three sites were being used, and Stage 2, when the West site alone continued in use: possibly a decrease of as much as 80%. The West burial ground includes one especially early adult female grave (G363) from which the isotopic evidence suggests the woman buried had lived her early life on the other side of the North Sea in the area of Denmark, possibly Jutland. An outstanding grave is G323, the burial of a young adult male with a full weapon set and a very richly harnessed sacrificed horse.

The 15 samples that have been analysed for DNA are mostly (13) from the west site with one each from the Central and East burial grounds. Chronologically, they range from the earliest identifiable burials to the latest, with a preponderance of the former. Eleven are from males and 4 from females. The sample includes very early graves from each of the three burial grounds: G363(W), G031(C) and G405(E). The principal criterion for sample selection, however, has been observable evidence suggesting close genetic relationship: e.g. collocation in what look like family plots, often supported by less common, potentially heritable, skeletal traits, and in the case of G141, G298a and G298b previous aDNA analysis indicating a close genetic relationship. G405 is also an outlier in terms of the isotopic profile, although less markedly so than G363. The principal clusters are summarized below. G323 appears in fact to have a dominant and influential position for much of the development and use of space in the west burial ground, although radiocarbon dating evidence and the grave goods do not support a view that it is a ‘founder grave’ in any strict sense. Other potentially heritable traits link G323 with other burials in the sample.

Provenance Note: Samples from RAF Lakenheath where provided by Cotswold Archaeology Suffolk. Permission to analyse was granted to coauthor Duncan Sayer in 2017.

#### Lincoln Castle, Lincoln, Lincolnshire

Nine intact inhumation burials were excavated within a trench for a stair and lift foundation positioned within the Masons’ Yard, Lincoln Castle in 2014 (Burials 1-7, 9 and 11). In addition, an assemblage of disarticulated human bone indicated the disturbed burials of 40 men, women and children, and a watching brief in 1993 encountered two further burials 24m to the northwest of the Masons’ Yard suggesting a long-lived and relatively sizable burial ground. The nine burials excavated within the Masons’ Yard were associated with a lime-mortared stone building identified as a lost church or chapel. Burial 9, a female aged 26-35, predated the stone building, while Burial 1, a male aged 18-25, had been reinterred carefully within the foundations during initial construction; the seven further burials were subsequently made inside the church or chapel. A programme of bioarchaeological analysis was carried out on the burials including high-precision radiocarbon dating and Bayesian analysis, multi-isotope analysis investigating diet and place of origin, and ancient DNA analysis.

Radio-carbon dating and statistical modelling using stratigraphic information placed the construction of the church or chapel and associated burials in the decades prior to the Norman Conquest – c.985-1070; the construction of the stone-built East Gate and East Range dating to the 1080s oversailed the remains of the church or chapel. Multi-isotope analysis indicated two individuals – Burial 4 and 6 – were probably born in or near Lincoln, with the other individuals having migrated from elsewhere in Britain or from warmer climes. Samples from the burials were submitted for aDNA analysis, of which three provided ancient DNA sufficient to have interpretive value: from Burial 2, a child aged 10-14; Burial 6, a male aged 36-45; Burial 11, a child aged 2-6.

Provenance Note: Samples from Lincoln Castle where provided by FAS Heritage. Permission to analyse was granted to coauthor Tom Booth in 2015.

#### Newquay, Crantock, Cornwall

Numerous burials have been found at Crantock since the 19^th^ century, although few have been recorded or published. In 1968, for example two adult male inhumations were found at Beach Road and in 1974 a pipeline cut across the old part of the churchyard revealed seven cist burials, oriented east-west[^11^](https://paperpile.com/c/hqnLqQ/Gy9n6). Odd bits of bone were found along the line, and at approximately SW 7900 6055 a complete skeleton of an adult female within a cist (Irwin, unpublished typescript; MCO26511). The bones recovered in March 1980 were excavated by workmen who were adding an extension to a cottage. They were removed from the site by an archaeologist, and subsequently studied by Frank Turk who wrote an unpublished report on the burials[^12^](https://paperpile.com/c/hqnLqQ/Lyhrp). They were found in the steeply sloping, terraced, much-disturbed garden of St Ambrose Cottage on the left-hand side and seaward end of Beach Road. As they were excavated vertically and not horizontally, not much could be said about method of burial, but one was ‘contracted’ in so far as the leg bones were by the head, and they were face-downwards with a big stone on top. A piece of glazed pottery found with them was not more than 200 years old. Turk dates the bones to the early part of the medieval period. They were found close to the site of St Ambrusca’s chapel and holy well.

Provenance Note: Samples from Newquay, Crantock where provided by the Royal Cornwall Museum. Permission to analyse was granted to coauthor Tom Booth in 2019.

#### Norton, Bishopsmill, Stockton on Tees

The site was excavated in 2003 by Tees Archaeology ahead of major alterations to a school building. In total eighty three graves were identified and c.100 individuals. The cemetery included four double burials and there were a number of finds of chest fittings. The burials were oriented east-west and there was a clear row structure. Four radiocarbon dates were obtained and gave a date range of the 7^th^ to 10^th^ centuries CE. The cemetery continued to the south and west beyond the excavated area and it should be noted that it lies within 200m of the earlier (6^th^ -7^th^ century CE) Norton East Mill cemetery (see below). The site has not been published but the archive and finds are held by Tees Archaeology

Provenance Note: Samples from Norton, Bishopsmill were provided by Tees Archaeology. Permission to analyse was granted to coauthor Tom Booth in 2019.

#### Norton, East Mill, Stockton on Tees

The accidental discovery of a grave with 6th century Anglo-Saxon finds in 1982 led to the survey and excavation of an almost complete Anglo-Saxon cemetery between 1983 and 1985. The excavation was carried out by Cleveland Archaeology Section (now Tees Archaeology). The cemetery contained 120 north-south oriented burials and three cremations accompanied by a range of finds. The finds mainly comprised copper alloy brooches, spearheads, shield bosses and beads with a few higher value objects. The full extent and boundaries of this cemetery were located. The cemetery is dated on the basis of the grave goods to the latter half of the 6^th^ and the beginning of the 7^th^ century. The archive and finds are held by Tees Archaeology. The cemetery is published as, An Anglo-Saxon Cemetery at Norton, Cleveland by Stephen J Sherlock & Martin G Welch, Council for British Archaeology Research Report 82, 1992.

Provenance note: Samples from Norton, East Mill where provided by Tees Archaeology. Permission to analyse was granted to coauthor Tom Booth in 2019.

#### Oakington, South Cambridgeshire, Cambridgeshire

Oakington is a small village in Cambridgeshire, UK, 7 km northwest of Cambridge. It was named Hochinton and Hochintone in the Domesday Book. In 1926, three early Anglo-Saxon burials were identified when pasture land was cultivated for the first time. The cemetery was rediscovered in 1993 and 1994, when 24 graves were excavated by the Cambridge County Council's Archaeological Field Unit[^13^](https://paperpile.com/c/hqnLqQ/skTi8). A further 17 individuals were identified in 2006 and 2007 by CAM ARC. Between 2010 and 2014, 78 further graves were excavated as part of a university research program taking the total to 124 inhumation graves dating between the later fifth and sixth centuries[^14^](https://paperpile.com/c/hqnLqQ/X64wa). Oakington seems to have been organised around a series of key burials and interestingly one of those contained a completely articulated cow buried adjacent to a young woman[^2^](https://paperpile.com/c/hqnLqQ/G8IaM) (Supp. Fig. 1.6). 26 graves were sampled for this project.

Provenance Note: Samples from Oakington where provided by the University of Central Lancashire. Permission to analyse was granted to coauthor Duncan Sayer in 2017.


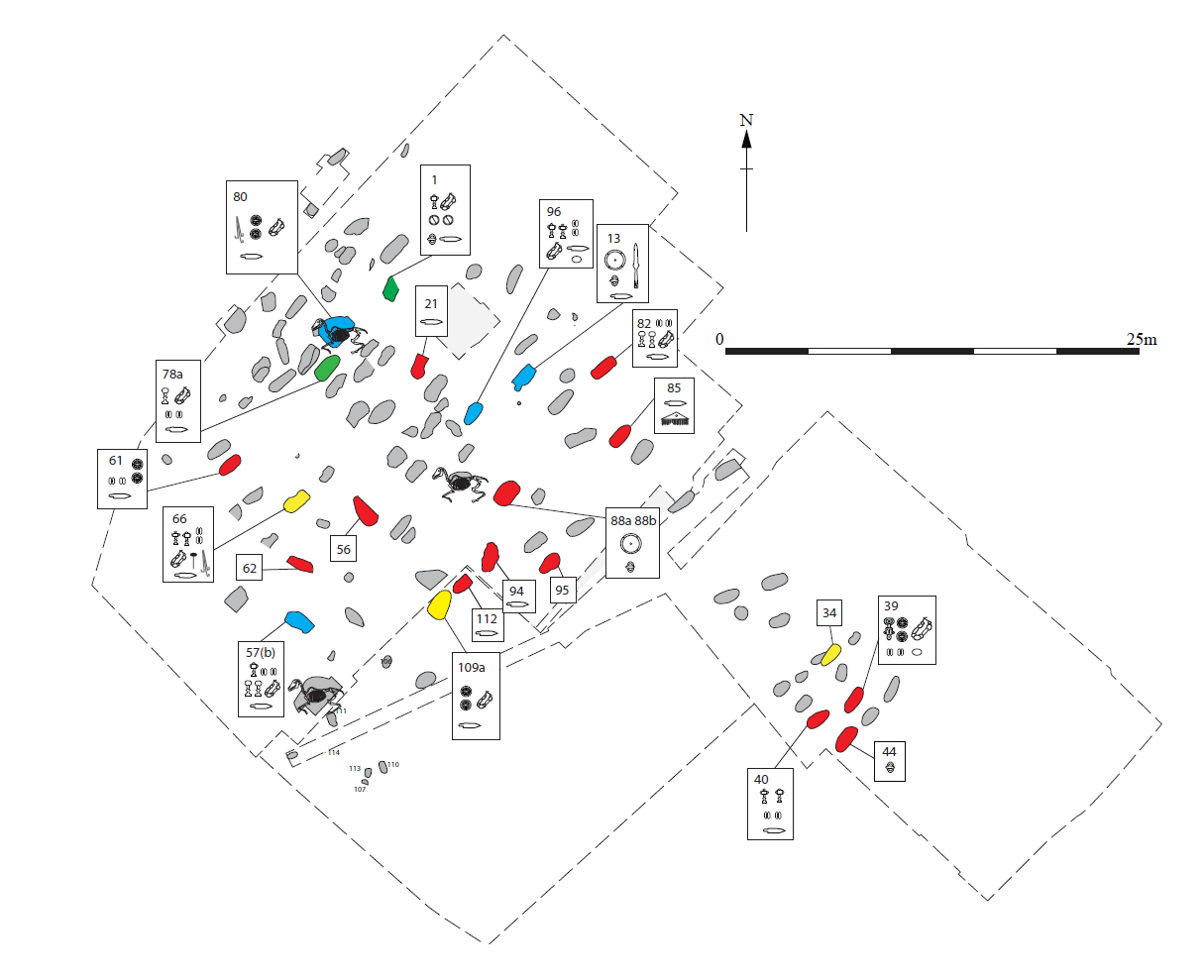


***Supplementary Figure 1.6, Site plan of Oakington.*** *Shown are the locations of the graves, major artifacts in them, the DNA attribution of each grave (red is CNE, yellow 2/3 CNE, green is WBI, blue 2/3 WBI).*

#### Polhill, Maidstone District, Kent

The Anglo-Saxon cemetery at Polhill near Sevenoaks in Kent has been known since the late 19^th^ century, and there were two phases of significant excavation work in 1967, and in 1982 revealing 162 graves. There were 107 remains excavated on or before 1967 and these were largely interred to the west of the site on an west-east orientation. In 1982 a further 51 remains were found to the east, and these were of mixed orientations in what appeared to the excavators to be small clusters, with eight ring ditches evident (Supp. Fig. 1.7). The graves from 1982 were sampled for this project, they are broadly seventh century in date, where the cemetery as a whole probably dates to the seventh and eighth centuries[^15^](https://paperpile.com/c/hqnLqQ/uGHi7).

Provenance Note: Samples from Polhill where provided by the University of Central Lancashire. Permission to analyse was granted to coauthor Duncan Sayer in 2017.

**
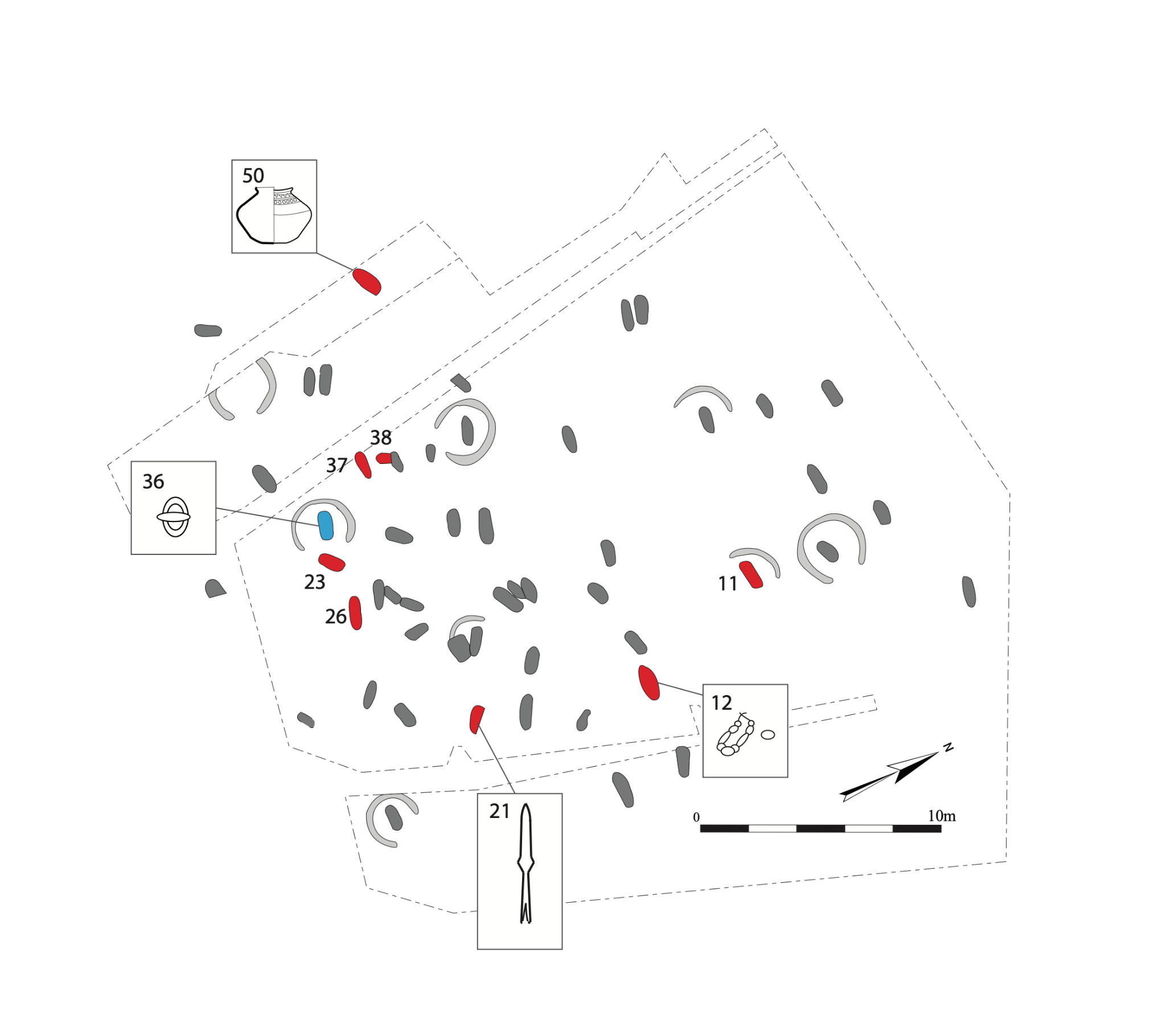
**

***Supplementary Figure 1.7, Site plan of Polhill.*** *Shown are the locations of the graves, major artifacts in them, the DNA attribution of each grave (red is CNE, yellow 2/3 CNE, green is WBI, blue 2/3 WBI). Orange borders denote biologically related individuals.*

#### Rookery Hill, Bishopstone, East Sussex

Rookery Hill is located in the region of East Sussex, on the dip slope of the South Downs, a range of chalk hills found in south-eastern England. The site was occupied intermittently between 5000 BCE to 600 CE. It comprised a settlement and associated cemetery both located on a south-facing hillside and surrounded by low lying alluvial land formed by the River Ouse tidal estuary[^16^](https://paperpile.com/c/hqnLqQ/ZKupT). The site was discovered by chance in September 1967 when a group of builders working for an estate of bungalows disturbed a group of human bones on the west side of the hill. These were reported to the Sussex Archaeological Society and the rescue excavations commenced in the winter of 1967 and continued until the following March. The excavations revealed a “pagan” Saxon cemetery and the traces of Anglo-Saxon buildings to the northeast of the cemetery. Subsequent excavations were conducted by the Brighton and Hove Archaeological Society that acted as the coordinating body of the 1970, 1972, 1974 and 1975 rescue and research excavations (Ibid).

The settlement comprised rectangular structures and sunken huts and the nucleus of the cemetery appeared to have been organised in and around an earlier Bronze Age round barrow. According to the original records[^16^](https://paperpile.com/c/hqnLqQ/ZKupT), the majority of the 118 burials excavated were graves orientated both north-south and east-west at variable depths and directly into the chalk. Based on contextual and artefactual information the occupation of the cemetery was believed to range from the 5^th^ – 6^th^ centuries CE. The population interred in the cemetery has been the subject of intensive osteoarchaeological and palaeopathological study by the writer (PP) of this summary.

Provenance Note: Samples from Rookery Hill were provided by the Brighton Museum and Art Gallery. Permission to analyse was granted to coauthor Tom Booth in 2019.

#### Sedgeford, King's Lynn and West Norfolk, Norfolk

Between 1996 and 2007, the Sedgeford Historical and Archaeological Research Project (SHARP) excavated part of an early medieval cemetery located at Sedgeford, North-West Norfolk, UK. The site has been known for many years, with the field name ‘Boneyard’ having been in long-standing use and several previous excavations having been carried out. SHARP’s large-area, open-trench excavations revealed nearly 300 discrete, supine burials each with very few, if any grave goods. Most of the burials had been disturbed; for example, by inter-cutting graves, ploughing, ditch digging, colluvial movement, root action or animal burrowing. Therefore, most burials were incomplete and generally, each grave fill included disarticulated bone from one or more other individuals. Several deposits of disarticulated bone that did not appear to be associated with a later burial were also found across the site. It is thought that these may be the result of a need to rebury remains that were disturbed during the later creation of ditches across the site, after the cemetery had gone out of use. ‘Pit 9000’ (SED006), which contained the remains of at least four individuals, is one such example. Soil conditions and skeletal preservation varied across the SHARP trench areas, with the Reeddam area in particular being waterlogged. The full extent of the cemetery has not yet been excavated. Radio-carbon dating of some human remains from the site indicates that the cemetery was in use during the approximate time period of 650 – 850 CE.

Results of osteological analysis of the human remains excavated by SHARP are being prepared for monograph publication, but a summary has been published by the SHARP Team[^17^](https://paperpile.com/c/hqnLqQ/tn7C8). Results so far indicate that there was an approximately even distribution of sexes buried within the cemetery. All age categories are represented, with approximately 10% of burials being categorised as sub-adult. Most individuals died with an age-at-death within the ‘mature adult’ category. Those who lived to an age-at-death within the very old adult category, the majority are estimated to be female. For most individuals, skeletal evidence indicates normal development and robust morphology and very little evidence of any malnutrition. Initial stature estimates suggest approximate mean heights of 177 cm for male individuals and 162 cm for females. There are some isolated case-study examples of individuals with rarer pathologies (such as a urinary stone (S3010, SED003), rheumatoid arthritis and systemic infection (S0044, SED007))[^17^](https://paperpile.com/c/hqnLqQ/tn7C8). However, in general, the skeletal evidence indicates that age-degenerative disease (such as osteo-arthritis) was common within the early medieval Sedgeford population, as were stress or work activity related pathologies or trauma. A small proportion of adult skeletons (*n* = 7) exhibited evidence of cranial, and in most cases post-cranial, trauma consistent with conflict. Six of these individuals were estimated to be male (S0067, S1016, S1018, S1033, S1049; SED014, SED020, SED021, SED018, SED019) and for one individual (S0023), sex was indeterminate. One individual (S0034, SED012) was buried with a layer of oyster shells placed over the body within the grave and the burial of another individual (S0025) was possibly buried associated with a horse. Since 2005, SHARP has also excavated within the field adjacent to Boneyard, an area known as Chalk Pit North East (CNE). Two separate crouched burials have been discovered within this area, approximately 10 metres apart and both minimally disturbed by early medieval settlement activity. The radio-carbon date results indicate that these individuals are Neolithic/Bronze-Age (S8001) and Iron Age (S8002, SED011).

Provenance Note: Samples from Sedgeford were provided by the Sedgeford Historical and Archaeological Research Project (SHARP). Permission to analyse was granted to coauthor Stephan Schiffels in 2017.

#### West Heslerton, Ryedale District, North Yorkshire

The early Anglo-Saxon cemetery at West Heslerton was discovered during sand extraction at Cook & Son, Sand Quarry at West Heslerton on the southern margin of the Vale of Pickering, North Yorkshire during 1977. Following preliminary salvage excavations by Dr John Dent from the Humberside Archaeological Unit in 1977-78, annual excavations, funded as part of the national rescue archaeology programme, administered by English Heritage and its predecessor organisations, were conducted by the Landscape Research Centre, under the direction of Dominic Powlesland, until 1986. The cemetery was one component of a large-scale multi-period excavation undertaken ahead of mineral extraction. The cemetery, the most completely excavated example in Yorkshire, was fully excavated to its boundaries apart from a strip c. 30m wide, where a major trunk road - the A64 - bisected the cemetery (Supp. Fig. 1.8). Evidence observed during excavation of a pipe trench on the northern side of the A64 indicates that intact graves most likely still survive beneath the road. 185 graves - containing 188 inhumations - and 15 cremations were excavated, and it is estimated that up to 170 graves may remain beneath the A64 giving a potential cemetery population of 300 - 350 individuals buried between the late fifth and middle of the seventh centuries. The Anglian cemetery was organized upon a prehistoric monument complex, which was used to divide and focus aspects of the site. Such a relationship is evident in many early Anglo-Saxon cemeteries, but particularly notable in West Heslerton because there appears to be a structure to the furnished graves (weapon graves in particular) which is connected to the prehistoric barrows. The Early Anglo-Saxon population from West Heslerton remains the subject of ongoing research.

Provenance Note: Samples from West Heslerton were provided by the Landscape Research Centre. Permission to analyse was granted to coauthors Martin Richards and Ceiridwen Edwards in 2016.


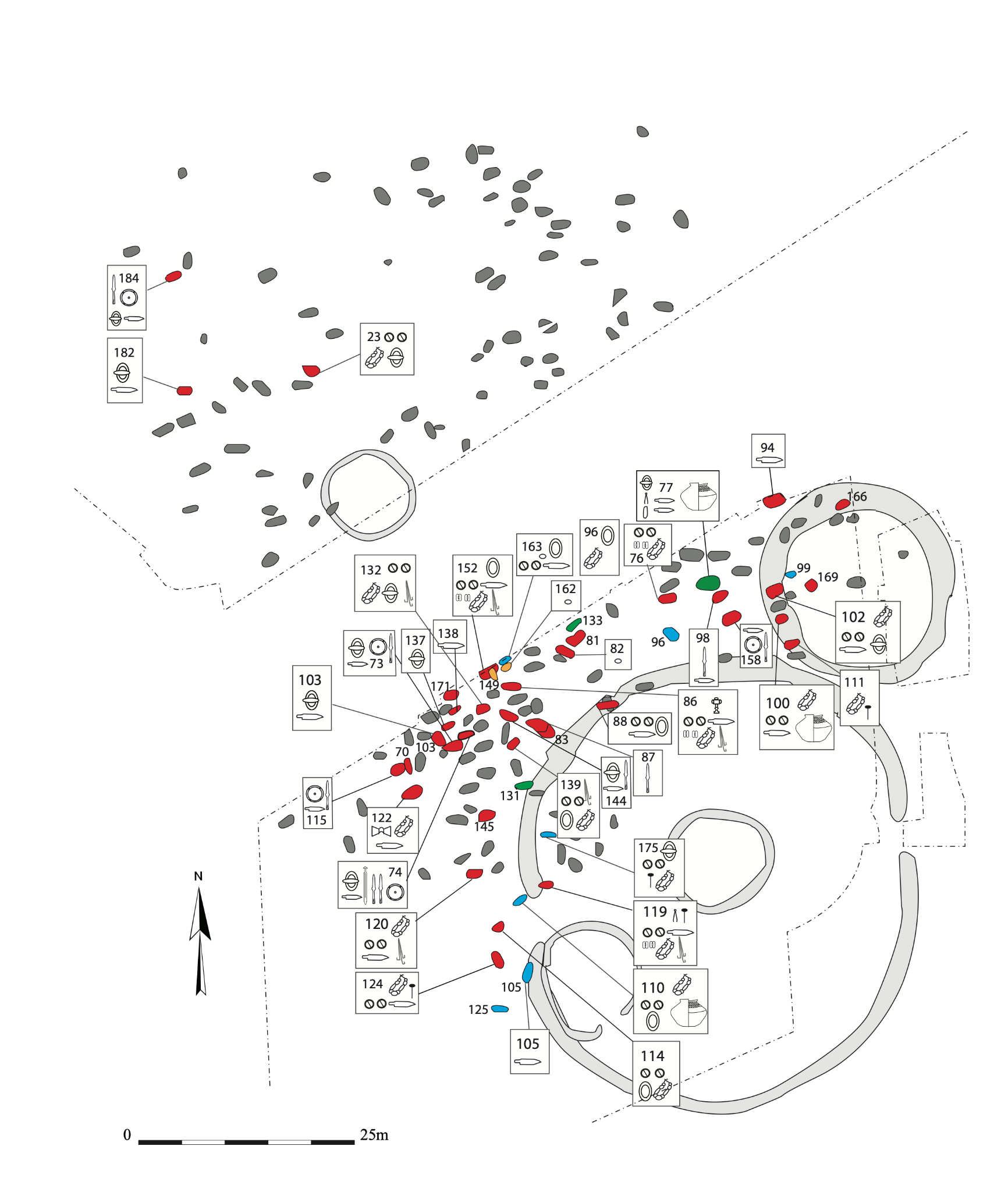


***Supplementary Figure 1.8, Site plan of West Heslerton.*** *Shown are the locations of the graves, major artifacts in them, the DNA attribution of each grave (red is CNE, yellow 2/3 CNE, green is WBI, blue 2/3 WBI). Orange borders denote biologically related individuals.*

#### Wolverton, Radcliffe School Mid-Saxon Cemetery, Borough of Milton Keynes

The Archaeological Services & Consultancy Ltd excavated two sites on the Radcliffe School campus prior to redevelopment. The excavation was commissioned and funded by the Milton Keynes Council, and the report was published by Bancroft Heritage Services. One of the two sites on the campus contained an extensive Saxon cemetery, dating from the 7^th^-8^th^ centuries CE. The cemetery contained 81 inhumations, 2 urned cremations, and one un-urned cremation. The site is the largest mid-Saxon cemetery found in Buckinghamshire so far, and probably served the nearby Saxon settlement at Wolverton Turn Enclosure. 74% of the burials contained skeletons that were 50-100% complete, allowing 60 of the 81 inhumations to be used for pathological analysis. While there were a high number of fairly complete skeletons in good condition, they were very fragmented, with a number of breaks. 59% of the skeletons were adults. There were a number of burial positions present, with supine being the most common (66%). Some unusual burial practices were observed, including one adult with no grave goods whose head had been removed and placed on the left side of the chest, while the mandible was left in situ. There were also two cases of deliberate removal of inhumations, as well as three double burials. 46 of the 81 inhumations were found with grave goods, the most common of those being an iron knife. Other grave goods included complete ceramic vessels, copper objects, spears, weights, scales, tweezers, and flints. Six individuals were sampled for radiocarbon dating and four for DNA analysis. The radiocarbon dating from the individuals indicates that the cemetery was in use between 550 and 720 CE.

SK2348 was identified as a 36-50 year old male who was found in a double burial with charnel present. SK2182 was identified as a 13-20 year old of undetermined sex, found supine with a spearhead, knife, buckle and plate, and nails. SK2087 was identified as a 21-35 year old female, also buried in a supine position with a spindle whorl. Lastly, SK2167 was identified as a 13-20 year old of undetermined sex, who was buried prone with a large amount of burial goods including a necklace, pin, shears, chatelaine, box fittings, and workbox with textile and an inscription.

Provenance Note: Samples from Wolverton were provided by the Milton Keynes Museum. Permission to analyse was granted to coauthor Tom Booth in 2015.

#### Worth Matravers, Football Field, Dorset

Worth Matravers is a multi-period hilltop site with activity stretching back to the early Neolithic. Located close to Chapman’s Pool on the south-central coast of England. In 2011, a small inhumation cemetery of 21 graves, containing 26 individuals, was excavated by volunteers from the East Dorset Antiquarian Society prior to a community housing development[^18^](https://paperpile.com/c/hqnLqQ/tBFMI).

The rectangular cemetery was approximately 17m by 13m and underlay one of the proposed houses. The graves were laid out in six short rows and comprised one triple burial (1685), three double burials (1633, 1678 and 1722) and 17 single burials (of which nine were analysed here) all placed on their backs with heads to the west. There were eleven probable females, four probable males and seven of indeterminate sex. Ages ranged from 5 years to 45-49 years. Six grave types were identified, five of which incorporated local limestone as grave furniture, some of this derived from collapsed Roman buildings, and the sixth being a simple earth-cut type. There was no evidence for coffins and the bodies had probably been shrouded. There were only two grave goods – a stone anchor placed with a male from a double burial (grave 1633), and a small 7^th^ century copper alloy buckle buried with a female (grave 1667).

Bone samples from seven individuals were submitted to the Scottish Universities Environmental Research Centre (SUERC) for radiocarbon dating. The results confirmed that the cemetery was in use from cal CE 540-675 (95% probability) to cal CE 665-790 (95% probability)[^18^](https://paperpile.com/c/hqnLqQ/tBFMI).

Provenance Note: Samples from Worth Matravers where provided by the East Dorset Antiquarian Society. Permission to analyse was granted to coauthors Martin Richards and Ceiridwen Edwards in 2012.

## Ireland

#### Kilteasheen, The Bishop’s Seat, County Roscommon

Located in the north of County Roscommon, on high ground overlooking Lough Key and the Boyle River, the Bishop’s Seat comprises a large (100m+) D-shaped earthen enclosure (RMP RO006:12/13). Within the enclosure are the remains of a 12^th^ century church and an adjacent burial ground. Just outside the enclosure, the low walls of a small fortified structure survive, interpreted as the “Bishop’s Palace”, erected by the Bishop of Elphin in 1253 CE. Historically, the site is also associated with the O’Conor kings of Connacht, particularly in the 13^th^ century. Excavations at Kilteasheen took place over 5 field seasons, from 2005-2009 (Excavation License 05E0531) and was funded by the Royal Irish Academy. A total of 12 cuttings were excavated with 4 targeting the cemetery. From these 4 cuttings, almost 150 skeletons were removed from an estimated burial population of 2500-3000. From a number of radiocarbon dates and associated artefacts, it is clear the burials were interred over a 700-year timespan from 600 CE to 1300 CE. The site also has a significant prehistoric component, with hundreds of lithicse recovered from all contexts and surrounding fields, dating primarily to the Early Bronze Age, 2400 BCE-1500 CE.

Provenance Note: Samples from Kilteasheen were provided by the Institute of Technology Sligo. Permission to analyse was granted to coauthor Kirsten Bos in 2013.

## Netherlands

#### Groningen, Groningen

Early medieval Groningen was a rural settlement that was transformed into a Carolingian royal domain with a church in the 9^th^ century, due to its strategic position at the border of the Frisian coastal districts. There is archaeological evidence for a marketplace since the 11^th^ or 12^th^ century, but possibly even earlier. This indicates the economic importance of Groningen for the area, which is further stressed by the many Groningen coins from the 11^th^ century found elsewhere, such as present-day Scandinavia and Russia[^19,20^](https://paperpile.com/c/hqnLqQ/ZQhag+L0Ztk). The churchyard of the Martini church in the center of the city was the only available cemetery in early and late medieval Groningen. During several excavation campaigns (GMK(96)) parts of the cemetery south-east of the present-day Martini church were excavated, recovering more than 500 graves from the Early and Late Middle Ages and Early Modern Period. The oldest recovered burials from this location are dated in the 8^th^ century CE and this cemetery was in use until 1827 CE[^21^](https://paperpile.com/c/hqnLqQ/1G4IX). There are relatively few non-adults (± 15%) and women (± 30%) among the early medieval burials, compared to what would be expected for a regular farming community. It is not clear if this is representative for this population or if age and sex was originally not equally distributed across the cemetery and a bias was introduced by choice of excavation location[^22^](https://paperpile.com/c/hqnLqQ/4RbPH). Strontium and oxygen isotope analysis was carried out on all individuals investigated here, except GMK96_V079 and GMK_V0592. Six individuals were likely migrants based on their values (GMK_V0604, V0773, V0805, V1053, V1253 and V1254)[^23^](https://paperpile.com/c/hqnLqQ/e8UES).

Provenance Note: Samples from Groningen were provided by the Gemeente Groningen. Permission to analyse was granted to coauthor Eveline Altena in 2017.

#### Midlum, Friesland

Archaeological contract company RAAP excavated a small early medieval graveyard in 2016 in the northern province of Friesland in the Netherlands (HAMZ3). The site is located along the coastline of the Wadden Sea in the area of Westergo, where most inhabitants left when the Roman empire collapsed in the 4^th^ century. Although the Romans never occupied this northern part of the Netherlands - they did not get further north than the Rhine - they were of great economic importance for this area. The area was repopulated in the 5^th^ century. The graveyard is dated to the 5^th^ and 6^th^ centuries and the people buried there can therefore be considered as new settlers, but despite multiple known inhabited sites (terps) nearby, it is not clear where they lived[^24^](https://paperpile.com/c/hqnLqQ/hRn6w). The graveyard consisted of eight inhumations of which several were disturbed to some extent. They contained the remains of six adult females (ind 1, 2, 3, 5, 6, 7), one 10-14-year-old female (ind 4) and one adolescent/young adult that was possibly male (ind 8). Additionally, a pit with secondary human bones of at least two individuals and animal bones was found (feature S140). Some of these secondary bones could belong to the primary inhumations[^25^](https://paperpile.com/c/hqnLqQ/13R3k). The primary inhumations were all single graves, but there were two sets of individuals that were buried directly next to each other (ind 2 with ind 3 and ind 4 with 5). All graves were in a north-south orientation, with all adult females facing south, the female child facing north and unclear direction for ind 8. The body position was unclear for ind 8, ind 3 was in prone position and the others were in supine position. The graves of ind 1, 2, 5, and 6 contained personal objects (beads, fibulae, buckle, knife, spindle)[^26^](https://paperpile.com/c/hqnLqQ/2b9vP). Strontium and oxygen isotope analyses were performed on all adult females (ind 1-3, 5-7) and indicated that one of them (ind 3) could have been born local, as her ratios were compatible with the region. The others had ratios that were not compatible with the region, but they can be found elsewhere in the Netherlands[^27^](https://paperpile.com/c/hqnLqQ/XG0p9).

Provenance Note: Samples from Midlum were provided by the Noordelijk Archeologisch Depot. Permission to analyse was granted to coauthor Eveline Altena in 2017.

## Denmark

#### Copenhagen, Sct. Clements, KBM3621, Denmark

In the first half of 2008, archaeologists from the Museum of Copenhagen (Københavns Bymuseum) excavated parts of the churchyard belonging to the medieval church of Sct. Clements in the center of Copenhagen, Denmark. Sct. Clements was a parish church, built in the 11^th^ century in the early years of the town’s existence. The church was taken out of use during the reformation, and demolished completely in the following decades. Today the site of the church and its former churchyard is located in the middle of the shopping street Strøget, close to City Hall Square in central Copenhagen. The area of excavation was located north of the church, comprising a total of 1048 graves. It was apparent that the churchyard had been intensely used during the whole of the medieval period, with up to seven stratigraphic layers of more or less preserved graves, and large amounts of disarticulated bones, stemming from distrubed graves, spread throughout the gravefills.

The buried individuals consisted of women, men and children of all ages, with a slight dominance of women. The mean height of the individuals was smaller than normal for Denmark during the time period, indicating they might have been living under poorer conditions than average. The churchyard showed a variety of burial practices, especially in the early medieval period (1050-1250 CE), for instance different shapes of grave cuts, preparation layers, and round stones placed on different parts of the body of the buried individuals. Other examples of burial practices represented among the sampled individuals are charcoal preparation layers and brick lined grave cuts. The individuals selected for ancient DNA analysis all lived during the early medieval period and comprise a selection of women, men and children. In-depth analyses of the burial population and their burial practices are still ongoing. Together they will shed new light on the life and identity of these early Copenhageners.

Provenance Note: Samples from Copenhagen were provided by the Museum of Copenhagen. Permission to analyse was granted to coauthor Hannes Schroeder in 2019.

## Germany

#### Alt-Inden, North Rhine-Westphalia

The Merovingian graveyard Alt-Inden is located west of Cologne and is hence situated in the brown coal region of the Rhineland. The surface mining is still active in the region and the reason for the characteristic shape of the landscape. This exceptional situation necessitates a special archaeological approach and sometimes complete graveyards and settlements have to be recovered, although generally only 10% of the historic area can be documented and excavated in time before being destroyed by mining activities. The graveyard of Alt-Inden was excavated in 2004 during a campaign supervised by Bernd Päffgen from the Ludwig-Maximilians-University Munich. Of the originally 800 inhumation graves approximately 25% were preserved. The occupancy of the graveyard starts in the 2^nd^ millennium CE and continues until the mid of the 8^th^ millennium CE. The individuals used for the first sampling were selected based on their grave good assemblages by Tobias Albrecht in 2015. These 17 inhumations were found with grave objects associated with male burials. All individuals were classified as adult, aged between 20 to over 60 years. Most of them could be determined as males osteologically. However, the individual buried in grave 579 exhibits skeletal traits of female sex.

Provenance Note: Samples from Alt-Inden were provided by the LVR-Amt für Bodendenkmalpflege im Rheinland. Permission to analyse was granted to coauthor Stephan Schiffels in 2021.

####

#### Hannover-Anderten, Hannover, Lower Saxony

The site was discovered in the first quarter of the 20^th^ century at the village of Anderten which is a part of the city of Hanover today. As a result of dredging work and the construction of a water gate for the *Mittelland-*Canal, human remains as well as medieval objects were initially retrieved. A rescue excavation was subsequently conducted by the Lower Saxony State Museum which captured a section of the cemetery, which is situated on an elevation of limestone marl. The excavations revealed around 130 inhumation graves, some cremations, and some horse burials. Inhumation burials from the late 6^th^ century are only attested by scatter finds which were retrieved by mechanical shovel-excavators. As far as the small number of artefacts tells us the cemetery was used mainly during the 7^th^ and 8^th^ century and was abandoned approximately around the beginning of the 10^th^ century. At Anderten, bone material has been preserved remarkably well, but due to the rarity of grave goods the dating of single burials is challenging. There are indications that some individual burials date to the late 7^th^ century (e.g. Grave 64, ADN013). This dating was subsequently confirmed by radiocarbon-dating in the course of the paleogenetic investigation. Based on comprehensive radiocarbon dating, a general dating span between the late 7^th^ and early 10^th^ century has to be assumed for the burials which were integrated into the DNA-sampling.

Provenance Note: Samples from Hannover-Anderten were provided by the Landesmuseum Hannover. Permission to analyse was granted to coauthor Stephan Schiffels in 2020.

#### Dunum, Wittmund, Lower Saxony

The early medieval cemetery of Dunum (Lskr. Wittmund) was archaeologically examined between 1966 and 1971 during multiple excavation campaigns conducted by the *Niedersächsisches Institut für historische Küstenforschung (NIHK)* under the direction of P. Schmid. The cemetery belongs to the group of so-called Frisian burial places in the south of the North Sea coast area. It is characterised by the contemporaneous usage of both urn and inhumation burials. Founded in the 7^th^ century, the graves of the early occupancy phase were numerously furnished with well-preserved grave goods. They include mainly clothing remains and jewellery but also tools as well as vessels made of ceramics and glass. By contrast, weapons or riding equipment are rare. As in contemporaneous cemeteries, starting in the middle of the 8^th^ century, burials were increasingly furnished without grave goods. This change in burial rite is commonly associated with the adaptation of Christian beliefs. The burial place was finally abandoned in the 10^th^ century. During the last decade, the cemetery of Dunum was frequently subject of archaeological investigations. Preliminary reports of the excavator[^28–33^](https://paperpile.com/c/hqnLqQ/FlXoC+1kMDv+bjvX4+dbi4A+eB8OS+mzebk) based on selected material and artefact groups interpreted the area as cemetery of five separately buried farm communities[^34–39^](https://paperpile.com/c/hqnLqQ/7mnhI+zChYj+6aRU5+q00ad+RgQj9+9nG37). In the last years, the Deutsche Forschungsgemeinschaft (DFG) financed a project involving the systematic analysis and evaluation of all findings of the cemetery as well as landscape archaeological investigation of the surrounding settlement area[^37^](https://paperpile.com/c/hqnLqQ/q00ad). Within the project, C^14^ dating of organic remains was conducted, providing support for the results of the older dating approaches. Further, extensive anthropological investigations were performed for the first time, although only a small fraction of the buried population was available for analysis due to the limited bone preservation. To this day, individual data of only 52 cremation remains and 20 inhumation burials without grave goods are available, which date into the last occupancy phase during the 10^th^ century[^40^](https://paperpile.com/c/hqnLqQ/hAGdN). The analysed cremation remains are extremely fragmented and show remarkable preservation conditions[^41,42^](https://paperpile.com/c/hqnLqQ/1hpVp+PMaX0). This observation indicates that the still glowing pyres were extinguished using a fluid, possibly to collect and bury the burned bones sooner[^40^](https://paperpile.com/c/hqnLqQ/hAGdN). Within multiple cremation remains Clinker (molten none) was found, demonstrating burning temperatures above 800 degrees Celsius[^43^](https://paperpile.com/c/hqnLqQ/8BEum). Such temperatures could only be achieved using high-quality oak wood, a conclusion which was also verified by material analysis. Further, animal bones were found across the human bone remains. These may either represent food sacrifices or the leftovers of a funeral banquet. Across the individuals selected for aDNA analysis, five discrepancies between osteological and molecular sex were detected. This is not surprising in light of the small sample size and high fragmentation of the bone material. The prevalence of male individuals within the buried population is an important finding and the fundament of future demographic analyses of the local burial community.

Provenance Note: Samples from Dunum were provided by the Niedersächsisches Institut für historische Küstenforschung (NIhK). Permission to analyse was granted to coauthor Stephan Schiffels in 2020.

#### Drantum, Cloppenburg, Lower Saxony

The burial ground at Drantumer Mühle has been known for a long time. It lay directly east of a destroyed megalithic grave, the so-called "Hexenberg". During excavations in 1906, a Franziska, an iron throwing axe, was recovered, which is certainly connected to the burial ground. In 1936, the first inhumation graves were discovered while digging sand, and then a small excavation took place in 1937. Although the site had been a listed building since 1939, sand was still being removed. Finally, the A1 motorway, which was to be built in 1964, led directly across the burial ground. As a result, the findings of the archaeological monument had to be secured by excavation in the endangered area within only four months. 511 inhumation graves, 18 cremation pits or "pyre graves", 24 horse burials and 12 post settlements were documented. The cemetery has certainly not been recorded in its entirety, not to mention the graves destroyed unobserved by sand mining.

Occupation began in the 7^th^ century and ended around the middle of the 9^th^ century. Drantum is one of the cemeteries with mixed occupation, i.e. inhumation and cremation graves occur side by side. In the early phase until the second half of the 8^th^ century cremations were common. In cremation graves, the cremated remains were filled into pits. In pyre graves, the cremated remains were mounded over. From the 7^th^ to the second half of the 8th century, north-south oriented inhumations also occurred. These include horse burials. At the end of the 8th and in the 9^th^ century, burials without grave goods in an east-west orientation appeared in the Christian manner.

Provenance Note: Samples from ​​Drantum were provided by the Landesmuseum Natur und Mensch Oldenburg. Permission to analyse was granted to coauthor Stephan Schiffels in 2020.

#### Häven, district of Kloster Tempzin, Ludwigslust-Parchim, Mecklenburg-Western Pomerania

The site of Häven lies on a sandy hill in the moraine area north of the lake Keetz. Since the second half of the 19^th^ century several graves were discovered at this site due to gravel extraction. The first three graves were discovered in 1868, another three graves came to light in the spring of 1869, one grave was examined in 1872 and in 1875 two further graves were discovered. The discoveries attracted a considerable interest, as the grave goods consisted of a wide range of Roman as well as local artefacts. Comparable grave inventories are known from Southern Scandinavia.

Nearly one century later, gravel extraction led to the find of three further graves in 1967 and 1968. These discoveries led to a preliminary examination in the southern part of the gravel pit, whereby another grave was discovered and excavated in 1971.

Until today 13 graves are known at Häven. Two of these are burials of children. All of the burials are inhumations with different tomb constructions including stone packages and wooden chambers. Based on the inventories, the graves are dated to the 3^rd^ century CE. They can be divided into several spatially separated groups. Similarities in construction and associated grave goods point to a relationship with graves in Zealand, Denmark, as well as at the Central German sites of Haßleben and Leuna.

Provenance Note: Samples from Häven were provided by the Landesamt für Kultur und Denkmalpflege Mecklenburg-Vorpommern. Permission to analyse was granted to coauthor Joscha Gretzinger in 2021.

#### Hiddestorf, Hannover, Lower Saxony

Since excavations from 2008, a pre- and protohistoric settlement north of the town of Hiddestorf, City of Hemmingen, Hanover region, Lower Saxony, Northern Germany, has been known. Rescue excavations were carried out again in 2012, during which further remains of houses, haystacks and wells were found. According to ^14^C-data, there was a settlement continuity between the 1^st^ century BC and the 5^th^/6^th^ century AD. The area covers approx. four hectares and is located on the northern edge of a watercourse with a possible ford situation. The site is furthermore situated in a favourable location in terms of traffic topography lying on the route of the Hellweg, the main prehistoric west-east trade route, in a fertile loess region[^44^](https://paperpile.com/c/hqnLqQ/Lkwvg).

The discovery of a small necropolis with five west-east-oriented inhumation burials and at least one horse grave in the middle of the settlement caused a sensation. Finds and ^14^C-dates show that it was used in the 5^th^/early 6^th^ century. Not only the type of burial – actually cremation graves were common in this region at that time – but also the design of the necropolis and the grave goods are remarkable. A big central chamber grave (Bef. 1995, sample HID001) was surrounded and highlighted by a rectangular wooden palisade. The grave contained a male skeleton with an extraordinary weapon equipment, personal tools and clothing components as well as considerable food offerings and an extensive tableware including several ceramic vessels, a glass drinking bowl and an exclusive bronze hand wash basin. His riding horse was also buried in a wooden chamber a few meters eastern[^45^](https://paperpile.com/c/hqnLqQ/9TLSP). On the other side, western of the central grave, there was a very similar inhumation grave (Bef. 2053, sample HID003). It was only slightly smaller, but also surrounded by a rectangular grave enclosure and contained, among others, an outstanding glass claw beaker. This grave was flanked to the south and to the north by two further burials with rather poor grave goods containing pottery and weapons (Bef. 2050, sample HID002 and Bef. 2055, sample HID004), furthermore it was overlaid by a younger grave without any objects (Bef. 2054, no sample material remained)[^46^](https://paperpile.com/c/hqnLqQ/n9Uaz).

The strictly symmetrical design, the elaborate grave construction, the quantity and quality of the grave goods show that the simultaneous burials followed a conscious hierarchical staging. Burial custom and grave goods show extensive long-distance contacts into the Merovingian realms in the west and to the Kingdom of the Thuringians in the east[^47^](https://paperpile.com/c/hqnLqQ/bGUNw). The continuity of the settlement and the fact that most of the ceramic vessels can be regarded as local production however emphasize the local, northwestern ties. Stable isotope analyses of human teeth (comparison of M1 and M3) and a horse tooth (laserablation MC-ICP mass spectrometry) do indeed proof mobility during lifetime, and they also suggest a common origin for the two individuals 1995 and 2053, but they do not refer to a non-local origin. The small necropolis represents some of the oldest inhumation graves of the region. It reflects the local elite and represents forms of allegiance systems, but it does not represent biologically or socially complete societies – the majority of the population adhered to the traditional cremation customs for several further generations.

Provenance Note: Samples from Hiddestorf were provided by the Landesmuseum Hannover. Permission to analyse was granted to coauthor Stephan Schiffels in 2020.

#### Issendorf, district of Stade, Lower Saxony

The cemetery at Issendorf was discovered in the first half of the 18^th^ century and had been selectively excavated since then. Regular archaeological excavations were conducted in several campaigns from the 1960s to the 1990s culminating in a research project at the Lower Saxony State Museum. During the different field activities more than 5000 cremation urns, around 20 remains of pyres and 79 inhumation graves were discovered. The cemetery is situated on a shallow elevation and reuses the area around a megalithic tomb dating to the Neolithic. With an extrapolated number of originally more than 6000 cremations it is one of the largest sites of the migration period in the so-called Elbe-Weser triangle. According to the artefact assemblages the burials are dated to the period between the 4^th^ to the 6^th^ century CE. Funeral rites and the material culture show clear similarities to artefacts – especially pottery and brooches – retrieved from early Anglo-Saxon cemeteries in England. The large archaeological potential is somewhat obstructed by the poor preservation of bone at the inhumation graves, resulting from the sandy soil. Thus, only the remains of two individuals could be integrated into this study as sufficient skeletal parts for DNA analysis were scarcely preserved. The burial of a juvenile female (grave 3540, ISS001) which provided sufficient hard tissue for aDNA analysis is dated on the basis of the two deposited cruciform brooches to the second phase of the Issendorf inhumation graves. The calibrated radiocarbon date places the burial in the second half of the 4^th^ century. In contrast, grave 3543 (ISS002) yielded no indicative artefacts, which would allow us to narrow down its dating itself, but according to its location on the cemetery a dating to the second or third phase (approx. 460–510 CE) seems plausible.

Provenance Note: Samples from Issendorf were provided by the Landesmuseum Hannover. Permission to analyse was granted to coauthor Stephan Schiffels in 2020.

#### Liebenau, Nienburg/Weser, Lower Saxony

The site has been known since the early 20^th^ century. The first excavations were conducted following accidental discoveries while sand was extracted for a brick manufacture. Following its initial discovery regular excavations were subsequently conducted and led to a long-term research project at the Lower Saxony State Museum. The cemetery is situated on an inland dune and was in use from the 4^th^ to the 9^th^ century CE. The excavations yielded a total of 522 graves, among them 143 inhumations as well as few horse and dog burials. The cemetery at Liebenau is an exceptional source for early medieval archaeology in northern Central Germany because of its long usage and the presence of richly furnished inhumation and cremation burials. According to the artefacts these assemblages mirror the integration of the population into different supraregional networks. During the early phases of the cemetery, assemblages show similarities to the Elbe-Weser-triangle and Early Anglo Saxon England, whilst in the later phases other connections preponderate. The preservation of bone is predominantly poor and fragments which are significant for DNA analysis were usually decayed. Despite this limitation by taphonomy, a wide spectrum of chronologically distinguishable burials was chosen for this study. These burials are primarily dated by brooch types: graves P9/A1 and N12/A2 (LBU009 and LBU002 approx. 400–450 CE), M11/A4 (LBU003 approx. 450–500 CE), H12/A4 and H12/A6 (LBU005 and LBU008 approx. 550–600 CE), J11/A2 (LBU001 approx. 600–650 CE), H11/A1 (LBU004 approx. 700–750 CE). The individuals from graves L12/A1(LBU007), J15/A1 (LBU010) lacked indicative artefacts. This is equally true for H9/A3 (LBU006) which could be broadly assigned to the 9^th^ century by radiocarbon dating. In summary, a significant selection of well-furnished inhumation burials was integrated into this study, in particular an outstanding burial with the equipment of a gold smith (H12/A6). But aDNA was in most cases too badly preserved for genome-wide analyses.

Provenance Note: Samples from Liebenau were provided by the Landesmuseum Hannover. Permission to analyse was granted to coauthor Stephan Schiffels in 2020.

#### Schleswig, Rathausmarkt, Schleswig-Flensburg, Schleswig-Holstein

Dating to 1070 to 1210 CE, the early medieval city of Schleswig represents one of the best studied sites in Northern Germany due to exceptional preservation conditions[^48^](https://paperpile.com/c/hqnLqQ/VgMMY). Its history is intrinsically tied to the prosperous ‘Viking’ trading centre Haithabu (8^th^ century to 1066 CE). Both settlements coexisted shortly and represent notable consecutive trading centres characterised by a cultural transition[^48–50^](https://paperpile.com/c/hqnLqQ/gnf6T+VgMMY+C8xjE). Strategically located at the connecting transit corridor between North and Baltic Sea, the Schlei Fjord enabled the surveillance of an extensive network of trading routes, controlling and exchanging raw materials and goods across borders[^51,52^](https://paperpile.com/c/hqnLqQ/IbNtM+zSSa7). Pavement workings in 1982 opened the possibility to rescue-excavate the market square (Rathausmarkt) of Schleswig, as part of the excavation program in the old city centre from 1964 to 1984[^48^](https://paperpile.com/c/hqnLqQ/VgMMY). An area of 250 m² was excavated, revealing foundations of a former church and a densely occupied Christian graveyard with complex stratigraphy. At a depth of 3 m, 240 graves were found, comprising 259 skeletons. High groundwater levels and a 2 m thick dung layer created humid and nearly anaerobic conditions leading to a remarkable preservation of wooden coffins and soft tissues of the buried individuals. Dendrochronological analyses of 28 wooden coffins resulted in absolute dates (1082 to 1205 CE). A full range of extensive bioarchaological analyses have been conducted so far, e.g. osteological, soft tissue and decomposition analyses[^48^](https://paperpile.com/c/hqnLqQ/VgMMY). A young adult female was buried in a unique mixed tuff-brick grave (Grave 100) which could be stratigraphically dated to 1180-1190 CE. While brick was locally available from 1160 onwards, tuff had to be imported from the Rhineland. The grave construction (Grave 214) of an adult man (22-33 years) is singular among the graveyard as his wooden coffin was placed on a bed of charcoal. Similar burial contexts have been reported from early medieval cemeteries in Lund, Great Britain and France[^48,53^](https://paperpile.com/c/hqnLqQ/VgMMY+0JrfH).

Provenance Note: Samples from Schleswig were provided by the Stiftung Schleswig-Holsteinische Landesmuseen Schloss Gottorf. Permission to analyse was granted to coauthor Ben Krause-Kyora in 2017.

####

#### Schortens, Friesland, Lower Saxony

Between the geest and the marsh in the coastal area of the North Sea lies one of the largest burial grounds of northern Germany. It was first noticed in 1938, when four cremation graves and a sword were found during the construction work for a new house in the south of the town Schortens and brought to the museum in Jever Castle. After this initial discovery, and with the transformation of previously unused farmland into building terrain in the following years, many more artifacts and urns were excavated unobserved. It was not until 1957 that an official survey was organized. A total of 15 inhumation and cremation graves with glass beads, needles, knives and pottery were found. Almost 20 years later - again initiated by the construction of another house - the last and largest excavation took place. Due to the building situation the excavation had to be carried out in the homeowner’s gardens, but almost 2880m^2^ (from originally about 6400m^2^) could be examined with more than 700 inhumation and cremation graves dating between the 5^th^ and the 9^th^ century. Since the sandy bottom of the geest hardly allows bone to be preserved, only corpse shadows could be found in most of the body graves. Nevertheless, it was possible to recover some bone fragments from four graves. The four sampled remains (85, 87, 88 and 455) are all from inhumation graves excavated in 1974. The graves 85, 87 and 88 lie in a row with grave number 86 in between. There are no artifacts, but contextually the graves can be dated to the late 8^th^ or early 9^th^ century. The last sample from grave 455 also dated to the 9^th^ century and belongs to a nearby row of 18 burials all facing west-east orientation and without grave goods. Although the graves cannot be archaeological assigned to any gender due to the lack of artifacts, DNA tests showed that all four were biologically male individuals and related to each other.

Provenance Note: Samples from Schortens were provided by the Landesmuseum Natur und Mensch Oldenburg. Permission to analyse was granted to coauthor Stephan Schiffels in 2020.

#### Zetel, Friesland, Lower Saxony

The mixed "Frisian" cemetery of Zetel was almost completely excavated from 1957 to 1964. 720 graves, 60 of which are cremations, date from the first half of the 7^th^ century to the second half of the 9^th^ century. An overall five-phase occupation of the cemetery was found. The first phase comprises mounded cremation burials, the second phase cremation graves and north-facing inhumations, which predominate in phases three and four. In the fifth phase the occupation ends with burials without grave goods oriented west-east. In addition to cremation pit graves, which are also found in Saxon regions, urn graves and bone storages with rest of pyre, were found in Zetel. Both types of burial occur almost exclusively in Frisian settlement areas and are considered characteristic of the tribal association. Three cremation burial complexes were originally mounded over and surrounded by a circular ditch. The burial ground was occupied by groups or clans, i.e. burials of the same period were located in different places and then formed the starting point for further occupation. From the middle of the 8^th^ century, the first south-north oriented inhumation graves were found in Zetel. At the end of the 8^th^/beginning of the 9^th^ century, east-west oriented burials were found. The burials without grave goods make up the bulk of this cemetery (approx. 80 %). The "Frisian" cemeteries are also subject to a gradual change in burial customs, the origin of which can be seen in the Christianisation pursued by the Frankish Empire. Even if there are no objects with Christian symbolism from Zetel, the change can be documented by the changing burial custom lacking grave goods. Despite forced Christianisation, the inner conversion of the population probably continued for quite a long time, at least until the construction of the first (wooden) church in Zetel in the 10^th^ century and the burial of the deceased at the church.

Provenance Note: Samples from ​​Zetel were provided by the Landesmuseum Natur und Mensch Oldenburg. Permission to analyse was granted to coauthor Stephan Schiffels in 2020.

# Supplementary Note 2 *Reference data*, *kinship, uniparental markers, and potential sex bias in early medieval England*

## Reference data

We compiled one main autosomal reference dataset of microarray SNP data for the purpose of analysing the ancestry of the ancient English and continental samples. We removed present-day individuals with <95% call rate, and loci that failed previous WTCCC2 SNP quality control procedures[^54–56^](https://paperpile.com/c/hqnLqQ/MxYPa+XdvpV+eLLr1). We ran ADMIXTURE with individuals of Asian and African ancestry and filtered out European individuals with <90% European ancestry. Finally, we filtered the reference datasets for related individuals, excluding pairs of first- and second-degree kin relationships and samples that failed WTCCC2 sample quality control procedures[^54–56^](https://paperpile.com/c/hqnLqQ/MxYPa+XdvpV+eLLr1). The remaining intersection of the autosomal SNPs were subsequently pruned for loci on three previously reported[^57–60^](https://paperpile.com/c/hqnLqQ/0fg8B+jJsdH+P78rj+tbeMT) long-range LD regions on chromosomes 6, 8 and 11 using PLINK[^61^](https://paperpile.com/c/hqnLqQ/BiJCM). Additionally, we removed a 15 Mb region surrounding the HLA region on chromosome 6 because a fraction of the European samples included multiple sclerosis case samples, a disease with strong HLA associations[^55,56^](https://paperpile.com/c/hqnLqQ/eLLr1+XdvpV). Our final reference dataset contained genotypes for 445,171 SNPs (the intersection of several different Affymetrix and Illumina chip types) from 10,176 contemporary individuals sampled from 45 mostly European populations from previously published datasets[^54–56,62–73^](https://paperpile.com/c/hqnLqQ/XdvpV+MxYPa+eLLr1+6zgL3+tJLVT+eNteK+3aPPV+boRxi+j8W3L+D6a2V+oRczI+E47th+fu8UO+QtLQE+RcjO):

- Norway (921)
- Sweden (1,110)
- Denmark (314)
- England (1,678)
- Scotland, excluding Orkney (91)
- Orkney (92)
- Wales (129)
- Northern Ireland (115)
- Ireland (332)
- Germany (1,093)
- Netherlands (498)
- Belgium (538)
- France (495)
- Spain (211)
- Italy (776)
- Finland (779)
- Estonia (6)
- Latvia (9)
- Lithuania (438)
- Belarus (21)
- Poland (80)
- Ukraine (27)
- Hungary (22)
- Slovakia (15)
- Slovenia (15)
- Romania (16)
- Moldavia (9)
- Bulgaria (13)
- Albania (3)
- Kosovo (9)
- Macedonia (14)
- Montenegro (14)
- Bosnia (15)
- Croatia (28)
- Serbia (18)
- Greece (36)
- Jordan (22)
- Morocco (10)
- Saami (3)
- Ingrian Finns (3)
- Tuscans from Italy (108)
- Northern Europeans from Utah (99)
- Yoruba in Ibadan (108)
- Han Chinese in Beijing (103)

This includes relevant populations from Great Britain and Ireland, as well as populations from the Netherlands, Germany, and Denmark). Unfortunately, while our reference dataset includes 1,093 present-day Germans, regional origin data reported in Leslie et al. 2015[^56^](https://paperpile.com/c/hqnLqQ/eLLr1) for those individuals was not available to us. This is limiting, since there is strong regional genetic differentiation across the German population, especially along an east-to-west and a north-to-south cline[^74–76^](https://paperpile.com/c/hqnLqQ/D9KYI+gWBuy+owf11) and the addressed German reference data was collected in three centres (Munich, Berlin, and Hamburg)[^55^](https://paperpile.com/c/hqnLqQ/XdvpV), covering most of the present-day German genetic diversity[^56^](https://paperpile.com/c/hqnLqQ/eLLr1). Treating all German samples as one population would therefore result in the loss of potentially relevant structural information, especially since Lower Saxony is assumed to be one of the emigration areas to England during the early medieval period studied here, and similarly Schleswig-Holstein (historical area of the historic group of the Angles), which highlights the relevance of the present-day northern German population as an important reference in the analysis of medieval samples from England and Lower Saxony. In theory, the northern German sampling was rural and population-based with the majority of samples recruited through out-patient (ambulatory) clinics and a minority recruited from neurological wards, especially around Hamburg. To account for this issue, we used a genetic approach to identify northern Germans. We used the unpublished genomes of seven German individuals, genotyped on 631,172 SNPs, for whom geographic origins of all four grandparents were known, in a hierarchical clustering approach using i) the first 10 PCs of Germany-exclusive principal component analysis (PCA) or ii) pairwise identity-by-state (IBS) data (computed in PLINK[^61^](https://paperpile.com/c/hqnLqQ/BiJCM)) of the same dataset to identify an intersection of 92 individuals clustering with the three known northern German genomes within both approaches. While this cannot guarantee that the identified German samples originated from northern Germany, PCA and Uniform Manifold Approximation and Projection (UMAP)[^77^](https://paperpile.com/c/hqnLqQ/GhEK2) indicate that the identified subsample falls indeed within the expected range of present-day Germans from northern Germany (Supplementary figure 2.1).

For the reference data set of contemporary individuals described above, principal component analyses (PCA) were conducted using the smartpca program (v.16000) from the EIGENSOFT (v.6.01) package[^78^](https://paperpile.com/c/hqnLqQ/LPCGB). We classify them as:

1. North Sea PCA

(PCs computed on Ireland, Northern Ireland, Scotland, Orkney, Wales, England, Netherlands, northern Germany, Denmark, Norway, Sweden)

1. Northwestern European PCA

(PCs computed on the above-mentioned North Sea populations plus Belgium, Germany, and France)

1. Northeastern European PCA

(PCs computed on the above-mentioned Northwestern European Populations plus Finland, Estonia, Latvia, Lithuania, Belarus, and Poland)

As observed in previous studies[^79,80^](https://paperpile.com/c/hqnLqQ/lvyLC+e38C6), the resulting plots noticeably resemble geographic maps of Europe. Individuals from the same geographic region cluster together and major populations are distinguishable. Geographically adjacent populations, and recognizable geographical features of Europe such as the Scandinavian peninsula, Britain and Ireland, or the Baltic Sea are apparent (Extended Data Fig. 7). A highly similar pattern emerges from the multidimensional scaling (MDS) visualisation of pairwise *F*_ST_ distances between the present-day populations (Supp. Fig. 2.2).

Consequently, we merged our present-day DNA reference data with previously published datasets of ancient individuals reported by the Reich Lab in the Allen Ancient DNA Resource v.50.0 (<https://reich.hms.harvard.edu/allen-ancient-dna-resource-aadr-downloadable-genotypes-present-day-and-ancient-dna-data>) as well as additional genome-wide data recently reported in Patterson et al 2021[^81^](https://paperpile.com/c/hqnLqQ/3Th9). In doing so, we corrected for reference allele and strand flips. The final dataset includes genome-wide ancient DNA data from 4,336 individuals, mostly from Europe (but also from Greenland, the Near East, and eastern Russia), ranging between the Mesolithic and the early modern Period[^81–145^](https://paperpile.com/c/hqnLqQ/L1lCW+X7M1r+0aHzs+VTvWq+CnCIo+r5iW6+VGqUa+Iw8qF+CxUY7+MyIFY+9O9CJ+I4Koe+eGMso+6OVBv+TA8vQ+gpuQm+whx7F+rN0Dy+B5zdX+n3TTx+5qba3+1MKZc+HE65i+RRCNM+zR5Kn+Ik8SB+yqoEm+cbdfo+knSCH+55V1D+L7WJT+DEho3+AdxKv+4qN2w+gMorQ+7F4ac+IJm1H+aElE1+spmY+jnKBm+ZjbXz+8E2G5+2oozR+1SkHO+e10z3+WPSxx+QVLVp+WGuuL+5danp+tfw6T+veAaL+eyTW6+0gmAb+3Ms3X+kmNjU+I6Awn+2YcIe+ZNX1g+cDAYL+Ag6wX+HNlDt+Gwgbe+pPRlO+2lYj1+3Th9). Population labels correspond to the labels used in the Allen Ancient DNA Resource v.50.0 annotation file.
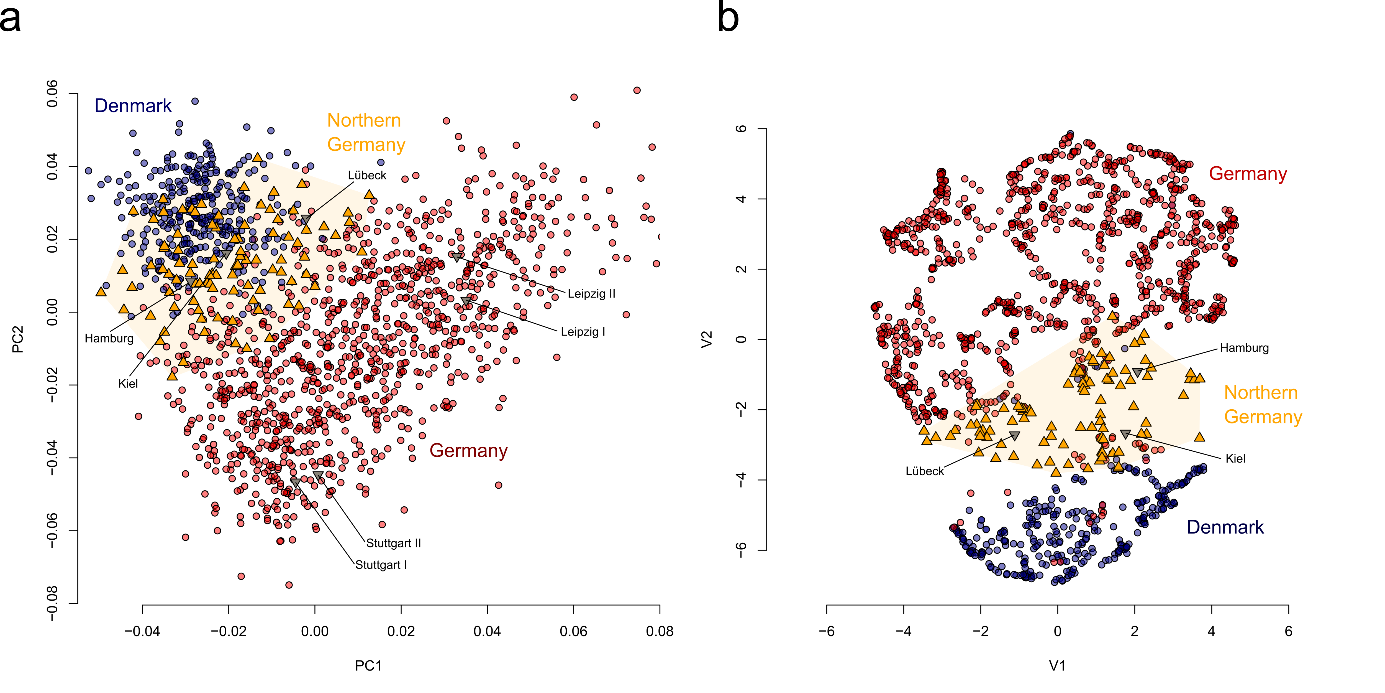


***Supplementary Figure 2.1, Identification of northern German ancestry within the German sample.*** *a) A PCA of present-day Germans and Danes recapitulates geography. The locations of the German genomes of known geographic origin are indicated. Genetically identified northern Germans cluster intermediate between Germans and Danes. b) UMAP of the same dataset.*


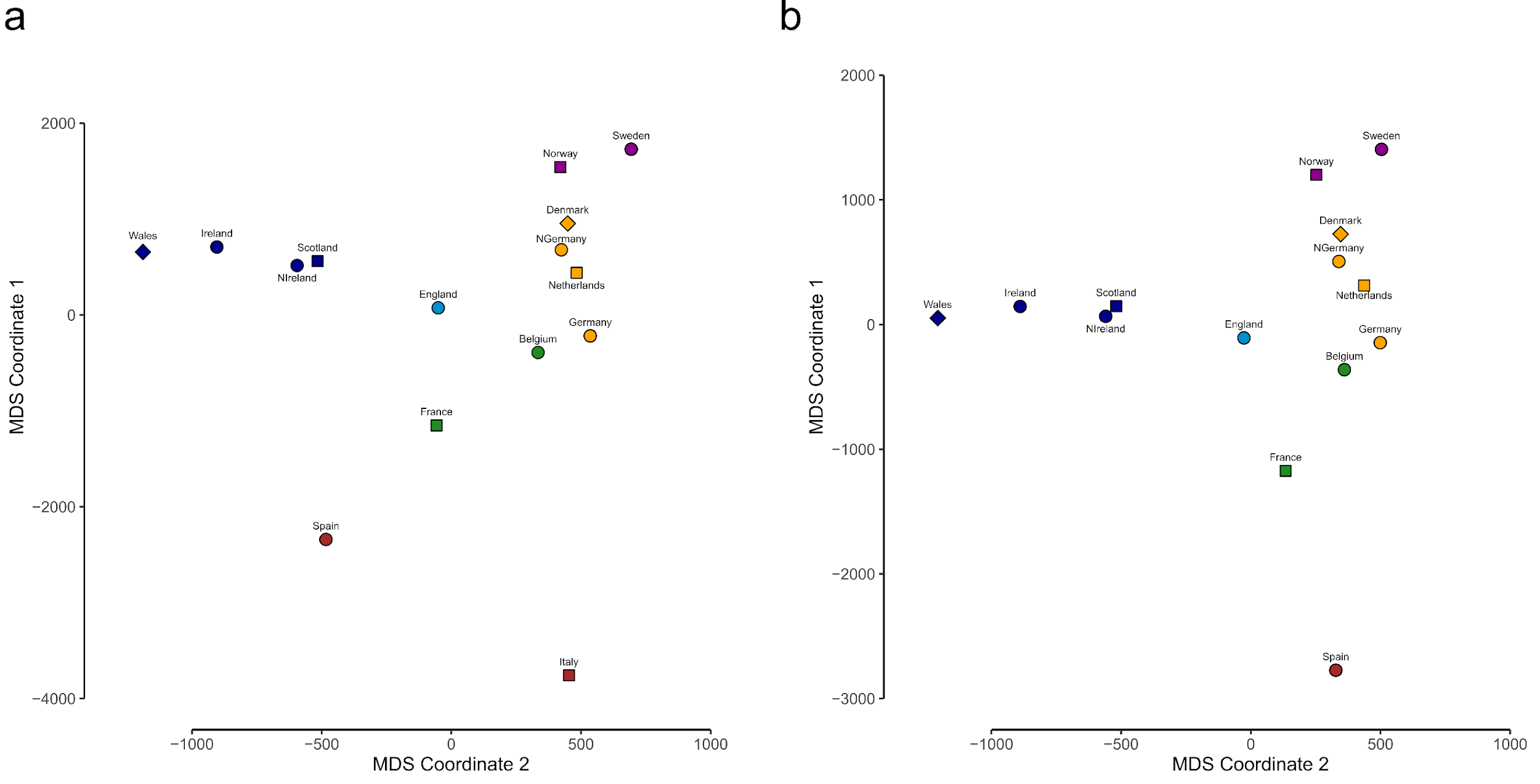


***Supplementary Figure 2.2, Genetic differentiation within the present-day reference dataset.*** *a) Multidimensional scaling (MDS) plot of pairwise F_ST_ distances between 15 European populations. b) Same as a) but excluding Italy.*

## Kinship

We identified several kinship networks (Methods, Supp. Table 1) across several sites in England and the continent. Notable examples include Anderten in Lower Saxony, as well as Dover Buckland, Sedgeford, Lakenheath, Ely, West Heslerton, and Rookery Hill in England. In these cases we compared kin relationships, uniparental markers, and estimates of ancestry components (as described in Supp. Section 2 and Section 3).

*Anderten*. Here we identify four related individuals within one family (ADN001, ADN003, ADN005, and ADN009/ADN010). Three of those individuals are male and carry the same Y-chromosome haplogroup J2b2a1a1, which is comparatively rare in Northern Europe but more frequent in Southern Europe and Southwestern Asia[^146^](https://paperpile.com/c/hqnLqQ/jWTHJ). Based on the mtDNA haplogroups and LcMLkin[^147^](https://paperpile.com/c/hqnLqQ/h5S9S), we infer that ADN001 (mtDNA: H56) is the father of the siblings ADN003, ADN005, and ADN009 (mtDNA: H2a2a1). While we detect a notable proportion of southern European (Italian-like) ancestry in ADN001 (25.6%), his offspring only exhibit small proportions (0–3%). We further conclude that the unsampled mother probably harboured mostly CNE (Danish/Northern German-like) ancestry. We also find two more first-degree related individuals (father ADN013 & daughter ADN011) at the site who show, however, no closer genetic relatedness to the family group mentioned above.

*Sedgeford*. Here we identify four related individuals (SED006, SED018, SED020, and SED021). All four individuals are male: three individuals (SED018, SED020, and SED021) share R1b-P312 haplogroups (R1b1a1b1a1a2), while one individual (SED006) carries an R1a-M420 haplotype (R1a1a1) commonly found in Scandinavia and eastern Europe[^148^](https://paperpile.com/c/hqnLqQ/6hnh9). Interestingly, although R1b-P312 is often associated with the Bronze Age populations of the British-Irish Isles[^87,130^](https://paperpile.com/c/hqnLqQ/r5iW6+5danp), all four individuals exhibit exclusively CNE ancestry. Based on the mtDNA haplogroups and LcMLkin output, we infer that sample SED020 is the father of SED018 (mtDNA: H1b5), and the brother of SED021, who carries mtDNA haplogroup V, which is frequent in northern Scandinavia[^149^](https://paperpile.com/c/hqnLqQ/n85ZY). SED006 neither shares a mtDNA or Y-chromosome haplogroup with the other three samples, but he may be located ancestral to SED020 and SED021 within the pedigree since he is a third–degree relative of both.

*Lakenheath*. Here we identify three related individuals (LAK002, LAK009, and LAK014). Two individuals are male (LAK002 and LAK009), one is female (LAK014). The two males both carry I1a2-Z58 Y-chromosome haplotypes, which are common in Scandinavia and Germanic-speaking Europe[^150^](https://paperpile.com/c/hqnLqQ/gzbxt). Based on the mtDNA haplogroups and LcMLkin output, we infer that LAK009 and LAK014 are siblings, carrying K1a1b2a1a haplotypes. LAK002 is the father of both individuals, exhibiting J1c2c2 as mtDNA haplogroup. Notably, LAK002, as well as his daughter and son, shows exclusively CNE ancestry, which indicates that the unsampled mother did not carry local British ancestry either.

*Ely*. Here we identify four related individuals (ELY001, ELY002, ELY004, and ELY005). Two individuals are male (ELY004 and ELY005) and two are female (ELY001 and ELY002). The two male individuals both carry I1-M253 haplotypes but are probably not paternally related. Instead, they are second–degree related on the maternal line, both carrying U5a1a1 haplotypes, which indicates an uncle–nephew relationship. ELY005 is again a second degree relative of ELY001 (mtDNA: J1c2b), but they do not share the same mtDNA haplogroup. ELY001 is also a third–degree relative of ELY002 (mtDNA: H1bb) but they do not share the same mtDNA haplogroup, thus excluding kinship on the maternal line. While the males exhibit exclusively CNE ancestry, congruent with their northern European Y-chromosome haplogroup, both females exhibit around 50% CNE ancestry (58% and 50%), which may indicate recent admixture with a local British source.

*West Heslerton*. Here we identify a kinship group with nine related individuals. Two individuals are female and seven are male. Both pairs I20649–I20638 and I20638–I11588 have a father–son relationship of unknown order. Since all three males are related on the paternal side, they share the same Y-chromosome haplotype (I1a2-Z58). The remaining individuals exhibit more cryptic second- and third-degree relationships to each other, which cannot be clearly resolved, suggesting a complex kin network in West Heslerton. Notably, they do not share matching mtDNA or Y-chromosome haplogroups. All nine individuals exhibit exclusively CNE ancestry, indicating no admixture with local English sources at all. Two further West Heslerton individuals (I20640 and I20656) which are second– or third–degree related to each other, show no closer genetic relatedness to the family group mentioned above.

*Rookery Hill*. Here we identify a kin group of three related individuals. Two individuals are female and one is male. Based on the mtDNA haplogroups and LcMLkin output, we infer that I14540 is the father of daughter I14541. Both individuals are second– or third–degree related to the female I14534, who does not carry a matching mtDNA haplogroup. All three individuals show exclusively WBI ancestry, which indicates no recent introgression of CNE ancestry into the family complex. We find also two more first–degree related individuals (father I14535 and daughter I14536) at the site who show no closer genetic relatedness to the family group mentioned above.

## Mitochondrial DNA

To analyse possible signals of early medieval immigration through the maternal line of descent, we looked at mitochondrial DNA haplogroup sharing across nine “geo-chronological” groups (Supplementary Figure 2.3). We assembled these by combining our new (*n* = 417) and published (*n* = 909) data (Supp. Table 2.1) with the goal of identifying and differentiating local pre-medieval British mitochondrial lineages from non-local ones.

To this end, we combined samples from Bronze Age and Iron Age Scotland, Ireland, and Wales (Sco_Ire_Wal_BA_IA), with Bronze Age and Iron Age England (Eng_BA_IA), to represent the local mitochondrial gene pool (BritLoc). The North Sea region (NorthSea) was represented by the Bronze Age, Iron Age and early medieval North Sea (NorthSea_BA_IA_EMA), and the later medieval “Viking” North Sea group from Denmark (VK_NorthSea). Taking into consideration the signal of Scandinavian influence that emerged from the genome-wide analyses, we also included the Scandinavian Peninsula (Scand) as a potential secondary source of non-local medieval lineages, here represented by two groups: a small sample of Iron Age and early medieval Scandinavians (ScandP_IA_EMA) and a much larger sample of later medieval Scandinavian “Vikings” from Norway and Sweden (VK_ScandP). We also included small groups of British and Irish “Vikings” (VK_Brit&Ire) and Irish medieval samples (Ire_MA). Finally, we included in the analysis only unrelated individuals on the maternal line of descent, and excluded duplicated individuals present in the dataset.

As discussed above, when we considered the distribution of the major mitochondrial haplogroups across the nine “geo-chronological” groups (Supplementary Figure 2.3), there were no significant patterns or discontinuities that could not be explained by the heterogeneous sample sizes, in contrast to Y-chromosome distributions.


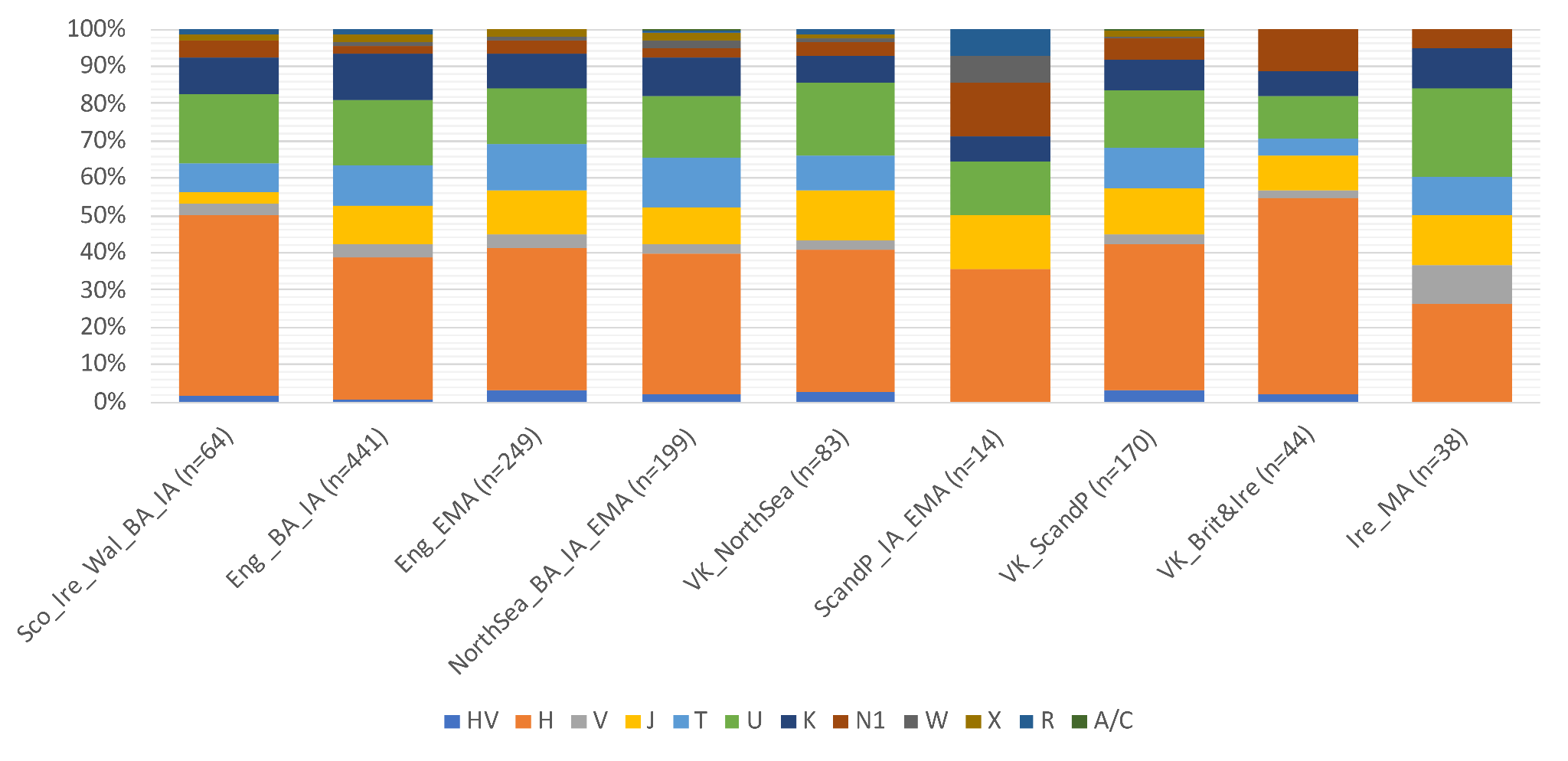


***Supplementary Figure 2.3. mtDNA diversity.*** *Distribution of the major mitochondrial lineages across the nine geo-chronological groups. Counts only include unrelated individuals.*

We then looked at the finest level of sub-haplogroup classification (which we refer to as “lineages”) to identify shared lineages between Eng_EMA and the two putative sources: the continental North Sea region (NorthSea_BA_IA_EMA + VK_NorthSea) and the Scandinavian Peninsula (ScandP_IA_EMA + VK_ScandP).

This approach has obvious limitations: the absence of a single shared lineage within or across regions is not necessarily evidence of absence; likewise, the presence of a shared lineage across regions is not necessarily evidence of a unique genetic link. To minimise the impact of these caveats, we focused our considerations on aggregate patterns involving multiple lineages, and for each potential candidate lineage, we considered, when possible, the phylogenetic relationships between sister/sub-lineages and their sharing pattern.

For example, we labelled as “uncertain” putative shared lineages that were deeply nodal within the haplogroup tree (like T2*) or nested among lineages that had been observed in pre-medieval Britain, such as K2b1a1, shared between Eng_EMA and the Scandinavian group (Supp. Table 2.5), and labelled “uncertain” because the sub-clade K2b1a1a was found in the Eng_BA_IA group. On the other hand, we considered as reliable I1*, shared between Eng_EMA and the NorthSea region (Supp. Table 2.3), despite being nodal, because all I1 lineages we found in the dataset (including I1a1, I1a1a3 and I1a1e) were identified among Vikings from Scandinavia, the North Sea and Britain, and the NorthSea_BA_IA_EMA, while no I1 lineages were detected in Britain BA_IA, or medieval Ireland, even though we did detect I2 lineages (I2*, I2a, and I2a1).

At the finest level of haplogroup sub-classification, Eng_EMA included 176 mitochondrial lineages. Of these, 74 were unique to the sample group, and 57 were shared with the putative British local gene pool (BritLoc), of which 19 were uniquely shared, while 38 were also found among the continental medieval samples (Medieval), here including both the North Sea region and the Scandinavian Peninsula. Eng_EMA shared 83 lineages with the medieval group, of which 38 were also detected in the local British gene pool, while 45 were exclusively shared (Supp. Figure 2.4).


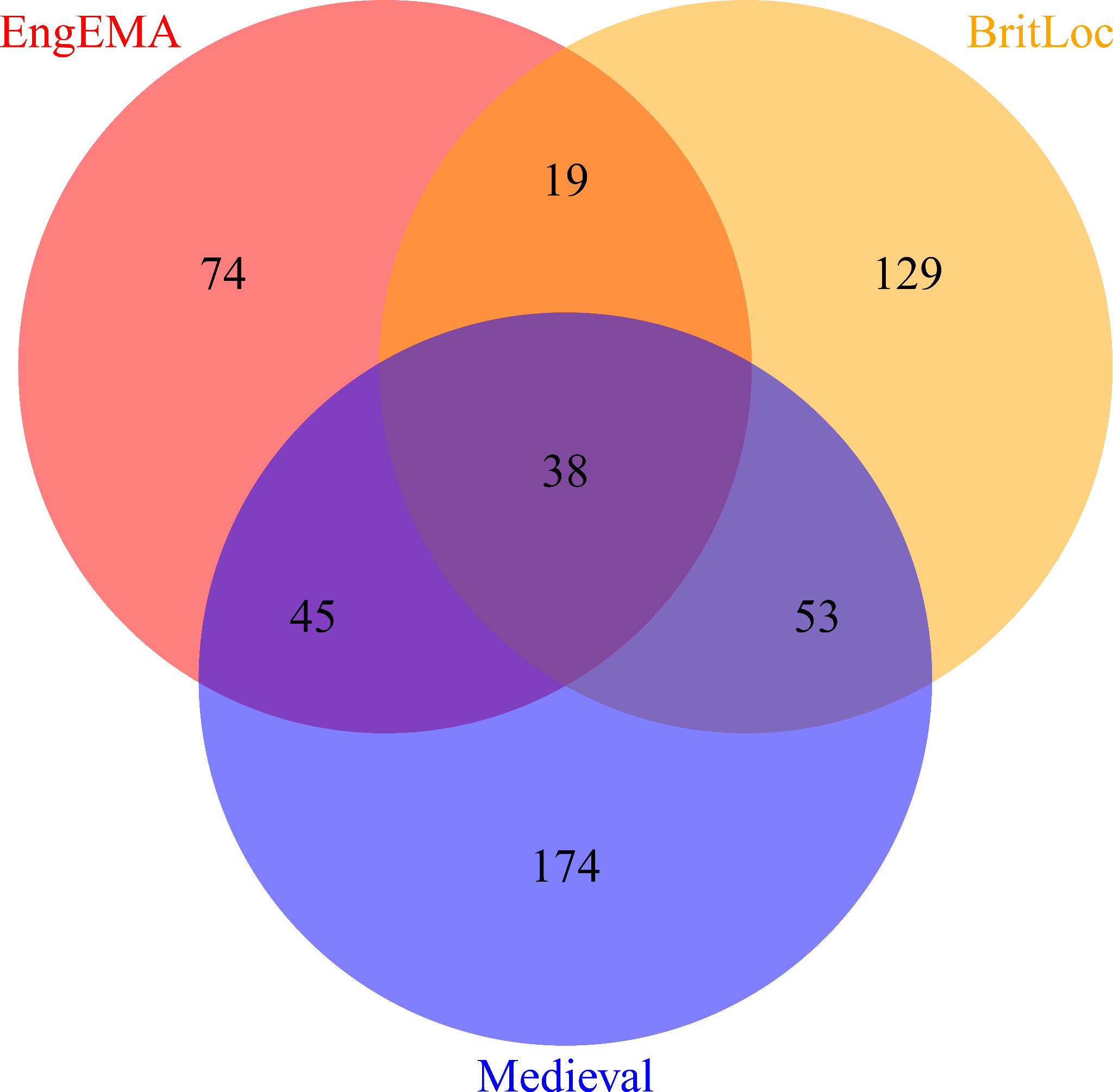


***Supplementary Figure 2.4. Mitochondrial lineage sharing among the three major population groups****.*

Local (Loc.) and continental (Cont.) shared lineages appear with similar frequencies (~30%) across most of the sampled Eng_EMA regions (Supp. Table 2.2 and Supp. Figure 2.5), with local lineages showing consistently higher values. However, 30–40% of the sampled individuals across the various populations carry haplogroups that remain “Unassigned” (Un.). This category includes both lineages that are unique to the populations compared, and thus not shared, and the 38 lineages that are shared among all analysed populations, and which therefore could not be assigned to any single source.

There are, however, local variations to this pattern. This is particularly evident when we look at England East, for which we have several numerically significant sites. Here, we observe a much higher presence of local lineages at Oakington (50% Loc. *vs* 31% Cont. – 19% Un.) and Sedgeford (41% Loc. *vs* 29% Cont. – 29% Un.), and slightly lower values at RAF Lakenheath (20% Loc. *vs* 13% Cont. – 67% Un.), although 67% of the samples from this site carry “Unassigned” lineages. Hatherdene Close on the other hand shows a completely opposite pattern, with a higher presence of non-local lineages (24% Loc. *vs* 47% Cont. – 29% Un.). England North also seems to display a higher frequency of putative non-local lineages (27% Loc. *vs* 35% Cont. – 38% Un.), although this value relies on frequencies observed at a single site, West Heslerton, one of the early Anglo-Saxon cemeteries, where genomic analysis has shown integration of individuals with CNE and WBI ancestry.

*Supp. Table 2.2. Distribution of individuals carrying shared lineages of Local and Continental medieval origin, respectively, across the Eng_EMA, British and Irish Vikings and Ireland MA groups.*

| **Eng_EMA** | ***n*** | **Shared** | | | | | |
| --- | --- | --- | --- | --- | --- | --- | --- |
|  |  | **Brit Loc** | **%^a^** | **Continental** | **%^a^** | **Unassigned** | **%^a^** |
| **England** | **249** | **90** | **36** | **76** | **31** | **83** | **33** |
| **England North** | **55** | **15** | **27** | **19** | **35** | **21** | **38** |
| Clapdale, Fox Holes Cave | 1 | 1 |  |  |  |  |  |
| Hartlepool, Olive Street | 1 |  |  | 1 |  |  |  |
| Norton Bishops Mill | 5 | 2 |  | 2 |  | 1 |  |
| Norton East Mill | 5 |  |  | 1 |  | 4 |  |
| Ribblesdale, Selside Grike | 1 | 1 |  |  |  |  |  |
| West Heslerton | 42 | 11 | 26 | 15 | 36 | 16 | 38 |
| **East Midlands** | **3** | **2** |  |  |  | **1** |  |
| Lincoln, Lincoln Castle | 3 | 2 |  |  |  | 1 |  |
| **England East** | **84** | **30** | **36** | **25** | **30** | **29** | **35** |
| Ely | 5 | 2 |  | 1 |  | 2 |  |
| Hatherdene Close | 17 | 4 | 24 | 8 | 47 | 5 | 29 |
| Hinxton | 3 | 1 |  | 1 |  | 1 |  |
| Linton | 1 |  |  |  |  | 1 |  |
| Oakington | 26 | 13 | 50 | 8 | 31 | 5 | 19 |
| RAF Lakenheath | 15 | 3 | 20 | 2 | 13 | 10 | 67 |
| Sedgeford | 17 | 7 | 41 | 5 | 29 | 5 | 29 |
| **England Southeast** | **92** | **32** | **35** | **30** | **33** | **30** | **33** |
| Apple Down, West Chichester | 9 | 4 |  | 3 |  | 2 |  |
| Dover Buckland | 56 | 19 | 34 | 17 | 30 | 20 | 36 |
| Eastry Updown | 5 | 3 |  | 2 |  |  |  |
| Polhill | 9 | 2 |  | 5 |  | 2 |  |
| Rookery Hill, Bishopstone | 9 | 2 |  | 3 |  | 4 |  |
| Wolverton, Radcliffe School | 4 | 2 |  |  |  | 2 |  |
| **England Southwest** | **15** | **11** | **73** | **2** | **13** | **2** | **13** |
| Bude, Widemouth Bay | 1 |  |  |  |  | 1 |  |
| Newquay, Crantock | 2 | 1 |  |  |  | 1 |  |
| Worth Matravers | 12 | 10 | 83 | 2 | 17 |  |  |
| **Viking_Britain/Ireland** | **44** | **8** | **18** | **6** | **14** | **30** | **68** |
| **England** | **31** | **5** | **16** | **4** | **13** | **22** | **71** |
| **England Southeast** | **21** | **3** | **14** | **2** | **10** | **16** | **76** |
| England, Oxford, St John's College Oxford | 21 | 3 | 14 | 2 | 10 | 16 | 76 |
| **England Southwest** | **10** | **2** | **20** | **2** | **20** | **6** | **60** |
| England, Dorset, Ridgeway Hill Mass Grave | 10 | 2 | 20 | 2 | 20 | 6 | 60 |
| **Scotland** | **8** | **1** |  | **1** |  | **6** |  |
| **Highlands & Islands** | **8** |  |  | **1** |  | **7** |  |
| Orkney, Brough Road Birsay | 3 |  |  | 1 |  | 2 |  |
| Orkney, Buckquoy Birsay | 2 |  |  |  |  | 2 |  |
| Orkney, Newark Deerness | 3 | 1 |  |  |  | 2 |  |
| **Wales** | **1** |  |  |  |  | **1** |  |
| **Anglesey** | **1** |  |  |  |  | **1** |  |
| Glyn Llanbedrgoch | 1 |  |  |  |  | 1 |  |
| **Ireland** | **4** | **2** |  | **1** |  | **1** |  |
| **Connaught** | **1** | **1** |  |  |  |  |  |
| Eyrephort | 1 | 1 |  |  |  |  |  |
| **Leinster** | **3** | **1** |  | **1** |  | **1** |  |
| Finglas | 1 |  |  |  |  | 1 |  |
| Islandbridge | 1 |  |  | 1 |  |  |  |
| Ship Street Great | 1 | 1 |  |  |  |  |  |
| **Ireland_MA** | **38** | **7** | **18** | **3** | **8** | **28** | **74** |
| **Ireland** | **38** | **7** | **18** | **3** | **8** | **28** | **74** |
| **Connaught** | **38** | **7** | **18** | **3** | **8** | **28** | **74** |
| Kilteasheen, The Bishop’s Seat | 38 | 7 | 18 | 3 | 8 | 28 | 74 |
| ^a^ Percentages are displayed only when *n* ≥ 10 |  |  |  |  |  |  |  |

A notable exception to this overall pattern is England Southwest, here mostly represented by a single site, Worth Matravers, where local lineages are carried by 83% of the sampled individuals, while only 17% carry lineages of putative continental medieval origin (there are no Unassigned lineages among the sampled individuals). Note, however, that, uniquely amongst our dataset, Worth Matravers is a post-Roman cemetery that would be expected to have minimal North Sea influence (as reflected in both the genome-wide and Y-chromosome analyses, which indicate ~6% and 18% North Sea impact respectively). Therefore, its extreme position here also broadly corroborates our heuristic approach to the mtDNA analysis whilst, if anything, suggesting that we might be slightly over-estimating the scale of the North Sea fraction.

The group representing the Vikings from Britain and Ireland, mostly represented by the English sample, shows a similar pattern to Eng_EMA, with comparable representation of local *vs* continental haplogroups among its individuals. However, it is important to note that most of the sampled individuals (~71%) carry lineages that remain unassigned (Supp. Figure 2.5).

Medieval Ireland, which appears as carrying almost 100% WBI ancestry according to the genome-wide results, here also shows a higher frequency of local *vs* continental mitochondrial lineages (18% Loc. *vs* 8% Cont.), although, in this case as well, a large proportion (74%) of sampled individuals carry Unassigned lineages.


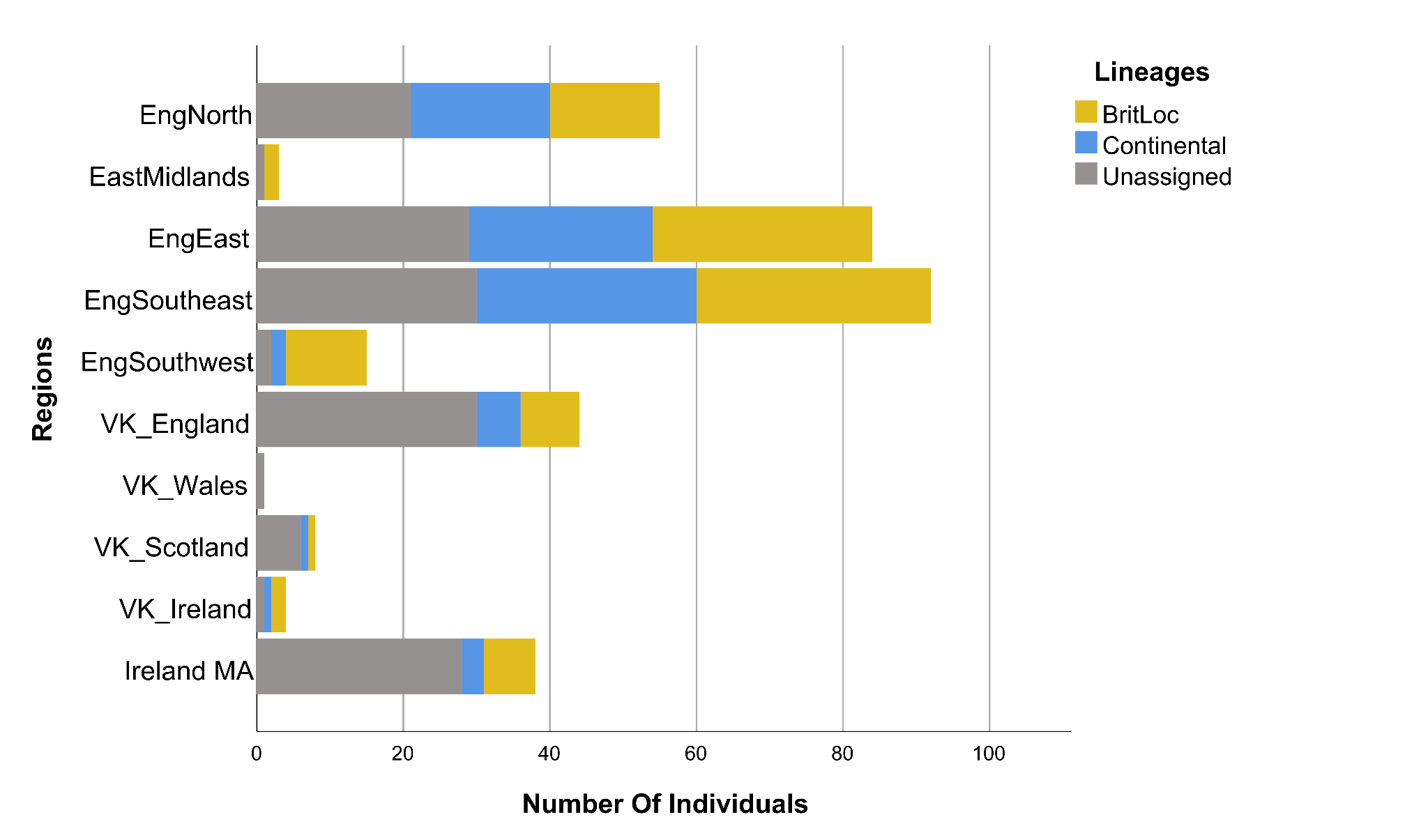


***Supp. Figure 2.5. Distribution of the individuals carrying “Local”, “Medieval”, and “Unassigned” mitochondrial lineages across the major geographic regions of the Eng_EMA, British and Irish Vikings, and Ireland_MA groups.***

To isolate the possible medieval signal further and investigate its sources, we excluded all lineages detected in pre-medieval Britain (BritLoc, *n* = 239) and focused our attention on the remaining 293 lineages shared or unique to the Eng_EMA and the medieval groups from the North Sea region and Scandinavian Peninsula (Supp. Figure 2.4). During this filtering we left the Irish medieval (Ire_MA), and British and Irish Viking (VK_Brit&Ire) groups unconstrained, under the assumption that, once all British pre-medieval lineages had been filtered out, any remaining shared lineages with the three target populations should be considered as deriving from one of the three.

Following the filtering, Eng_EMA included 119 mitochondrial lineages (Supp. Figure 2.6), of which 74 were unique to the group, 26 were uniquely shared between Eng_EMA and the continental North Sea region (NorthSea), eleven were uniquely shared with the Scandinavian Peninsula (Scand), and eight were shared across the three groups. 91 lineages were unique to the NorthSea group, 66 to the Scand group and 17 were shared between the two.


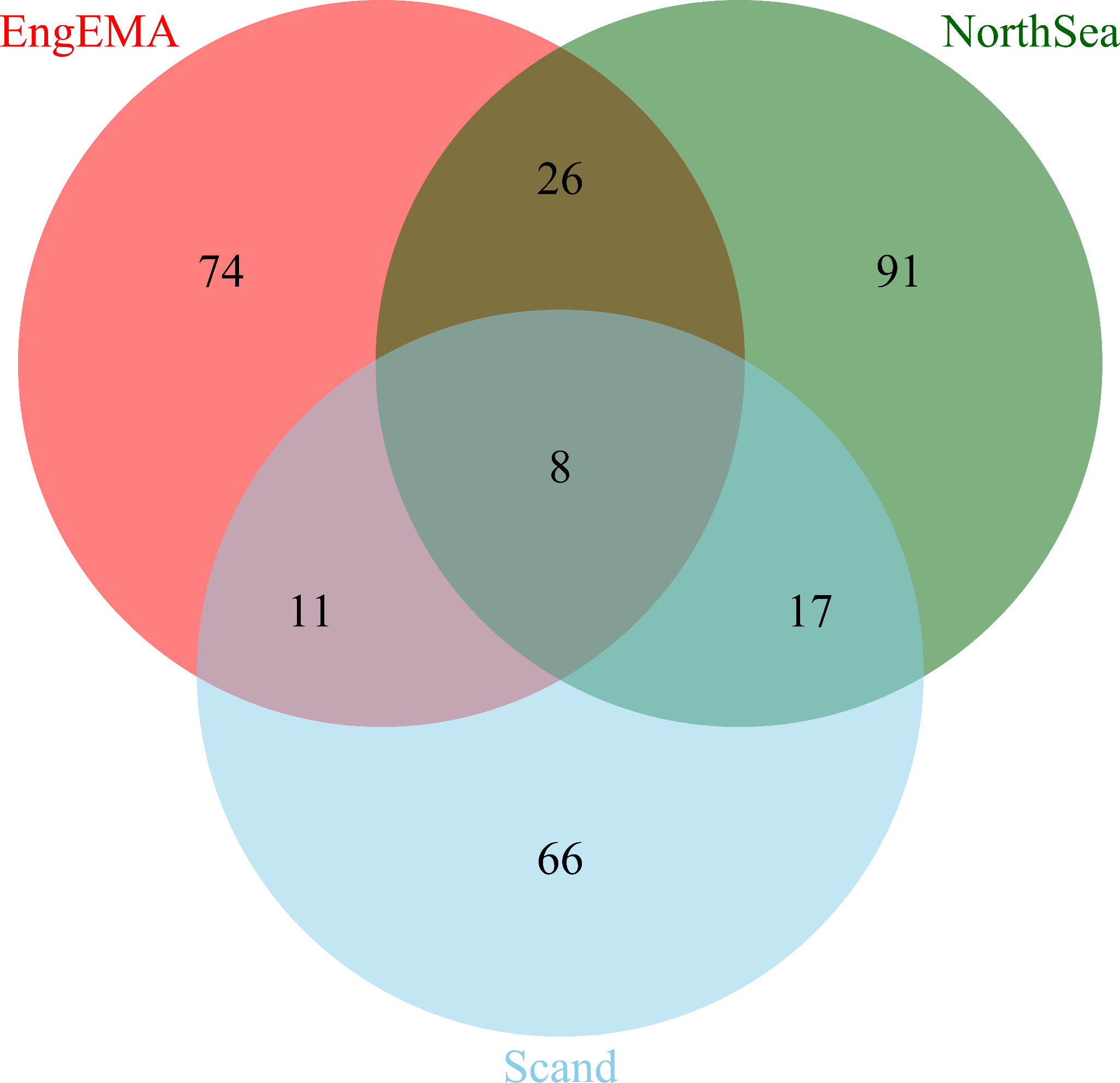


***Supplementary Figure 2.6. Mitochondrial lineage sharing between EngEMA and the two Continental Medieval sources: the NorthSea region and Scand (Scandinavian Peninsula).***

Of the 26 lineages exclusively shared between Eng_EMA and the NorthSea region (Supp. Table 2.3), we found 21 among the larger NorthSea_BA_IA group (*n* = 199); of these, 17 were uniquely shared, while four were also present in the smaller VK_NorthSea group (*n* = 83). The remaining five were exclusively shared between Eng_EMA and the VK_NorthSea group. Among the 26 lineages, we labelled five (H1b, U5a1a1+16362, U5a1a1d, V+@16298 and X2b4) as “uncertain” as they were either too nodal to be geographically restricted and/or were nested within clusters of lineages that had been detected in pre-medieval Britain. We also detected several NorthSea linked lineages among the Ire_MA group (two occurrences), and British and Irish Vikings (five occurrences from England, Scotland and Ireland).

*Supp. Table 2.3. Mitochondrial lineages exclusively shared between Eng_EMA and the continental North Sea region. Underlined lineages are classed as “uncertain” as they are either too nodal, or not supported by the sharing pattern of other sister lineages or subclades. Tabulated numbers represent the number of individuals carrying each lineage.*

| **Lineages** | **Eng_EMA (*n*=249)** | NorthSea_  BA_IA_EMA  (*n*=199) | VK_NorthSea (*n*=83) | VK_Scand (*n*=170) | Scand_IA_EMA  (*n*=14) | VK_Brit&Ire (*n*=44) | Ire_MA (*n*=38) |
| --- | --- | --- | --- | --- | --- | --- | --- |
| V+@16298 | **1** | 1 |  |  |  |  |  |
| HV6 | **4** |  | 1 |  |  | 1 |  |
| HV9a1a | **3** | 1 |  |  |  |  |  |
| H1b | **1** | 2 | 1 |  |  |  | 1 |
| H1c2 | **3** | 1 |  |  |  |  |  |
| H4a1a4b | **1** | 1 | 1 |  |  | 1 |  |
| H5a1c1a | **2** | 1 |  |  |  | 1 |  |
| H5b | **1** | 1 |  |  |  |  |  |
| H5b1 | **1** | 1 |  |  |  |  |  |
| H6a1b3 | **2** | 1 |  |  |  |  |  |
| H11a2a | **1** | 1 |  |  |  |  |  |
| H18b | **1** | 1 |  |  |  |  |  |
| J1c2c | **1** | 1 |  |  |  |  |  |
| J1c5a1 | **2** |  | 1 |  |  |  |  |
| J2a1a1a2 | **5** | 4 |  |  |  |  |  |
| T1a1c | **1** |  | 1 |  |  |  |  |
| U4b1b1 | **1** | 1 |  |  |  | 2 |  |
| U5a1a1+16362 | **1** |  | 1 |  |  |  |  |
| U5a1a1d | **1** |  | 1 |  |  |  |  |
| K1a1b2a1a | **1** | 2 |  |  |  |  |  |
| K1a4d | **1** | 2 |  |  |  | 1 |  |
| K1c2 | **2** | 1 | 1 |  |  |  | 1 |
| K1d | **1** | 1 |  |  |  |  |  |
| K2a6 | **2** | 2 | 1 |  |  |  |  |
| I1* | **1** | 1 |  |  |  |  |  |
| X2b4 | **1** | 2 |  |  |  |  |  |

We found NorthSea-specific associated lineages across all regions of England (Supp. Table 2.4). Similarly, when looking at the distribution of these lineages across the continental groups, we found these across all three countries: Germany, the Netherlands, and Denmark (including the “Viking” group from Denmark). Only three lineages appeared country specific; these are J2a1a1a2, found in samples from Oakington, Norton Bishops Mill, Dover Buckland and Polhill, and also at just one site (Groningen) in the northern Netherlands; K1a1b2a1a, found at both RAF Lakenheath in East Anglia, and at the 7^th^ century CE Alemannic burial site of Niederstotzingen, in southern Germany; and X2b4 found at West Heslerton, and again at Niederstotzingen.

*Supp. Table 2.4. Distribution of samples carrying mitochondrial lineages of putative continental medieval origin across the Eng_EMA sample. The “N.Sea” and “Scand” columns include counts for lineages that are found exclusively in each group, while the “N.Sea&Scand” column, includes counts of lineages that are shared by both.*

|  |  | **Shared** | | | | | |
| --- | --- | --- | --- | --- | --- | --- | --- |
| **England_EMA** | ***n*** | **N.Sea** | **%^a^** | **Scand** | **%^a^** | **N.Sea&Scand** | **%^a^** |
| **England** | **249** | **42** | **17** | **21** | **8** | **13** | **5** |
| **England North** | **55** | **9** | **16** | **4** | **7** | **6** | **11** |
| Clapdale, Fox Holes Cave | 1 |  |  |  |  |  |  |
| Hartlepool, Olive Street | 1 | 1 |  |  |  |  |  |
| Norton Bishops Mill | 5 | 1 |  |  |  | 1 |  |
| Norton East Mill | 5 | 1 |  |  |  |  |  |
| Ribblesdale, Selside Grike | 1 |  |  |  |  |  |  |
| West Heslerton | 42 | 6 | 14 | 4 | 10 | 5 | 12 |
| **East Midlands** | **3** |  |  |  |  |  |  |
| Lincoln, Lincoln Castle | 3 |  |  |  |  |  |  |
| **England East** | **88** | **13** | **15** | **9** | **10** | **3** | **3** |
| Ely | 6 | 1 |  |  |  |  |  |
| Hatherdene Close | 17 | 5 | 29 | 2 | 12 | 1 | 6 |
| Hinxton | 3 |  |  |  |  | 1 |  |
| Linton | 2 |  |  |  |  |  |  |
| Oakington | 26 | 4 | 15 | 4 | 15 |  |  |
| RAF Lakenheath | 16 | 2 | 13 |  |  |  |  |
| Sedgeford | 18 | 1 | 6 | 3 | 17 | 1 | 6 |
| **England Southeast** | **93** | **18** | **19** | **8** | **9** | **4** | **4** |
| Apple Down, West Chichester | 9 | 3 |  |  |  |  |  |
| Dover Buckland | 56 | 9 | 16 | 7 | 13 | 1 | 2 |
| Eastry Updown | 6 |  |  |  |  | 2 |  |
| Polhill | 9 | 4 |  | 1 |  |  |  |
| Rookery Hill, Bishopstone | 9 | 2 |  |  |  | 1 |  |
| Wolverton, Radcliffe School | 4 |  |  |  |  |  |  |
| **England Southwest** | **20** | **2** | **10** |  |  |  |  |
| Bude, Widemouth Bay | 1 |  |  |  |  |  |  |
| Newquay, Crantock | 2 |  |  |  |  |  |  |
| Worth Matravers | 17 | 2 | 12 |  |  |  |  |
| **Viking_Britain/Ireland** | **45** | **6** | **13** |  |  |  |  |
| **England** | **32** | **5** | **16** |  |  |  |  |
| **England Southeast** | **22** | **2** | **9** |  |  |  |  |
| Oxford, St John's College Oxford | 22 | 2 | 9 |  |  |  |  |
| **England Southwest** | **10** | **2** | **20** |  |  |  |  |
| Dorset, Ridgeway Hill Mass Grave | 10 | 2 | 20 |  |  |  |  |
| **Scotland** | **8** | **1** |  |  |  |  |  |
| **Highlands & Islands** | **8** | **1** |  |  |  |  |  |
| Orkney, Brough Road Birsay | 3 | 1 |  |  |  |  |  |
| Orkney, Buckquoy Birsay | 2 |  |  |  |  |  |  |
| Orkney, Newark Deerness | 3 |  |  |  |  |  |  |
| **Wales** | **1** |  |  |  |  |  |  |
| **Anglesey** | **1** |  |  |  |  |  |  |
| Glyn Llanbedrgoch | 1 |  |  |  |  |  |  |
| **Ireland** | **4** | **1** |  |  |  |  |  |
| **Connaught** | **1** |  |  |  |  |  |  |
| Eyrephort | 1 |  |  |  |  |  |  |
| **Leinster** | **3** | **1** |  |  |  |  |  |
| Finglas | 1 |  |  |  |  |  |  |
| Islandbridge | 1 | 1 |  |  |  |  |  |
| Ship Street Great | 1 |  |  |  |  |  |  |
| **Ireland_MA** | **38** | **2** | **5** | **1** | **3** |  |  |
| **Ireland** | **38** | **2** | **5** | **1** | **3** |  |  |
| **Connaught** | **38** | **2** | **5** | **1** | **3** |  |  |
| Kilteasheen, The Bishop’s Seat | 38 | 2 | 5 | 1 | 3 |  |  |
| ^a^ Percentages are displayed only when *n* ≥ 10 | | | | | | | |

Of the eleven lineages exclusively shared between Eng_EMA and the groups from the Scandinavian Peninsula (Supp. Table 2.5), we observed ten among the larger sample of the Vikings from Scandinavia (*n* = 170); only one, H1a3b, appeared as unique to the Scand_IA_EMA group (*n* = 14), matching one individual from Öland in Southeast Sweden. Among the eleven matches, we labelled four (H1b1, I4a1, K2b1a1 and U5a2b) as “uncertain,” as they were nested among lineages found in pre-medieval Britain. We also detected one occurrence among the Ire_MA group, but no matches among the British and Irish Vikings.

*Supp. Table 2.5. Mitochondrial lineages exclusively shared between Eng_EMA and the Scandinavian Peninsula. Underlined lineages are classed as “uncertain” as they are either too nodal, or not supported by the sharing pattern of other sister/sub-lineages. Tabulated numbers represent the number of individuals carrying each lineage.*

| **Lineages** | **Eng_EMA (*n*=249)** | NorthSea_  BA_IA_EMA  (*n*=199) | VK_NorthSea (*n*=83) | VK_Scand (*n*=170) | Scand_IA_EMA (*n*=14) | VK_Brit&Ire (*n*=44) | Ire_MA (*n*=38) |
| --- | --- | --- | --- | --- | --- | --- | --- |
| H1a3b | **1** |  |  |  | 1 |  |  |
| H1b1 | **2** |  |  | 1 |  |  |  |
| H1b5 | **1** |  |  | 1 |  |  |  |
| H1g1 | **4** |  |  | 1 |  |  |  |
| H10e | **1** |  |  | 1 |  |  |  |
| J1c3f | **5** |  |  | 1 |  |  | 1 |
| T2b11 | **1** |  |  | 1 |  |  |  |
| T2b24 | **1** |  |  | 1 |  |  |  |
| U5a2b | **2** |  |  | 1 |  |  |  |
| K2b1a1 | **1** |  |  | 2 |  |  |  |
| I4a1 | **1** |  |  | 1 |  |  |  |

The lineages of putative Scandinavian origin are detected across all sampled English regions except for England Southwest (mostly represented by the post-Roman Worth Matravers), where there are no matches (Supp. Table 2.4). Frequencies are usually lower than those characterising lineages of NorthSea origin, with some exceptions in England East: Sedgeford (*n =* 18) with 17% Scand *vs* 6 % NSea, and Oakington (*n =* 26) with 15% Scand *vs* 15 % NSea. Matches at the source are found in Sweden Southeast (seven occurrences), incidentally the largest sample from the Scandinavian dataset (*n =* 103), at sites on the two major islands on the Baltic Sea: Gotland and Öland; in Sweden South at Varnhem (two occurrences); and in Norway, at Nordland in the north, and Telemark in the south.

We found eight lineages shared among Eng_EMA and both the NorthSea and the Scandinavian groups (Supp. Table 2.6), of these, we labelled three (H1a*, H7*, and T2*) as “uncertain” as they were all nodal haplogroups, the remaining five seemed well supported as they nested within clusters that had no representatives in pre-medieval Britain.

*Supp. Table 2.6. Mitochondrial lineages shared between Eng_EMA and the Continental Medieval groups (North Sea region & Scandinavian Peninsula). Underlined lineages are classed as “uncertain” as they are either too nodal, or not supported by the sharing pattern of other sister/sub-lineages. Tabulated numbers represent the number of individuals carrying each lineage.*

| **Lineages** | **Eng_EMA (*n*=249)** | NorthSea_  BA_IA_EMA (*n*=199) | VK_NorthSea (*n*=83) | VK_Scand (*n*=170) | Scand_IA_EMA (*n*=14) | VK_Brit&Ire  (*n*=44) | Ire_MA (*n*=38) |
| --- | --- | --- | --- | --- | --- | --- | --- |
| HV9 | **1** | 1 |  | 1 |  |  |  |
| H1a* | **1** | 1 | 1 | 2 |  |  |  |
| H7* | **1** | 1 |  | 1 |  |  |  |
| T2* | **2** | 2 | 1 | 1 |  |  |  |
| T2f1a1 | **3** | 1 |  | 1 |  |  |  |
| U5b1c2b | **2** | 2 |  | 1 |  |  |  |
| K1a4a1a2b | **2** | 1 |  | 2 |  |  |  |
| I1a1 | **1** |  | 1 | 1 | 1 |  |  |

As before, these lineages were present in all sampled regions in England EMA, with the exception of England Southwest (Supp Table 2.4). Five out of 13 occurrences were found at a single site, West Heslerton, in the North of England. While the matches at the sources were found across all sampled countries. These observations remain valid even when the “uncertain” lineages are excluded.

We detected 17 lineages shared only among the Continental Medieval groups (Supp. Table 2.7) and we labelled six of these (HV+16311, H13a1a1a, K1a*, T2b+152, U4a2a and W1*) as “uncertain.” We found a single occurrence of one of these “uncertain” lineages, T2b+152, in the Ire_MA group.

*Supp. Table 2.7. Mitochondrial lineages shared between the two Continental Medieval groups (North Sea region & Scandinavian Peninsula). Lineages in italics are labelled as “uncertain” as they are either too nodal, or not supported by the sharing pattern of other sister lineages. The numbers represent the number of individuals carrying each lineage.*

| **Lineages** | **Eng_EMA (*n*=249)** | NorthSea_BA_IA_EMA (*n*=199) | VK_NorthSea (*n*=83) | VK_Scand (*n*=170) | Scand_IA_EMA (*n*=14) | VK_Brit&Ire (*n*=44) | Ire_MA (*n*=38) |
| --- | --- | --- | --- | --- | --- | --- | --- |
| *HV+16311* |  | 2 |  | 1 |  |  |  |
| H2a2b |  | 1 |  | 2 |  |  |  |
| H3h |  | 1 |  | 1 |  |  |  |
| H8c |  | 1 |  | 1 |  |  |  |
| *H13a1a1a* |  |  | 1 | 1 |  |  |  |
| H27 |  | 1 |  | 1 |  |  |  |
| H56 |  | 1 |  | 1 |  |  |  |
| J1c2c1 |  |  | 1 |  | 1 |  |  |
| J1c2k |  |  | 1 | 1 |  |  |  |
| J2b1a3 |  | 2 |  | 1 |  |  |  |
| T1a1b |  | 1 | 1 | 1 |  |  |  |
| *T2b+152* |  |  | 1 | 1 |  |  | 1 |
| *U4a2a* |  | 1 |  | 1 |  |  |  |
| U5b2a1a2 |  | 1 |  | 1 |  |  |  |
| *K1a** |  | 3 |  | 1 |  |  |  |
| *W1** |  | 1 |  |  | 1 |  |  |
| W3a1 |  |  | 1 | 1 |  |  |  |

In conclusion, our analysis revealed that, albeit with some local exceptions, early medieval English individuals (Eng_EMA) carried similar frequencies of mitochondrial lineages of putative local (BritLoc) and continental medieval origin. We also found that Eng_EMA shared more lineages with the continental North Sea region than with the Scandinavian Peninsula, which is not surprising given that most of the Scandinavian sample is represented by individuals from Viking-era burials, and thus might carry different lineages only partially representative of the earlier Scandinavian mitochondrial gene pool.

To assess whether this difference is statistically significant – or, more precisely, whether shared lineages are more strongly associated, and hence occur more frequently, with the North Sea region than with the Scandinavian Peninsula – we performed a Pearson’s chi-squared (*X^2^)* test of independence under the null hypothesis that there is no association between shared/non-shared lineages and the two sources North Sea/Scandinavia using an alpha = 0.05.

To perform the test, we counted only the Eng_EMA lineages that are uniquely shared with NorthSea (*n =* 26) and Scand (*n =* 11), as a proportion of the total number of lineages available between each pair (*n* = 194 and *n* = 119, respectively), and excluded from the count the eight lineages that were detected across the three groups (Supp. Figure 2.6). We found that there was a significant association between the two variables, *X^2^* (1, *n* = 413) = 4.851, *p* = 0.028. We repeated the same test using a more conservative approach and excluded from the count the shared lineages classed “uncertain” (with *n* = 7 and 21 uniquely shared with the two groups, out of *n* = 190 and 114, respectively). The result was again significant, *X^2^* (1, *n* = 404) = 5.861, *p* = 0.015. Therefore, we can conclude that the observed difference in number of shared lineages between Eng_EMA and the two continental sources is significant, and that the shared lineages are more likely to be associated with the North Sea group than with Scandinavia.

##

## Y-chromosome

The Y-chromosome haplogroups observed in early medieval males from England show a typical north-west European distribution (Supplementary figure 2.7a, Supp. Table 5). We detect a prevalence of R1b-M343 lineages (57%), which is the most common haplogroup in western Europe, with a peak in the Iberian Peninsula and in the British-Irish Isles and a west-east gradient in central Europe[^151^](https://paperpile.com/c/hqnLqQ/ewRM1). In our medieval Irish sample from Kilteasheen, 96% of the typed haplogroups belong to R1b-M343. Sub-haplogroup R1b-P312 has been, since the Bell Beaker period, the most frequent haplogroup in Ireland and western Britain[^87,130^](https://paperpile.com/c/hqnLqQ/r5iW6+5danp) (80% of all haplogroups during the Bronze and Iron Age), as well as in continental Europe southwest of the Rhine river[^151^](https://paperpile.com/c/hqnLqQ/ewRM1). In particular, its subclade R1b-L21 is dominant in northern and western Britain as well as in Ireland (at 94%, it is almost fixed in the west of Ireland[^152,153^](https://paperpile.com/c/hqnLqQ/71w4f+oRaH6)) and is found in at least nine of our medieval English samples (45% of all R1b-P312 haplogroups), 16 of our medieval Irish individuals (70% of all R1b-P312 haplogroups), and the majority of the previously published Bronze/Iron Age British and Irish males (76% of all R1b-P312 haplogroups)(Supp. Fig. 2.7b). While in Bronze Age and Iron Age Britain R1b-P312 haplogroups are dominant, during the Early Middle Ages, we measure a substantial increase of haplogroup R1b-U106 (66% of all haplogroups typed past R1b-L151, 53% of all R1b* haplogroups, 30% of all classified haplogroups) (Supp. Fig. 2.7b), especially subclade R1b-L48 which peaks in Friesland and North Holland[^154^](https://paperpile.com/c/hqnLqQ/Ok2Cw) (39% of all R1b-U106 haplogroups). The distribution of the R1b-U106 lineage in present-day Europe is centred along the Rhine River. It is the dominant R1b-M343 sub-haplogroup in Germany, the Netherlands, Denmark, Sweden, and Norway[^151,153,155^](https://paperpile.com/c/hqnLqQ/ewRM1+oRaH6+PsVeU). In the Iron Age and medieval samples from the Netherlands, northern Germany (Lower Saxony, Schleswig-Holstein, and Mecklenburg-Vorpommern), and Denmark 25% of all classified haplogroups belong to the R1b-U106 lineages (50% of all R1b-M343 haplogroups and 65% of all R1b-M343 haplogroups typed past R1b-L151). It peaks in Groningen (75% of R1b-L151, 50% of all haplogroups) and Lower Saxony (69% of R1b-L151, 29% of all haplogroups). While R1b-U106 is completely absent during Bronze Age times in Britain and Ireland[^130^](https://paperpile.com/c/hqnLqQ/5danp), it is first observed in England in an Early Iron Age individual from Teversham[^81^](https://paperpile.com/c/hqnLqQ/3Th9), Cambridgeshire, as well in two Roman period males from York[^121^](https://paperpile.com/c/hqnLqQ/jnKBm). During the Early Middle Ages it is more frequent than the R1b-P312 lineages (15% of all haplogroups, 26% of all R1b-M343 haplogroups, and 32% of R1b-L151) in England.

Almost as large a percentage of male lineages belongs to haplogroup I-M170 (39%), both in the I2-M438 (23% of I-M170 haplogroups) and, more abundantly, in I1-M253 sub-haplogroups (77% of I-M170 haplogroups), which were previously associated with migrations to Britain and Ireland during the Early Middle Ages and the Viking period, as suggested by the decreasing east-to-west frequency cline of I-M170 lineages in present-day England[^156,157^](https://paperpile.com/c/hqnLqQ/9PRMH+wOVd7) and their absence during Bronze and Iron Age times in Britain and Ireland (only one instance from Iron Age England is known so far[^81^](https://paperpile.com/c/hqnLqQ/3Th9)). I1-M253 sub-haplogroups are only found in ancient[^120^](https://paperpile.com/c/hqnLqQ/spmY) and modern northern Europe in high frequencies, especially in Scandinavia (reaching 52% in Central Sweden)[^158,159^](https://paperpile.com/c/hqnLqQ/oGgkh+FqI9e). In our continental sample subclade I1-M253 peaks in Schleswig, Häven, and Drantum, where it represents 31%, 38%, and 60% of all haplogroups respectively, as well as generally in Iron Age and Viking Age Denmark (42%).

In our ancient sample (and in the present-day), I2-M438 lineages are found only at low frequencies (7% of all haplogroups in the North Sea sample belong to this lineage). We find them in Anderten, Groningen, Alt-Inden and Iron Age and Viking Age Denmark, exhibiting the highest frequency in Dunum (18% of all Y-chromosome lineages). I2-M438 haplogroups, especially the 12a1b-M436 lineages that comprise the majority of our early medieval English I2-M438 haplogroups, were found across Neolithic Europe (including Germany, France, England, Wales, Scotland, and Ireland[^85,88,130^](https://paperpile.com/c/hqnLqQ/5danp+VTvWq+VGqUa). However, their frequencies in Britain and Ireland dropped drastically at the beginning of the Bronze Age due to the introduction of R1b-P312 lineages, so that they account for less than ~4% during the Bronze and Iron Age in Britain and Ireland)[^87,130^](https://paperpile.com/c/hqnLqQ/5danp+r5iW6). Nevertheless, I2a1b1a2b-Z161 in particular is distributed in present-day Denmark, Germany, the Netherlands, and England[^150^](https://paperpile.com/c/hqnLqQ/gzbxt), with ancient individuals belonging to this haplogroup in Lakenheath, Sedgeford, West Heslerton, Dover Buckland, as well as Schleswig, Anderten, Alt-Inden, and Groningen on the continent. I2a1a-P37.2 lineages, which were common in Neolithic Atlantic Europe as well[^160^](https://paperpile.com/c/hqnLqQ/DlY7), are also found in Lakenheath, West Heslerton, Groningen, and are reported in Late Iron Age England[^81^](https://paperpile.com/c/hqnLqQ/3Th9).

Rarer lineages in early medieval England include R1a-M420 sub-haplogroups (~2% of all haplogroups), which show higher occurrence in eastern Europe but are also present in central and northern Europeans such as Germans and Norwegians[^148,161^](https://paperpile.com/c/hqnLqQ/6hnh9+MNI8E). Consistently, we measure a relatively similar frequency of R1a-M420 lineages in our North Sea continental samples (~7% of all haplogroups) and a substantially higher frequency in Iron Age and medieval males from the Scandinavian Peninsula (~23%). The major haplogroup R1a1-M459 especially was previously identified as a marker for “Viking” migrations into England since it shows the highest frequency within northwestern Europe in Norway and Sweden[^148,162^](https://paperpile.com/c/hqnLqQ/f2Rv7+6hnh9), as well as high percentages in Viking Age Scandinavians[^120^](https://paperpile.com/c/hqnLqQ/spmY). Our results indicate that at least certain sub-haplogroups of R1a1-M459 had already entered Britain before the establishment of the Danelaw. Four sub-haplotypes found in Copenhagen, Drantum, Polhill, and Sedgeford may belong to haplogroup R1a1a1b1a3-Z289, which is nearly exclusively confined to Scandinavia in the present-day and peaks in Norway (up to 20%)[^161^](https://paperpile.com/c/hqnLqQ/MNI8E), while two individuals found in Schortens could potentially carry lineages belonging to R1a1a1a-CTS4385, which is the most frequent R1a-M420 haplogroup in Denmark (43%) and Frisia (57%) and also represents a substantial fraction of the R1a haplotypes in Norway (8%), Sweden (20%) and England (20%)[^148^](https://paperpile.com/c/hqnLqQ/6hnh9). Furthermore, one haplotype in West Heslerton may belong to R1a1a1b1a2b-CTS1211, which shows highest frequencies in northeastern and eastern Europe (reaching 33% in Russia and 26% in Poland[^148,161^](https://paperpile.com/c/hqnLqQ/MNI8E+6hnh9)).

Three much rarer haplogroups, E1b1-P2, J2-L228, and T1a-PF5651 (all at ~1%), show a Mediterranean distribution with a prevalence in southern Europe, in particular across the Balkan, Iberian, and Italian Peninsulas[^146,163,164^](https://paperpile.com/c/hqnLqQ/jWTHJ+eVyLN+mViEN) but are also present in low frequencies in present-day[^92^](https://paperpile.com/c/hqnLqQ/9O9CJ) and medieval continental northern Europe and Viking Age Scandinavia[^120^](https://paperpile.com/c/hqnLqQ/spmY). Interestingly, rather than in one of the more cosmopolitan trading centres like Groningen, Schleswig, or Copenhagen, we find J2-L228 in Anderten, where the haplogroup peaks due to the presence of a paternally–related kin group of four males. Within England, haplogroup J2-L228 was first observed in a Roman individual from York who originated from the Middle East, as suggested by genomic and isotopic evidence[^121^](https://paperpile.com/c/hqnLqQ/jnKBm).


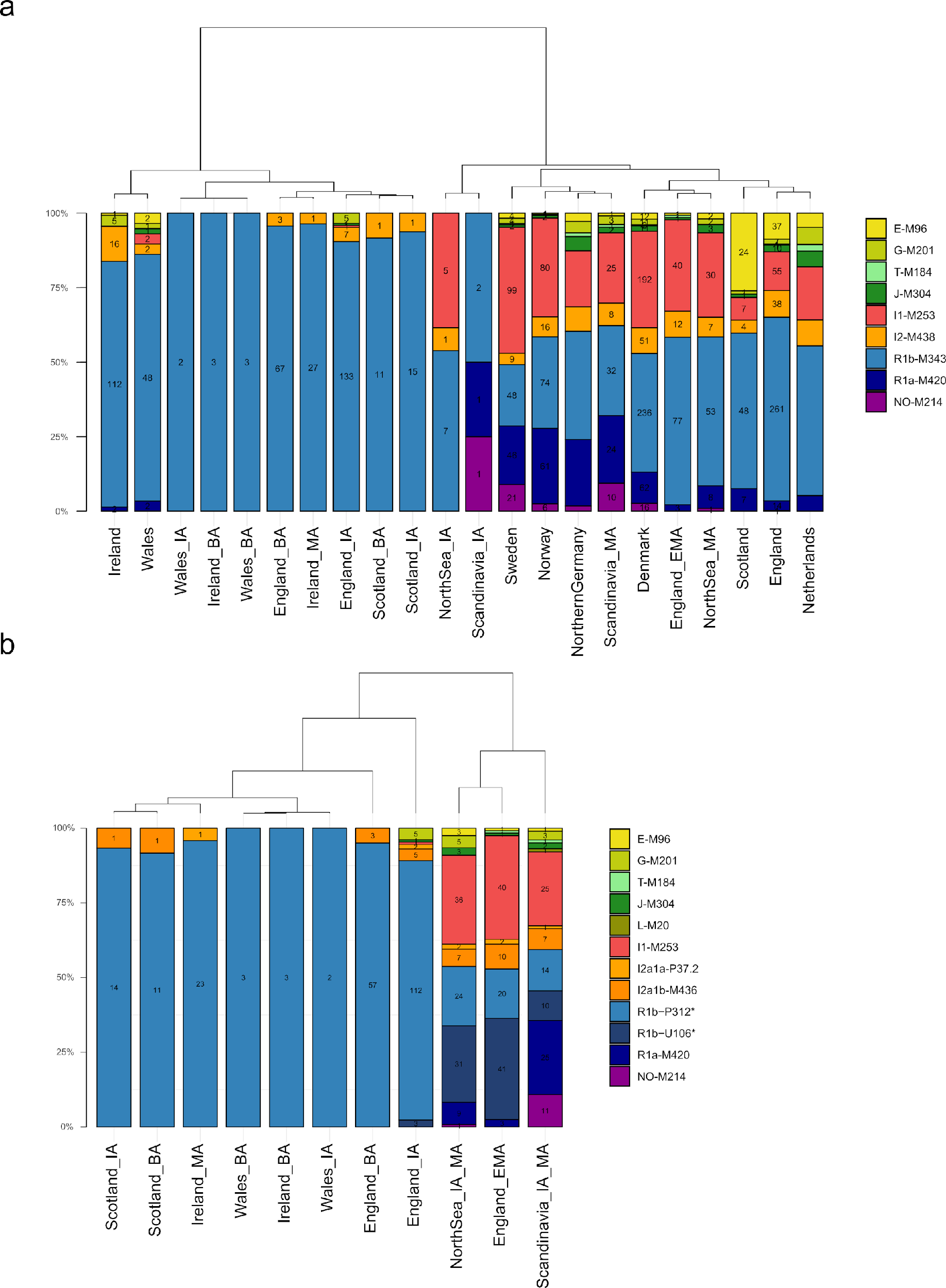


***Supplementary Figure 2.7, Y-chromosome diversity.*** *Relative and absolute frequencies of Y-chromosome haplogroups across ancient samples from this and previous studies for which Y-chromosome haplogroups could be determined. Hierarchical cluster analysis applying Ward’s minimum variance method is shown as a dendrogram. a) Comparing ancient and present-day populations. Present-day absolute frequencies for Britain and Ireland as well as Scandinavia are taken from Ebenesersdóttir et al. 2018*[*^92^*](https://paperpile.com/c/hqnLqQ/9O9CJ)*. For northern Germany and the Netherlands, only relative frequencies were available. Individuals for which coverage was too low to determine haplotypes for the major haplogroups depicted here, were excluded. b) Comparing ancient populations with a focus on R1b-M343 and I-M170 sub-haplogroups. Individuals for which coverage was too low to determine haplotypes downstream of R1b-P311 and I-M170 were excluded.*

Using Fisher’s exact test, we infer that *England_EMA* differs significantly in their Y-chromosome frequencies both from *England_BA* and *England_IA* (for both *p* < 9.999e-05). Contrarily, for *England_EMA* and *NorthSea_IA_EMA* we do not detect such a differentiation (*p* = 0.1627). This signal is even clearer when we exclude R1b-M343 lineages that could not be classified downstream of R1b-L151 due to low coverage and therefore insufficient genotyping (*p* = 0.2821). The comparison of early medieval England, considered as a whole, with modern European groups shows a marked resemblance with the present-day Danish population because of a similar ratio between R1b-U106/R1a/I haplogroups[^92,165^](https://paperpile.com/c/hqnLqQ/9O9CJ+wv8hC), but with some lineages present in the ancient and modern western British and Irish, indicating a major continental northern European component, and a noticeable contribution from insular British populations (Supp. Fig. 2.7).

To estimate those contributions based on the frequencies of local and immigrant Y markers, we counted the early medieval English individuals with Y-chromosomes belonging to the sub-haplogroups R1b-U106, R1b-S1194 (found only once in the entire dataset), R1a-M420, I1-M253, as well as E1-CTS955, J2-L228, and T1-PF5636, which are all present in early medieval samples from England and continental northern Europe but rare or absent in Bronze and Iron Age English individuals[^121,130,137^](https://paperpile.com/c/hqnLqQ/5danp+I6Awn+jnKBm). The early medieval continental northern European individuals also carried R1b-P312 lineages (which we defined here as local due to their continuous presence since pre-EMA times), so the proportion of immigrant haplogroups represents the minimum paternal continental northern European contribution. Lineages that could not be assigned to any of the aforementioned sub-haplogroups due to low coverage were excluded as well as haplogroup I2-M438, which occurred in roughly similar frequencies in pre-EMA Britain and at the continent (5% in Bronze and Iron Age Britain & Ireland, 7% in the Iron Age and medieval North Sea sample). We computed binomial confidence intervals for the proportion of haplogroups associated with each ancestry type using the Agresti–Coull[^166,167^](https://paperpile.com/c/hqnLqQ/9rSCq+zzrMr) method implemented in the binom package in R. We infer 73% (95%CI: 65% - 81%) CNE-related ancestry on the Y-chromosome.

We further tested whether, in ancient post-Neolithic English males, continental Y-chromosome haplogroups were also correlated with immigrant CNE ancestry on the autosomes (estimated as in Fig. 3 & Supplementary Note 3). The comparison of autosomal CNE ancestry showed that ancient post-Neolithic English individuals with immigrant Y-chromosome haplogroups (R1b-U106, R-S1194, R1a-M420, I1-M253, E1-CTS955, J2-L228, T1-PF5636) carried significantly more CNE ancestry on the autosomes (Wilcoxon rank sum test; *W* = 16784*, p* < 2.2e-16) than those with local haplogroups. Average autosomal CNE ancestry differed by 81% between the two paternal ancestries (95% CI: 74% - 87%). This effect was also, though less pronounced, significant when only considering early medieval English individuals (Wilcoxon rank sum test; *W* = 1279*, p* = 7.744e-05). In the Early Middle Ages, average autosomal CNE ancestry differed by 33% between the two paternal ancestries (95% CI: 13% - 54%) (Supp. Fig. 2.8).


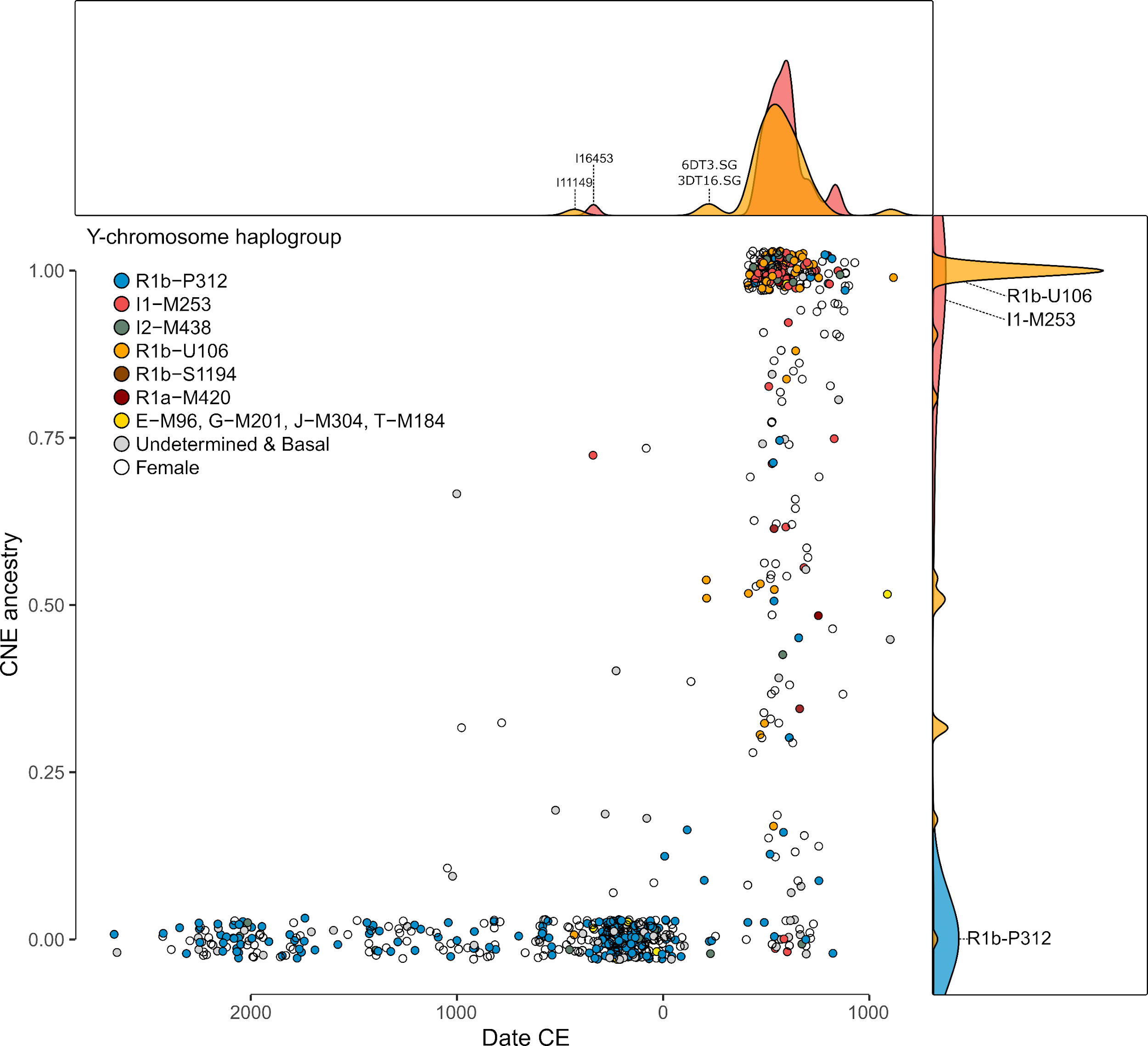


***Supplementary Figure 2.8, CNE ancestry on the autosome and the Y-chromosome.*** *Relative proportion of the CNE-related ancestry component for each ancient post-Neolithic English individual, calculated with supervised ADMIXTURE****.*** *White dots represent female individuals and filled dots male individuals, coloured according to their Y-chromosome haplogroup.*

## Sex bias

### Descriptive statistics

We then proceeded to quantify a potential sex bias within our early medieval samples from England. We first highlight that there is no significant difference in CNE ancestry between males and females (Wilcoxon rank sum test; *W* = 9608, *n_1_* = 142, *n_2_* = 143, *p* = 0.3852). Early medieval English males carry only 0.04% more autosomal CNE ancestry than females on average (95% CI: -8.2% - 8.1%). We further observe no significant difference in the distribution of admixture across sexes (Two-sample Kolmogorov-Smirnov test; *D* = 0.10233, *p* = 0.446) (Supp. Fig. 2.9). Neither females nor males appear more often admixed (5% < CNE < 95%) or unadmixed (CNE < 5% or CNE > 95%) in absolute counts (Pearson's Chi^2^ with simulated *p*-value (based on 10,000 replicates) ; *Chi^2^* = 3.9948, *d.f.* = NA, *p* = 0.0561). We also computed the statistic *Q*, which is an estimator of the ratio of effective population size of the X-chromosome with the ratio of effective population size of the autosomes based on the *F*_ST_ measure of genetic differentiation[^168^](https://paperpile.com/c/hqnLqQ/AnR16). Doing so, we followed the method of Keinan et al. and Waldman et al. and measured relative genetic drift between the X-chromosome and the autosomes, $Q=\frac{ln(1-2{FST}_{A})}{ln(1-2{FST}_{X})}$, where *FST_A_* and *FST_X_* are autosomal and X-chromosomal *F*_ST_ values[^169,170^](https://paperpile.com/c/hqnLqQ/m3c0y+0uPIW). Under a simple demographic model with equal male and female effective sizes, *Q* is expected to be 3/4, because there are three X-chromosomes for every four autosomes in the population. Deviations from 3/4 may therefore show sex-biased effective population sizes, which indicate different population histories for males and females[^168^](https://paperpile.com/c/hqnLqQ/AnR16). Comparing the early medieval Lower Saxony (the best fitting source of CNE ancestry in medieval England according to Section 3) and early medieval English populations, the ratio of X- and autosomal differentiation is consistent with what is expected for a non–sex-biased process (*Q* = 0.816 ± 0.232) (Supp. Table 2.8). Similarly, there is low relative differentiation on the X-chromosome between the Iron Age and early medieval English populations (*Q* = 0.682 ± 0.484) (Supp. Table 2.9), indicating no strong male or female bias during the immigration of people from continental Europe (*p*-value for difference between observed and expected = 0.777 and 0.888). However, as evidenced by the large error bars, those observations represent only weak evidence and cannot securely identify potential sex bias.


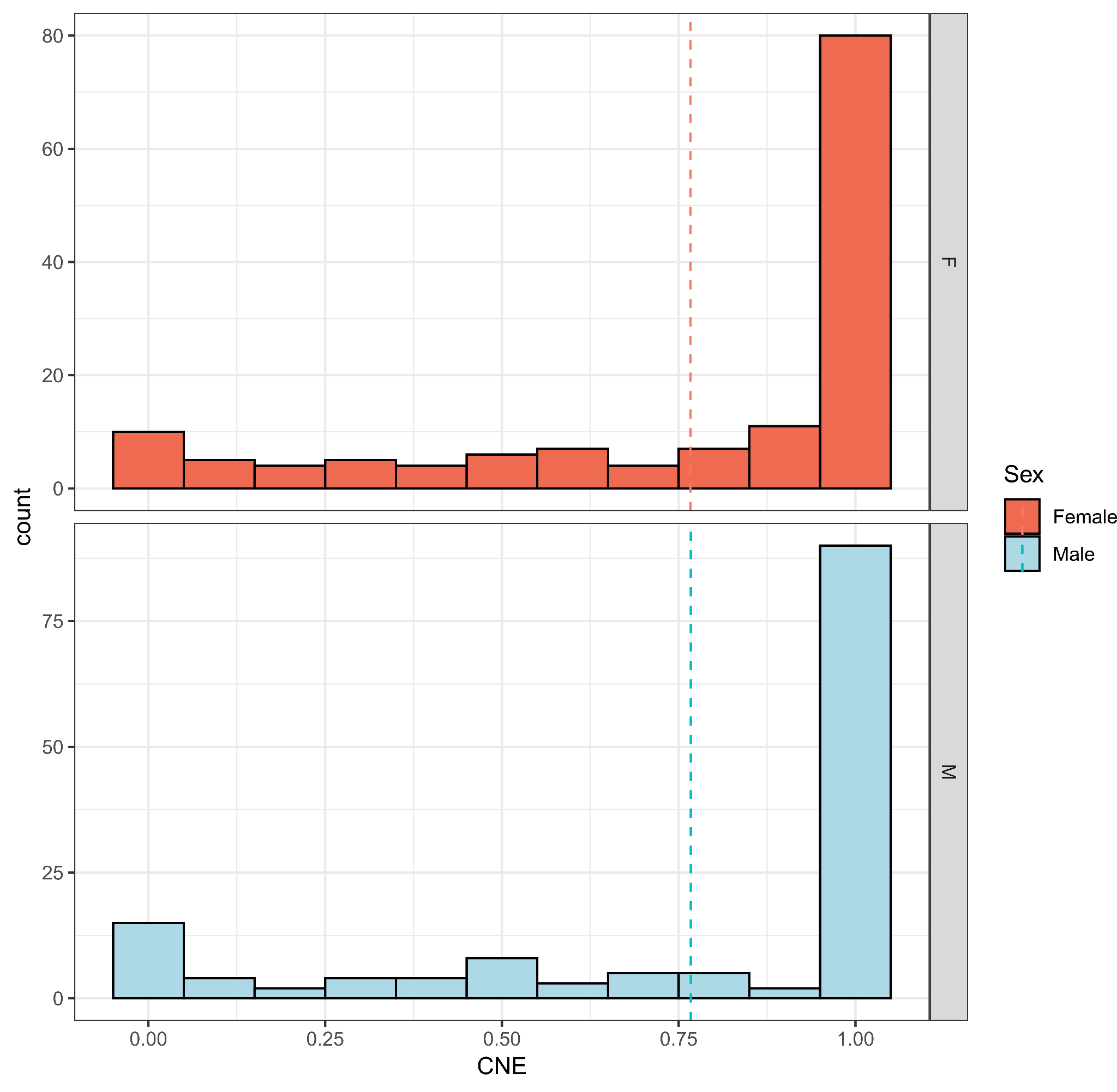


***Supplementary Figure 2.9, CNE ancestry in males and females.*** *The distribution of the relative proportion of the CNE-related ancestry across the early medieval English male and female population, calculated with Supervised ADMIXTURE. The respective mean CNE ancestry is indicated as dotted line.*

### ADMIXTURE analysis of the X-chromosome

A different approach relies on the comparison of admixture estimates on the autosomes and the X-chromosome. This is because males always inherit a maternal X-chromosome, so differences in these proportions imply sex-biased mixture. Since most of our modern reference data, especially from the continent, is not sufficiently genotyped on the X-chromosome, it cannot be used in the *qpAdm*[*^171^*](https://paperpile.com/c/hqnLqQ/eK1xv)-based approach described in Mathieson et al.[^124^](https://paperpile.com/c/hqnLqQ/2oozR) or in a direct supervised ADMIXTURE[^172^](https://paperpile.com/c/hqnLqQ/TNMqU) analysis as in Fig. 3. We therefore designed a supervised ADMIXTURE[^172^](https://paperpile.com/c/hqnLqQ/TNMqU) setup similar as in Fig. 3 that employs data sufficiently genotyped on the X-chromosome[^168,173^](https://paperpile.com/c/hqnLqQ/AnR16+VgyN1). To approximate the ancient admixture sources, we grouped ancient post-Bronze Age British and Irish together. To increase sample size we further included the haploid genomes of 100 modern Welsh and labelled this metapopulation as “ancient and modern western British and Irish” (mixed WBI, *n* = 600). Similarly, we proceeded to group medieval Dutch, Danish, and northern Germans genomes together as “ancient continental northern European” (ancient CNE, *n* = 124). We then used ADMIXTURE in supervised mode[^168,172,173^](https://paperpile.com/c/hqnLqQ/TNMqU+AnR16+VgyN1), where we estimated autosomal admixture proportions for a subset of 195 early medieval genomes from England that had coverage on the X-chromosome using those two reference populations with *K* = 2. This analysis was run on 905,679 SNPs across chromosomes 1 to 22. Subsequently, we compared the ancient CNE (aCNE) ancestry estimates on the autosomes to the estimates of “modern CNE” ancestry obtained using exclusively present-day reference populations (WBI, *n* = 667 & CNE, *n* = 407) on the same subset of 195 individuals and 431,824 autosomal SNPs. Although both approaches utilize different reference data, the autosomal estimates for each individual are highly correlated (*r* = 0.87, *t* = 24.6, *d.f.* = 193, *p* < 2.2e-16) with the mean CNE ancestry estimates obtained using modern and ancient references not being substantially different (76 ± 2.1% vs. 74.8 ± 2.7%) (Supp. Fig. 2.10). We therefore proceeded to model our early medieval subset exclusively on the X-chromosome (49,459 SNPs). We calculated a mean of 72.5 ± 3.2% ancient CNE ancestry on the X-chromosome, which is not significantly different to the estimates obtained on the autosomes using either modern sources (Wilcoxon signed rank test; *V* = 1815, *n* = 195, *p* = 0.3506) or ancient sources (Wilcoxon signed rank test; *V* = 2008, *n* = 195, *p* = 0.3136) or to the estimate based on the Y-chromosome haplogroups of this subset (73 ± 2.4%) (*p* = 0.835) of the investigated subsample (Supp. Fig. 2.11). Using the formula described in Mathieson et al.[^124^](https://paperpile.com/c/hqnLqQ/2oozR), $Z=\frac{P_{A}-P_{X}}{\sqrt{{\sigma_{A}}^{2}+{\sigma_{X}}^{2}}}$ in which *P_A_* and *P_X_* are the admixture proportions on the autosomes and the X-chromosome, and *σ_A_* and *σ_X_* are the corresponding standard errors[^124^](https://paperpile.com/c/hqnLqQ/2oozR), we calculated a sex-bias *Z* score of 0.54, which suggests no bias towards male CNE ancestry in early medieval samples from England (two-sided *p* = 0.586). However, we note that there may be regional differences between the sites.


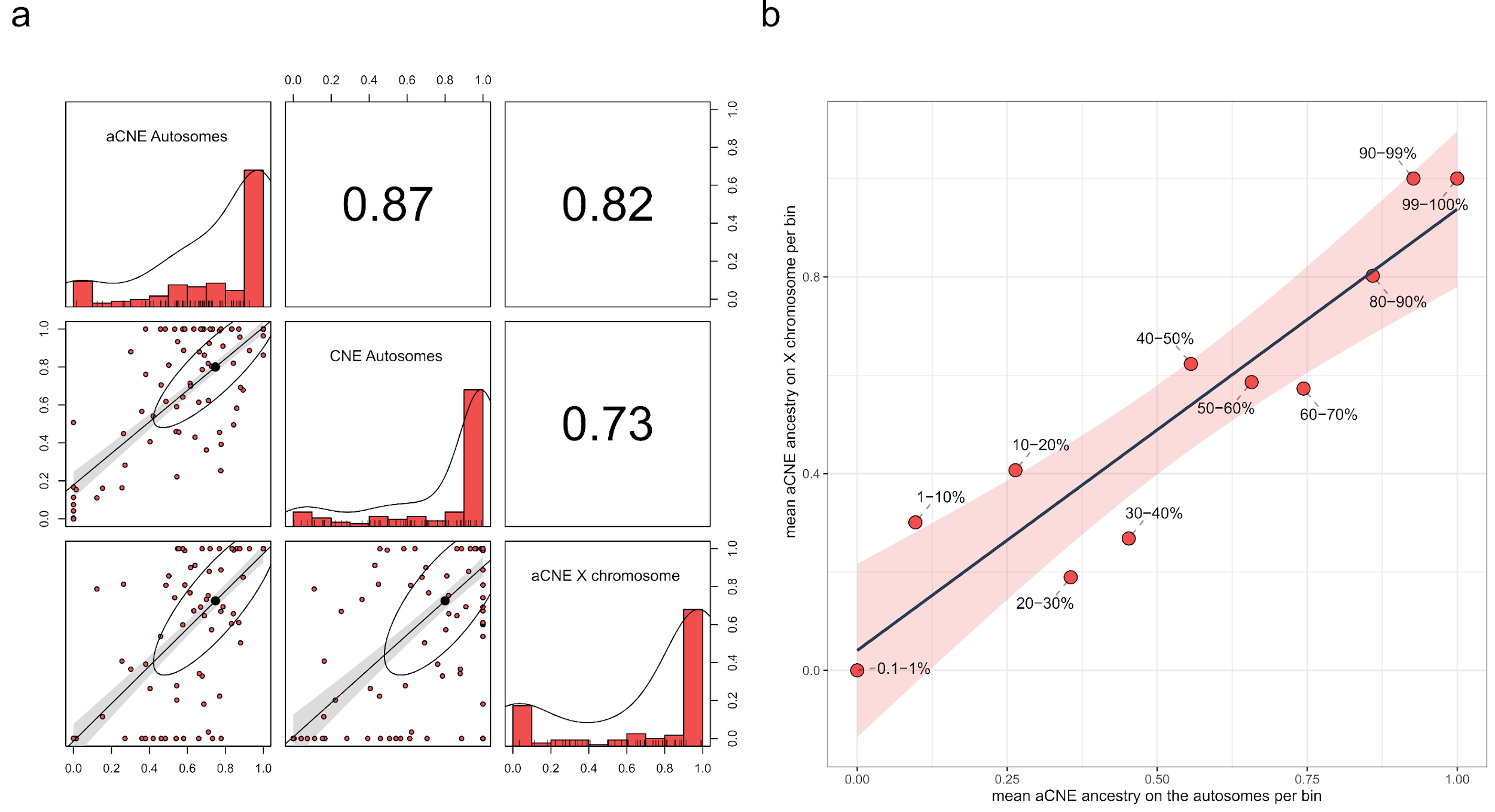


***Supplementary Figure 2.10, Validation of CNE estimates on the autosomes and X-chromosome using ADMIXTURE.*** *Correlogram illustrating pairwise correlations between different estimates of CNE ancestry on the autosomes and X-chromosome. Pearson’s r values for each pairwise correlation test are indicated in the upper triangle of the matrix. Error bands represent ± 2 standard errors. b) Linear regression of mean aCNE ancestry on the X-chromosome as function of mean aCNE ancestry on the autosomes. Samples were grouped in 10 bins of autosomal aCNE ancestry as indicated in the plot. The error band represents ± 2 standard errors.*

*
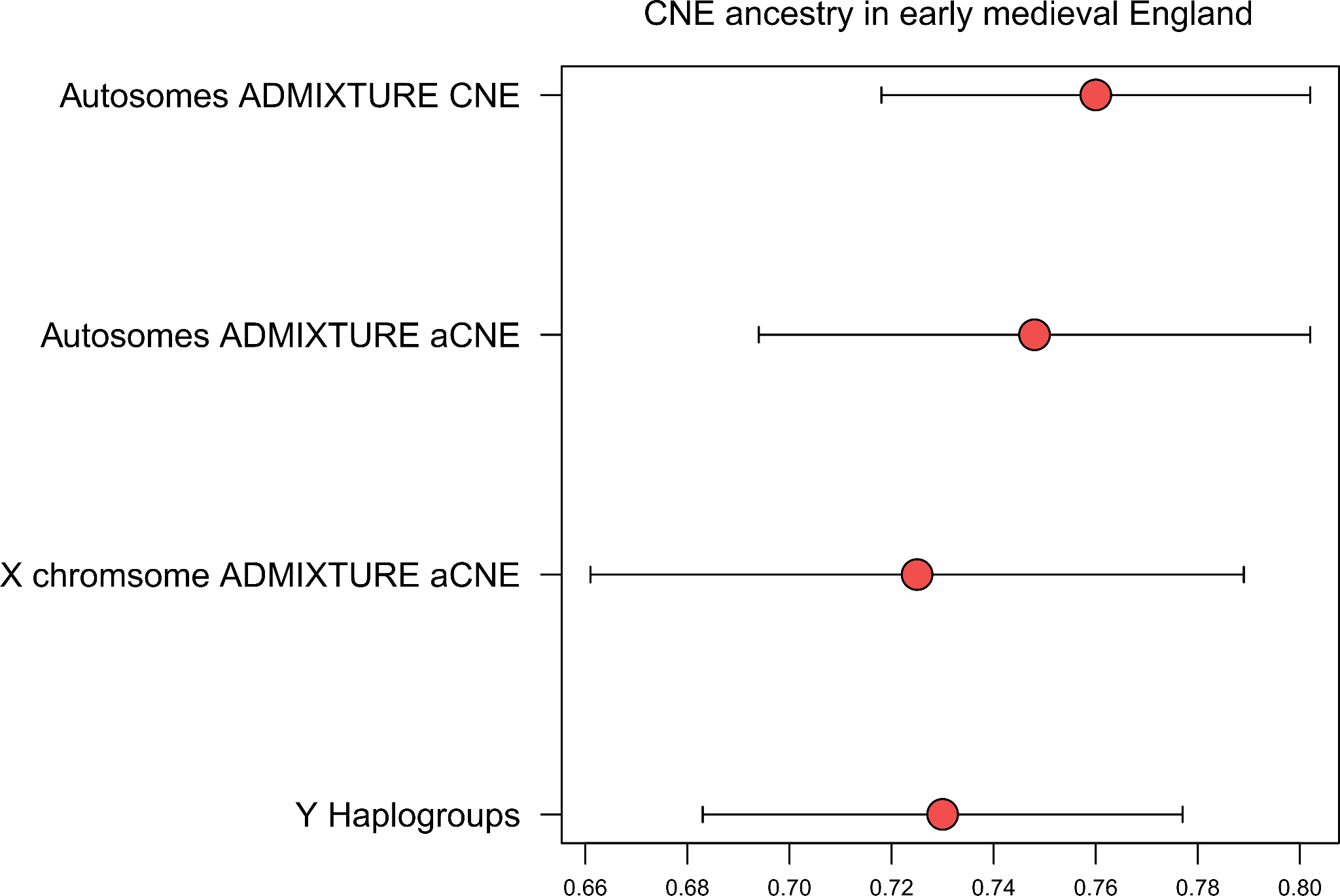
*

***Supplementary Figure 2.11, CNE ancestry in a subset of early medieval English individuals.*** *Data are presented as mean supervised ADMIXTURE estimates (autosomes and X chromosome, n = 195) or maximum likelihood (Y chromosome, n = 89) estimates. Error bars represent ± 2 standard errors of the mean.*

### Estimating sex-biased ancestry from uniparental markers in the presence of variable admixture proportions

We used a third approach to quantify potential sex bias in admixture using uni-parental markers (Y-chromosome- and mitochondrial haplotypes). Specifically, we consider admixed early-medieval individuals from England, for which we have estimated CNE-WBI admixture proportions. We would like to use the observed uniparental markers in these individuals, and the frequency of these haplogroups in the putative source populations, to compare the likelihood of drawing a male (or female) CNE haplogroup with the autosomal admixture proportion. In the following, we denote WBI as source 1, and CNE as source 2.

We first will derive a simple calculation of the likelihood for whether a given haplotype was inherited from one group or another group, based on the sample counts of that haplogroup in the given source population. We first assume that we know precisely the population haplogroup frequencies in both sources, denoted by $x_{ij}$ for haplogroup $i=1\ldots H$ in source $j=1,2$ (we use $x$ to denote the set of $\{x_{ij}\}$). The likelihood for an individual to carry haplogroup $H=i$ contributed from source $S=j$ would then simply be

$L\left( H=i,S=j|x \right)=x_{ij}$.

But the true population haplogroup frequencies are not known. Rather, we have a finite number of individuals in both sources. Specifically, we use $N_{ij}$ to denote the number of individuals with haplogroup $i$ in source $j$ (and $N$ to denote the entire matrix). The unobserved population frequency is now a latent variable following a conditional distribution, $p\left( x|N \right)$, which - following standard Bayesian computation - takes the form of a beta distribution with parameters $1+N_{ij}$ and $1+N_{.j}-N_{ij}$, where $N_{.j}=\sum_{i} N_{ij}$:

$L\left( x|N \right)=Beta\left( x|1+N_{ij},1+N_{.j}-N_{ij} \right)$

which means that the full likelihood is a beta-binomial distribution with sample size 1, which can be written using beta functions:

$L\left( H=i,S=j|N \right)=\int_{0}^{1} x Beta\left( x|1+N_{ij},1+N_{.j}-N_{ij} \right)dx=\frac{B\left( 2+N_{ij},1+N_{.j}-N_{ij} \right)}{B\left( 1+N_{ij},1+N_{.j}-N_{ij} \right)}$

We can now compute the probability $P(s|N)$ that a given target haplogroup was contributed from Source $s$ using Bayes rule:

$p\left( s|N \right)=\frac{L\left( H=i,S=s|N \right)}{\sum_{j} L\left( H=i,S=j|N \right)}$

Here is a summary breakdown of the entire data into groups:

datj %>% dplyr::group_by(Pop2) %>% dplyr::summarise(n = dplyr::n())

## # A tibble: 16 × 2
## Pop2 n
## <chr> <int>
## 1 England_BA 140
## 2 England_EMA 294
## 3 England_IA 304
## 4 France_MA 17
## 5 Ireland_BA 3
## 6 Ireland_MA 39
## 7 NorthSea_BA 37
## 8 NorthSea_IA 19
## 9 NorthSea_MA 209
## 10 Scandinavia_BA 4
## 11 Scandinavia_IA 7
## 12 Scandinavia_MA 180
## 13 Scotland_BA 28
## 14 Scotland_IA 25
## 15 Wales_BA 7
## 16 Wales_IA 7

We now declare three groups of samples. First, a group of admixed individuals from England_EMA, defined as individuals with CNE (continental northern European) admixture proportions between 2 and 98%. Then two source groups: one consisting of individuals from Britain before the study period plus early medieval individuals with less than 2% CNE admixture (Source 1), and one consisting of individuals from Britain with more than 98% CNE ancestry and those sampled from across the North Sea (Source 2).

####

#### Y-chromosomes

We can now get the Y-chromosome haplogroup frequencies in the two sources, and compute the probability of that haplogroup to come from source 2:

## # A tibble: 14 × 4
## Y_1 n1 n2 source2_prob
## <chr> <int> <int> <dbl>
## 1 G 5 2 0.418
## 2 I1 4 61 0.947
## 3 I2a1a 3 3 0.590
## 4 I2a1b 10 14 0.662
## 5 J 1 4 0.782
## 6 R1b 19 7 0.365
## 7 R1b-L151 5 3 0.489
## 8 R1b-L51 10 3 0.343
## 9 R1b-P312 206 24 0.148
## 10 R1b-U106 3 59 0.956
## 11 E 0 3 0.852
## 12 I 0 3 0.852
## 13 N 0 1 0.742
## 14 R1a 0 10 0.940

None of the probabilities is lower than 5% or higher than 95%, suggesting that none of these haplogroups by themself is strictly indicative of male ancestry from a specific source. Nevertheless, we can view these probabilities as a function of autosomal admixture proportion:


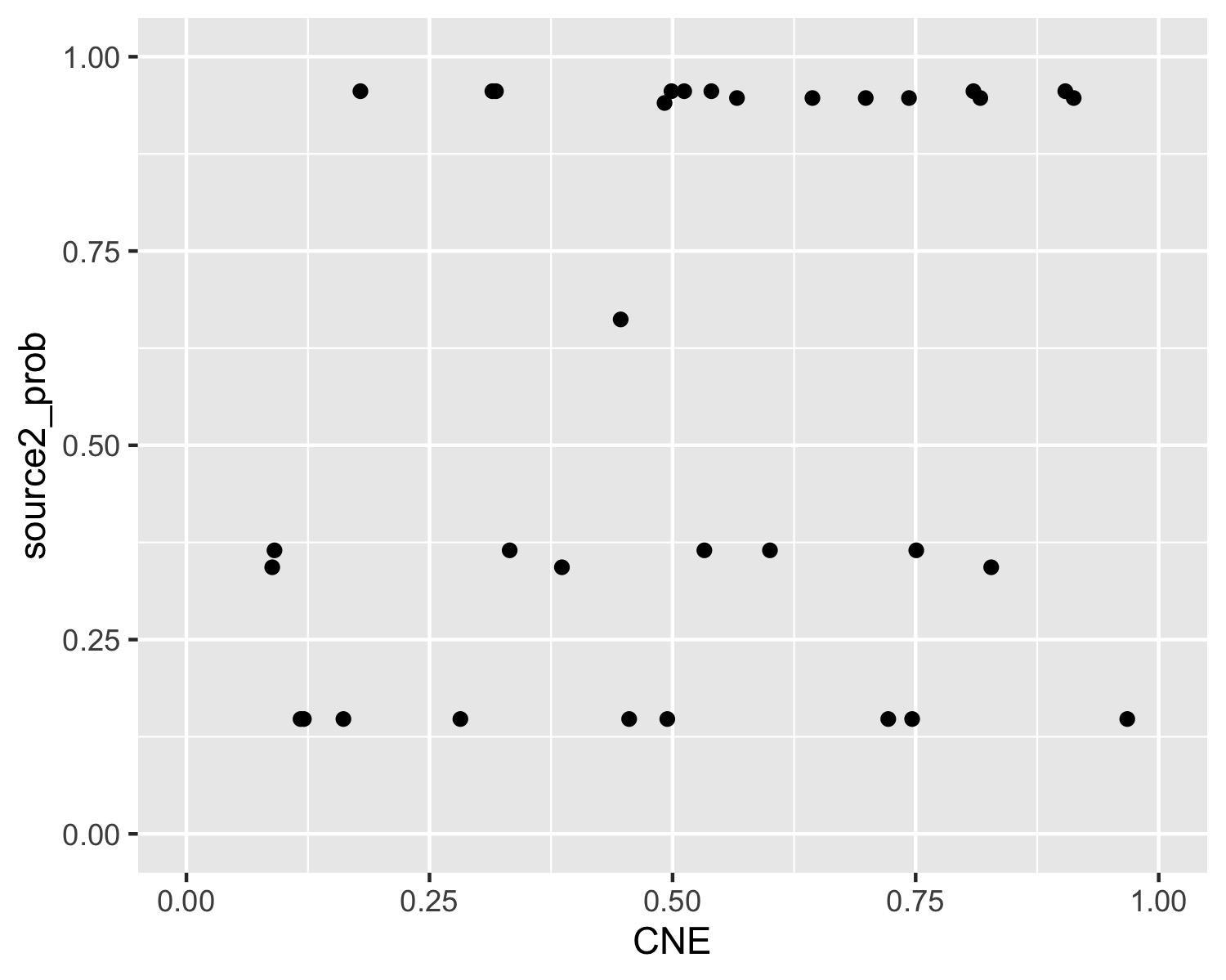


***Supplementary Figure 2.12, Y-chromosome haplogroup origin probabilities as a function of autosomal aCNE admixture proportion.***

To understand this data, we set up a model with two parameters: The first parameter, $\alpha$, determines the amount of an individual’s CNE ancestry (Source 2). The second parameter, $\beta$ determines the proportion of males within Source 2, with $1-\beta$ being the proportion of males within Source 1. Here is a schematic:


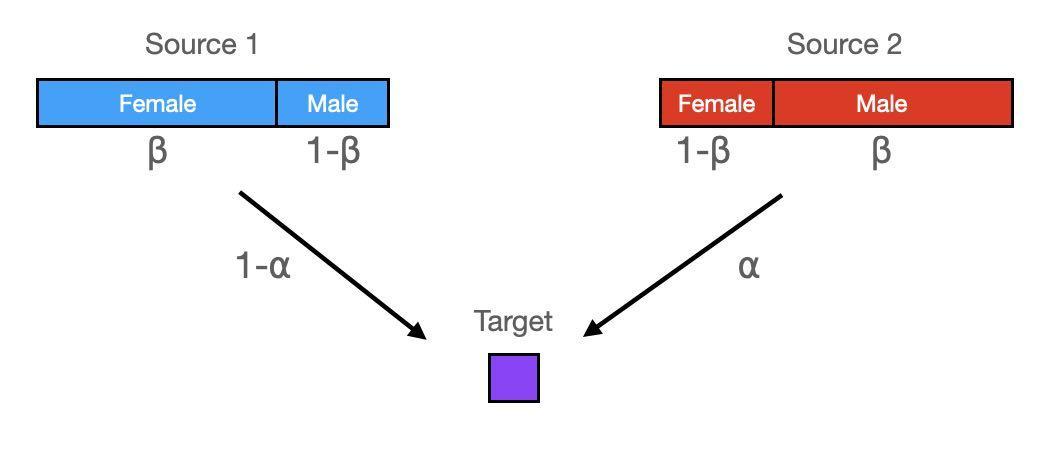


***Supplementary Figure 2.13, Schematic of our sex-bias model and its two parameters α and β.***

This leads to a simple calculation of male Source2-related ancestry over total male ancestry, $\gamma$:

$\gamma\left( \alpha,\beta\right)=\frac{\beta\alpha}{\beta\alpha+\left( 1-\beta\right)\left( 1-\alpha\right)}$

We can visualize this model as follows:


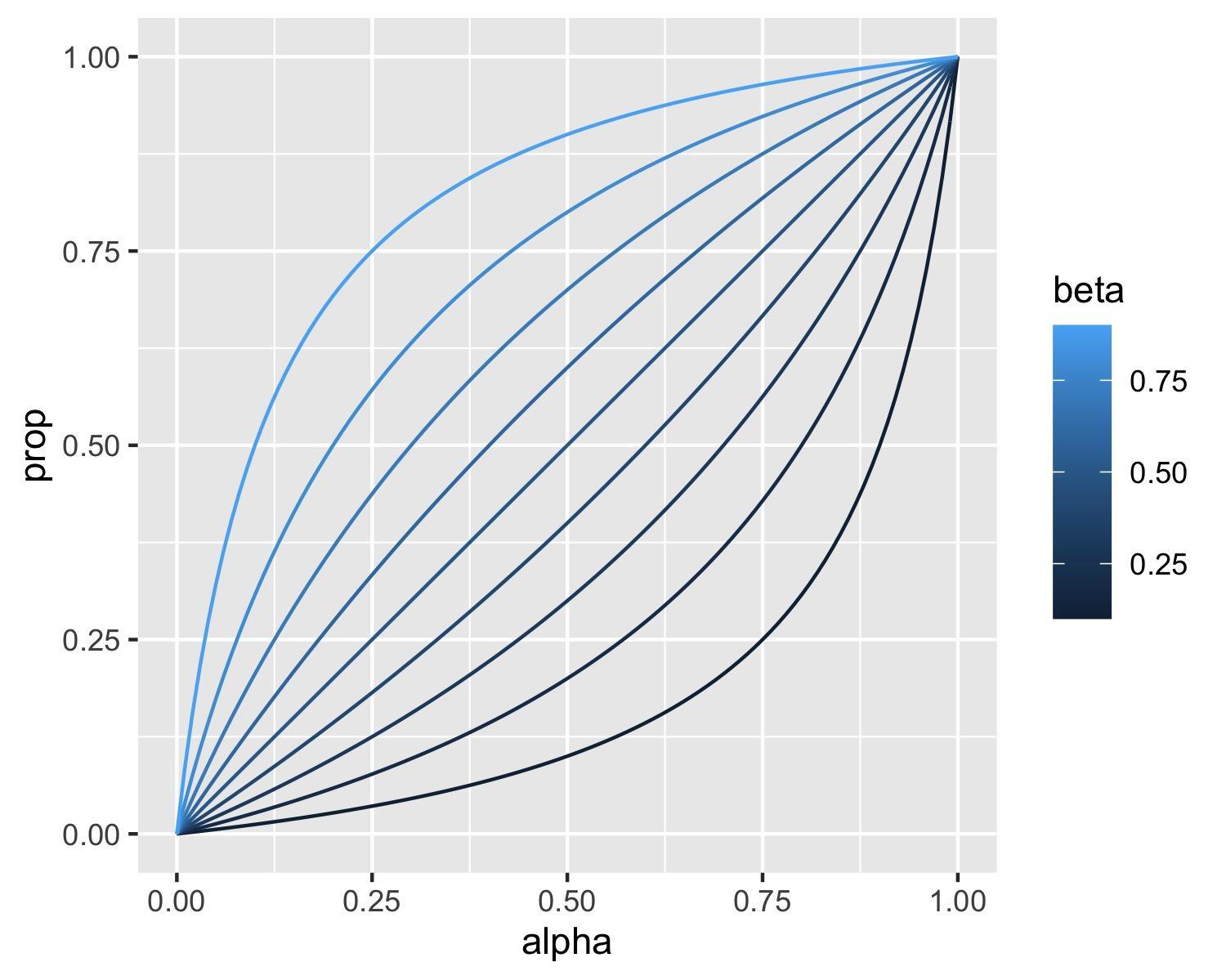


***Supplementary Figure 2.14, showing the proportion of male ancestry from Source 2 over total male ancestry, as a function of the admixture proportion alpha, for different values of the sex-bias parameter beta.***

which shows that values of $\beta$ different from 0.5 can increase or decrease the male admixture proportion and deviate the curve from a model without sex-bias (a straight line).

In order to estimate $\beta$ from the data, we use our above derived individual-based likelihood model of a haplogroup coming from Source2. The likelihood for a single individual with haplogroup H and an autosomal admixture estimate $\alpha$ is

$L\left( H|\alpha,\beta,N \right)=\gamma\left( \alpha,\beta\right)p\left( 2|N \right)+\left( 1-\gamma\left( \alpha,\beta\right) \right)p\left( 1|N \right)$

where we have used the individual-based posterior probability derived above.

We can now compute the log-likelihood as a sum over all male admixed individuals

$log L\left( \{H_{k}\},\{\alpha_{k}\}|\beta,N \right)=\sum_{k} log p\left( H_{k}|\alpha_{k},\beta,N \right)$

where index $k$ runs over all individuals with haplotype $H_{K}$ and autosomal admixture estimate $\alpha_{k}$.

We can estimate the maximum of that likelihood function under variation of $\beta$ numerically, and find

## $maximum
## [1] 0.7242914
##
## $objective
## [1] -20.51125

We can plot that model together with our frequency-based estimates:


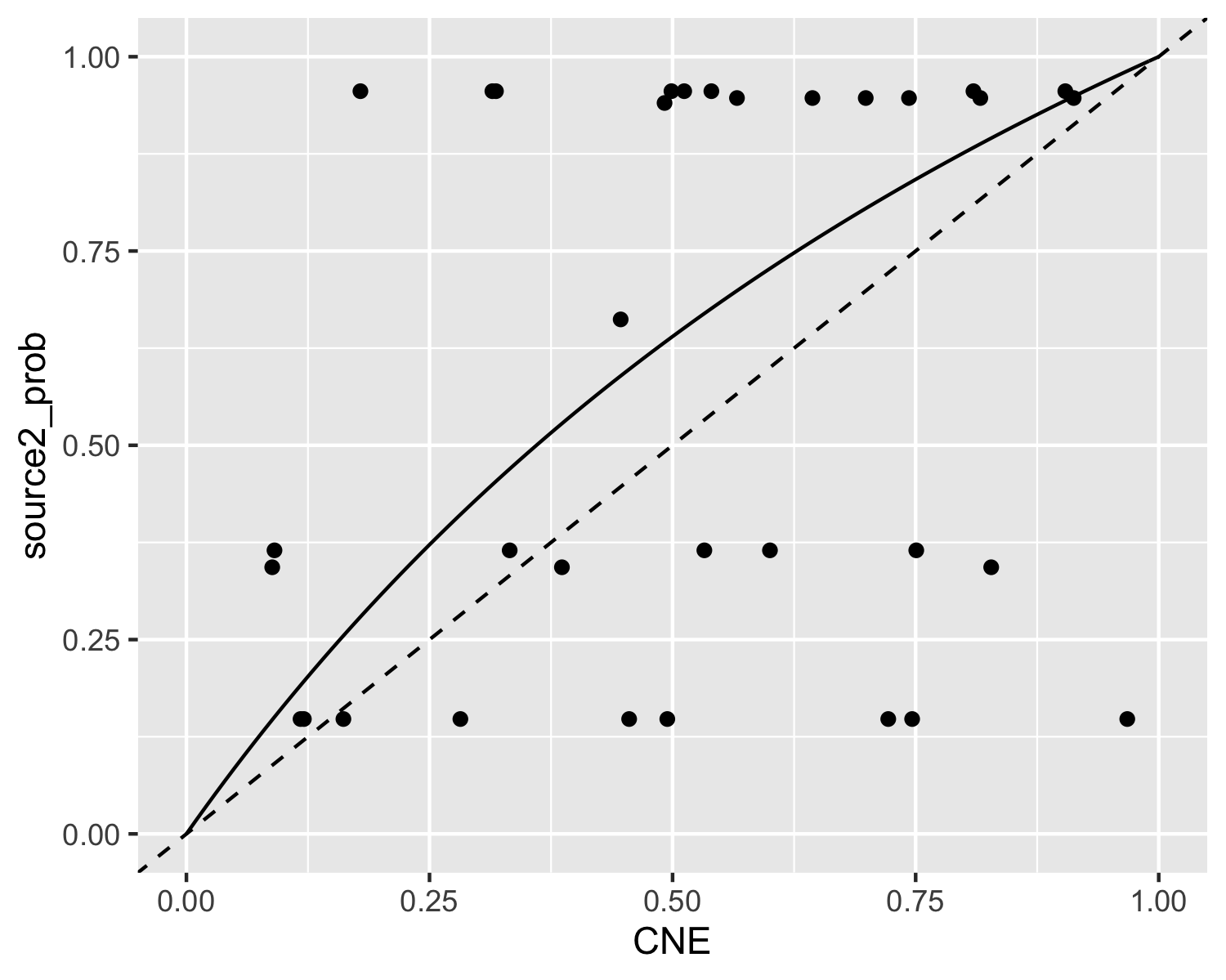


*Supplementary Figure 2.15, showing the best-fitting model (solid line), together with a model with no sex bias (dashed line). The fitting model suggests a slight male sex-bias.*

So, this would suggest a moderate male sex-bias in the CNE ancestry. But how significant is this? We can use our likelihood model to compute a posterior probability density over $\beta$:

$p\left( \beta|\{H_{k}\},\{\alpha_{k}\},N \right)=\frac{L\left( \{H_{k}\},\{\alpha_{k}\}|\beta,N \right)}{\int_{0}^{1} L\left( \{H_{k}\},\{\alpha_{k}\}|\beta,N \right)d\beta}$

where the integral in the denominator can be approximated using a mesh over $\beta$, and we have implicitly taken a uniform prior for $\beta$ over the interval from 0 to 1:


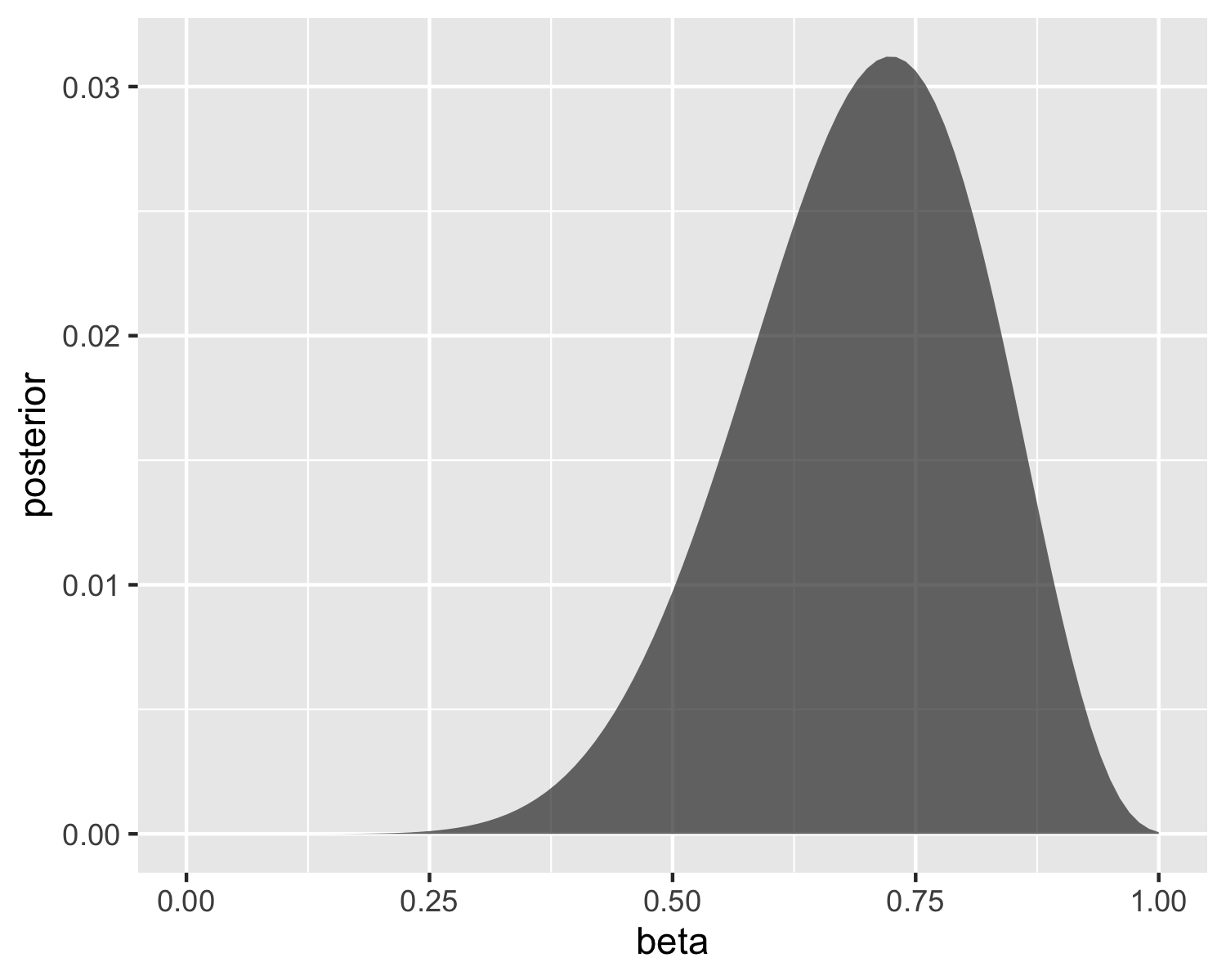


***Supplementary Figure 2.16, Posterior distribution for β, including β=0.5 which is a model without sex-bias.***

We can now see that the posterior distribution for $\beta$ comfortably includes the value $\beta=0.5,$ which means that the 95% credibility interval includes values below and above 0.5, suggesting models of male as well as female sex-bias being consistent with the data. We conclude that the Y haplotype data is not conclusive with respect to sex bias.

#### Mitochondrial haplogroups

We can apply the same methodology to mitochondrial (MT) haplotypes, with the main difference being the interpretation of $\gamma\left( \alpha,\beta\right)$ now describing the proportion of *female* Source2 ancestry over total female ancestry.

We get a similar table of posterior probabilities for every mtDNA haplogroup coming from source 2:

## # A tibble: 12 × 4
## mtDNA_simple n1 n2 source2_prob
## <chr> <int> <int> <dbl>
## 1 H 206 134 0.484
## 2 HV 5 9 0.705
## 3 I 11 8 0.519
## 4 J 52 47 0.565
## 5 K 66 35 0.436
## 6 N 2 1 0.489
## 7 R 7 1 0.264
## 8 T 54 41 0.523
## 9 U 91 55 0.466
## 10 V 18 11 0.476
## 11 W 7 4 0.473
## 12 X 11 5 0.418

which for our admixed individuals can be visualised again:


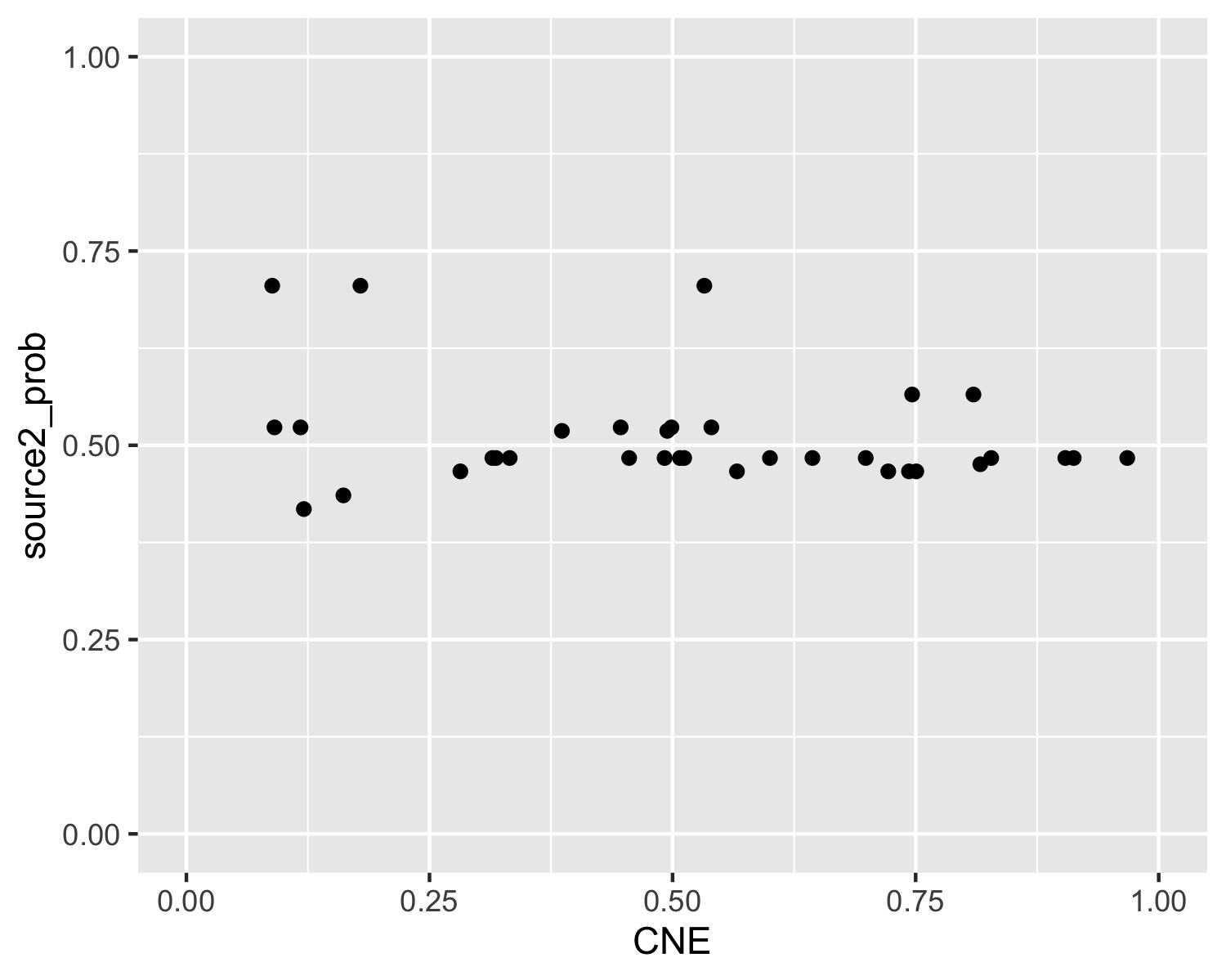


***Supplementary Figure 2.17, mtDNA haplogroup origin probabilities as a function of autosomal aCNE admixture proportion.***

but with all probabilities around 50%, the MT data conveys almost no information at all about source origin, since haplogroup frequencies are too similar in the two source populations.

Indeed, the posterior distribution of $\beta$ in this case yields:


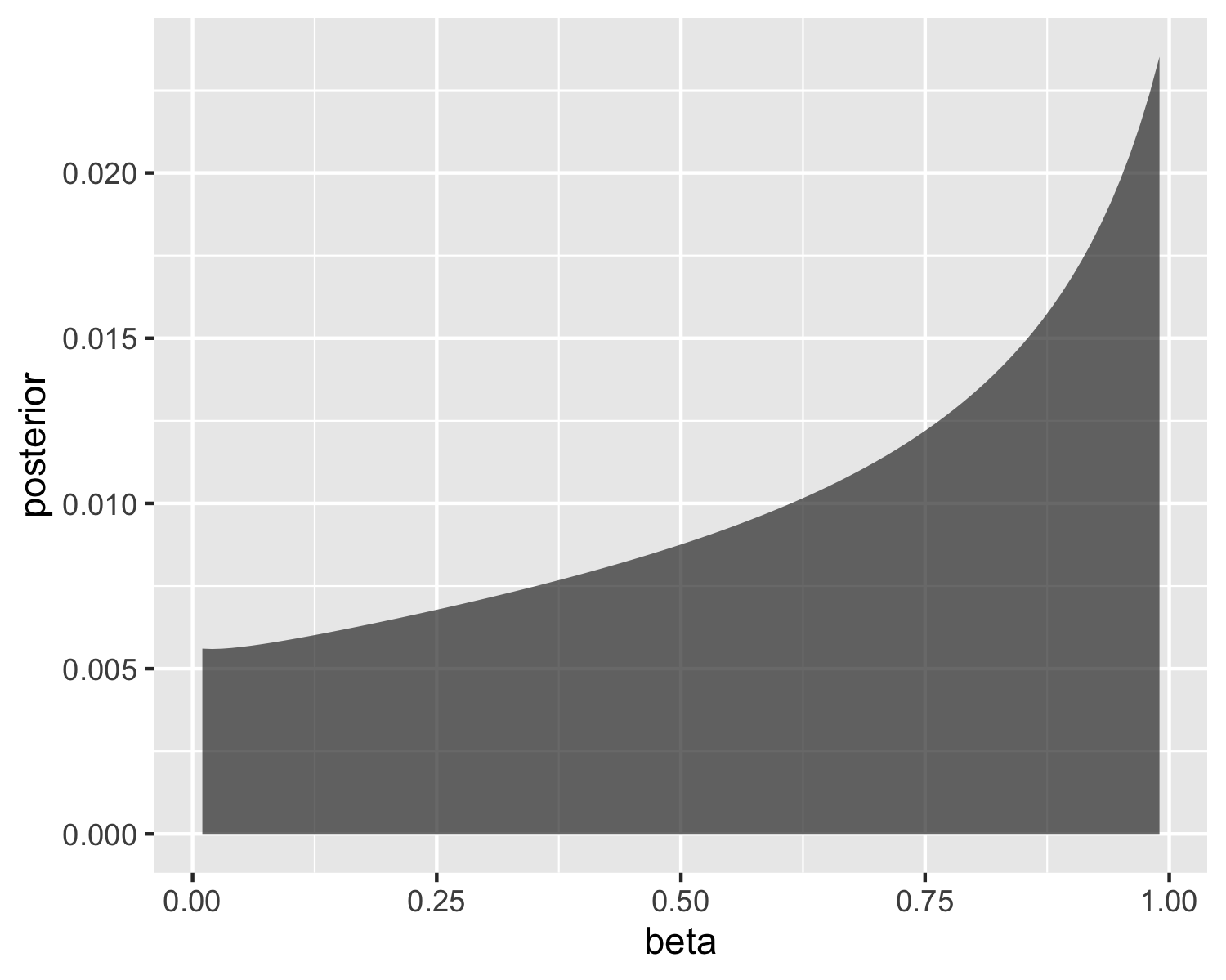


***Supplementary Figure 2.18, Posterior distribution for β.***

which is even broader than the already insignificant results from the Y-chromosomal haplogroups, again showing that the data is consistent with models of both female and male sex-bias.

Note: The code used here can be found at <https://github.com/stschiff/AngloSaxon_Y-chrom_sex-bias>

#

#

# Supplementary Note 3 *Continental affinities in early medieval samples from England*

Our analysis of immigrant vs. local genetic ancestry in early medieval England follows a multi-step approach using a variety of methods. Because of space limitations in the main article, we here describe in full detail the steps involved.

## General population genetic affinities

To assess the population genetic affinities of early medieval samples from England we performed two principal component analyses[^78^](https://paperpile.com/c/hqnLqQ/LPCGB), as described in *Methods*. Both the *North Sea PCA* as well as the *Northwestern European PCA* imply strong genetic similarity between the early medieval genomes from England and i) present-day genomes of northern Germans and Danish as well as ii) medieval genomes from the Netherlands, northern Germany, and Denmark (Fig. 2, Ext. Data Fig 6).

The positioning in the Principal Component analyses was also reflected in unsupervised clustering using ADMIXTURE[^172^](https://paperpile.com/c/hqnLqQ/TNMqU). We ran unsupervised ADMIXTURE on the present-day data and projected the ancient data on the learned allele frequencies (Supp. Fig. 3.1a for *K* = 5 and Supp. Fig. 3.1b for *K* = 6). At *K* = 5, early medieval genomes from England harbour two major genetic components, one “central and northern European” component maximized in ancient individuals from Lower Saxony (especially Schortens, Dunum, and Drantum) as well as present-day Scandinavians (Norway and Sweden), and one “Insular Celtic” component maximized in Iron Age English and medieval Irish as well as present-day Irish, Welsh, and Scottish. Hierarchical clustering of Euclidean distances calculated on the inferred proportions using Ward's minimum variance method (ward.D2) verifies that the early medieval English form a clade with medieval and present-day continental northern European populations and not with ancient and/or modern British-Irish populations (Supp. Fig. 3.2a). At *K* = 6, the “central and northern European” component splits into a “Scandinavian” component (maximised in modern Swedes and Norwegians) and a “North Sea” component, which is maximised in ancient samples from Lower Saxony, as well as in modern Dutch, Danish, English, and Northern Germans (Supp. Fig. 3.2b). Remarkably, modern English display a mosaic of different components, with the “North Sea” component showing the largest proportion. Furthermore, we detect a large “Scandinavian” and “Insular Celtic” component as well as a substantial “southern European” introgression (Supp. Fig. 3.2b).


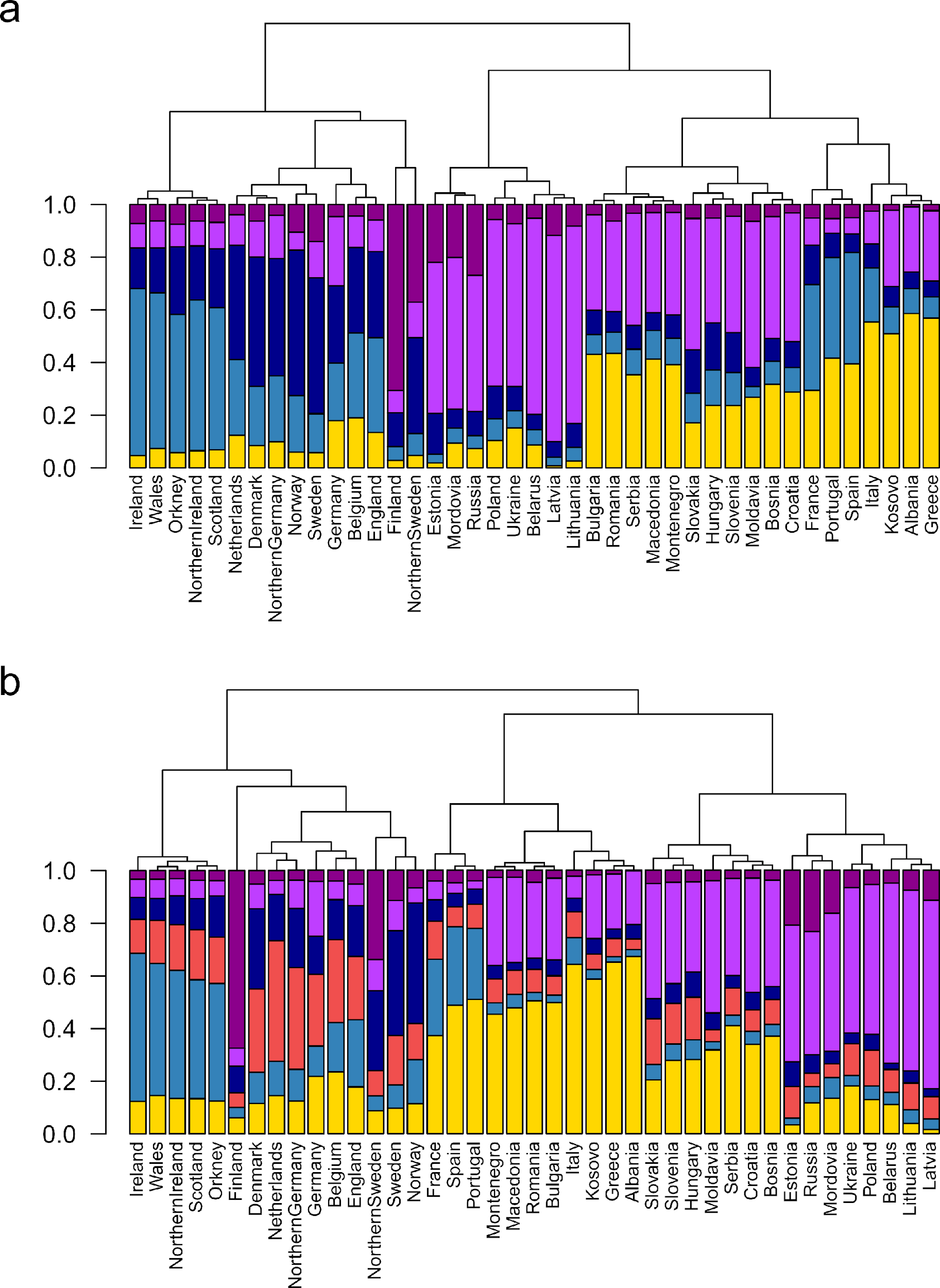


***Supplementary Figure 3.1, Unsupervised ADMIXTURE analysis of present-day samples.*** *Hierarchical clustering of Euclidean distances calculated on the mean ancestry component proportions from unsupervised ADMIXTURE analysis, using Ward's minimum variance method (ward.D2) presented as a phylogram. The bar chart below each branch shows the mean ancestry proportions from unsupervised ADMIXTURE for the respective population. a) For K = 5. b) For K = 6.*


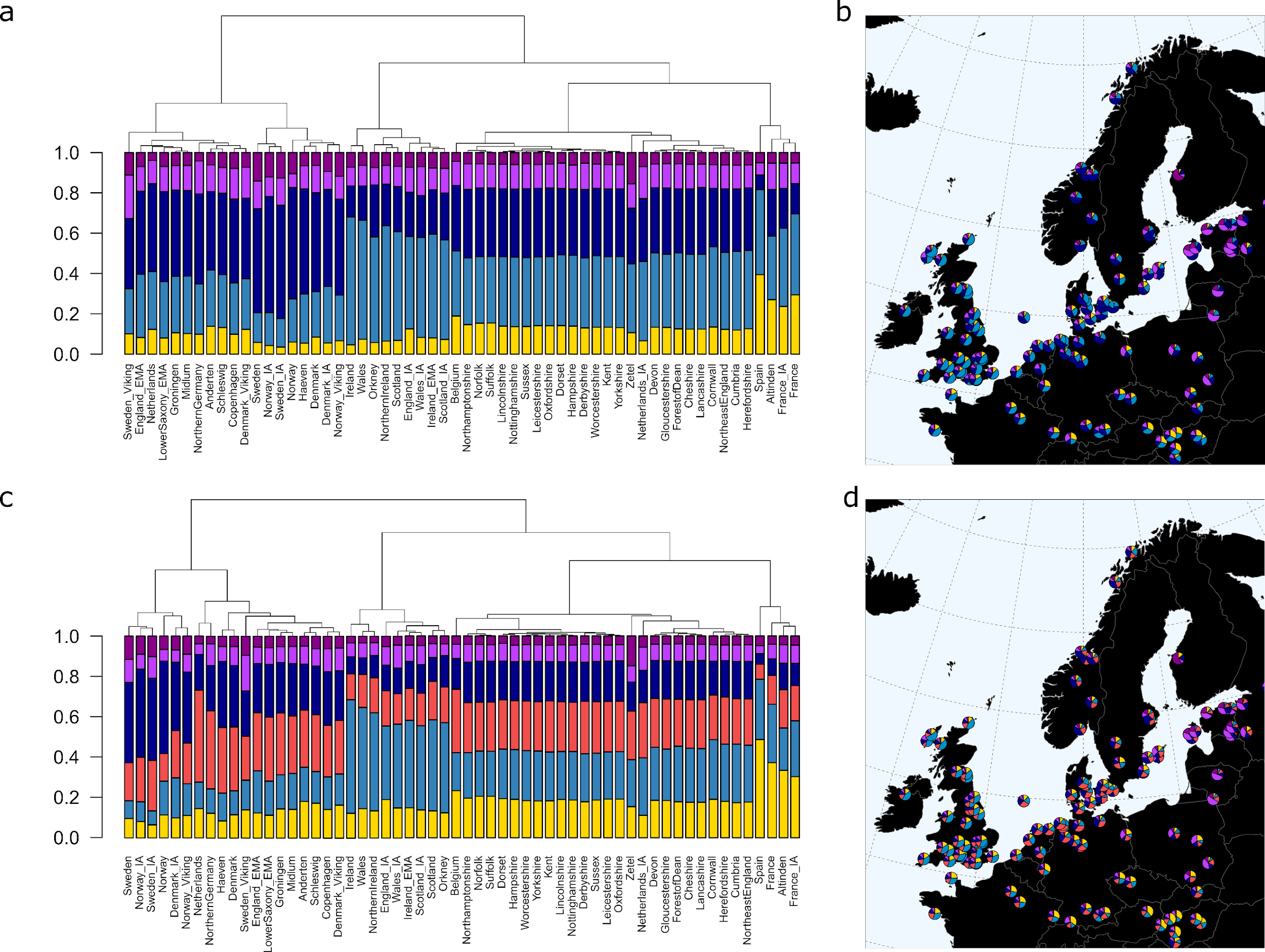


***Supplementary Figure 3.2, Unsupervised ADMIXTURE analysis of present-day and ancient samples.*** *Hierarchical clustering of Euclidean distances calculated on the mean ancestry component proportions from unsupervised ADMIXTURE projection analysis, using Ward's minimum variance method (ward.D2) presented as a phylogram. The bar chart below each branch shows the mean ancestry proportions from unsupervised ADMIXTURE for the respective population. The averaged proportions are also depicted as pie charts on top of their respective geographical origin. a) For K = 5 at the population level. b) For K = 5 at the site level. c) For K = 5 at the population level. d) For K = 5 at the site level.*

The affinity to present-day populations from the North Sea area was verified using i) F_ST_ (Methods) calculated between all early medieval genomes from England grouped together and a set of present-day European populations (Extended Data Fig. 1a, Supp. Table 3.1), ii) *F*_4_ statistics[^174^](https://paperpile.com/c/hqnLqQ/5aYUV) (Methods) of the form *F*_4_(YRI, *TestA*; Ireland, *TestB*) and of the form *F*_4_(YRI, *TestA*; Denmark, *TestB*), where *TestB* iterates through 11 present-day European populations and *TestA* iterates through 9 ancient and present-day English populations (Extended Data Fig. 1c and Extended Data Fig. 1d, Supp. Table 3.4 and 3.4) and iii) *F*_4_ statistics of the form *F*_4_(YRI, *X*; Denmark, *Test*) (Supp. Fig. 3.3a, Supp. table 3.5) and of the form *F*_4_(YRI, *X*; Ireland, *Test*) (Supp. Fig. 3.3b, Supp. Table 3.6), where *Test* iterates through 13 European populations and *X* iterates through each ancient genome from England individually (*n* = 728) (corresponding *Z*-values are depicted in Supp. Fig. 3.3a and Supp. Fig. 3.3b, respectively). While our early medieval genomes from England were significantly closer related to present-day northern Germans, Danes, and English than to any other population, Iron and Roman Age individuals showed higher similarity to present-day Irish, northern Irish, Scottish and Welsh. We therefore consider these populations to be the closest modern proxies for the respective ancient populations.


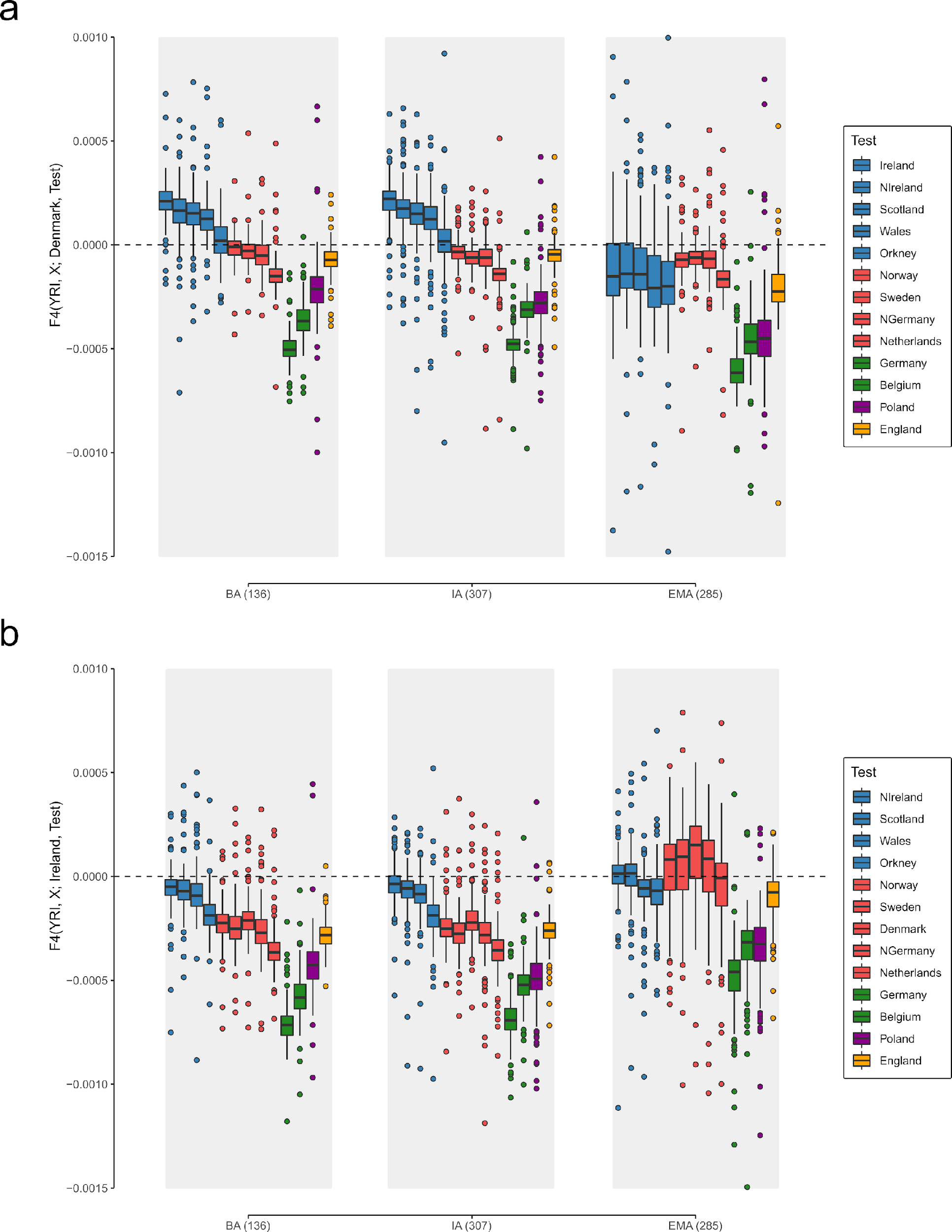


***Supplementary Figure 3.3, Individual-based population affinities through time****. a) F_4_ statistics of the form F_4_(YRI, X; Denmark, Test), where Test iterates through European populations (n = 13) and X iterates through each ancient post-Neolithic genome from England individually (n = 728) with the Yoruba from Nigeria (YRI) as an outgroup (n = 91). F_4_ values are shown as boxplots. b) Same as in a) but for the F_4_ statistic of the form F_4_(YRI, X; Ireland, Test). Bounds of the Box represent the 25^th^ and 75^th^ Percentile. The centre represents the median. Whiskers represent the smallest value greater than the 25^th^ Percentile minus 1.5 times the interquartile range and largest value less than the 75^th^ Percentile plus 1.5 times the interquartile range, respectively. Outliers present the minimum and maximum values in the data.*


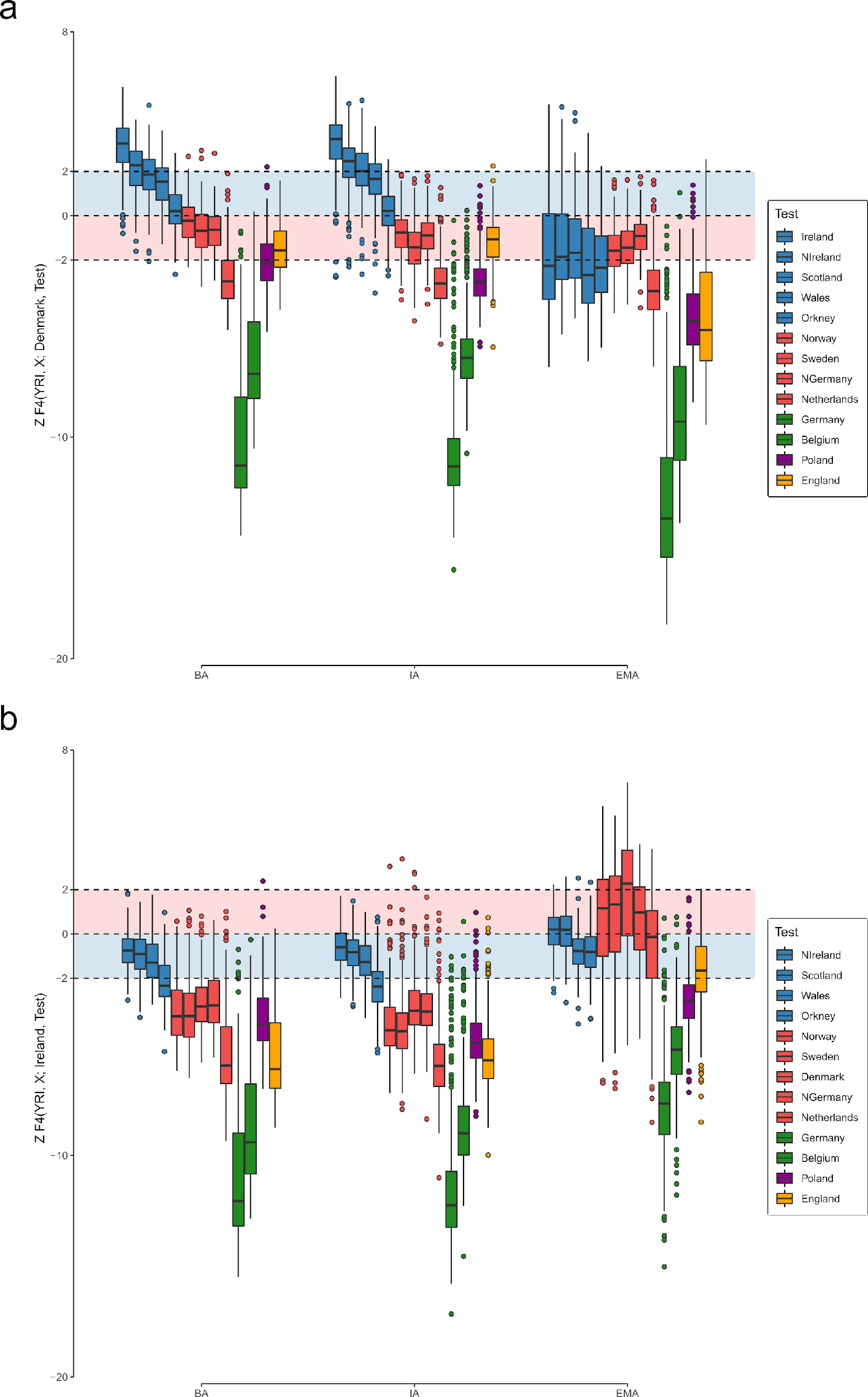


***Supplementary Figure 3.4, Individual-based population affinities through time****. a) Z-values from F_4_ statistics of the form F_4_(YRI, X; Denmark, Test), where Test iterates through European populations (n = 13) and X iterates through each ancient post-Neolithic genome from England individually (n = 728) with the Yoruba from Nigeria (YRI) as an outgroup (n = 91). Z-values are shown as boxplots. b) Same as in a) but for the F_4_ statistic of the form F_4_(YRI, X; Ireland, Test). Bounds of the Box represent the 25^th^ and 75^th^ Percentile. The centre represents the median. Whiskers represent the smallest value greater than the 25^th^ Percentile minus 1.5 times the interquartile range and largest value less than the 75^th^ Percentile plus 1.5 times the interquartile range, respectively. Outliers present the minimum and maximum values in the data.*

## Estimating continental admixture

To approximate the ancient admixture sources, we grouped present-day northern Germans and Danes together and labelled this metapopulation as “*continental northern European*” (CNE, *n* = 407). Similarly, we grouped present-day Irish, northern Irish, Welsh, and Scottish (excluding Orcadians), together as “*Western British and Irish Isles*” (WBI, *n* = 607). We then used ADMIXTURE[^172^](https://paperpile.com/c/hqnLqQ/TNMqU) in supervised mode, where we estimated admixture proportions for the prehistoric and early medieval genomes from England using those two reference populations with *K* = 2 (Fig. 3, Extended Data Fig. 2, Supp. Table 3.7). These analyses were run on haploid data and bootstrapped 1,000 times to calculate standard errors for each estimate. The obtained ancestry estimates were highly correlated with PC1 and PC2 position as well as *F*_4_ statistics of the form *F*_4_(YRI.SG, X; WBI, CNE)) (Supp. Fig. 3.5, Supp. Table 3.7), which indicates that the measured proportions indeed reflect true ancestry components. Standard errors (Methods) for the individual estimates were mainly associated with the coverage of the modelled ancient individual, with the standard errors ranging between 0% and 44.9% (the mean standard error for the individual estimates of the 885 modelled samples was 3.5% ± 7.1% SD, and the median standard error 0%).


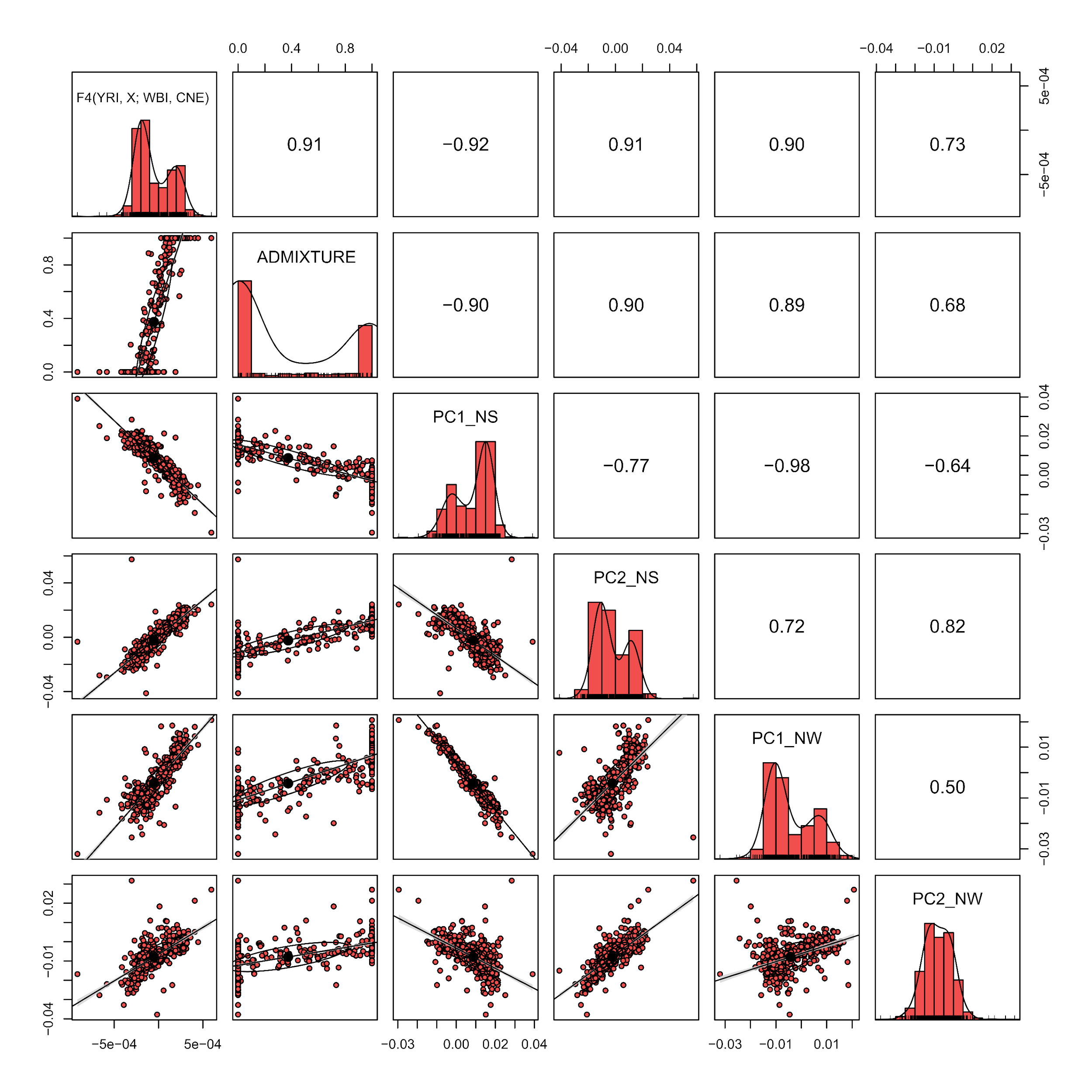


***Supplementary Figure 3.5,*** ***Validation of CNE ancestry estimates.*** *Correlogram illustrating pairwise correlations between different measurement of genetic affinity to CNE and WBI (supervised ADMIXTURE estimates at K = 2, F_4_ statistics of the form F_4_(YRI, X; WBI, CNE), PC1, and PC2 coordinates in our North Sea and Northwestern European PCA) for 585 Iron Age, Roman Period, and early medieval English individuals. Pearson’s r for each pairwise correlation test are indicated in the upper triangle of the matrix.*

## Specific population genetic affinities

We then identified a subset of 109 (unrelated) individuals from the Early Anglo-Saxon Period which exhibited more than 95% CNE ancestry, labelling them as *England_EMA_CNE*. This subset is intended to most closely resemble the immigrant population prior to admixture with the local British gene pool. To infer the population genetic affinities of this population we compared it to several published and our new medieval continental samples from northern Europe. We first calculate i) shared drift from an outgroup via pairwise outgroup *F*_3_ statistics[^174^](https://paperpile.com/c/hqnLqQ/5aYUV) between *England_EMA_CNE* and contemporaneous ancient populations (Extended Data Fig. 5a), ii) symmetry tests via *F*_4_ statistics between ancient and present-day populations (Extended Data Fig. 5b), and iii) a maximum likelihood phylogeny based on *F*_2_ statistics using Treemix v1.12 (Supp. Fig. 3.8). We computed the outgroup *F*_3_ and *F*_4_ statistics in ADMIXTOOLS[^174^](https://paperpile.com/c/hqnLqQ/5aYUV) using the programs *qp3Pop* (with the option inbreed set to YES) and *qpDstats* (with f4mode st to YES), and used Han Chinese from Beijing (CHB.SG) as outgroup population in *F*_3_ and Yoruba in Ibadan as outgroup population in *F*_4_ statistics.

For the *F*_3_ statistics, we translated the shared drift calculated using *f*-statistics of the form *F*_3_(CHB; ancient A, ancient B) into distances (via the formula 1-*F*_3_) and visualised the resulting distance matrix using Multidimensional Scaling (MDS) (Extended Data Fig. 5a).

For the *F*_4_ statistics, we calculated for 71 Bronze Age, Iron Age, and medieval populations all possible combinations of the *f*-statistic *F*_4_(YRI, X; *TestA*, *TestB*). *TestA* and *TestB* iterate through the following 15 present-day European populations:

- Denmark
- Norway
- Sweden
- Finland
- Ireland
- Scotland
- Wales
- Poland
- Lithuania+Latvia+Estonia
- Germany
- Netherlands
- Belgium
- France
- Spain
- Italy

We extracted all unique combinations per ancient population X and then performed PCA on the computed *F*_4_ statistics (Extended Data Fig. 5b, Supp. Table 3.10). We see that in this PCA ancient population position along PC1 and PC2 strongly resembles the mean population location in our Northwestern European PCA calculated on individual present-day genotype data.

To verify this observation, we projected per present-day population five individuals as well as 18 modern English populations onto the PCA (Supp. Fig. 3.6). Positioning of the projected samples highly corresponds with the location in the genotype PCA. As in our Northwestern PCA, the English populations do not form a cline between Iron Age and early medieval samples from England. Since this PCA should not be affected by drift e.g. that accumulated after the Early Middle Ages, we conclude that the position of modern English within the PCA is the product of admixture.

In comparison to medieval and present-day Icelanders[^92^](https://paperpile.com/c/hqnLqQ/9O9CJ), we detect only minor insular-specific (here: England-specific) drift. This is supported by a scatterplot of two *F*_4_-statistics, *F*_4_(CHB, X; CNE, England) and *F*_4_(CHB, X; WBI, England), which should effectively distinguish the signature of England-specific drift (an axis parallel to the diagonal line) from WBI-CNE ancestry (perpendicular deviation from that line) (Supp. Fig. 3.7, Supp. Table 3.8).


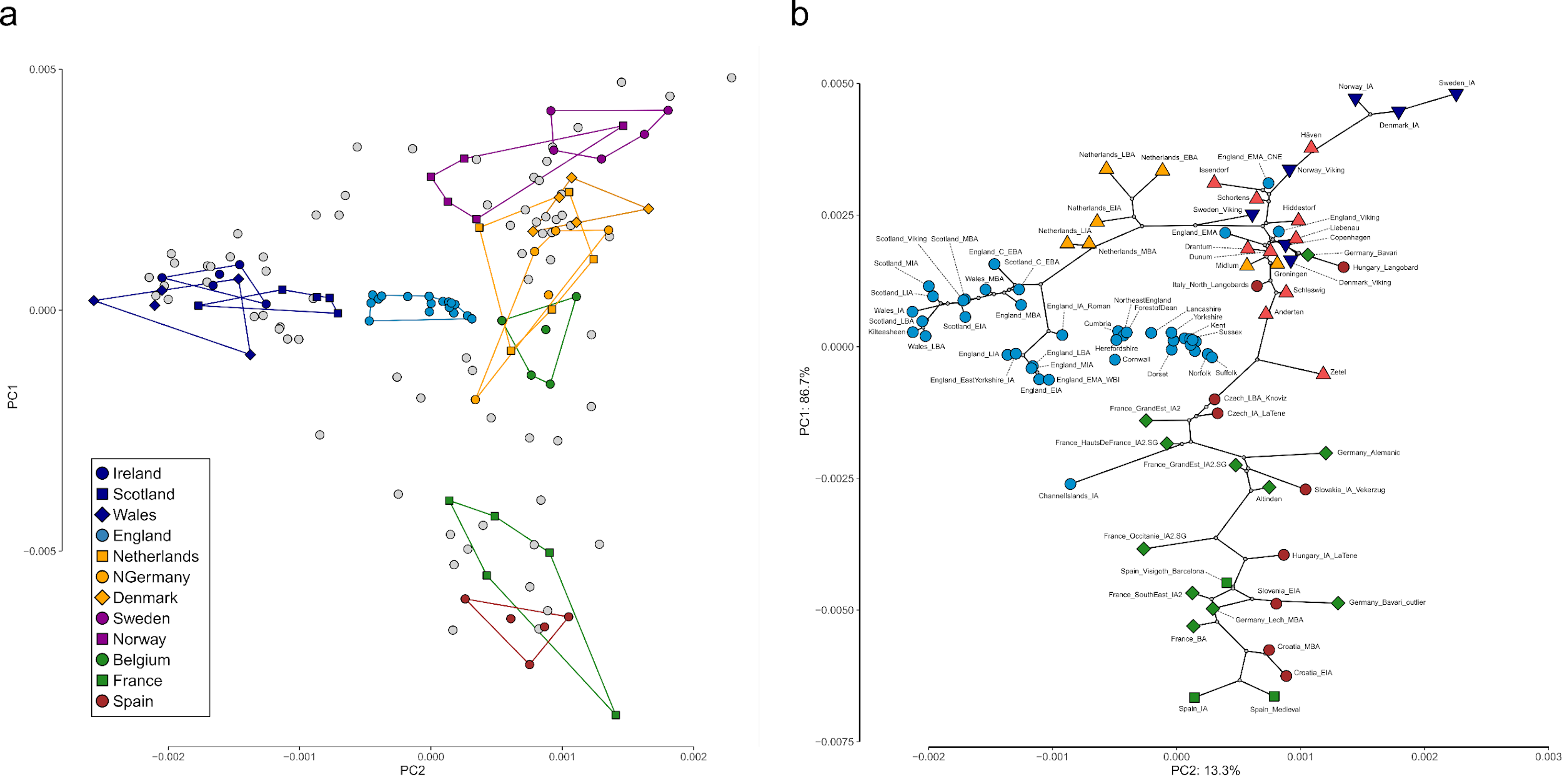


***Supplementary Figure 3.6, Population genetic affinities of present-day Europeans.*** *a) PCA of all unique combinations of the f-statistic F_4_(YRI, X; TestA, TestB). Present-day individuals and populations were projected onto the first two PCs. Ancient populations are shown in grey. b) Highlighted location of 18 present-day English populations within the ancient variation of all unique combinations of the f-statistic F_4_(YRI, X; TestA, TestB).*


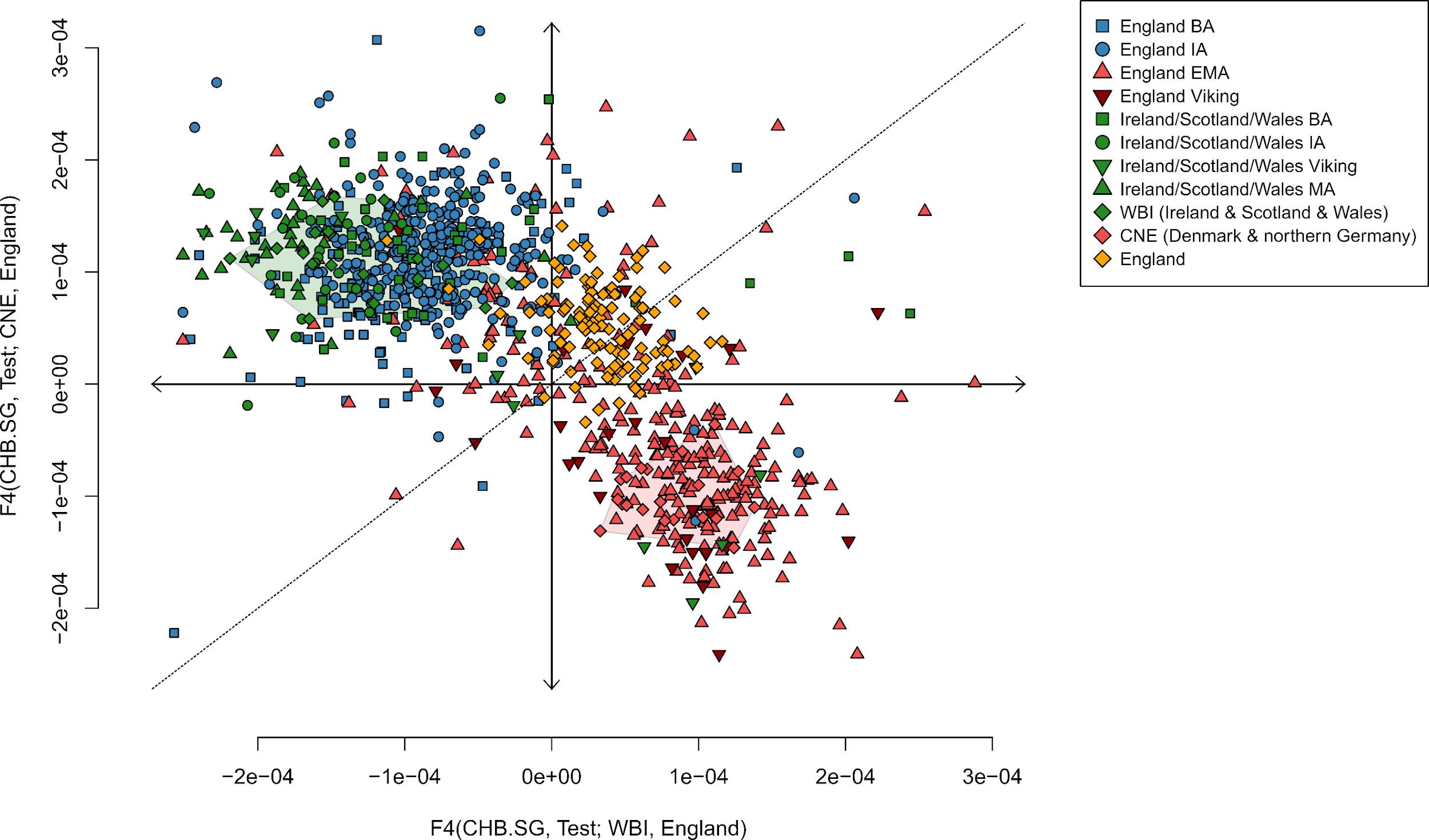


***Supplementary Figure 3.7. England-specific genetic drift.*** *Scatterplot of f-statistics of the form F_4_(CHB, Test; CNE, England) and F_4_(CHB, Test; WBI, England) reflecting England-specific drift. To aid interpretation, we included values for ancient British-Irish Islanders and a subset of contemporary individuals (who were correspondingly removed from the reference populations).*


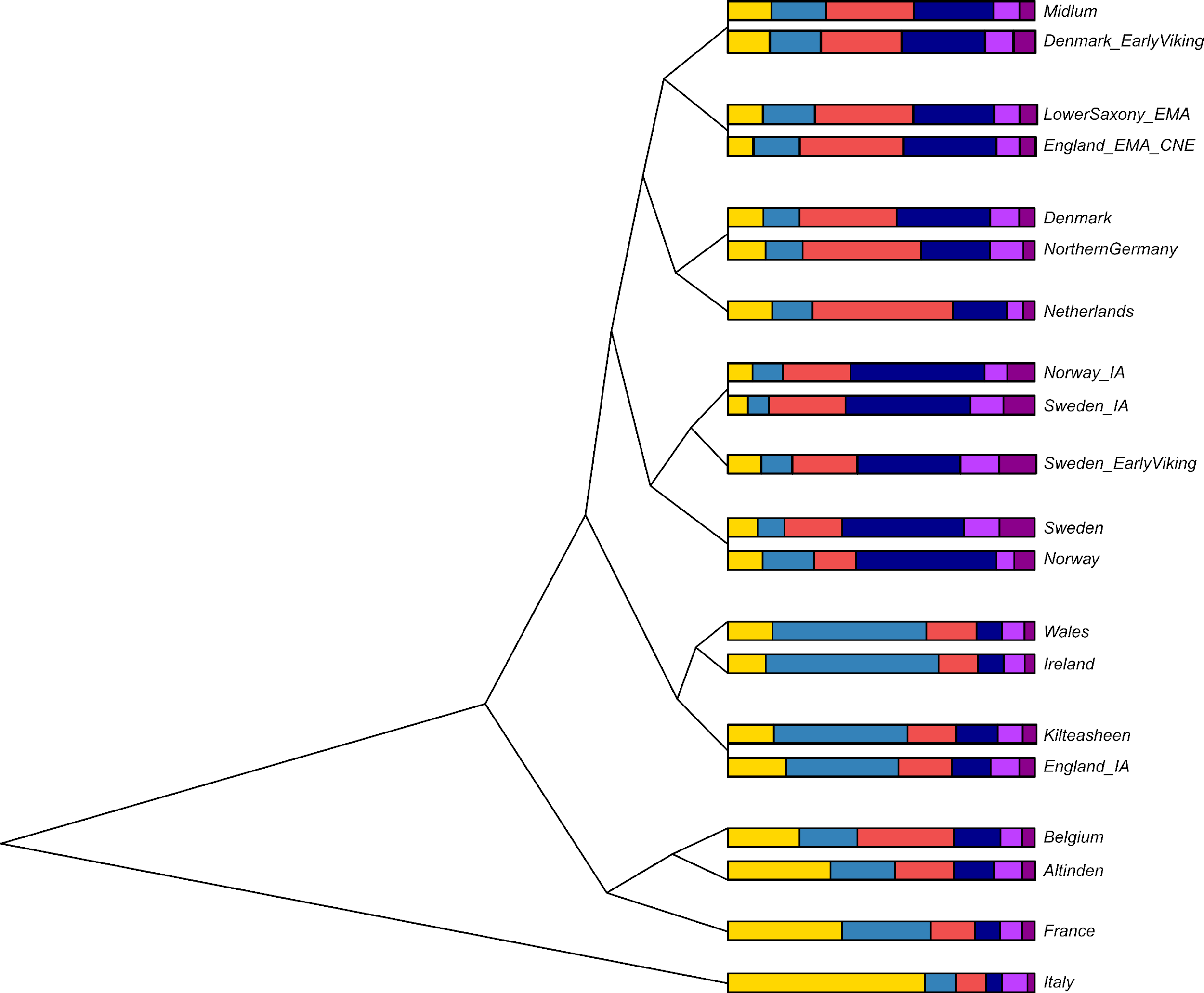


***Supplementary Figure 3.8. Treemix analysis.*** *Maximum likelihood tree of genome sequences from present-day and ancient European populations****.*** *The bar chart next to each population shows the ancestry proportions from unsupervised ADMIXTURE at K = 6 for each population.*

We devised two test-criteria to statistically test whether continental sites could be considered a direct source for the immigrating gene pool in England (as defined in *England_EMA_CNE*). First, we tested on the site-specific level whether the *England_EMA_CNE* individuals were consistent with forming a clade with 77 French, Czech, Polish, Dutch, German, Danish, Swedish, or Norwegian site populations using *qpWave*[*^171,175^*](https://paperpile.com/c/hqnLqQ/egNye+eK1xv) from ADMIXTOOLS^60^ (v.4.1). We set the option allsnps: YES to specify that the *F*_4_ statistics, which are the basis of *qpWave* analyses, should be computed using the union of SNPs with coverage in all four groups that contribute to each *F*_4_ statistic. We used a set of 11 Outgroups in the following order: YRI.SG, Poland, Finland, Sweden, Denmark, Ireland, Wales, Italy, Spain, Belgium, Netherlands (Supp. Table 3.11). Second, as a somewhat weaker condition, we tested which of the 77 aforementioned site populations are suitable proxies for the immigrant ancestry in the whole *England_EMA* population using a two-way model including *England_LIA_Roman* as source of local English ancestry in *qpAdm* (Supp. Table 3.11)*.*

We identify 23 populations in northern Germany, Denmark, and Sweden that fulfil the first criterion, so are indistinguishable from *England_EMA_CNE* in terms of their allele frequencies with respect to the outgroups in *qpWave*. For the second criterion, we found 27 Dutch, northern German, Danish, and Swedish populations as fitting proxies for CNE ancestry in the *England_EMA* population with *qpAdm*, however, only 20 of them produce feasible models with admixture proportions between 0 and 1 (Supp. Fig. 3.9b). Of those 20 populations, all pass the *qpWave* cladality criterion as well. Ten sites are located in Denmark (Jutland, Funen, Langeland, and Seeland), two in Sweden (Oland and Skåne), and eight in northern Germany (Lower Saxony and Mecklenburg-Vorpommern)(Supp. Fig. 3.9a) . We highlight that 40% of the populations that fulfil both criteria are located in northern Germany, although they only represent 13% of the tested sites. In total, eight out of ten (80%) tested northern German sites fulfil the criteria, ten out of 27 (37%) Danish sites, and two out of eleven Swedish sites (18%). The estimates of CNE ancestry within the *England_EMA* population vary between 50% and 93%, with a mean of 76.9% and a SD of 12%.


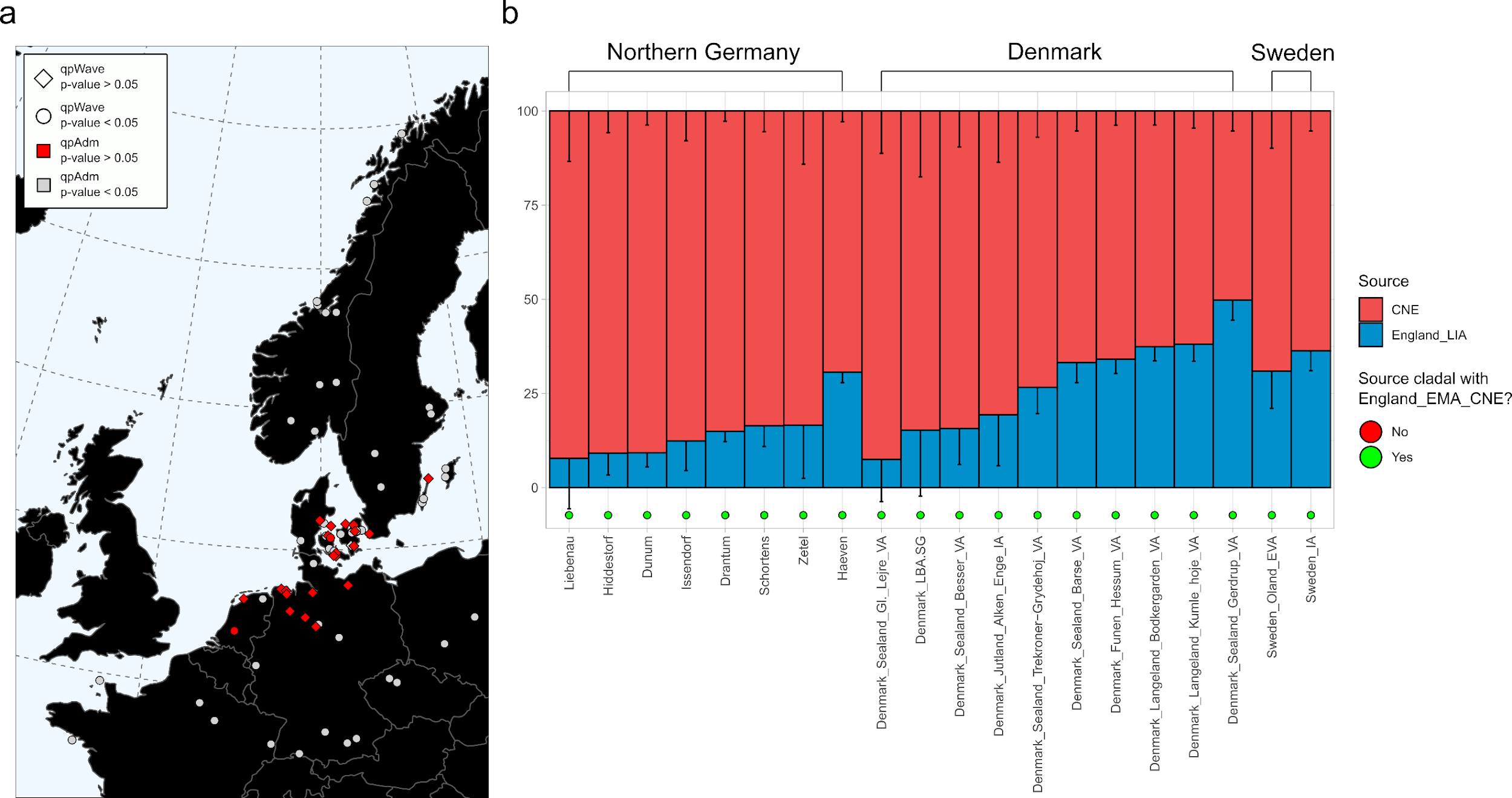


***Supplementary Figure 3.9. Continental populations that represent feasible proxies for CNE ancestry in early medieval English.*** *a) Distribution of populations (on the site level) that are i) genetically indistinguishable from England_EMA_CNE (n = 109) in qpWave (plotted as diamonds) and ii) feasible proxies in a two-way admixture model of England_EMA (n = 285) in qpAdm (plotted in red). b) Corresponding admixture proportions ordered according to country and CNE quantity. Samples sizes for continental populations are indicated in Supplementary Table 3.11. Error bars represent ± 1 standard error.*

The obtained results indicate a close relationship between *England_EMA_CNE* and the early medieval individuals from Lower Saxony as well as with several medieval Danish populations. We further tested a potential cladal relationship of *England_EMA_CNE* and *LowerSaxony_EMA* using a *F*_4_ statistics of the form *F*_4_(*England_EMA_CNE*, *LowerSaxony_EMA*; *Test*, YRI.SG), where *Test* iterates through 256 diverse ancient and modern Eurasian populations (Supp. Table 3.12). While 255 populations seem to be equally related to *England_EMA_CNE* and *LowerSaxony_EMA*, we find that *Latvia_BA* (*Z* = -3.944) is exhibiting excess affinity to *LowerSaxony_EMA*. This signal is still present after correcting for multiple testing using the Benjamini & Hochberg method for *p*-value adjustment[^176^](https://paperpile.com/c/hqnLqQ/g6Vrb) (*p* = 0.02051422). Noticeably, this is not the case for earlier or later populations from Latvia or contemporaneous Bronze Age populations from Lithuania and Estonia. Viking Age populations from Jutland, Zealand, Funen, and Langeland do not share significantly more alleles with *England_EMA_CNE* than with *LowerSaxony_EMA*. We therefore conclude that *LowerSaxony_EMA* is a close proxy to the continental northern European (CNE) ancestry that migrated to England during the Early Middle Ages. Based on this observation, we attempted to design a more direct approach of identifying CNE ancestry in early medieval England that is not resting on modern European references as sources. We carried out the analysis using *qpAdm*[*^171^*](https://paperpile.com/c/hqnLqQ/eK1xv) from ADMIXTOOLS[^174^](https://paperpile.com/c/hqnLqQ/5aYUV) and the basic set of 11 outgroups. We then modelled all early medieval English samples as a two-way admixture between a subset of England Iron Age and Roman period samples (*England_LIA_Roman*; *England_LIA* + *England_IA_ERoman.SG* + *England_IA_Roman.SG,* excluding one Middle Eastern outlier, *n* = 32)[^121,137^](https://paperpile.com/c/hqnLqQ/I6Awn+jnKBm) and a subset of early medieval, non-related Lower Saxony individuals (*LowerSaxony_EMA*, *n* = 39; Supp. Table 1). The obtained estimate of Lower Saxony ancestry (86.4% ± 2.2%) is only slightly higher than the CNE estimate obtained using present-day Danish and northern Germans as sources (86.4% vs. 76.3%), although the *p*-value of this *qpAdm* model is borderline to significance (*p* > 0.018). This may be related to heterogeneity within the early medieval English populations, especially with respect to affinity to the Scandinavian Peninsula (Supp. Note 6) and France Iron Age-related ancestry (Supp. Note 5).

#

# Supplementary Note 4 *Genetic structure of continental medieval samples*

## General population genetic affinities

We investigated population genetic structure across our samples in continental Europe using PCA. Specifically, we projected samples from each site onto our *Northwestern European PCA* (Supp. Fig. 4.1). In general, we observe that samples from all sites (with the exception of Alt-Inden) generally cluster together with those northern German, Danish, or northern Dutch groups that are close to their respective present-day regions of origin. However, in the sites of Groningen, Schleswig, Anderten, and especially Alt-Inden, we observed a major north-to-south cline of genetic variation. The spread of the respective genomes comprises the genetic variation of present-day southern Dutch, Belgians, Germans, and French.

To test if this shift was reflected in allele frequency correlations, we computed for each medieval continental genome *F*_4_ statistics of the form *F*_4_(YRI, *TestA*; CNE, *TestB*), where *TestB* iterates through six European populations, and *TestA* iterates through our ancient continental sample (Supp. Fig. 5.2, Supp. Table 4.1). Indeed, we observe that the sites of Groningen, Schleswig, Anderten, and Alt-Inden exhibit considerable variation in their samples’ affinities to present-day southern Europeans like Italians or Spanish. On the other hand, genetic stratification based on the affinity to the present-day Polish and Scandinavian Peninsula populations was especially visible in samples from Copenhagen, Schleswig, and Viking Age Swedes and Norwegians.
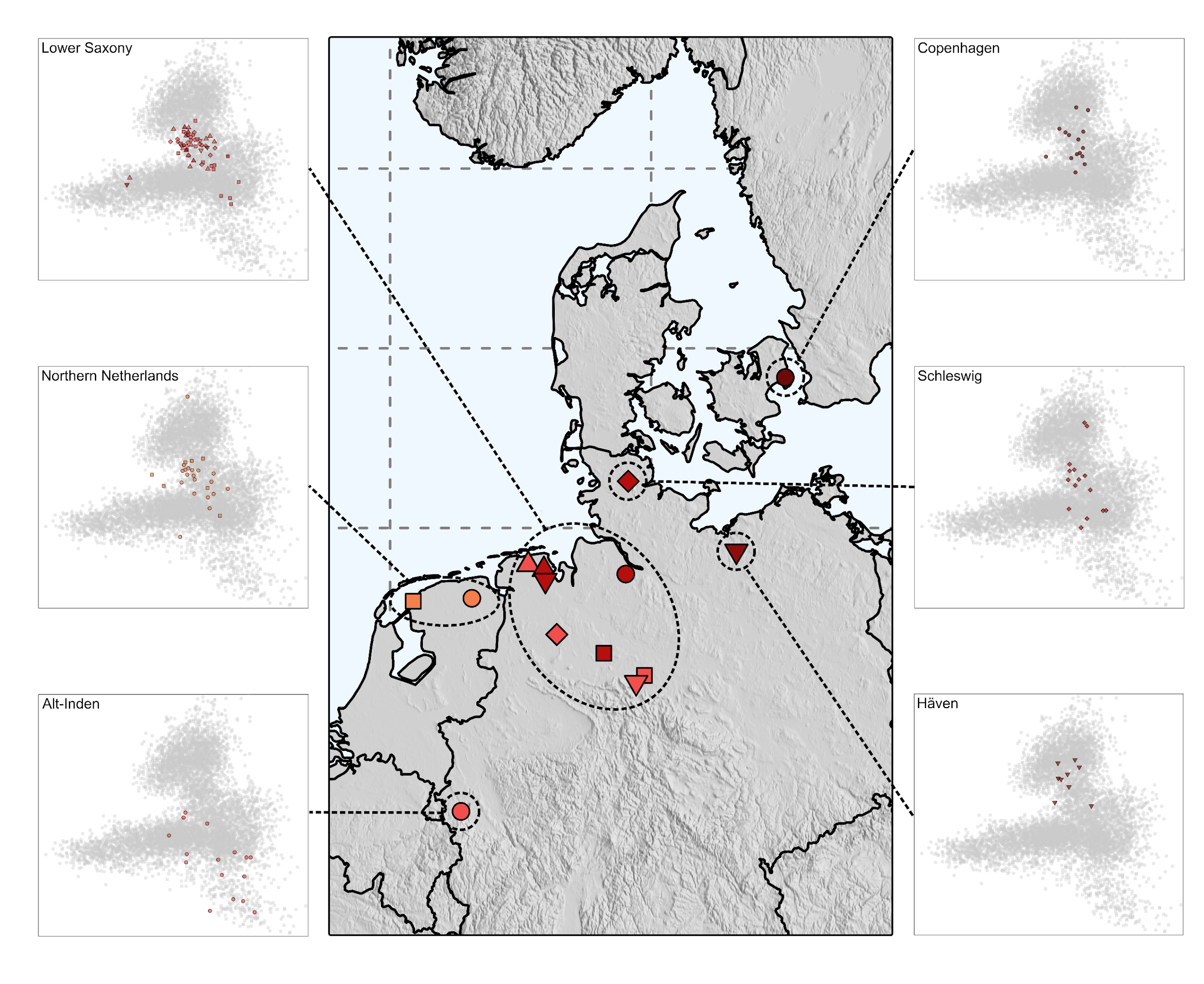


***Supplementary Figure 4.1, Geographic and genetic structure of 164 analysed continental individuals.*** *Shown are the locations of 15 newly reported sites. Per region, ancient individuals were projected onto principal components defined by 7,413 northwestern Europeans.*


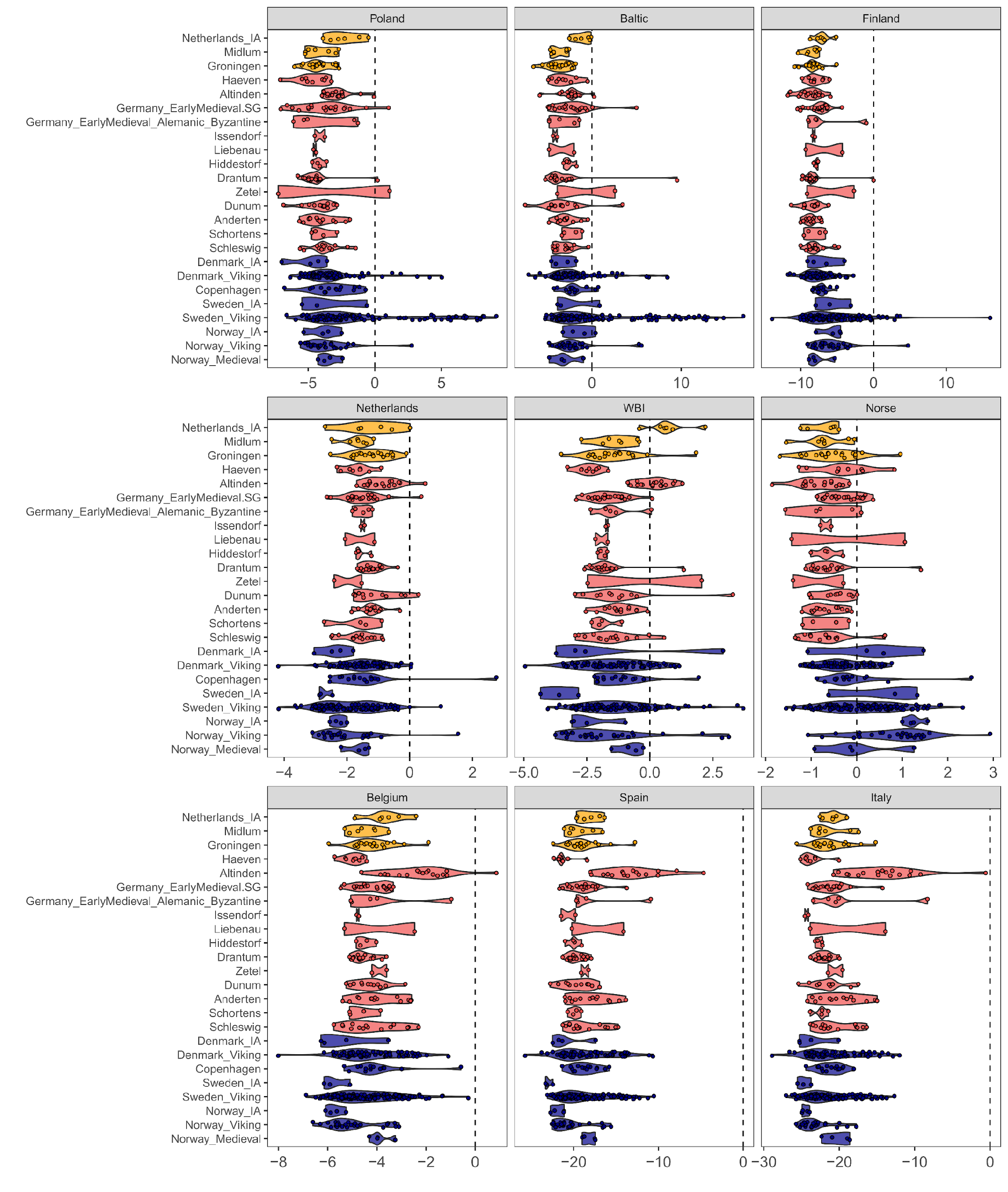


***Supplementary Figure 4.2, Contrasting allele-sharing between the CNE metapopulation of present-day Denmark & northern Germany and other European populations.*** *Violin plots showing distributions of the f-statistic F_4_(YRI, TestA; CNE, TestB) for ancient northwestern European individuals. TestB iterates through nine present-day European populations.*

## Identifying outliers

To investigate the presence of genetically more northern and north-eastern European outliers, we computed a third PCA, the *Northeastern European PCA*, which also includes Uralic, Baltic, and Slavic populations from north-eastern, eastern, and central Europe (Supp. Fig. 4.3). In this PCA the clustering of the present-day samples strongly resembles their geographical distribution. Topological features like the Scandinavian Peninsula and the Baltic Sea coastline are distinctly marked. While we find no notable outliers in sites from Lower Saxony, several individuals in Groningen, Schleswig, and Copenhagen were shifted in the direction of Norwegians and Swedes or Balto-Slavic populations. For those outliers, namely GRO021, KPN002, KPN014, SWG005, and SWG009, we calculated *F*_4_ statistics of the form *F*_4_(YRI, *TestA*; Denmark, *TestB*), where *TestB* iterates through 32 present-day European populations (Supp. Table 4.2). The individuals GRO021, KPN002, and SWG004 share more alleles with populations from the Scandinavian Peninsula than with Danish. On the other hand, KPN014 and SWG014 exhibit less pronounced affinities, sharing most alleles with modern Swedish and Baltic populations, especially Latvians, which indicates mixed ancestry for those two samples. Individual testing of allele sharing with present-day populations also revealed the presence of individuals with excess affinity to British and Irish populations. Those individuals are GRO006, DUN003, KPN010, and ZET002.


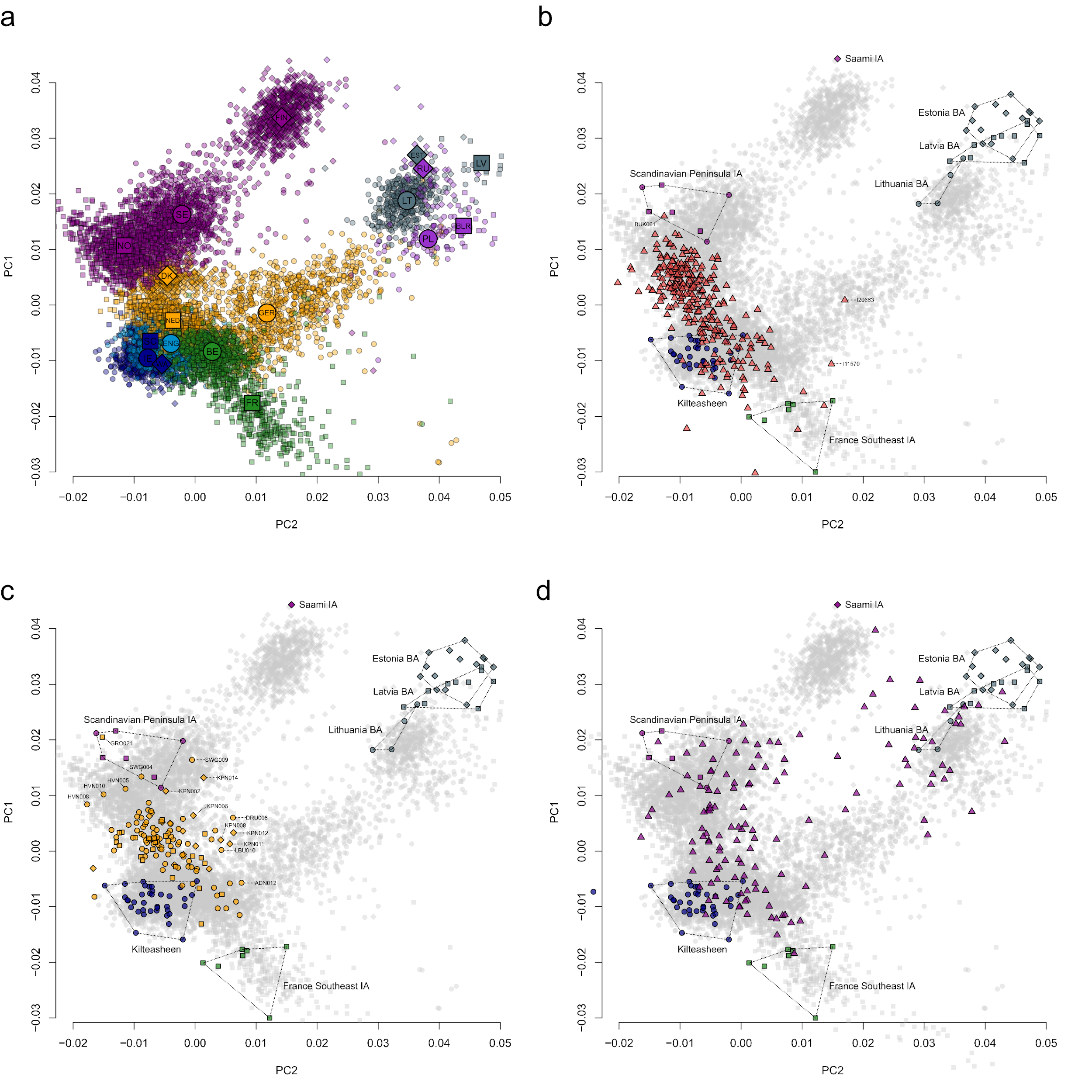


***Supplementary Figure 4.3, Population structure related to northeastern European ancestry.*** *a) Principal Components Analysis of present-day genomes from northwestern Europe. IE = Northern Ireland & Ireland, WA = Wales, SC = Scotland, ENG = England, NL = Netherlands, GER = Northern Germany, DK = Denmark, NO = Norway, SE = Sweden, FIN = Finland, BE = Belgium, FR = France, LIT = Lithuania, LV = Latvia, EST = Estonia, POL = Poland, BLR = Belarus, RU = Russia. b) Genetic structure of published and novel ancient England_EMA individuals in this study, projected onto a). c) Same for novel medieval Dutch, German, and Danish samples. d) Same for published Viking Age individuals from Sweden.*

## Estimating admixture

We next analysed our ancient continental samples using again the supervised model-based clustering implemented in ADMIXTURE[^172^](https://paperpile.com/c/hqnLqQ/TNMqU) against a Europe-wide panel of 7 modern reference population (*K* = 7) (Supp. Fig. 4.4, Supp. Table 4.3). This panel consists of the CNE (Denmark, northern Germany; *n* = 407), WBI (Ireland, Northern Ireland Scotland, Wales; *n* = 667), NOR (Norway, Sweden; *n* = 1910), FIN (Finland, *n* = 780), POL (Poland, *n* = 96), IBS (Spain, *n* = 211), and IT (Italy, *n* = 776). As expected, mean CNE ancestry represents the largest ancestry component in our North Sea sample (Supp. Table 4.4). It is maximised in Hiddestorf (99.6% ± 0.3%), Issendorf (96.7% ± 3.3%), Schortens (94.6% ± 5.4%), Drantum (90.8% ± 6.1%), and Dunum (89.8% ± 6.8%). Congruent with PCA positioning and *F*_4_ statistics, we detect the largest average Italian-like ancestry components in Alt-Inden (25.9% ± 7.2%) and Anderten (6.8% ± 3.2%), the southernmost sites in our analysis. In Alt-Inden (the most southwestern site), we also measure the highest average IBS-like ancestry contribution (24.4% ± 6.4%). The largest mean Polish ancestry component was found in Copenhagen (8.8% ± 2.5%). The amount of Polish ancestry is medieval Copenhagen was therefore already highly similar to the one measured in present-day Danes from Zealand (6.28%)[^177^](https://paperpile.com/c/hqnLqQ/4dRfU). The Copenhagen individuals, together with samples from Schleswig and Häven, also exhibit on average the highest ancestry contribution from the Scandinavian Peninsula (13.6% ± 3.8% for Copenhagen, 14.5% ± 7.2% for Häven, and 11.3% ± 6.7% for Schleswig), which is expected based on their geographic position between continental Europe and the Scandinavian Peninsula (where this ancestry in maximised in Iron Age and Viking Age individuals, eg. 97.5% in *Norway_IA* or 75.8% in *Sweden_IA*). Western British and Irish ancestry was present in several sites, scoring highest in Zetel (13.8%), Midlum (9.2% ± 5%), and Alt-Inden (7% ± 2.5%). These sites are located in the west of our sampling range, closest to the British-Irish Isles. Even higher estimates were measured for example in Iron Age samples from the Netherlands (35.9%) or from northern France (20.3%). Consistently with the *F*_4_ statistics, western and northern outlier individuals that we have identified exhibited additional ancestry from Britain and Ireland or from Scandinavia and the Baltics.


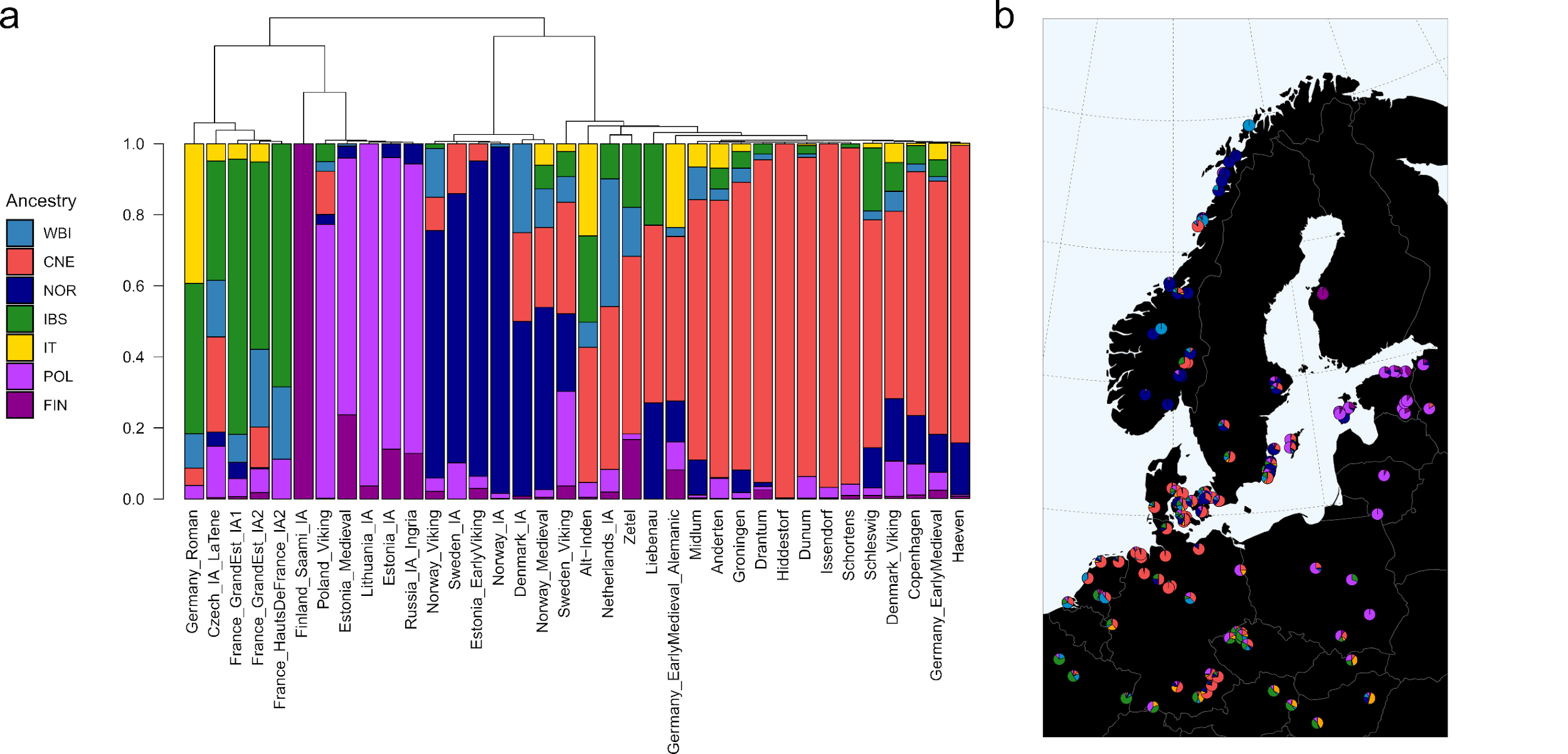


***Supplementary Figure 4.4, Supervised Admixture analysis across continental medieval northwestern Europe.*** *Shown is a supervised ADMIXTURE analysis, modelling Iron Age and medieval continental individuals as a mixture of CNE (red, n = 405), NOR (dark blue, n = 1910), Finland (FIN) (violet, n = 780), Poland (POL) (dark magenta, n = 96), WBI (light blue, n = 667), Spain (IBS) (forestgreen, n = 536), and Italy (IT) (yellow, n = 776). a) Averaged proportions per population are shown as barplot. b) Averaged proportions per population are shown as pie charts.*

Since we identified several outlier individuals within sites in Copenhagen, Groningen, Alt-Inden, and Anderten, we investigated the relationship between populations on a site-specific level. Using *qpWave* we tested, for each site, whether it was consistent with being a clade with all other studied sites (Supp. Fig. 4.5). We used again our basic set of 11 Outgroups (YRI.SG, Poland, Finland, Sweden, Denmark, Ireland, Wales, Italy, Spain, Belgium, Netherlands) and set the option allsnps: YES. Only Schleswig, Anderten, Alt-Inden, and Copenhagen were uniformly not consistent with being a clade with the rest of the Lower Saxony populations (Supp. Table 4.5). As described above, PCA, *f*-statistics, and ADMIXTURE suggest that this was due to those populations including individuals with significantly higher western, southern, or eastern European admixture than the rest of the continental individuals. Thus, we tried to model i) Alt-Inden, Anderten, and Schleswig as a mixture of *LowerSaxony_EMA* + *southern European Test*, and ii) Copenhagen as mixture of *LowerSaxony_EMA* + *northeastern European Test*.


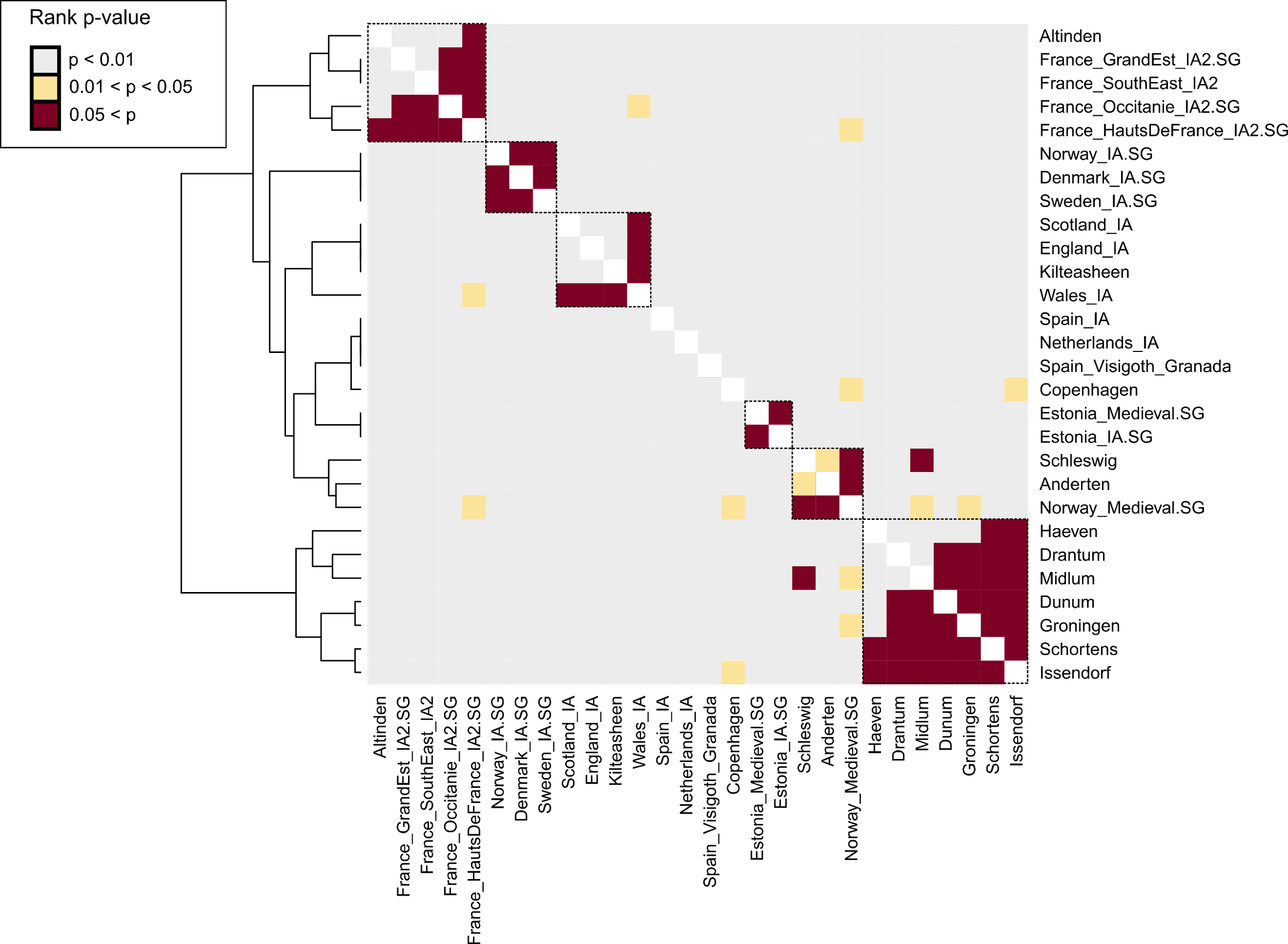


***Supplementary Figure 4.5, qpWave analysis.*** *Pairwise testing for genetic continuity and homogeneity between continental northern European medieval sites. Coloured squares depict whether a particular target group (row) can be modelled using a single source group (column). Rank p-values higher than 0.05 (corresponding to a single source group) were obtained using qpWave with a static set of 11 present-day outgroups.*

We carried out the analysis using *qpAdm* from ADMIXTOOLS^60^ and the basic set of 11 Outgroups. To investigate i) southern European ancestry in Alt-Inden, Anderten, and Schleswig we added the following populations to the 11 Outgroups:

- Greece
- Serbia
- Morocco

To investigate ii) north-eastern European ancestry in Copenhagen we added the following populations to the 11 Outgroups:

- CHB.SG
- Estonia
- Belarus

Regarding the southern European ancestry in medieval Germany, many populations can be used to explain the mixture observed in Alt-Inden, Anderten, and Schleswig. Those possible sources include Bronze, Iron Age and medieval populations from the Iberian Peninsula, Italy, southern Germany, the northern Balkans, as well as France (Supp. Table 4.6). For Alt-Inden, we see that all ancestry could be derived from French Iron Age sources without any (GrandEst & HautsDeFrance) or considerable (Occitanie & SouthEast) contribution from *LowerSaxony_EMA* (Supp. Table 4.6)*.* This similarity between the Merovingian site Alt-Inden and the Iron Age French is the first insight into the population structure of the Frankish Kingdom and points at genetic continuity and homogeneity across France and western Germany from the Iron Age until the Early Middle Ages.

Regarding the north-eastern European ancestry in Copenhagen, we find that fitting sources are Lithuanian Late Bronze Age and Iron Age, and Polish medieval populations. The estimated contributions for all models with a rank *p*-value larger than 0.01 range between 7.6% and 14.7%, which is close to our averaged estimate obtained using ADMIXTURE. The observation of notable proportions of eastern European ancestry in Zealand is consistent with previous studies of modern and ancient DNA, suggesting that the medieval and present-day Danish population was and is to some extent structured by the proportion of eastern European ancestry[^120,177^](https://paperpile.com/c/hqnLqQ/spmY+4dRfU).

#

# Supplementary Note 5 *Continental west European ancestry in ancient and modern England*

## Modelling early medieval England

PCA implies that several sites, especially from southern England (namely Apple Down, Buckland, Eastry, and Rookery Hill) exhibit remarkable diversity in terms of their ancestry. Besides England Iron Age and early medieval Lower Saxony-like ancestries, we also find individuals that cluster with present-day southern and western Europeans, especially with Belgians and French. This area of the PCA space is also occupied by Iron Age French and early medieval individuals from Alt-Inden (Extended Data Fig. 7). We therefore suggest that early medieval admixture patterns were heterogeneous across Britain, with ancestry from Lower Saxony being dominant in central and northern England (e.g. Oakington, Hatherdene, Lakenheath, and West Heslerton), while ancestry from western and southern Europe is observed in southern England, especially Sussex and Kent (Eastry, Rookery Hill, and Apple Down).

### Identifying outliers

To investigate this southern and western ancestry, we computed the widely-used West Eurasian PCA (introduced by Lazardis et al. 2014[^113^](https://paperpile.com/c/hqnLqQ/DEho3)) and projected our *England_EMA* samples onto the genetic variation of 59 West-Eurasian populations (Supp. Fig. 5.1). We note that with the exception of one individual, all *England_EMA* samples fall within the European diversity, especially of northwestern Europeans like English, Norwegians, and French. Only a very limited number of individuals falls outside the variation of those northwestern Europeans. This is consistent with our previous PCA results and highlights the issue that immigrant individuals either came from adjacent regions within Europe (especially from western Europe like Spain or France or southern Europe like Italy) or were only slightly admixed with non-European sources. PCA alone is not suitable to differentiate between those two patterns.


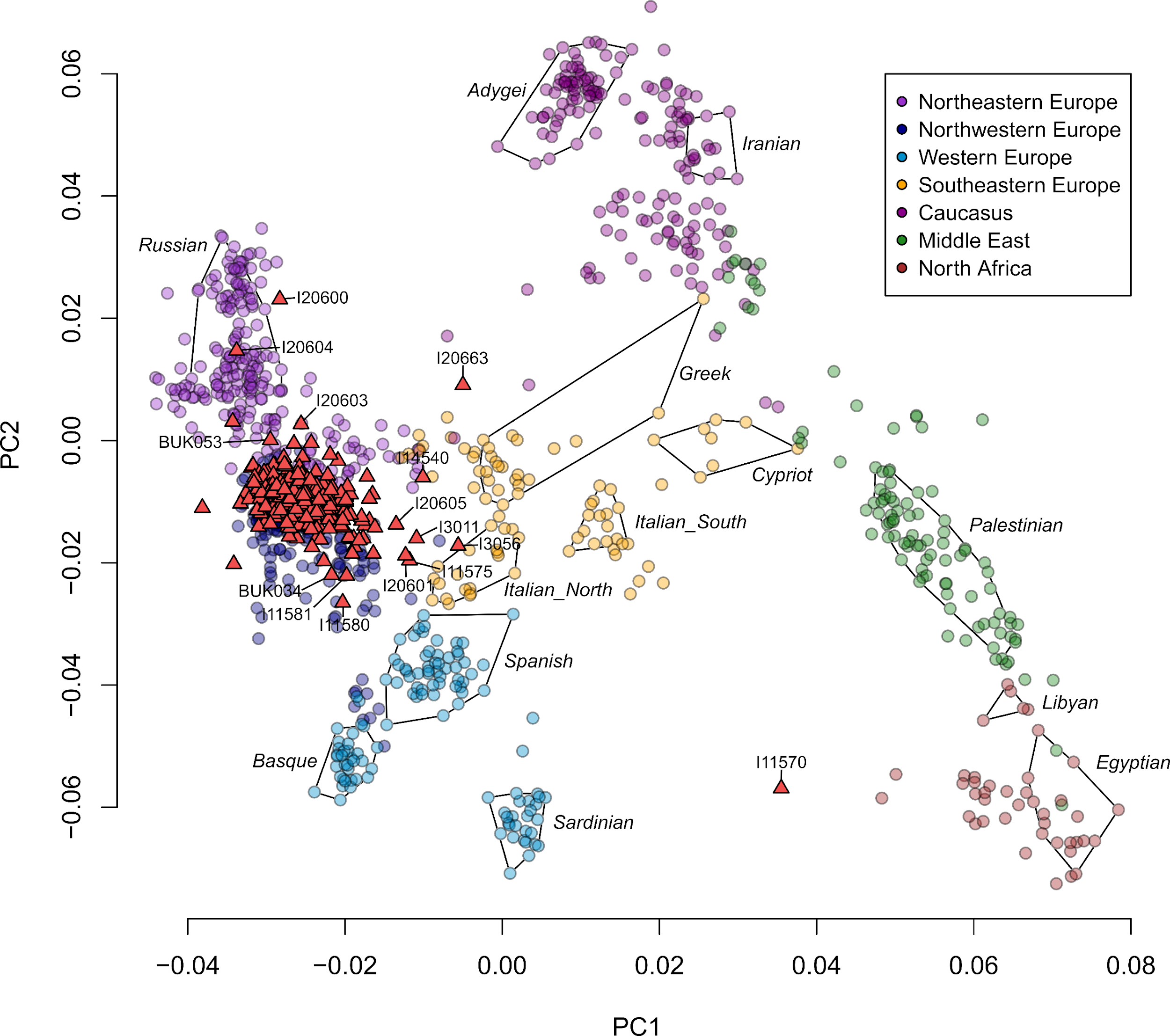


***Supplementary Figure 5.1, PCA of present-day genomes from western Eurasia.*** *Relevant present-day populations are highlighted. Ancient England_EMA individuals are projected onto the PCs as red up-facing triangles.*

We therefore used unsupervised ADMIXTURE to identify potential Asian, Middle Eastern, or African ancestry within our ancient samples. At *K* = 7, we identify two components mainly located in sub-Saharan Africa (a southern and western African), one north African/Middle Eastern component, one northern European component, one component maximised in populations from the Caucasus and South Asia, as well as two components located in eastern Asia, one maximised in northern Eurasians and one in East Asians (Supp. Fig. 5.2a). We identify several individuals in the *England_EMA* population that harbour elevated amounts of the North African/Middle Eastern and Caucasus/South Asia component, although this ancestry accounts only for 3.4% and 2.4% in the mean *England_EMA* population (Supp. Fig. 5.2c). Similar high amounts of those ancetries were also found in the Alt-Inden population (where they represent 19.1% and 4.6% of the ancestry on average) as well as in outlier individuals from Anderten, Schleswig, and Groningen (Supp. Fig. 5.2b). However, those proportions are very similar to the ones measured in present-day French, Spanish, Italians, and Bulgarians, and do therefore probably not reflect recent admixture with a north African or Middle Easterns source. Rather, those outlier came from regions in Europe, where North African/Middle Eastern ancestry is elevated, e.g. the Southwest and Southeast. The only exception is the outlier individual I11570 from Worth Matravers. This individual exhibits 22.4% ancestry from the western African component maximised in present-day Yoruba, Mende, and Esan. Similar amounts are measured in present-day north Africans like Algerians and Mozabites, however, those populations also carry high amounts of the North Africa/Middle Eastern component, which is minimal in I11570. It is therefore more likely that I11570 is the product of recent admixture between a northern European and a west African source. While small amounts of sub-Saharan African ancestry might be the result of low-coverage and/or contamination, there is no reason to assume this for I11570.


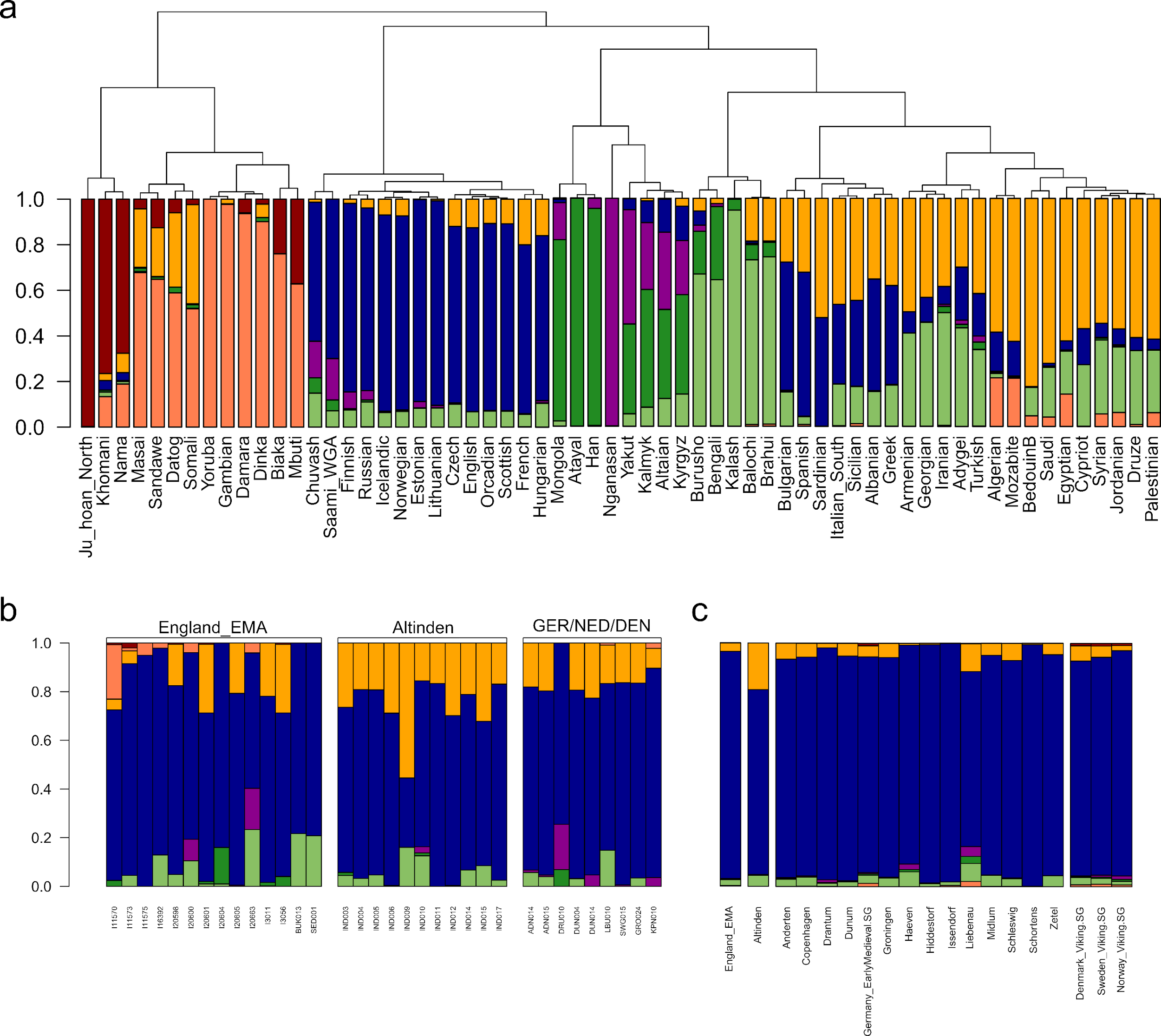


***Supplementary Figure 5.2 Unsupervised ADMIXTURE analysis.*** *Hierarchical clustering of Euclidean distances calculated on the mean ancestry component proportions from unsupervised ADMIXTURE analysis, using Ward's minimum variance method (ward.D2) presented as a phylogram. The bar chart below each branch shows the mean ancestry proportions from unsupervised ADMIXTURE for the respective population. a) For K = 7, only depicting present-day populations. b) For K = 7, highlighting outlier individuals. c) For K = 7, depicting population means of ancient populations.*

Based on the PCA and ADMIXTURE results, we tried to identify the geographic origin of the continental southwestern outliers. We grouped individuals together that deviated from the *England_IA*-*England_EMA* cline as *England_EMA_CWE* (CWE= Continental Western European). We then calculated pairwise *F*_3_ statistics as mentioned in Supp. Note 3 and visualised the results using MDS (Supp. Fig. 5.3a). The *England_EMA_CWE* population plots separate from the *England_MBA*, *England_LBA*, and *England_IA* populations, closer to continental populations like Anderten, Schleswig, Norway_medieval, as well as Altinden and Germany_EarlyMedieval_Alemannic on coordinate 2. A higher resolution was obtained using our *F*_4_-PCA approach from Supp. Note 3 (Supp. Fig. 5.3b). Here, the *England_EMA_CWE* population clusters distinctly from other English groups between the *Czech_LBA_Knoviz*, *Czech_IA_LaTene*, *France_GrandEst_IA2*, and *France_HautsDeFrance_IA2* populations due their elevated affinity to present-day French and Spanish.


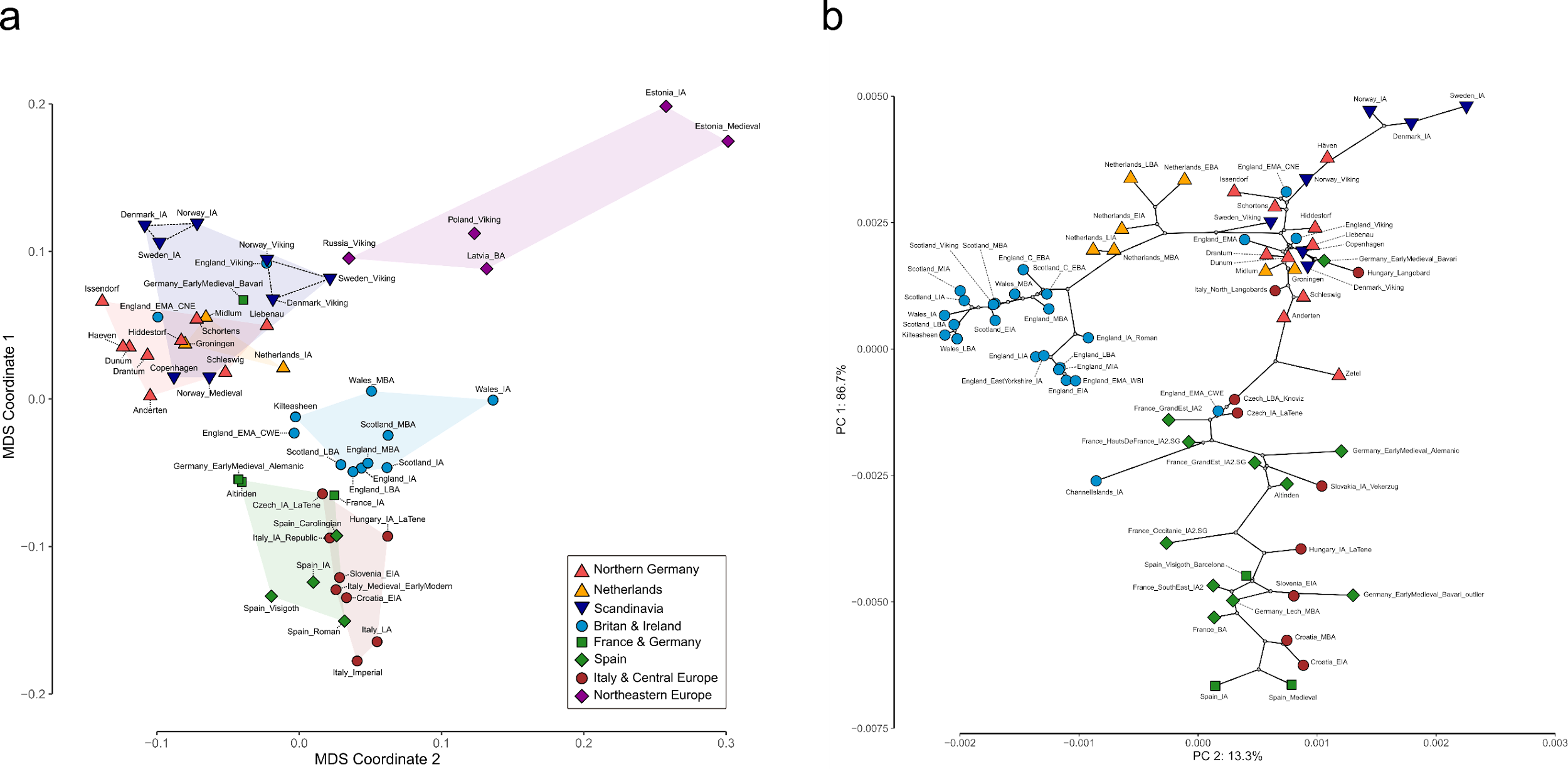


***Supplementary Figure 5.3, F_3_ and F_4_ statistics.*** *Genetic affinity between early medieval English individuals that deviate from the England_IA-England_EMA cline (England_EMA_CWE) and contemporary continental populations. a) Multidimensional Scaling Plot of pairwise F_3_ distances of the form F_3_(CHB, ancient population A, ancient population B). b) PCA of F_4_ statistics of the form F_4_(YRI, ancient population; TestA, TestB). TestA and TestB iterate through 15 present-day European populations (Methods). Additionally, a neighbour-joining tree of the same dataset was projected onto the two PCs.*

However, PCA positions imply that most outlier individuals are not unadmixed immigrants but already harbour varying amounts of *England_IA* or *LowerSaxony_EMA* ancestry. We therefore tested which continental populations qualify as source of ancestry for this *England_EMA_CWE* group in the three-way model *England_LIA_Roman* + *England_EMA_CNE* + *Test* using *qpAdm*[*^171^*](https://paperpile.com/c/hqnLqQ/eK1xv) from ADMIXTOOLS[^174^](https://paperpile.com/c/hqnLqQ/5aYUV) (v.4.1). We set the option allsnps: YES. For *Test*, we selected 163 prehistoric and historic populations from Europe, especially covering central, southern, southwestern and western European populations. While 37 Bronze, Iron, and Middle Age European populations produce fitting models, the majority of those are located in western and central Europe. Highest density of suitable proxy populations is found in France, followed by the Alps region, the northwestern Balkans, and the Iberian peninsula (Supp. Fig. 5.4, Supp. Table 5.1).


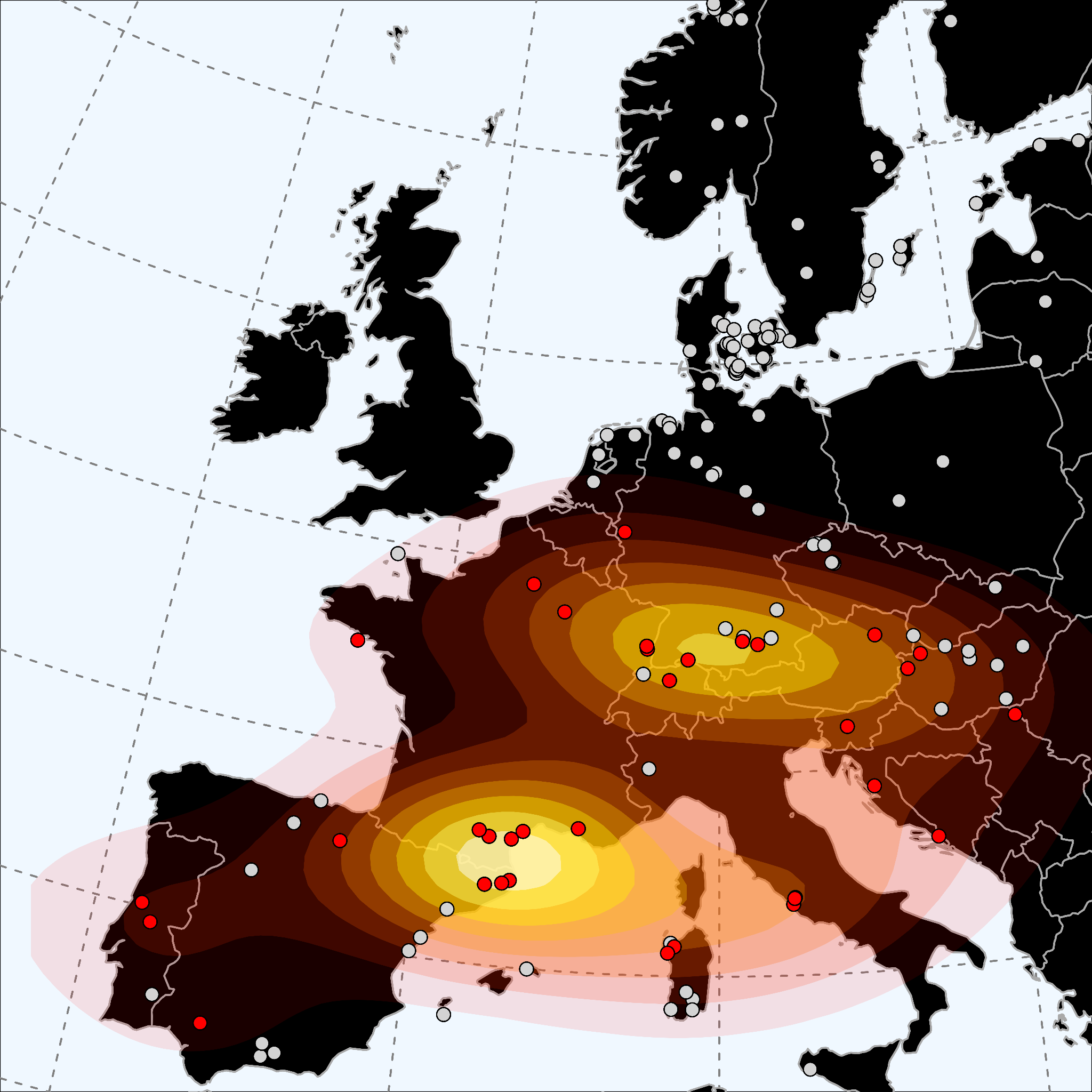


***Supplementary Figure 5.4, Potential sources of CWE ancestry in early medieval England.*** *Shown is a 2D kernel density estimation of continental populations that act as feasible proxies for a three-way qpAdm admixture model of England_EMA_CWE.*

### Modelling early medieval England

To assess the impact of immigration on the early medieval English population from western and southwestern European regions, we used a modification of the *qpAdm* model introduced in Supplementary Note 3 on a site-specific level. Initially, we calculated for each early medieval English site (*n* = 28) the proportions of *England_LIA* and *LowerSaxony_EMA*-related ancestry. As stated earlier, the estimates of “modern” (ADMIXTURE) and “ancient” (*qpAdm*) CNE ancestry for each site are highly congruent and correlated, indicating that both methods independently identify the same signal of admixture and accurately measure the respective ancestry proportions (Supp. Fig. 5.5, Supp. Table 5.3).


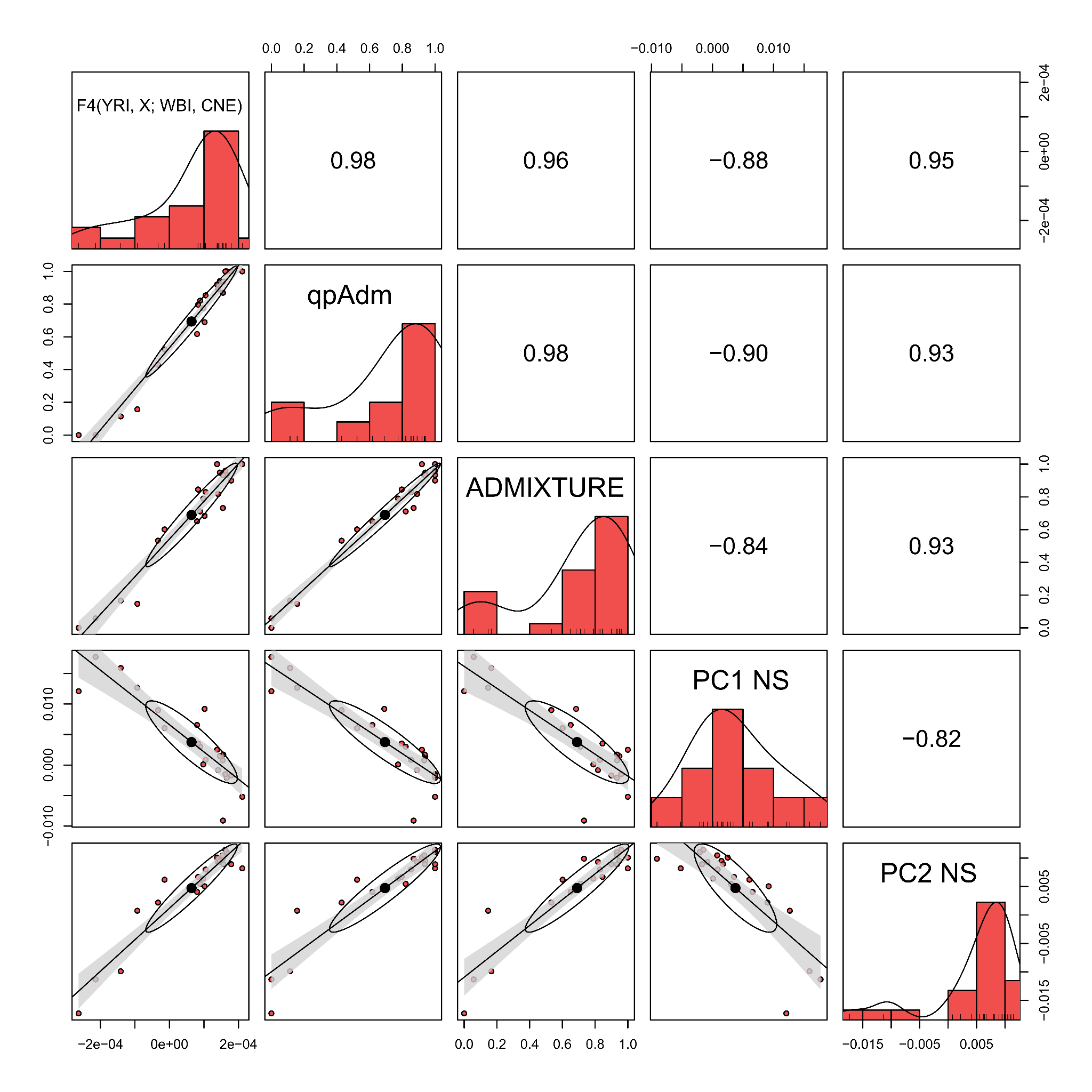
***Supplementary Figure 5.5, Validation of CNE estimates across different methods.*** *Correlogram illustrating pairwise correlations between different measurement of genetic affinity to CNE and WBI (mean supervised ADMIXTURE estimates at K = 2, F_4_ statistics of the form F_4_(YRI, X; WBI, CNE), mean PC1, and PC2 coordinates in our North Sea PCA) for 28 early medieval English sites. Pearson’s r for each pairwise correlation test are indicated in the upper triangle of the matrix. Error bands represent ± 2 standard errors.*

However, for the southern English sites of Apple Down, Eastry, and Rookery Hill we obtain either no fitting or only borderline-fitting *p*-values (*p*-values < 0.05) (Supp. Table 5.3). We therefore allowed for a third source approximating western or southwestern European ancestry. Based on *qpAdm* analysis presented above we suggest populations from France, the Alps region, or the Iberian peninsula as reasonable proxies for this kind of ancestry (Supp. Fig. 6.4). For the following *qpAdm* models, we decided to group published French Iron Age individuals together, called here *France_IA*, due to their temporal and geographical proximity. Most central and northern English sites do not profit from adding this third western European source; instead, three-way models for those populations are infeasible, producing negative *France_IA* admixture coefficients. In contrast, for Apple Down, Rookery Hill, Eastry, and Lincoln we obtain significantly better fits (*p*-values > 0.05) using a three-way model with *France_IA* ancestry included as a source (Supp. Table 5.4). In all these sites, *France_IA* ancestry comprises a substantial part of the ancestry (Supp. Table 5.4).

To check this observation of French-like ancestry, we then calculated admixture proportions for each medieval English individual and site in a supervised ADMIXTURE analysis using an extended three-way model with CNE, WBI, and CWE as the source populations. Here, CWE (“continental western European”) consists of present-day French and Belgians (*n* = 1074), who show the highest genetic affinity to *France_IA* across all modern Europeans in our dataset (*F*_ST_ _France_: 0.0037 ± 0.0007; *F*_ST Belgium_: 0.0041 ± 0.0007) and therefore most closely approximate the ancient admixture source. As indicated by PCA, supervised ADMIXTURE identifies sizable proportions of modern French/Belgian-like ancestry in our ancient samples, reaching as much as 100% in some individuals (Supp. Fig. 5.6a, Supp. Table 5.5). Calculating the average for each site, we find, congruently with our *qpAdm* approach, the highest proportions of French/Belgian-like ancestry in Lincoln (59.9%), Rookery Hill (43.3%), Apple Down (27.8%), Eastry (25.6%), and Dover Buckland (22.5%) (Supp. Fig. 5.6b). In the remaining sites, French/Belgian-like ancestry accounts for less than 10% of the total ancestry. In summary, additional western and/or southern European related ancestry appears to be the main cause for the remarkable genetic diversity observed especially in southern English early medieval sites (Supp. Fig. 5.7).


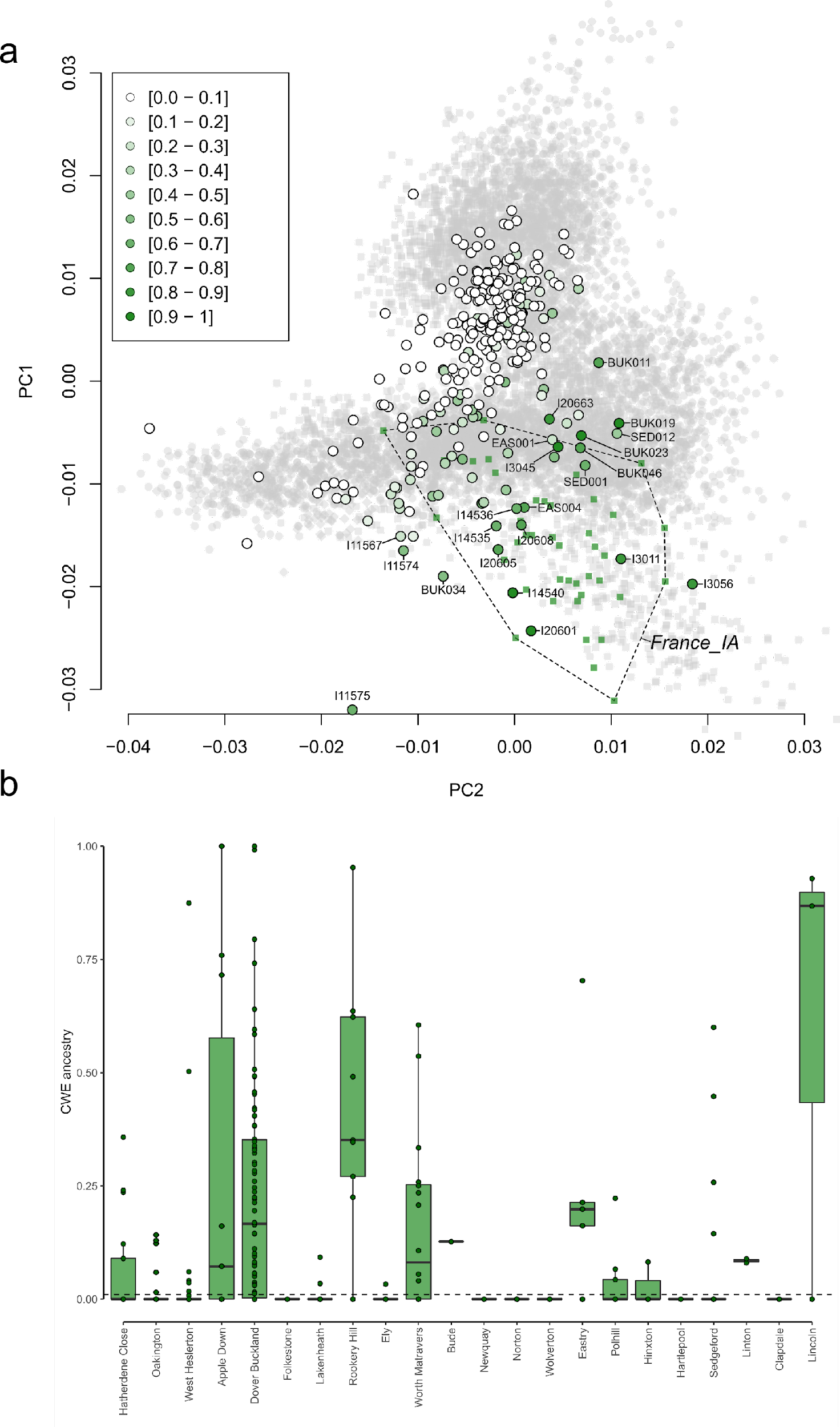


***Supplementary Figure 5.6 Distribution of continental western European (CWE) ancestry across early medieval England.*** *a) Distribution of continental western European (CWE) ancestry across early medieval English corresponding to PC1 and PC2 in our Northwestern European PCA as inferred using supervised ADMIXTURE. b) Shown are the estimates as boxplots per site. Bounds of the Box represent the 25^th^ and 75^th^ Percentile. The centre represents the median. Whiskers represent the smallest value greater than the 25^th^ Percentile minus 1.5 times the interquartile range and largest value less than the 75^th^ Percentile plus 1.5 times the interquartile range, respectively. Outliers present the minimum and maximum values in the data. Samples sizes for early medieval English sites are indicated in Supplementary Table 5.3.*


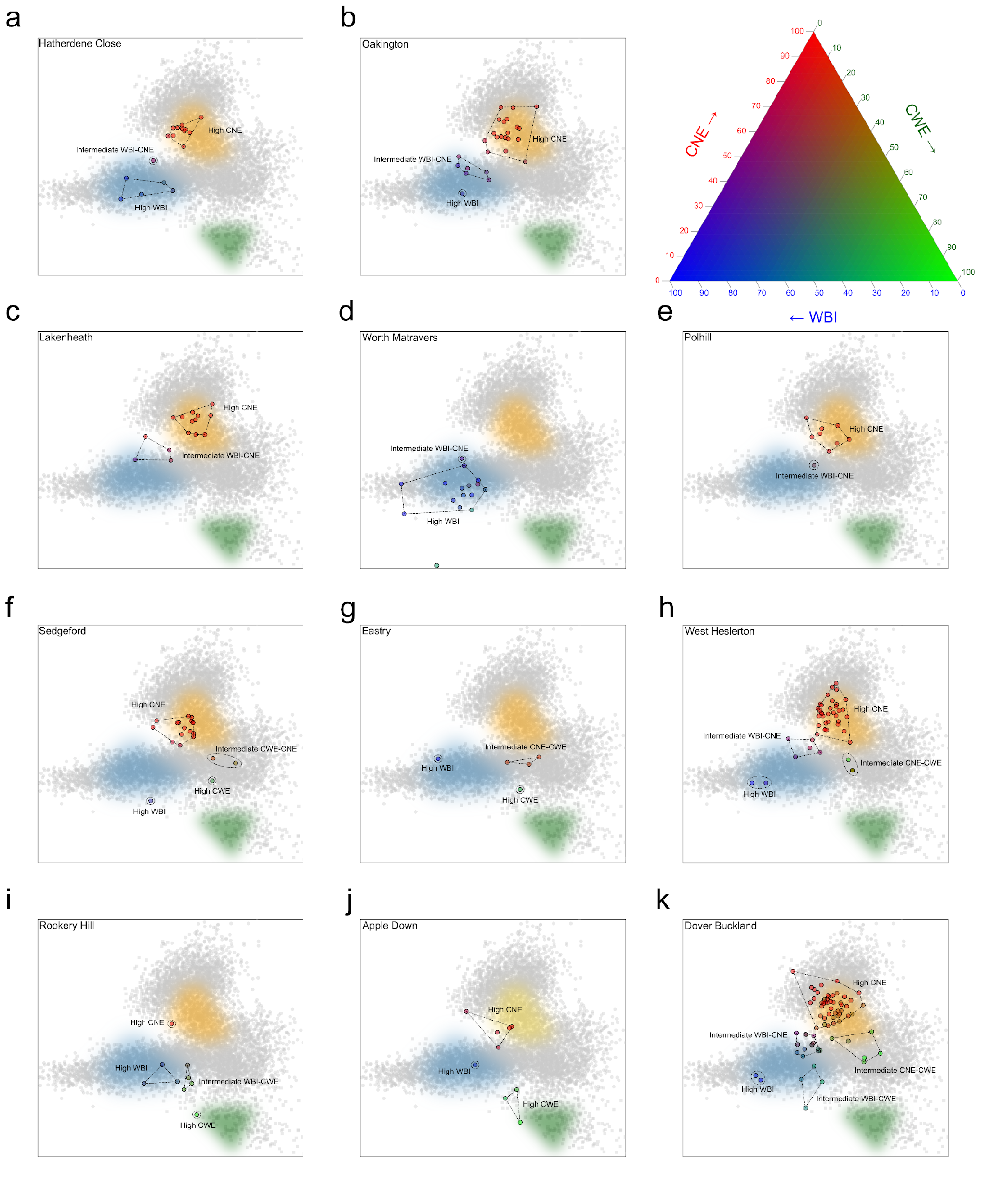

***Supplementary Figure 5.7 Variation of ancestry and admixture patterns across early medieval English sites.*** *For eight selected English sites, ancient individuals are projected onto the first two principal components of our Northwestern European PCA. The Individuals are coloured according to their WBI, CNE, and CWE ancestry proportions as inferred using supervised ADMIXTURE. For England_LIA, France_SouthEast_IA, and LowerSaxony_EMA the distribution across PC1 and PC2 are indicated. a) Hatherdene Close, b) Oakington, c) RAF Lakenheath, d) Worth Matravers, e) Polhill, f) Sedgeford, g) Eastry, h) West Heslerton, i) Rookery Hill, j) Apple Down, and k) Dover Buckland.*

## Modelling present-day England

Since our approach to identifying and measuring admixture worked well in an ancient DNA context, we went on to model present-day British and Irish populations. First, we tested if six Irish and British metapopulations (England (randomly downsampled to 100 individuals), Wales, Scotland, Orkney, the Republic of Ireland, and Northern Ireland) could be modelled as simple two-way admixture between *England_EMA_CNE* and one of twelve post-Neolithic British and Irish sources (*England_C_EBA*, *England_MBA*, *England_LBA*, *England_IA*; *Ireland_EBA*; *Scotland_C_EBA*, *Scotland_MBA*, *Scotland_LBA, Scotland_IA, Wales_MBA, Wales_LBA, Wales_IA*) (Supp. Fig. 5.8, Supp. Table 5.6). We find that Ireland and Northern Ireland can be both modelled successfully as such a two-way mixture involving either Welsh or Scottish Bronze Age sources, with a higher proportion of CNE ancestry in Northern Ireland. Higher amounts of CNE ancestry in Northern Ireland (13.5% ± 3.2% using *Scotland_LBA*) than in the Republic of Ireland (5.9% ± 3.8% using *Scotland_LBA*) may reflect the plantation of Ulster, the colonisation of Ulster by people from Britain, beginning in 1609, as suggested by previous studies[^56,178–180^](https://paperpile.com/c/hqnLqQ/eLLr1+2b2nD+0Jl9L+qyDmA). We further obtain fitting models for Scotland and Orkney, where we estimate a higher amount of CNE ancestry in Orcadians (29.5% ± 2.8% using *Scotland_LBA*) than in Scottish (20.5% ± 3.1% using *Scotland_LBA*), which is probably the result of intensive Viking and later Scandinavian colonisation (Orkney was a part of Norway from 875 to 1472)[^56,180^](https://paperpile.com/c/hqnLqQ/eLLr1+qyDmA). Wales can be well modelled using Welsh Bronze Age or Iron Age populations as proxy for local British ancestry, involving only a minor CNE contribution (15.7% ± 4.8%), which supports earlier assumptions that the Welsh population indeed most closely resembles the Iron Age population of Britain[^56,121^](https://paperpile.com/c/hqnLqQ/eLLr1+jnKBm). However, strikingly, none of the two-way models generates significant fits for England (all *p* < 0.01), which is congruent with previous analyses rejecting a simple two-way admixture in England[^56,120^](https://paperpile.com/c/hqnLqQ/eLLr1+spmY).


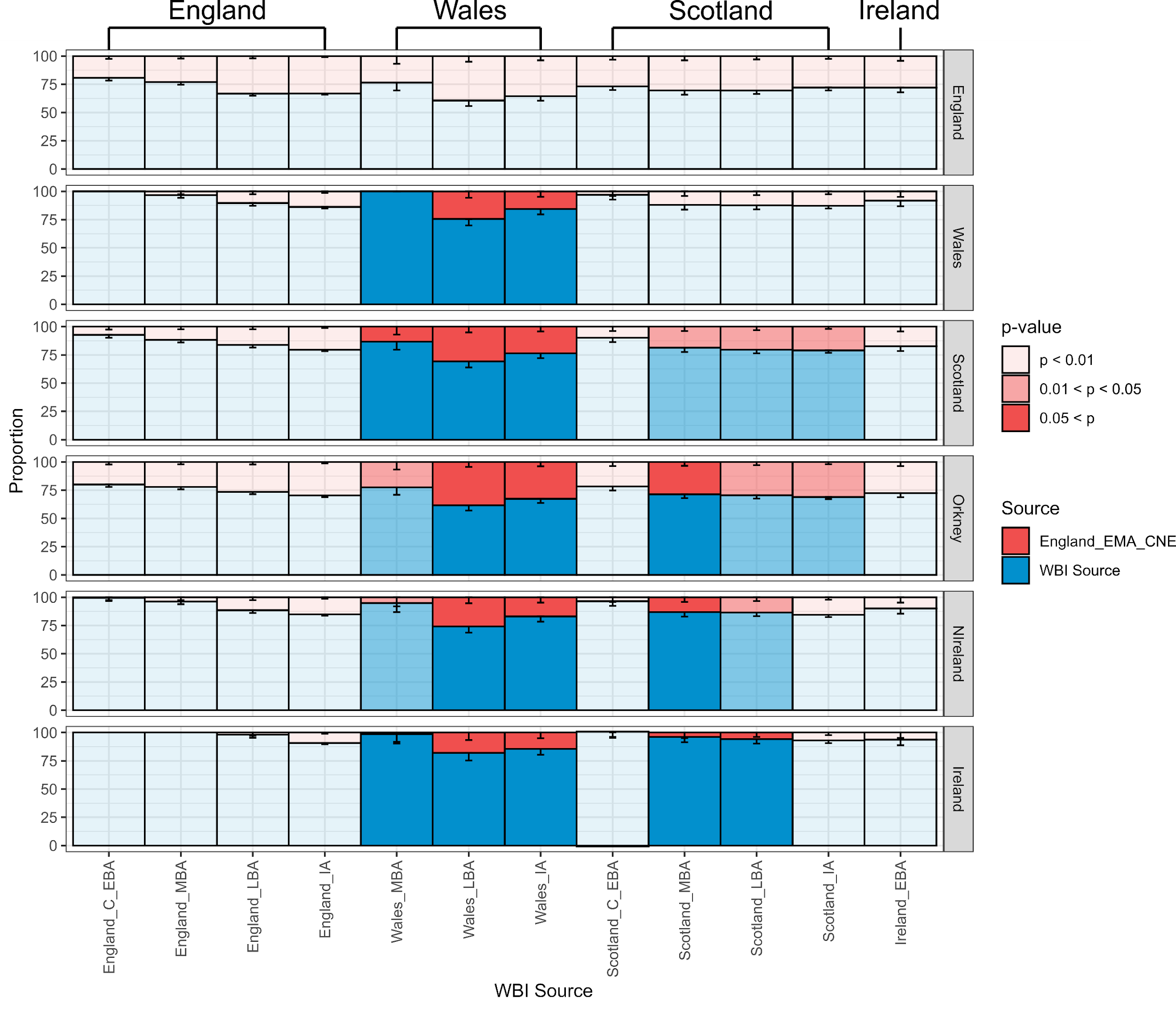


***Supplementary Figure 5.8, qpAdm analysis of present-day Britain and Ireland.*** *Ancestry proportions of present-day British and Irish populations (n = 6) inferred using qpAdm. Target groups were modelled using two distal sources representing CNE (England_EMA_CNE) and WBI (eight English, Scottish, and Irish Bronze and Iron Age Test populations) ancestry. Opacity is determined by the corresponding qpAdm p-value. Samples sizes are indicated in Supplementary Table 5.6. Error bars represent ± 1 standard error.*

We therefore tested if the addition of a third source may improve model fit as observed in the ancient population modelling. Due to the temporal and geographic proximity, we used England Late Iron and Roman Age (*England_LIA* + *England_IA_Roman,* excluding one Middle Eastern outlier, *n* = 27)[^121,137^](https://paperpile.com/c/hqnLqQ/I6Awn+jnKBm) as proxy for local English ancestry (*England_LIA*). As source of CNE ancestry we used *England_EMA_CNE*: A subset of 109 unrelated Early Anglo-Saxon period individuals that exhibit not more than 5% WBI ancestry in our supervised ADMIXTURE approach. Data for these individuals was produced using both 1240k capture and SG sequencing.

As a potential third source, we selected 163 prehistoric and historic populations from Europe, especially covering central, southern, southwestern and western European populations (Supp. Fig. 5.9). Indeed, the inclusion of a third source from continental Europe resulted in 32 cases in fitting models (Supp. Table 5.2, Supp. Table 5.7). As observed in our ancient sample, the best fitting populations across all models are from central, western, and southwestern Europe, especially France, Iberia, and the alpine countries, which supports our observation that England underwent additional introgression of southern and/or western European ancestry, consistent with previous analyses of modern and ancient mtDNA diversity[^181^](https://paperpile.com/c/hqnLqQ/vtUXz). The extent of this introgression varies between 8% and 50% depending on the source, with a mean of 22% +/- 9% SD (Supp. Fig 6.10). We highlight that 10 out of 32 non-rejected source populations (31%) are located in France, and twelve (38%) on the Iberian Peninsula, strongly suggesting a southwestern European origin of this ancestry.


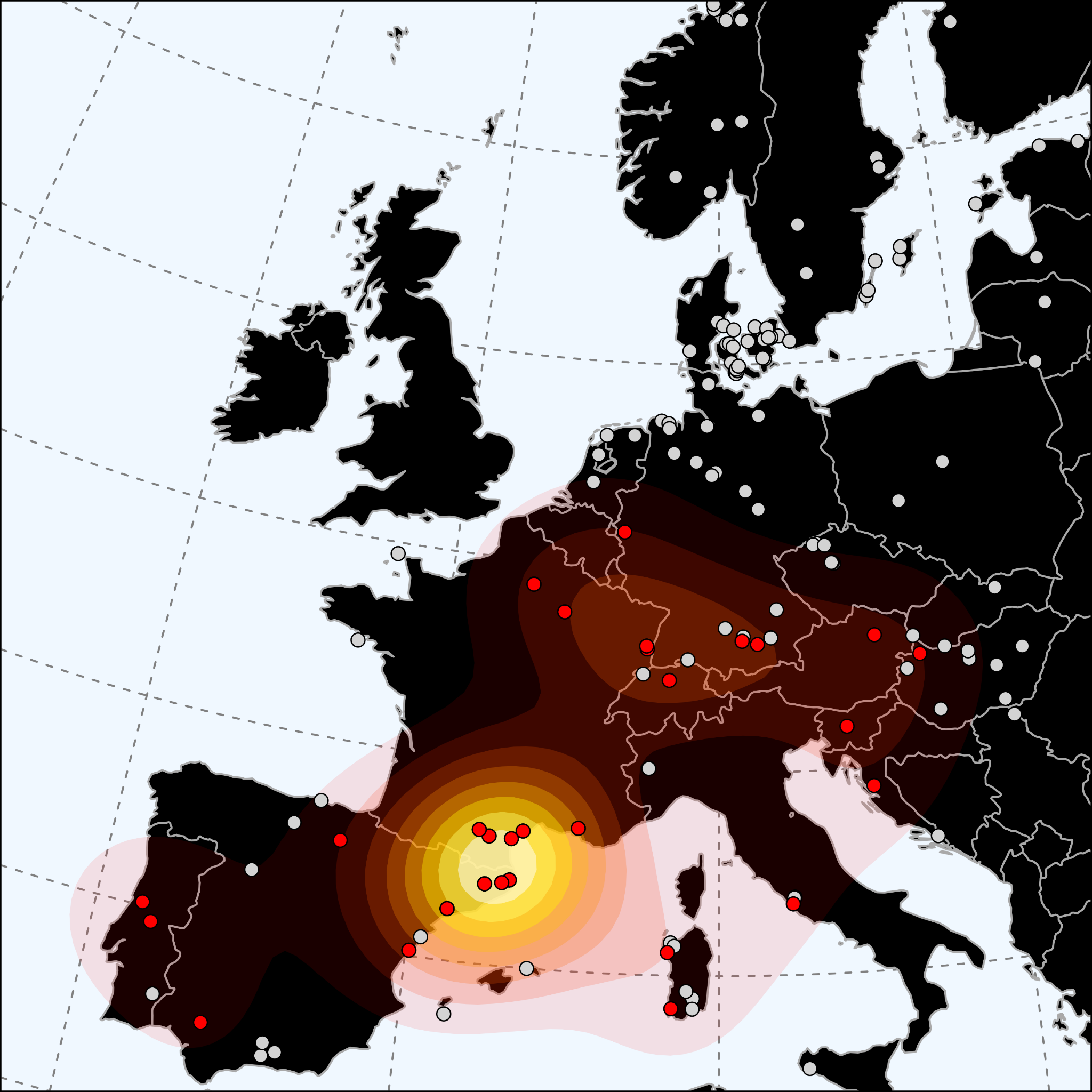


***Supplementary Figure 5.9, Potential sources of CWE ancestry in present-day England.*** *Shown is a 2D kernel density estimation of continental populations that act as feasible proxies for a three-way qpAdm admixture model of present-day England.*


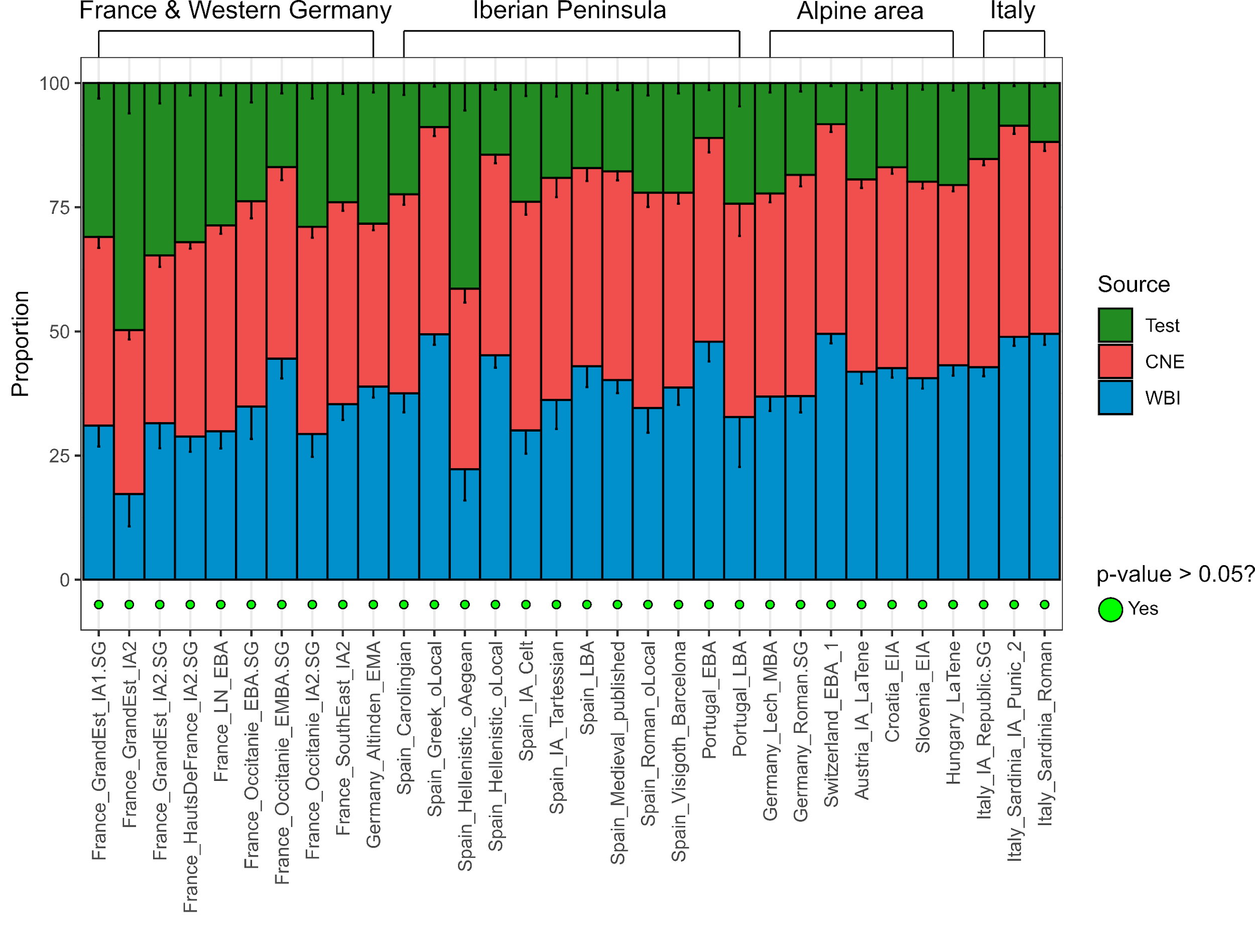


***Supplementary Figure 5.10, Sources of CWE ancestry in present-day England.*** *Admixture proportions in present-day England (n = 1678) corresponding to the feasible models presented in Supp. Fig 6.5****.*** *Bars are ordered according to the country of the southwestern source. Samples sizes for continental populations are indicated in Supplementary Table 5.7. Error bars represent ± 1 standard error.*

### Identification of southern European-reated ancestry in England

We then proceeded to investigate regional admixture in contemporary England. First, we considered the possibility that the presence of continental western European ancestry in present-day England was due to the resurgence of ancient English ancestry with higher proportions of western/southwestern European ancestry than *England_LIA*. To assess whether continental western European ancestry entered to a large extent after the Iron Age and Roman period or was already present during earlier times, we modelled 23 sampling regions from the PoBI project in *qpAdm* using either a two-way (*pre-EMA England* + *CNE*) or three-way model (*pre-EMA England* + *CNE* + *France_IA*) (Supp. Fig. 5.11, Supp. Table 5.8). Using Neolithic, Bronze, or Iron Age English populations as proxy for local English ancestry, we do not find any two-way model that provides a fit with *p* > 0.01 (Supp. Fig. 5.11). Only using Roman period individuals, we obtain for Cumbria a model with a *p*-value > 0.01. In contrast, applying a three-way model with *France_IA* as an additional third source we were able to model all 23 English regions successfully (*p* > 0.05) using *England_LIA* or *England_IA_Roman* individuals (Supp. Fig. 5.11). Noteworthy, the number of fitting models reduces with the age of the chosen English populations. Using Iron Age/Roman period individuals as a proxy, we were able to obtain 83 fitting (*p* > 0.05) three-way models out of a total of 115. Bronze Age individuals only produced fitting models in 38 out of 92 cases. Finally, using Neolithic individuals as proxy, neither one of the 23 two-way nor one of the 23 three-way models produced a significant fit. This indicates that the prevalence of western European ancestry observed in present-day English cannot be explained by a resurgence of ancient ancestry carrying more western European ancestry than the English population at the transition from the Iron to the Early Middle Ages, since none of the preceding ancient English populations harboured sufficient proportions of this specific southern European ancestry. We further explored this hypothesis by assessing the effect of geographical stratification. To do this, we tested whether ten present-day PoBI sampling regions for which Iron Age data was available, could be modelled as a simple two-way admixture between the respective local Iron Age population and the *England_EMA_CNE* population. For none of the ten tested populations this was the case (Supp.Fig. 5.12, Supp. Table 5.9). We furthermore highlight that not only do the preceding English populations exhibit insufficient proportions of southern European ancestry, but the southern European ancestry found in present-day English seemingly differs from the one introduced during the Neolithic. In a three-way *qpAdm* setup, modelling present-day England as mixture between *England_IA*, *England_EMA_CNE*, and a British-Irish Neolithic source (*England_N*, *Wales_N*, *Scotland_N*, *Ireland_MN.SG*), each of the four models gets strongly rejected (p < 7.86e-20, p < 2.34e-6, p < 3.95e-16, and 1.63e-19), disagreeing with the hypothesis that southern European ancestry resurged from refugia within the Britain or Ireland. Rather this shows that modern English harbour a distinctive type of southern European ancestry not present in Early Neolithic British farmers.


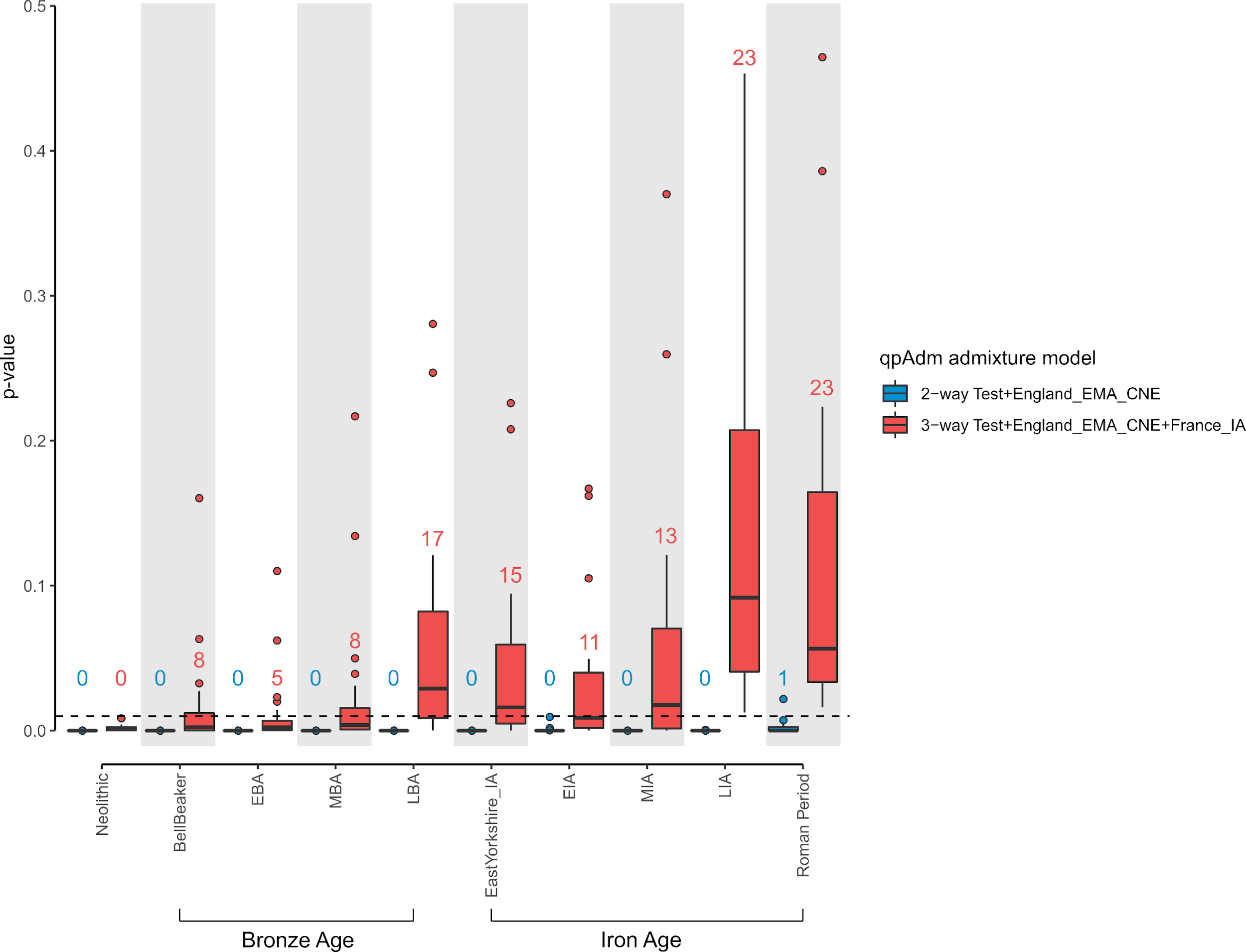


***Supplementary Figure 5.11, Modelling present-day English regions (n = 23) as two-way and three-way admixtures.*** *The source for local English ancestry iterates through 10 ancient English populations. The distributions of p-values obtained from qpAdm are visualised as boxplots. Bounds of the Box represent the 25^th^ and 75^th^ Percentile. The centre represents the median. Whiskers represent the smallest value greater than the 25^th^ Percentile minus 1.5 times the interquartile range and largest value less than the 75^th^ Percentile plus 1.5 times the interquartile range, respectively. Outliers present the minimum and maximum values in the data. Samples sizes are indicated in Supplementary Table 5.8.*


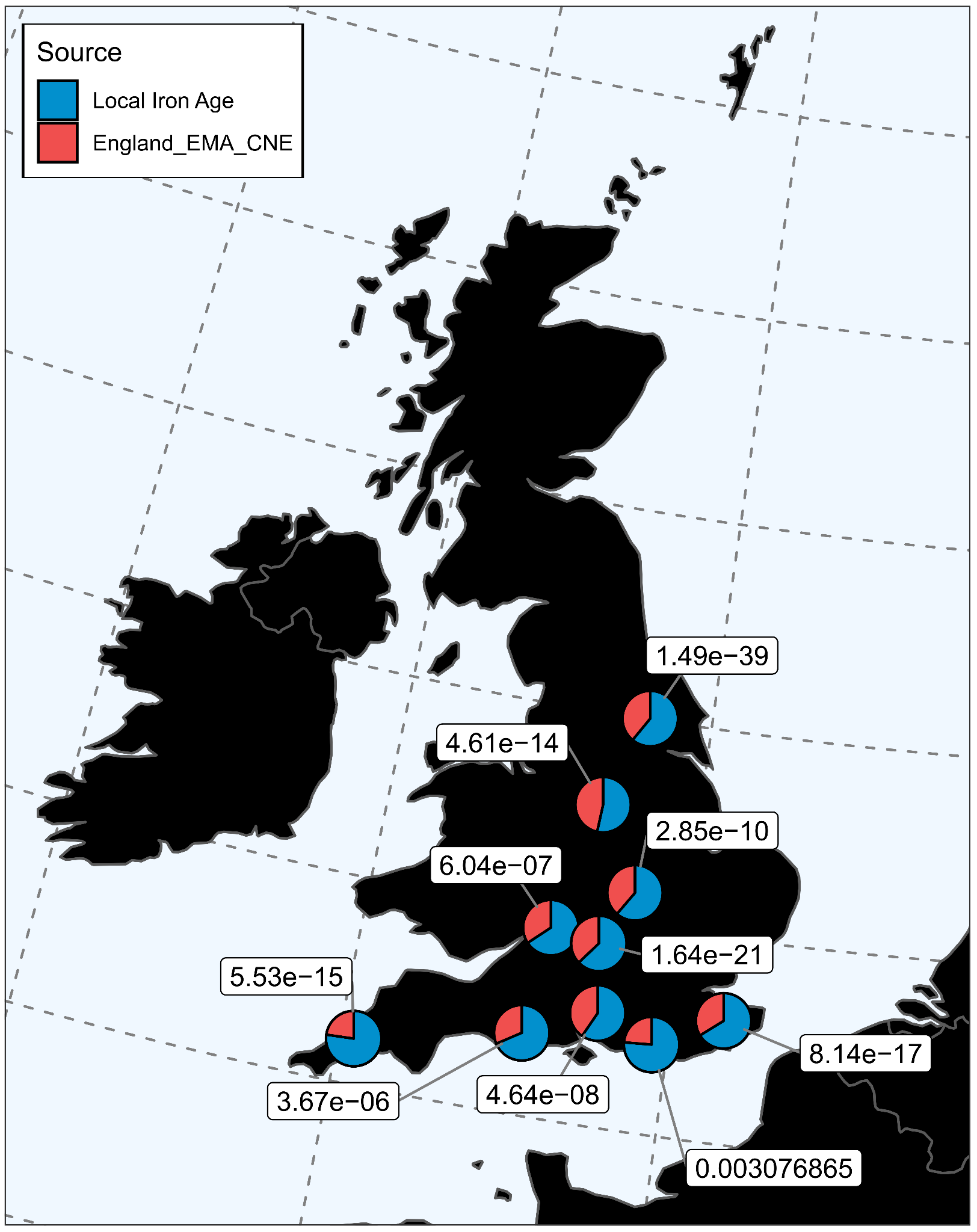


***Supplementary Figure 5.12. qpAdm analysis using region-specific sources.*** *P-values and admixture estimates obtained from modelling present-day English regions as a two-way admixture between their corresponding local Iron Age population and England_EMA_CNE using qpAdm. Samples sizes for local Iron Age populations are indicated in Supplementary Table 5.9.*

This observation is congruent with observations made by Patterson et al. 2021[^81^](https://paperpile.com/c/hqnLqQ/3Th9). They demonstrated that Early European Farmer (*EEF*) ancestry increased in southern Britain (England and Wales) due to incorporation of migrants who arrived between 1000 and 875 BCE and over previous centuries, and who were genetically most similar to ancient individuals from France. To better compare the results of this study with our observations, we reproduced the approach of decomposing ancient English ancestry into three ancestral sources (Balkan Neolithic farmers with minimal hunter–gatherer admixture (*EEF*), Yamnaya and Poltavka pastoralists (*Steppe*), and 18 Mesolithic hunter–gatherers from across western Europe (*WHG*)) using the same set of right and outgroup populations (Supp. Table 5.10).

Similar to the results of Patterson et al. 2021, we observe a marked increase of *EEF* ancestry in England (Supp. Fig. 5.13, Supp. Fig. 5.14a) (but not Scotland (Supp. Fig. 5.14a)) during the Bronze Age, reaching a local maximum during the Iron Age, which is consistent with prolonged migration from southern Europe into southern Britain during the Middle and Late Bronze Age (Supp.Fig. 5.13, Supp. Fig. 5.14a, Supp. Table 5.11). In contrast, with the beginning of the Early Middle Ages, we note a drop in *EEF* ancestry that reflects the introduction of *Steppe-* and *WHG*-enriched CNE ancestry. During this time, the English population closely resembles Iron Age and medieval populations from northern Germany and Scandinavia (Supp. Fig. 5.14b, Supp. Table 5.12), reflecting the demographic changes illustrated with previous PCA, *F*_4_, ADMIXTURE analyses. This depletion of *EEF* ancestry continued at least during the following Viking period corresponding to the Scandinavian origin of migrants during this time period. However, when analysing present-day English (1000 Genomes samples from Cornwall and Kent), we infer that those present-day individuals on the other hand harbour higher levels of *EEF* ancestry than the population of the preceding Early Middle Ages (Supp. Fig. 5.13, Supp. Fig. 5.14a) but lower levels of WHG ancestry (Supp. Fig. 5.14b). In fact, we observe in present-day English individuals the highest average proportion of *EEF* ancestry since the end of the Neolithic. We therefore conclude that that *EEF* ancestry must have risen again after the end of the Early Middle Ages, although there are so far no genomes from later medieval or early modern period England. The extent of this introgression event was comparable to the increase of *EEF* ancestry from the MBA to the IA, and resulted in an average *EEF* proportion in present-day English higher than ever before since the Neolithic, making it implausible that a resurgence of preceding local British Iron Age groups was the potential cause of this genetic shift. More plausible, the rise of *EEF* ancestry was due to resuming migration from the same or at least a genetically similar continental group that already introduced *EEF*-enriched ancestry to England during the Bronze Age, and that we see also in some early medieval individuals as described above.


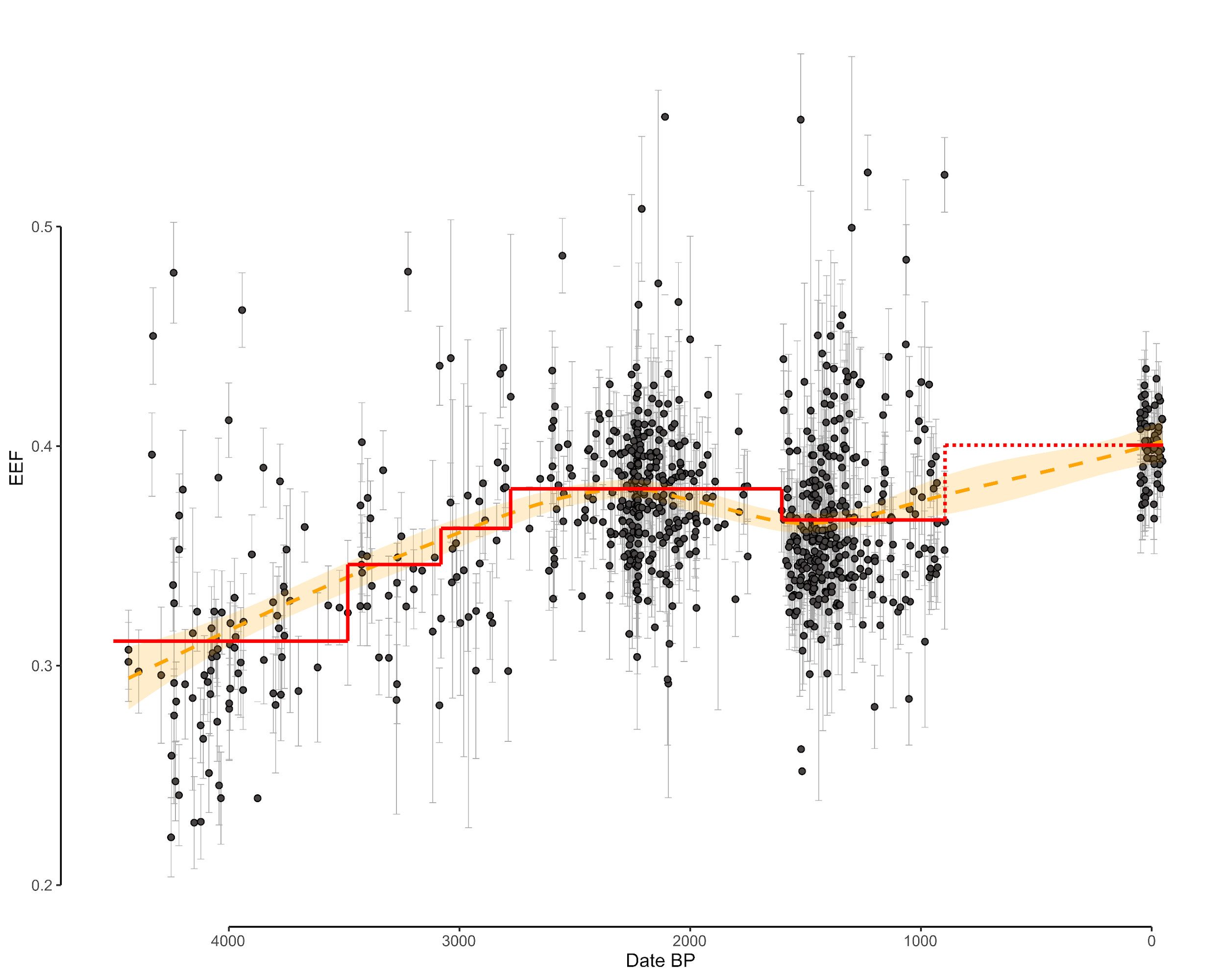


***Supplementary Figure 5.13. Early European Framer ancestry as inferred using qpAdm in ancient and present-day English individuals (n = 825).*** *Shown is a Loess Regression and corresponding confidence interval in orange and the mean per period (EBA, MBA, LBA, IA, EMA, and present-day) in red. Error bars represent ± 1 standard error.*


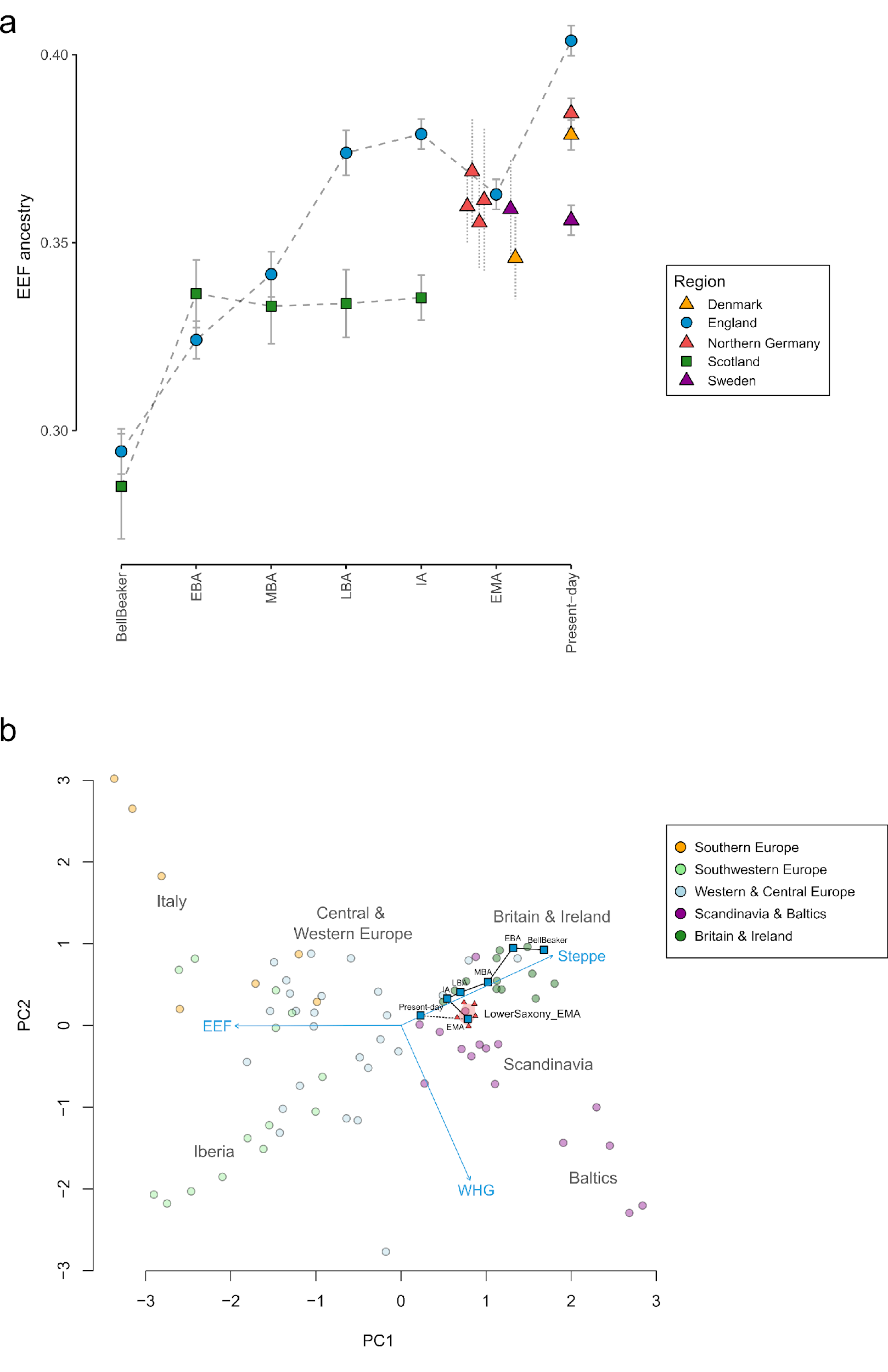


***Supplementary Figure 5.14, Changes in ancestral admixture component.*** *a) Early European Farmer ancestry through time as inferred using qpAdm. For Germany during the Early Middle Ages we plot the sites of Hiddestorf, Liebenau, Issendorf, and Schortens. Error bars represent ± 1 standard error. Samples sizes are indicated in Supplementary Table 5.11. b) PCA of WHG, EEF, and Steppe ancestry estimates for 104 ancient and one present-day populations. Samples sizes are indicated in Supplementary Table 5.12.*

To more directly investigate changing affinities to continental western European populations, we tested the affinity of ancient English individuals to present-day French in relation to our WBI reference group using the *F*_4_ statistic *F*_4_(CHB.SG, *Test*; WBI, France) (Supp. Fig. 5.15a, Supp. Table 5.13). Consistent with the increase in *EEF* ancestry we observed intensiving affinity to present-day French during the Bronze and Iron Age. Again, during the Early Middle Ages coinciding with the introgression of CNE ancestry, we note a decline of affinity to present-day French to a pre-LBA level. On the other hand, present-day English show elevated affinity to present-day French, higher than any time before in the BA and IA, congruent with their higher proportions of *EEF* ancestry. A corresponding *F*_4_ statistic of the form *F*_4_(YRI.SG, *Test*; WBI, CNE) demonstrates that these results are not influenced by the introduction of northern European ancestry during any time in the Bronze or Iron Age (supp. Fig. 5.15b, Supp. Table 5.14). Instead, substantial proportions of *EEF*-enriched ancestry must have entered England after the Early Middle Ages, perhaps as continuation of a process that already started during the Bronze Age. The observation of outlier individuals with southern/western European-related ancestry in early medieval and Viking period England[^120^](https://paperpile.com/c/hqnLqQ/spmY) additionally supports a continuing influx of southern/western European ancestry into England also during times of CNE introgression. Therefore, our results show that additional French-related ancestry must have entered England after the Roman Period as suggested by previous analyses[^56,120,181^](https://paperpile.com/c/hqnLqQ/vtUXz+eLLr1+spmY). Margaryan et al.[^120^](https://paperpile.com/c/hqnLqQ/spmY) suggested that the presence of southern European ancestry may be related to the Norman Conquest and the associated increase in population movement during that era.


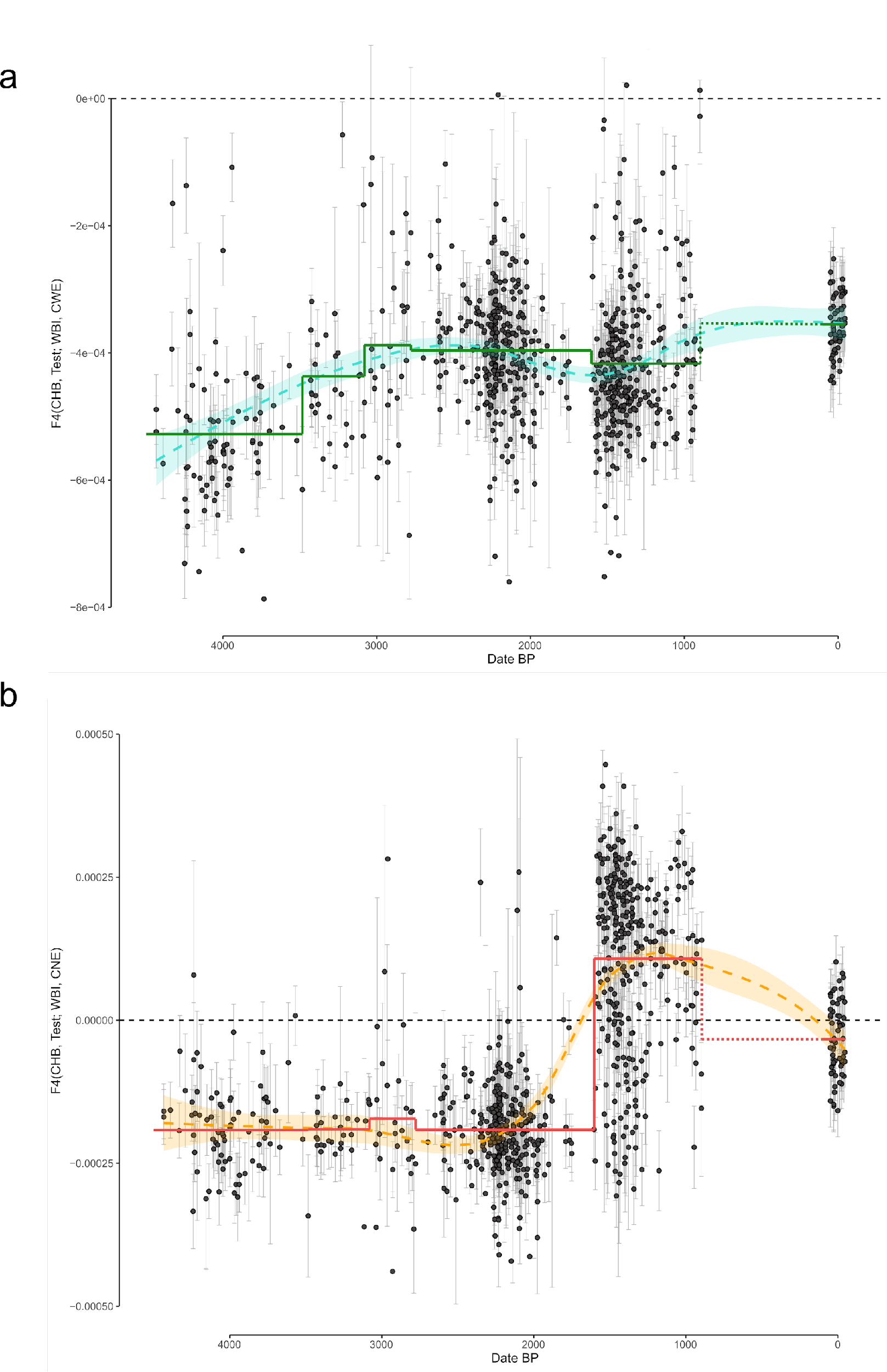


***Supplementary Figure 5.15, Changing affinities in ancient and present-day English individuals (n = 825) in respect to continental populations****. a) F_4_ statistic of the form F_4_ (CHB, X; WBI, France). Shown is the Loess Regression and corresponding confidence interval in turquoise and the mean per period (EBA, MBA, LBA, IA, EMA, and present-day) in darkgreen. Error bars represent ± 2 standard errors. b) a) Same for the F_4_ statistic of the form F_4_(YRI, X; WBI, CNE).*

To further test the relationship between those ancient admixture components, we used an Admixture Graph History (AGH) approach developed by Pugach et al. This approach relies on the principle that after a second admixture event has introduced a new ancestry component into an already admixed population, the proportion of the two older ancestry components in each individual is re-adjusted by an amount which depends on the proportion of the newly arriving component in the individual. This means that the distribution of the first two ancestry components across individuals in the population will covary with the new ancestry, yet the ratio of the older components throughout the population will be independent of the recent ancestry. Thus, the covariance of the recent ancestry and the ratio of the two older ancestries should be zero.

First, we tested if this approach was able to identify the correct sequence of the admixture events associated with the transition from the Mesolithic to the Neolithic, and from the Middle Neolithic to the Late Neolithic/Early Bronze Age. We compared all possible orderings of the three ancestries *EEF*, *WHG*, and *Steppe ancestry* in 1,678 present-day English individuals. We find that the ordering that produces the covariance closest to zero is (EEF, WHG), Steppe (-0.003000143), agreeing with the true order of admixture vents.

Consequently, to determine the sequence of admixture events in post-Iron Age England, we compared all possible orderings of the three ancestries WBI (*England_LIA_Roman*), CNE (England_EMA_CNE), and CWE (*France_IA*) in 1,300 present-day English individuals (as inferred using *qpAdm* as mentioned earlier in this section) (Supp. Fig. 5.16) and chose the one which produced the least absolute value of covariance. We find that the ordering that produces the covariance closest to zero is (WBI, CNE), CWE (0.0201693), followed by (CNE, CWE), WBI (0.04975982). In contrast, a sequence in which CWE and WBI admixed before the arrival of CNE ancestry produces the largest covariance deviation from zero (0.06992912). The same result was obtained using supervised ADMIXTURE and present-day source populations for 1,678 present-day English (Supp. Fig. 5.17). Similarly, we find that the ordering that produces the covariance closest to zero is (WBI, CNE), CWE (9.469532). This implies that most of the additional CWE ancestry in present-day English arrived either after the introgression of CNE ancestry into England or only partly with CNE ancestry during the Early Middle Ages.


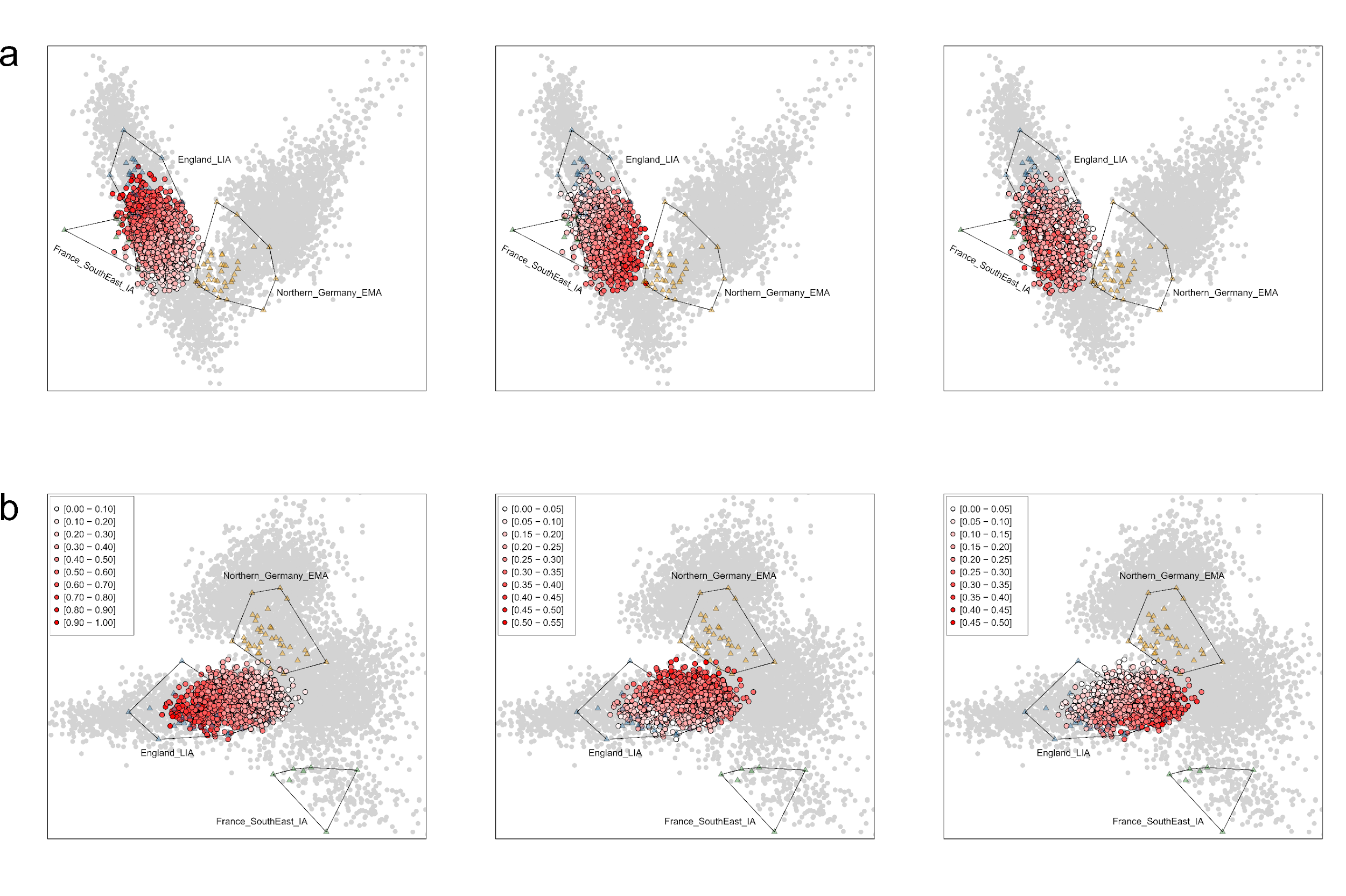


***Supplementary Figure 5.16, qpAdm analysis of present-day English.*** *Individual-based ancestry inference of England_LIA_Roman, England_EMA_CNE, and France_IA ancestry for 1,300 present-day English individuals using qpAdm. a) Admixture proportions projected onto our North Sea PCA from left to right: England_LIA_Roman, England_EMA_CNE, and France_IA. b) Same as in a) but projected onto our Northwestern European PCA.*


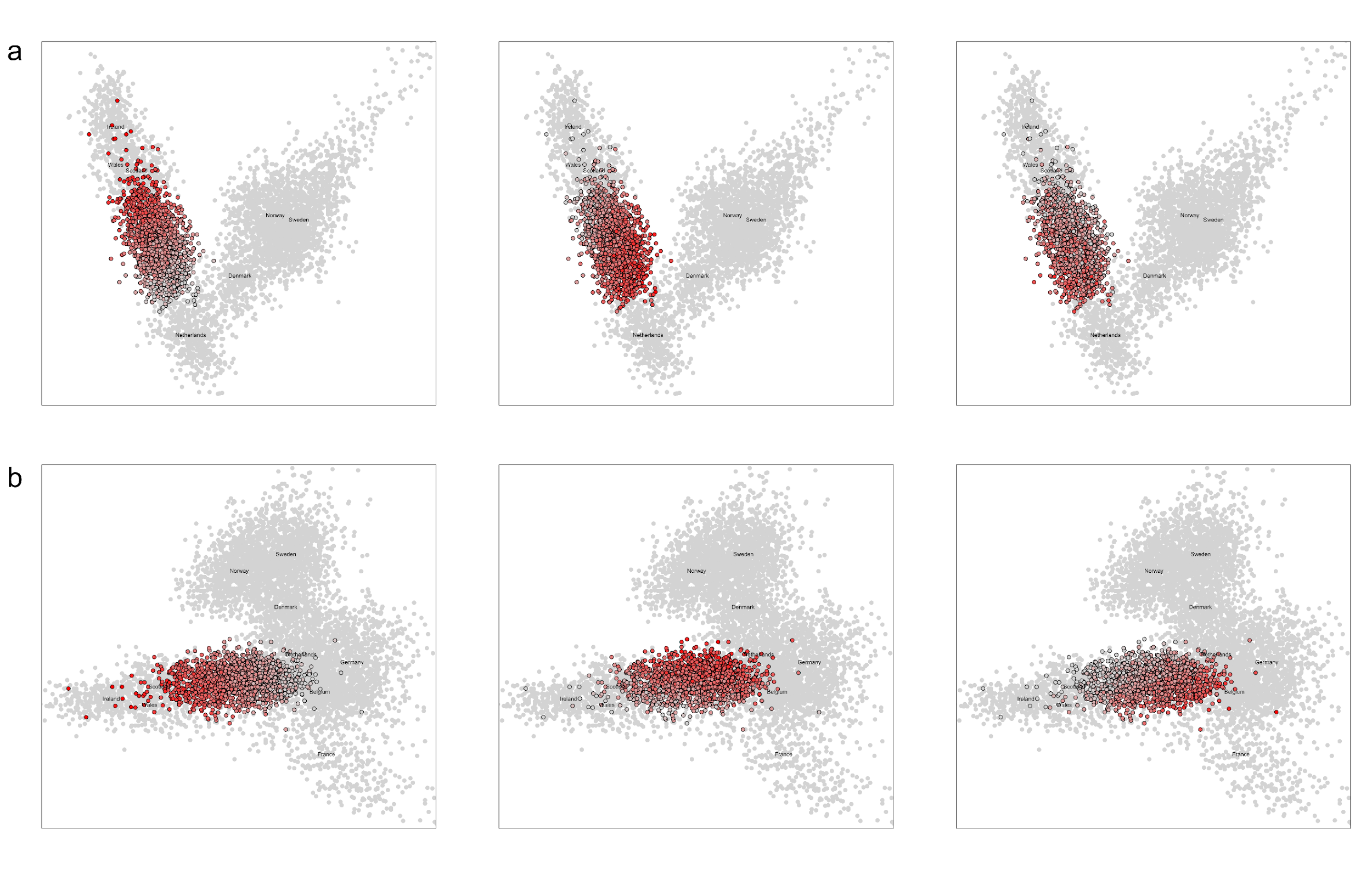
***Supplementary Figure 5.17, Supervised ADMIXTURE analysis of present-day English.*** *Individual-based ancestry inference of WBI, CNE, and CWE ancestry for 1,678 present-day English individuals using supervised ADMIXTURE. a) Admixture proportions projected onto our North Sea PCA from left to right: England_LIA_Roman, England_EMA_CNE, and France_IA. b) Same as in a) but projected onto our Northwestern European PCA.*

### Quantifying the impact on present-day England on a regional level

Finally, we quantified the impact of continental western European ancestry introgression after the Iron Age in present-day English on a regional level. To do this, we compared the estimates of local English, CNE, and continental western European (from here CWE) ancestry in present-day English populations obtained with different methods. Those methods are:

1. A three-way *qpAdm* model using *England_LIA_Roman* as source of local British ancestry, *England_EMA_CNE* as source of CNE ancestry, and *France_IA* as source of CWE ancestry) (Supp. Table 5.15).
2. A three-way *qpAdm* model using *WorthMatravers,* a WBI-rich early medieval site, as source of local British ancestry, *England_EMA_CNE* as source of CNE ancestry, and *France_IA* as source of CWE ancestry) (Supp. Table 5.17).
3. A three-way *qpAdm* model using *England_LIA_Roman* as source of local British ancestry, *LowerSaxony_EMA* as source of CNE ancestry, and *France_IA* as source of CWE ancestry) (Supp. Table 5.16).
4. A supervised ADMIXTURE model using present-day Irish, Northern Irish, Scottish, and Welsh (WBI) as source of local British ancestry, present-day Danish and northern Germans (CNE) as source of CNE ancestry, and present-day Belgians and French as source of CWE ancestry (Supp. Table 5.18).

While all four approaches employ different methods and/or reference data, obtained estimates of the three ancestries are highly similar (Supp. Fig. 5.18a/c) and strongly correlated with each other (Supp. Fig. 5.18b/d) as well as with different methods of quantifying genetic affinity like PCA and *F*_4_ (Supp. Fig. 5.19).


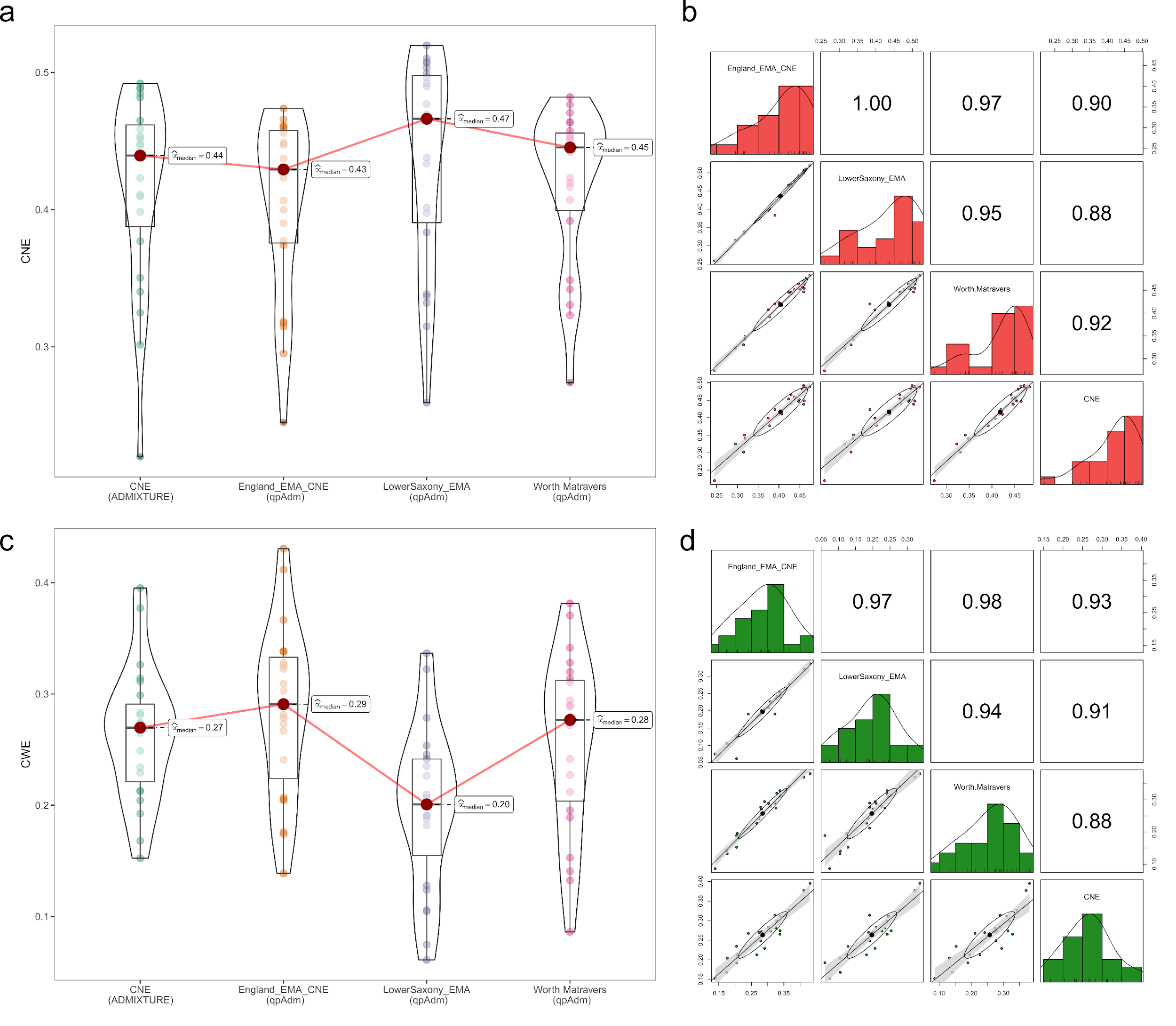


***Supplementary Figure 5.18, Validation of admixture estimates using different source populations.*** *a) Distribution of CNE ancestry in 23 present-day English regions as inferred using qpAdm and supervised ADMIXTURE shown as Boxplot. As a source of CNE ancestry in qpAdm we used England_EMA_CNE (n = 109) and LowerSaxony_EMA (n = 39). For England_EMA_CNE, we also calculated a model where England_LIA_Roman was replaced with the early medieval Worth Matravers population (n = 16). Results of supervised ADMIXTURE at K = 3 using CNE (n = 407), WBI (n = 667), and CWE (n = 1074) are shown for comparison. Median estimates are denoted. Bounds of the Box represent the 25^th^ and 75^th^ Percentile. The centre represents the median. Whiskers represent the smallest value greater than the 25^th^ Percentile minus 1.5 times the interquartile range and largest value less than the 75^th^ Percentile plus 1.5 times the interquartile range, respectively. Outliers present the minimum and maximum values in the data. Samples sizes for present-day PoBI sampling regions are indicated in Supplementary Table 5.15-18. b) Correlogram illustrating pairwise correlations between different measurements of CNE ancestry using the aforementioned sources in qpAdm. Error bands represent ± 2 standard errors. c) Same as in a) but for CWE ancestry. d) same as in b) but for CWE ancestry.*


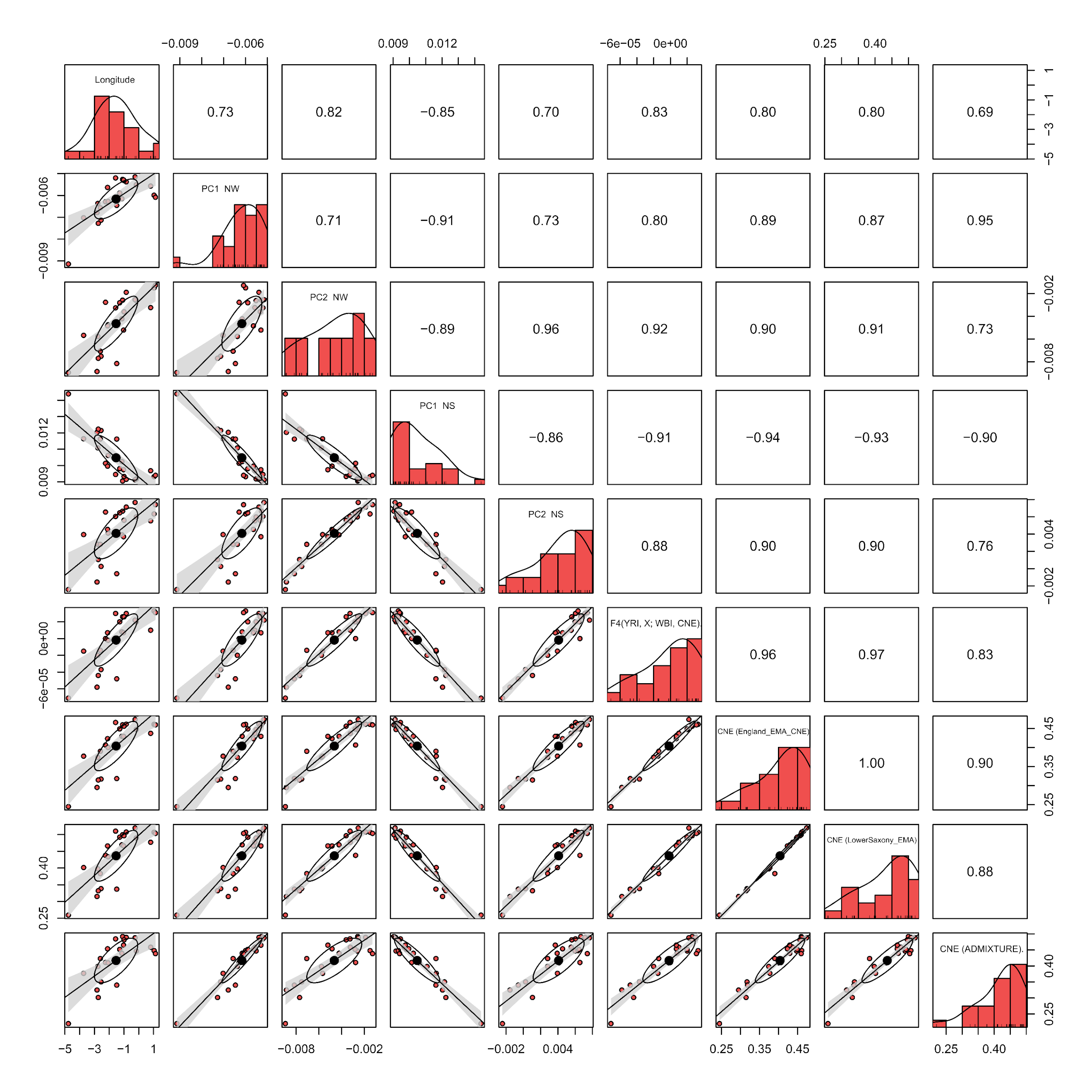
***Supplementary Figure 5.19, Validation of CNE estimates across different methods.*** *Correlogram illustrating pairwise correlations between different measurement of genetic affinity to CNE (mean PC1 and PC2 coordinates in our Northwestern European PCA, mean PC1 and PC2 coordinates in our North Sea PCA, F_4_ statistics of the form F_4_(YRI, X; WBI, CNE), supervised ADMIXTURE estimates at K = 2, qpAdm three-way admixture estimates using either England_EMA_CNE or LowerSaxony_EMA as sources) across 23 present-day English populations. Spearman's r for each pairwise correlation test are indicated in the upper triangle of the matrix. Error bands represent ± 2 standard errors.*

Additionally, we investigated the effects of different sample sizes and sequencing data generation techniques on our estimates. We grouped the available *England_LIA_Roman*, *England_EMA_CNE*, and *France_IA* data according to the sequencing approach (1240k capture versus shogun sequencing) into three groups: i) exclusively 1240 capture data, ii) exclusively shotgun sequencing data and ii) mixed 1240k capture and shotgun sequencing data. For each of these groups, two subgroups were created: a) all available samples per population (resulting in large differences between source population sample sizes), and b) randomly downsampled subsets of the same sample size (across all three source populations). For those six combinations of source populations, we calculated the three-way admixture model of present-day English PoBI sampling regions described earlier and compared the inferred estimates of *England_EMA_CNE* and *France_IA* ancestry. While there are small absolute differences in median CNE ancestry (ranging between 49% and 45% across all six groupings) (Supp. Fig. 5.20a) and median CWE ancestry (ranging between 23% and 30% across all six groupings) (Supp. Fig. 5.20c), the distribution of CNE (Supp. Fig. 5.20b) and CWE (Supp. Fig. 5.20d) ancestry is highly correlated across the different groupings (Spearmans’ |*r*| > 0.92). Miniscule differences and deviations are expected since all six groupings comprise different individuals, which affects the population genetic conditions of the source populations. Completely similar results are therefore impossible. Yet, we show in general that our inferred admixture proportions for present-day England are stable and reliable even using heterogeneous data or small sample sizes and that the choice of sample size or sequencing technique does not severely affect our *qpAdm* model outcome.


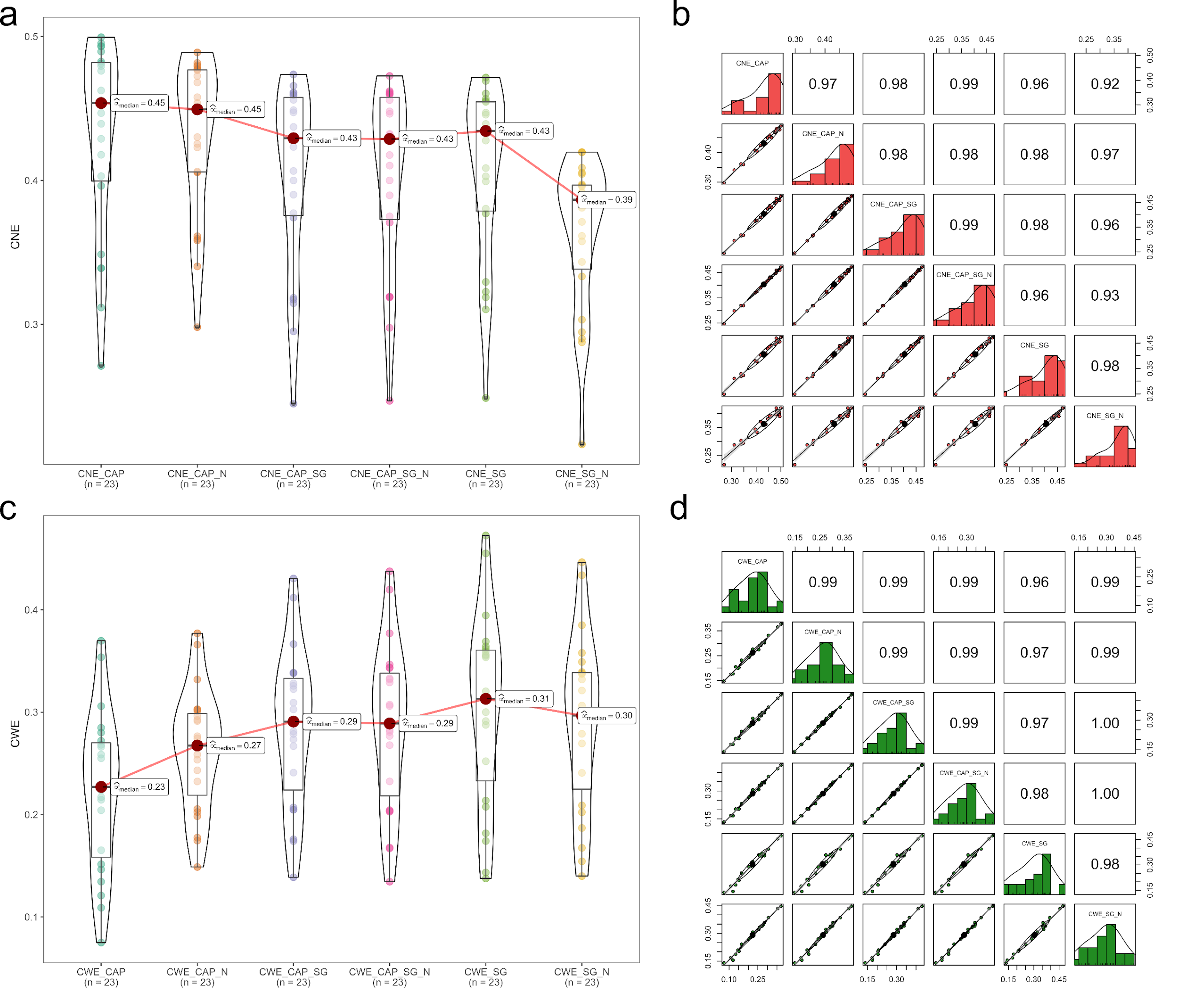


***Supplementary Figure 5.20, Validation of admixture estimates using different input data.*** *a) Distribution of CNE ancestry in 23 present-day English regions as inferred using qpAdm shown as Boxplots. Input data was divided in six categories: 1240k capture data only (CAP, n = 86, 265, and 7 for England_EMA_CNE, England_IA, and France_IA respectively), shotgun sequencing data only (SG, n = 23, 8, and 19 for England_EMA_CNE, England_IA, and France_IA respectively), and 1240k capture and shotgun sequencing data mixed (CAP_SG, n = 109, 273, and 26 for England_EMA_CNE, England_IA, and France_IA respectively). For each of these categories a downsampled version was generated so that all three admixture sources had the same sample size (CAP_N, SG_N, CAP_SG_N; n = 7, 8, and 26 respectively). Median estimates are denoted. Bounds of the Box represent the 25^th^ and 75^th^ Percentile. The centre represents the median. Whiskers represent the smallest value greater than the 25^th^ Percentile minus 1.5 times the interquartile range and largest value less than the 75^th^ Percentile plus 1.5 times the interquartile range, respectively. Outliers present the minimum and maximum values in the data. b) Correlogram illustrating pairwise correlations between different measurements of CNE ancestry using the aforementioned sources in qpAdm. Samples sizes for present-day PoBI sampling regions are indicated in Supplementary Table 5.15. Error bands represent ± 2 standard errors. c) Same as in a) but for CWE ancestry. d) same as in b) but for CWE ancestry.*

Using *LowerSaxony_EMA* as CNE source and *France_IA* as CWE source, we increase the number of significant (*p* > 0.05) *qpAdm* models for the 23 present-day English populations in comparison to the two-way model from zero to 20. For *England_EMA_CNE*, the number increases from zero to 23 (Supp. Fig. 5.21).


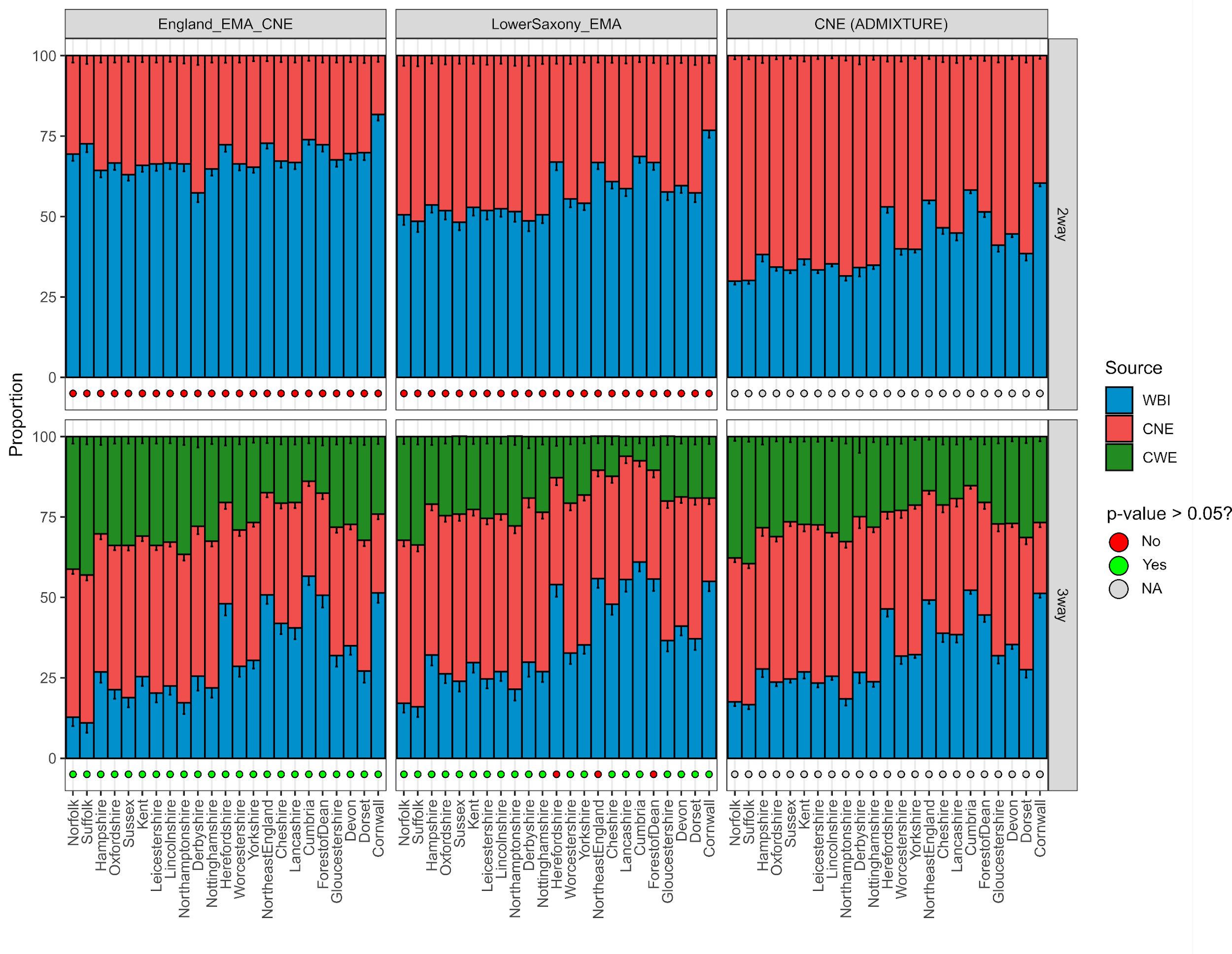


***Supplementary Figure 5.21, Ancestry proportions of 23 present-day English populations inferred using qpAdm and supervised ADMIXTURE****. Upper) Modern England was modelled using two proximal sources representing CNE (England_EMA_CNE, LowerSaxony_EMA, CNE; n = 109, 39, and 407, respectively) and WBI (England_LIA_Roman, WBI; n = 32 and 667, respectively) ancestry. Lower) Modern England was modelled using three proximal sources representing CNE (England_EMA_CNE, LowerSaxony_EMA, CNE) and WBI (England_LIA_Roman, WBI) ancestry, and a continental western European (France_IA, CWE; n = 26 and 1074, respectively) ancestry; Error bars indicate ± 1 standard error obtained from qpAdm or ± 1 standard error of the mean calculated from averaged supervised ADMIXTURE components. qpAdm p-values higher than 0.05 are highlighted in green, p-values lower than 0.05 in red. For the supervised ADMIXTURE analyses no p-values were produced. Samples sizes for present-day PoBI sampling regions are indicated in Supplementary Table 5.15-18.*

According to the *qpAdm* models, *France_IA*-related ancestry is more highly concentrated in southern and southeastern England, especially in East Anglia where it provides, depending on the model, up to 27% (model 3) or 43% (model 1) of the ancestry. On the other hand, it is nearly absent in the northern parts of England as well as in Wales, Scotland, and Ireland (Fig. 5, Supp. Fig. 5.22). *England_EMA_CNE*/*LowerSaxony_EMA* ancestry is relatively homogenous distributed across the Midlands, East of England, and South East, but found in lower quantities in the North, South West, and along the Welsh border (Fig. 5, Supp. Fig. 5.22). We note that the distribution and proportion of continental ancestry across Britain and Ireland is very similar regardless which proxy for the immigrant CNE and CWE ancestry is used, even when considering present-day sources in a supervised ADMIXTURE setup (Supp. Fig. 5.18, Supp. Fig. 5.21). Using ADMIXTURE, we estimate 40.7% ± 0.4% CNE, 33.9% ± 0.5% WBI, and 25.4% ± 0.4% CWE ancestry in England, which is strongly consistent with our *qpAdm* models using ancient sources.


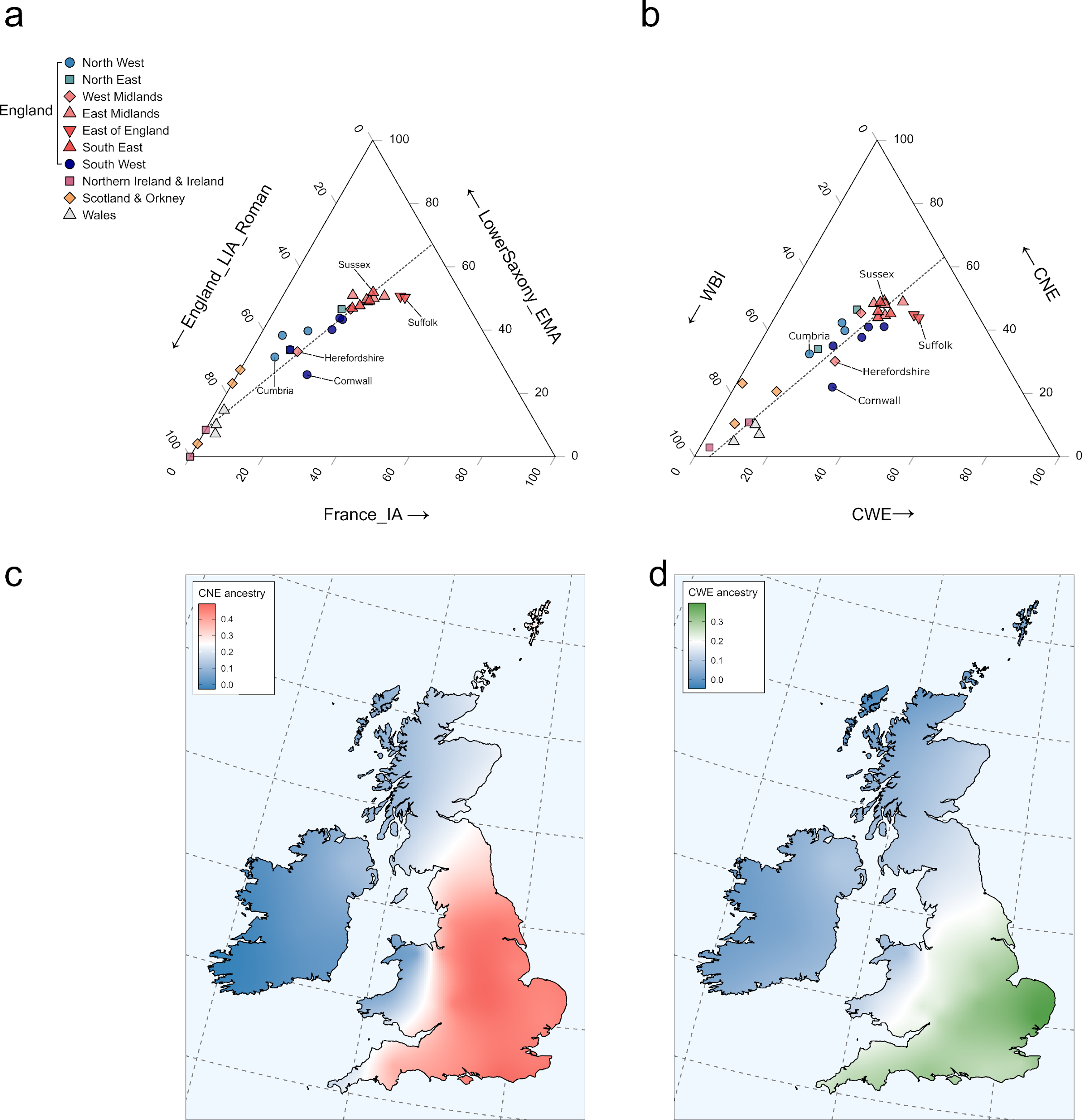


***Supplementary Figure 5.22, Population structure across Britain and Ireland.*** *a) Ternary plot of present-day British-Irish populations as a 3-way admixture between England_LIA_Roman, LowerSaxony_EMA, and France_IA using qpAdm. b) Ternary plot of present-day British-Irish populations as a 3-way admixture between WBI, CNE, and CWE using supervised ADMIXTURE. c) Geographic distribution of CNE ancestry based on the interpolation of 31 present-day population estimates obtained using supervised ADMIXTURE. d) Same as c) for CWE.*

However, while the Iron Age and Roman Period samples used in the qpAdm models originate from from geographically diverse regions in England, the use of central and northern English individuals as source of the local British ancestry in our *qpAdms* models may cause overestimation of *France_IA*-related ancestry in present-day southern England since Patterson et al. 2021 demonstrated that *EEF*-enriched southern European ancestry was more prevalent in the south than in the north of Iron Age Britain. Additionally, sampling from Roman England is still sparse in comparison to the Iron Age and may not fully capture the documented cosmopolitan character of this time period. We therefore used in model ii) the early medieval site Worth Maltravers as source of local English ancestry in the fourth model within *qpAdm* together with *England_EMA_CNE*. This site should in theory harbour *France_IA*-related ancestry that entered England before the Early Middle Ages, even after the Iron Age. Furthermore, it is located in the south of England, where *France_IA*-related ancestry most plausibly must have entered and where we still detect above-average levels of *France_IA* ancestry today. Comparing *England_LIA* and *Worth Matravers* as sources of local British ancestry, we detect no substantial difference in *France_IA*-related ancestry among present-day English populations (Fig. 5b) . This indicates that substantial proportions of *France_IA* ancestry measured in present-day England must have entered after the Roman Period, some time between the Early Middle Ages and the modern period.

To which degree additional *France_IA*-related ancestry entered after the Iron Age, varies between the British-Irish regions. To better understand this process and quantify the impact of CWE ancestry on present-day Britain and Ireland in relation to pre-Iron Age immigration at a regional level, we computed for 32 British-Irish populations a three-way admixture model in *qpAdm* using *England_EMA_CNE* and *France_IA* as immigrant source ancestries. As source of local ancestry we used either i) a corresponding local Early Bronze Age source (*England_C_EBA*, *Scotland_C_EBA*, or *Ireland_C_EBA*) (Supp. Fig. 5.23a, Supp. Table 5.19) or ii) a corresponding local Iron Age source (*England_LIA*, *Wales_IA*, *Scotland_LIA*, or *Ireland_MA*) (for Ireland, no Iron Age population was published so far) (Supp. Fig. 5.23b, Supp. Table 5.20). We find the largest differences in required *France_IA* ancestry between the two models in Ireland, Wales, and western Scotland (Argyll and Bute), which might suggest that the majority of *France_IA* ancestry detected in those present-day populations entered Ireland and western Britain prior to the Iron Age. In England, especially in the East and Southeast, the differences between the models are relatively smaller, suggesting that comparably similar amounts entered also after the Iron Age.


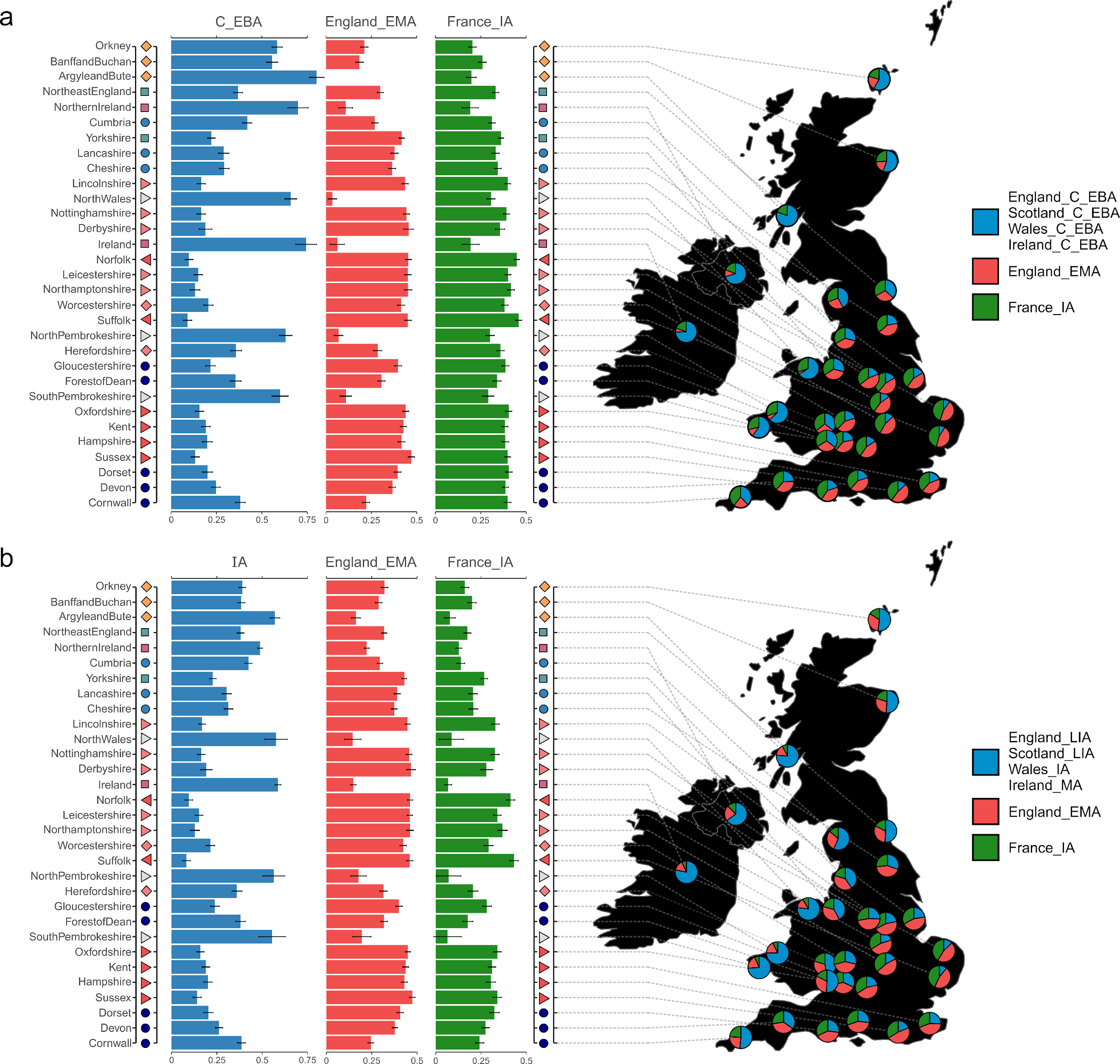


***Supplementary Figure 5.23, European ancestry profiles of the British-Irish regions.*** *a) The pie charts represent the ancestry profiles of the British-Irish PoBI sampling regions obtained using qpAdm. Each region was modelled as a three-way admixture between their corresponding local Early Bronze Age source (e.g. present-day England modelled using England_C_EBA (n = 46), Wales using Wales_C_EBA (n = 1), Scotland using Scotland_C_EBA (n = 14), and Ireland using Ireland_C_EBA (n = 3)), England_EMA_CNE (n = 109), and France_IA (n =26). The graph in the left part of the plot depicts the same ancestry profiles of the sampling regions as barplots including error bars of one standard error obtained from qpAdm. b) Same but using a corresponding Iron Age population as source of local ancestry (England_LIA_Roman, n = 32; Wales_IA, n = 6; Scotland_LIA, n = 13). Since so far no Iron Age samples from Ireland are reported, we used the medieval Kilteasheen population (n = 39) as proxy. Samples sizes for present-day PoBI sampling regions are indicated in Supplementary Table 5.19-20. Error bars represent ± 1 standard error.*

Summarising, we infer across all four qpAdm and supervised ADMIXTURE models approximately between 25% and 50% CNE ancestry in modern English populations, which is consistent with previous estimates based on modern and ancient DNA[^56,120,137^](https://paperpile.com/c/hqnLqQ/eLLr1+I6Awn+spmY). Combining CWE-related and ancient CNE ancestry as “non-local ancestry”, we find that variation in nonlocal ancestry explains 99% of the variation in mean PC2 position of the tested English region populations in our *Northwestern European PCA* setup. We therefore conclude that in Britain, the genetic structure of the east-to-west (the “Anglo-Celtic”[^178^](https://paperpile.com/c/hqnLqQ/2b2nD)) cline is mainly explained by introgressed continental ancestry that arrived within the last 4,500 years. Especially the variation in population genetic affinity between English regions observed in PCA, as well as *F*_ST_, and *F*_4_ statistics (Supp. Fig. 5.24, Supp. Table 5.21-25) is indeed the product of different proportions of continental admixture. Within England, *England_LIA* ancestry is correlated mainly with longitude (Pearson's |*r*| = 0.77, *t* = -5.5356, *d.f.* = 21, *p* = 1.712e-05), which verifies previous observations of an east-to-west clinal distribution of continental affinity in present-day England^3,41^ and may be related to settlement patterns of immigrating groups during the Middle Ages. This Anglo-Celtic cline is even more pronounced in the British-Irish context, where longitude is even more highly correlated with English Iron Age ancestry (Pearson's |*r*| = 0.86, *t* = -8.9696, *d.f.* = 29, *p* = 7.325e-10). Populations exhibiting lower proportions of *England_LIA* ancestry harbour instead higher fractions of CNE (and CWE) ancestry. *F*_ST_ between *England_EMA_CNE* and present-day English populations is highly correlated with the *England_EMA_CNE* ancestry proportion inferred using *qpAdm* (Pearson's |*r*| = 0.85, *t* = -7.5203, *d.f.* = 21, *p* = 2.187e-07). The same holds true for *F*_ST_ between modern Danes and English populations (Supp. Fig. 5.25a) (Pearson's |*r*| = 0.91, *t* = -10.091, *d.f.* = 21, *p* = 1.654e-09) (Supp. Fig. 6.14b). Similarly, *F*_ST_ between modern French and present-day English populations is highly correlated with the *France_IA* ancestry proportion inferred using *qpAdm* (Pearson's |*r*| = 0.86, *t* = -7.5472, *d.f.* = 21, *p* = 2.069e-07) (Supp. Fig. 5.25b).


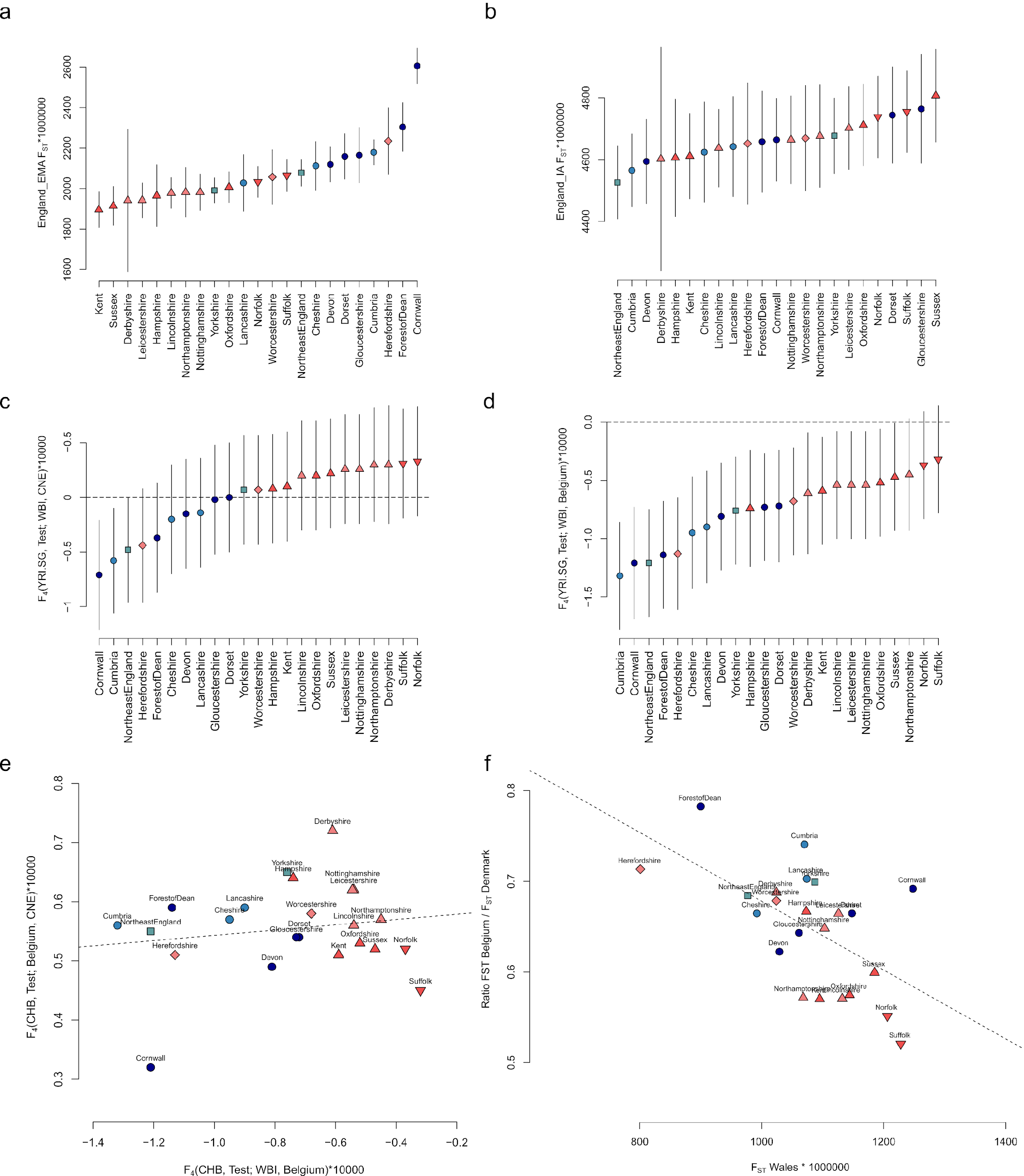


***Supplementary Figure 5.24, Differentiating patterns of genetic affinity across 23 present-day English populations as seen in f-statistics****. Samples sizes for present-day PoBI sampling regions are indicated in Supplementary Table 5.21-25. a) F_ST_ between each region and England_EMA (n = 285). Error bars represent ± 3 standard errors. b) F_ST_ between each region and England_IA (n = 290). Error bars represent ± 3 standard errors. c) F_4_ statistics of the form F_4_(YRI, Test; WBI, CNE). Error bars represent ± 2 standard errors. d) F_4_ statistics of the form F_4_(YRI.SG, Test; WBI, Belgium). Error bars represent ± 2 standard errors. e) Scatterplot of the F_4_ statistics F_4_(CHB, Test; Belgium, CNE) and F_4_(CHB, Test; WBI, Belgium). f) Scatterplot of the ratio F_ST_ to Belgium/ F_ST_ to Denmark and the F_ST_ to Wales for each region.*


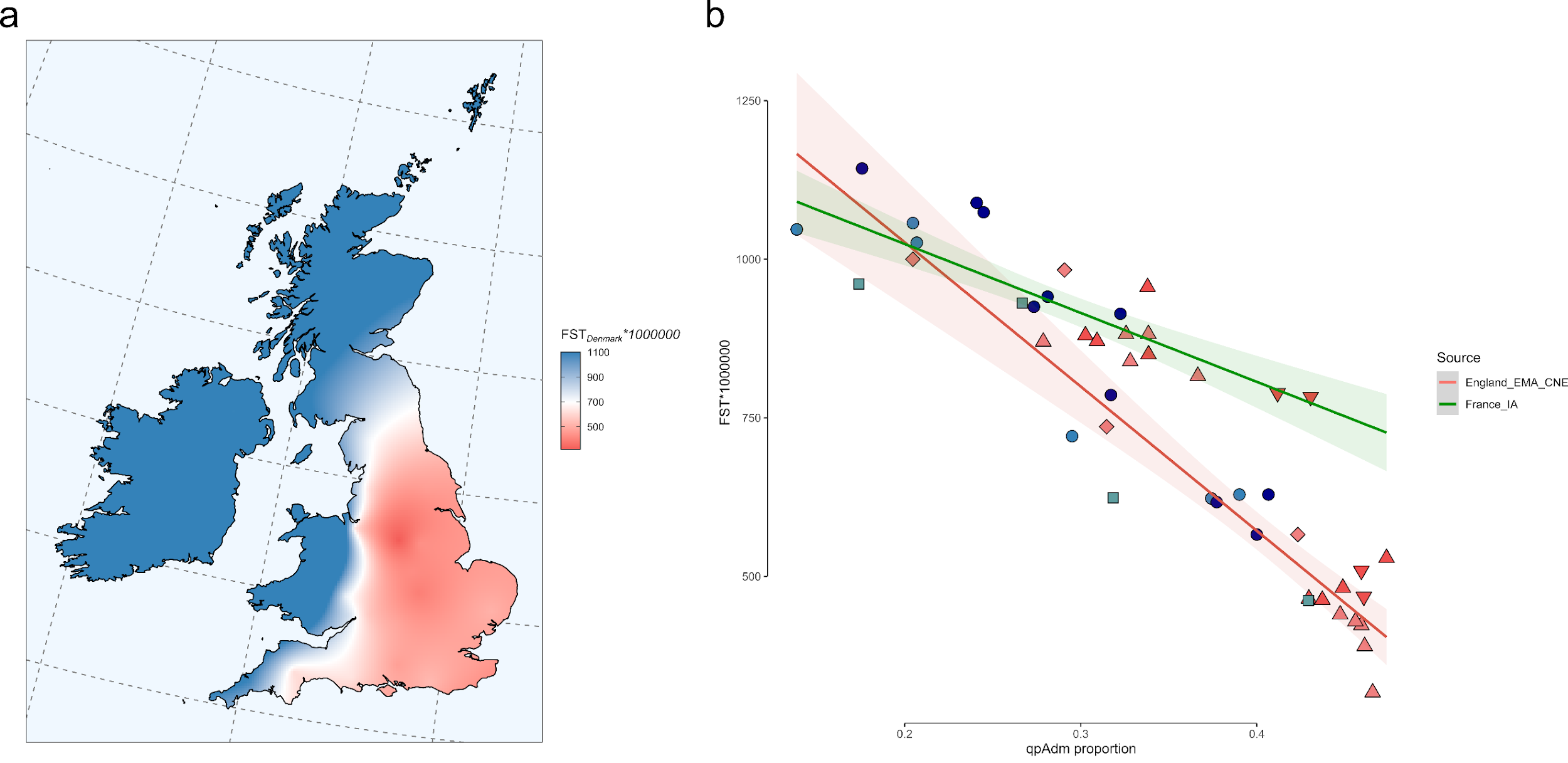


***Supplementary Figure 5.25, Differentiating genetic affinities across present-day Britain and Ireland caused by historic admixture.*** *a) Geographic distribution of the F_ST_ distances between 32 present-day populations from Britain and Ireland and modern Denmark. b) Correlations between F_ST_ distances (between France/Denmark and 23 English populations) and qpAdm ancestry proportions obtained using a three-way admixture model (England_EMA_CNE & France_IA). The error band represents ± 2 standard errors.*

#

#

# Supplementary Note 6 *Scandinavian Peninsula ancestry in ancient and modern Britain*

## Early medieval England

The migration of Germanic tribes to England comprised three major ethnic entities according to the historical sources: Saxons, Angles, and Jutes[^182^](https://paperpile.com/c/hqnLqQ/HD1jA). Since the Saxons were supposed to live in Lower Saxony, the Angles in southern Jutland, and the Jutes and northern Jutland[^182^](https://paperpile.com/c/hqnLqQ/HD1jA), varying fractions of ancestry from the Scandinavian Peninsula (Sweden & Norway) in early medieval English could be informative about geographic origin of the respective individuals insofar as they allow to identify more northern or southern genetic affinities that again could correspond to the named ethnic groupings. Consequently, we assume that we might find larger Scandinavian Peninsula ancestry components in individuals from the historical Jutish settlement area in Kent, intermediate contributions in Anglian areas in north-eastern England, and the lowest contributions in the Saxon kingdoms of southern and south-western England.

### Identifying outliers

The majority of early medieval samples from England cluster in PCA closely together with present-day Danes, Northern Germans, and Northern Dutch. However, in the *North Sea PCA* as well as in the *Northwestern European PCA* some individuals tend to be shifted northwards across PC1 in the direction of modern Swedish and Norwegians.

To investigate whether this shift was reflected in allele frequency correlations, we computed for each medieval genome (*TestA*) the *F*_4_ statistic of the form *F*_4_(YRI, *Test*; CNE, NOR), where *NOR* represents present-day Swedes and Norwegians grouped together (Supp. Fig. 6.1, Supp. Table 6.1) and *Test* each early medieval English sample. While a majority of individuals are asymmetrically related to present-day Danes in comparison to modern Swedes and Norwegians, they are significantly closer to modern Danes, several individuals exhibit a symmetrical relationship to the Danish and the Swedish /Norwegian populations. Those individuals harbour either i) ancestry that is admixed between Denmark and Sweden/Norway, or ii) ancestry basal to the split of southern and northern Scandinavian populations, *e.g.* British-Irish-like WBI ancestry.

None of the *England_EMA* individuals is significantly (*Z* > 2) closer related to *NOR* than to *CNE*. The same pattern was also observed in early medieval Lower Saxony. In contrast, during the Viking Age, at least two English individuals show higher affinities to populations from the Scandinavian Peninsula than to *CNE* (6% of the population). Expectedly, this proportion is also much higher in the Viking Age populations of Sweden (20%) and Norway (63%). On the other hand, 29% of the *England_EMA* and 39% of the *LowerSaxony_EMA* population are significantly closer related to *CNE* than to *NOR*. Consequently, this proportion was much lower during the Viking Age in Norway (7%), Sweden (11%), Denmark (16%), and England (6%).

We further tested whether early medieval individuals from England were asymmetrically related to present-day Swedes or Norwegians using an *F*_4_ statistic of the form *F*_4_(YRI, *Test*; Sweden, Norway) (Supp. Fig. 6.1, Supp. Table 6.2). Only about 4% of the *England_EMA* individuals are closer related to present-day Swedes than to present-day Norwegians, while less than 2% are closer related to Norwegians than to Swedes. Thus, the majority (~95%) is symmetrically related to both populations. Similarly, in early medieval Lower Saxony, only one individual is asymmetrical related to both populations, exhibiting higher affinities to present-day Norwegians. In contrast, 46% of the Viking Age Swedish individuals are significantly closer related to Swedes than to Norwegians. Also, 22% of the English Viking Age and 21% of the Danish Viking Age individuals are closer related to Swedes than to Norwegians, with only one English individual being closer related to Norwegians.


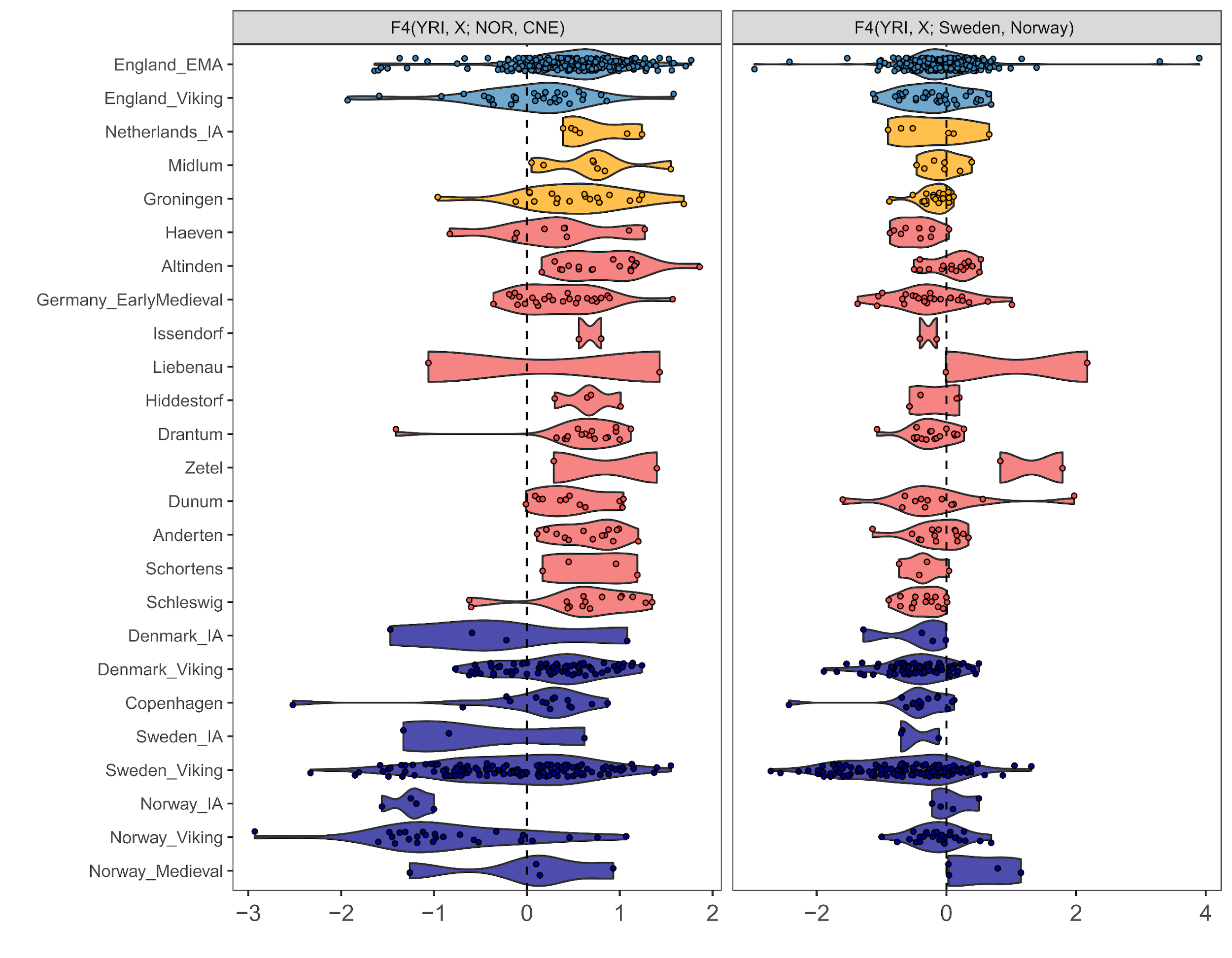


***Supplementary Figure 6.1. Genetic affinities among England_EMA and other ancient northwestern European individuals towards populations from the Scandinavian Peninsula****. left) F_4_-statistics of the form F_4_(YRI, X; NOR, CNE). Positive values indicate that X is closer to CNE (n = 407) than to NOR (n = 1910); negative values indicate that X is closer to Norse than to CNE. Right) F_4_-statistics of the form F_4_(YRI, England_EMA; Sweden, Norway). Negative values indicate that X is closer to Sweden (n = 987) than to Norway (n = 923); positive values indicate that X is closer to Norway than to Sweden.*

### Estimating admixture from the Scandinavian Peninsula

Drawing on the results of the *F*_4_ statistics described above, we performed a supervised ADMIXTURE[^172^](https://paperpile.com/c/hqnLqQ/TNMqU) analysis, setting *K* = 3, treating samples from Norway and Sweden as a distinct additional parental group (NOR, *n* = 1910) (Supp. Fig. 6.2, Supp. Table 6.3). In agreement with the *F*_4_ statistics, no individual exhibits exclusively *NOR* ancestry, although smaller ancestry components were found across most individuals and sites. Consistent with the *F*_4_ statistics, larger proportions of *NOR* ancestry were found in the outlier individuals identified above. We then averaged the computed components and calculated the mean Scandinavian Peninsula ancestry per site (Supp. Fig. 6.2b). Excluding the low-coverage individual from Folkestone, Kent, we find that Scandinavian Peninsula ancestry does not exceed 16% in early medieval England (15.8% in Bude and 12.5% in Lincoln) and is absent in Wolverton, Linton, Hartlepool, and Rookery Hill, and low in Eastry (0.6%) and Worth Matravers (0.1%). Higher proportions between 5% and 10% were found in Ely, West Heslerton, Hatherdene Close, and Dover Buckland. Overall, the Anglo-Saxon Period population of England harbours 5.4% Scandinavian Peninsula ancestry. In contrast, for the preceding Iron Age, we estimate only 0.4% Scandinavian Peninsula ancestry. Notably, with the beginning of the Viking Age, a much higher mean proportion was measured in the ancient individuals, accounting for up to 30.6% of the total ancestry (Supp. Fig. 6.2a). This is consistent with previous analyses of the Viking population of England, indicating that English Viking individuals showed high genetic similarity to Anglo-Saxon period individuals, however, additionally exhibiting excess affinity to the present-day Swedish population[^120^](https://paperpile.com/c/hqnLqQ/spmY). The standard errors for the individual estimates of *NOR* ancestry ranged between 0% and 40.8% (with the mean SEs for the *CNE* component being 9.3% (median 9.1%), for *NOR* component 5.9% (median 3.7%), and for the *WBI* component 6.5% (median 5.9%) in all 759 modelled individuals).

We therefore conclude that there is no association between geographic location and fraction of ancestry from the Scandinavian Peninsula. If Saxons, Angles, and Jutes were meaningful biological categories that remained valid after the migration to England, then they were not correlated with varying degrees of ancestry from the Scandinavian Peninsula.


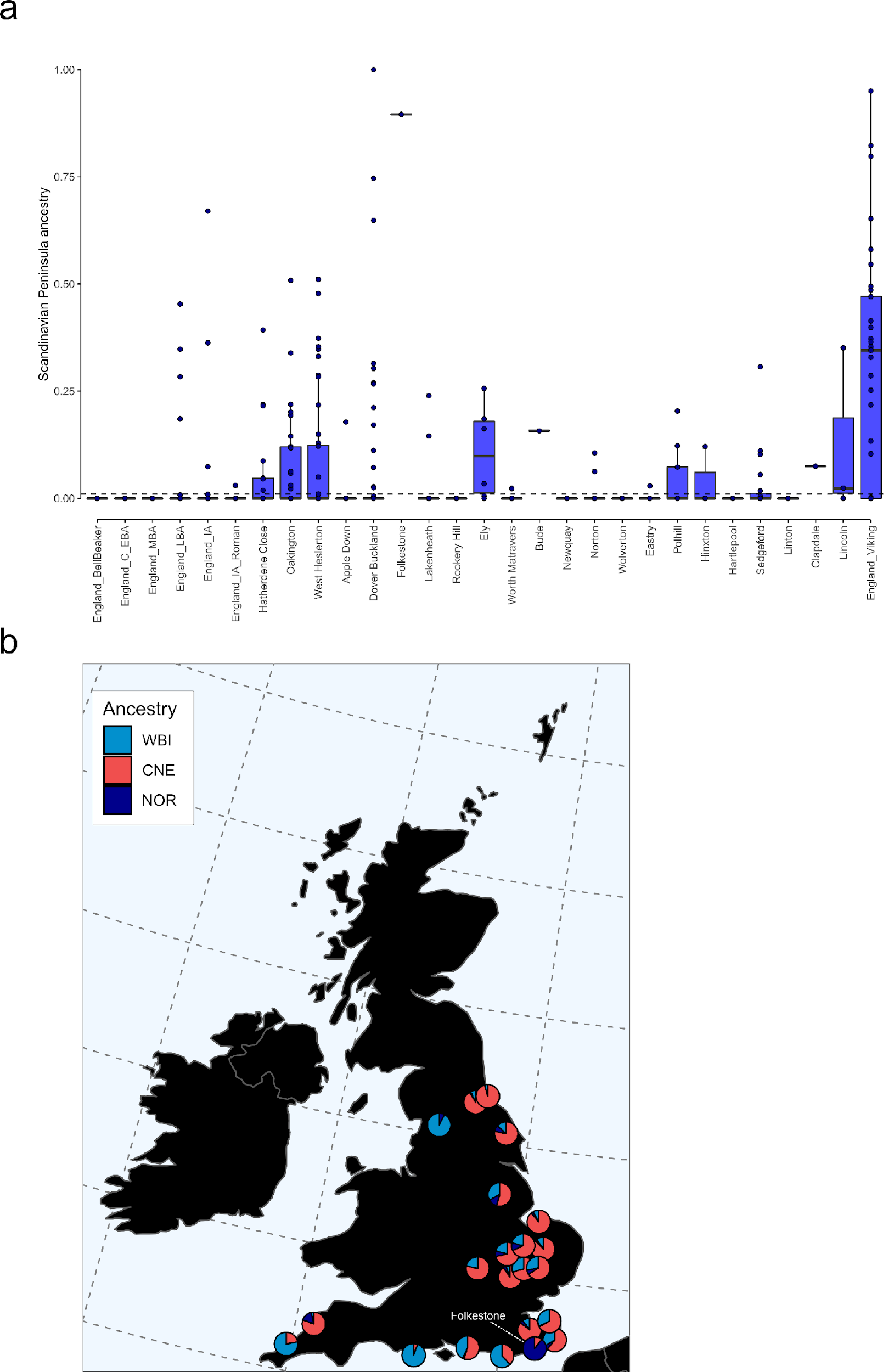


***Supplementary Figure 6.2, Scandinavian Peninsula ancestry in early medieval England.*** *Supervised ADMIXTURE analysis, modelling ancient English individuals as a mixture of CNE (n = 405), WBI (l n = 667), and NOR ( n = 1910) reference populations. a) Shown are NOR estimates as boxplots. Bounds of the Box represent the 25^th^ and 75^th^ Percentile. The centre represents the median. Whiskers represent the smallest value greater than the 25^th^ Percentile minus 1.5 times the interquartile range and largest value less than the 75^th^ Percentile plus 1.5 times the interquartile range, respectively. Outliers present the minimum and maximum values in the data. b) The mean CNE, WBI, and NOR ancestry proportions of 22 early medieval English sites are shown as pie charts. Jitter was induced for enhanced readability. NOR ancestry is colored in dark blue, WBI in lighter blue, and CNE in red.*

## Modelling present-day Britain and Ireland

### qpAdm

We note that our *qpAdm* approaches produce fitting three-way models for all present-day English populations without further ancestry introgression from other parts of Europe. However, recent analyses of present-day individuals indicate at least a minor contribution from the Scandinavian Peninsula, which was introduced by migrations from Sweden and Norway during the Viking period[^56,120,179,180^](https://paperpile.com/c/hqnLqQ/eLLr1+spmY+0Jl9L+qyDmA). While we cannot rule out that this component is too small and/or too undifferentiated from our ancient CNE sources to be detected in the *qpAdm* models, we highlight that supervised ADMIXTURE modelling indicates that at least ~5.4% of the ancestry in early medieval England was related to present-day Norwegians and Swedes (Supp. Fig. 6.2), which suggests that a considerable part of that ancestry was already present in England before the Viking period and subsequent migrations may therefore have contributed less to the modern English gene pool than previously suggested.

However, to find potential signals of post-Anglo-Saxon period introgression from the Scandinavian Peninsula, we further modified our *qpAdm* approach to include four Norwegian Iron Age samples (*Norway_IA*) as fourth source, and adding to the outgroups Norway, Latvia and Saami. The *qpAdm* models obtained are mostly infeasible, suggesting either no additional admixture or too low resolution of our outgroup set (Supp. Table 6.4-6). However, for several regions in eastern and northern England, like Yorkshire and Cumbria, as well as generally in the territory of the Danelaw, we obtained feasible admixture proportions in the range of 1% to 6% (Supp. Fig. 3.3a). For the whole of England, we estimate 1.1% ± 3.4% *Norway_IA* contributions using *England_EMA_CNE* as the source of CNE ancestry (*p* > 0.27)

### Supervised ADMIXTURE

Additionally, we also modelled each of the 1678 modern PoBI individuals as a mixture between CNE, WBI, CWE, and NOR. As in supplementary note 3, this *Norse* metapopulation included 1910 individuals from Sweden and Norway grouped together. We estimate an average of 6% ± 0.2% Norse ancestry in present-day England, with peaks in Cumbria, Northeast England, and East Anglia and lower proportions in western and southwestern England (*e.g.* Cornwall, Sussex, Herefordshire, Forest of Dean)(Supp. Fig. 6.3b, Supp. Table 6.7-8), which is close to previous estimates based on ancient DNA[^120^](https://paperpile.com/c/hqnLqQ/spmY). Correspondingly, the overall fraction of CNE ancestry in England was reduced by inclusion of the fourth source population to 32.7% ± 0.4% (WBI = 36.4% ± 0.4%, CWE = 24.9% ± 0.4%).

Altogether, our analyses suggest that later migration from northern Europe into England during the Viking period did not have a substantial effect on the English population and that only minor ancestry contributions from the Scandinavian Peninsula are present in England, mainly restricted to the northern regions. This finding contrasts with results obtained from modelling non-English British and Irish populations. For example, we find that Orkney received 12.2% ± 0.9% NOR-related or 15.4% ± 4.5% *Norway_IA*-related ancestry and a minor contribution from CNE-like (6.1% ± 0.8%) or *England_EMA_CNE*-like sources (9.5% ± 5.9%). This is highly congruent with previous estimates of Norse ancestry in the population of the Orkney Islands[^56,180^](https://paperpile.com/c/hqnLqQ/eLLr1+qyDmA).

On the other hand, we observe that mainland Scotland was rather influenced by CNE-like ancestry, in agreement with previous Y-chromosome analyses indicating a mainly Danish rather than Norwegian introgression[^157^](https://paperpile.com/c/hqnLqQ/wOVd7) (16.8% ± 1.4% CNE vs. 5.3% ± 0.8% NOR ancestry or 10.1% ± 6.3% *England_EMA_CNE* vs. 5.5% ± 4.8% *Norway_IA* ancestry), with higher proportions of CNE ancestry in the East than in the West (22.9% ± 1.5% vs 8.6% ± 2.0%) (Supp. Table 6.5, 6.6 and 6.8). Taken together, this highlights that our *qpAdm* and supervised ADMIXTURE approaches are generally able to identify excess affinity to populations from the Scandinavian peninsula, supporting our inferences about the magnitude of Norse Viking ancestry contribution to the modern English population.


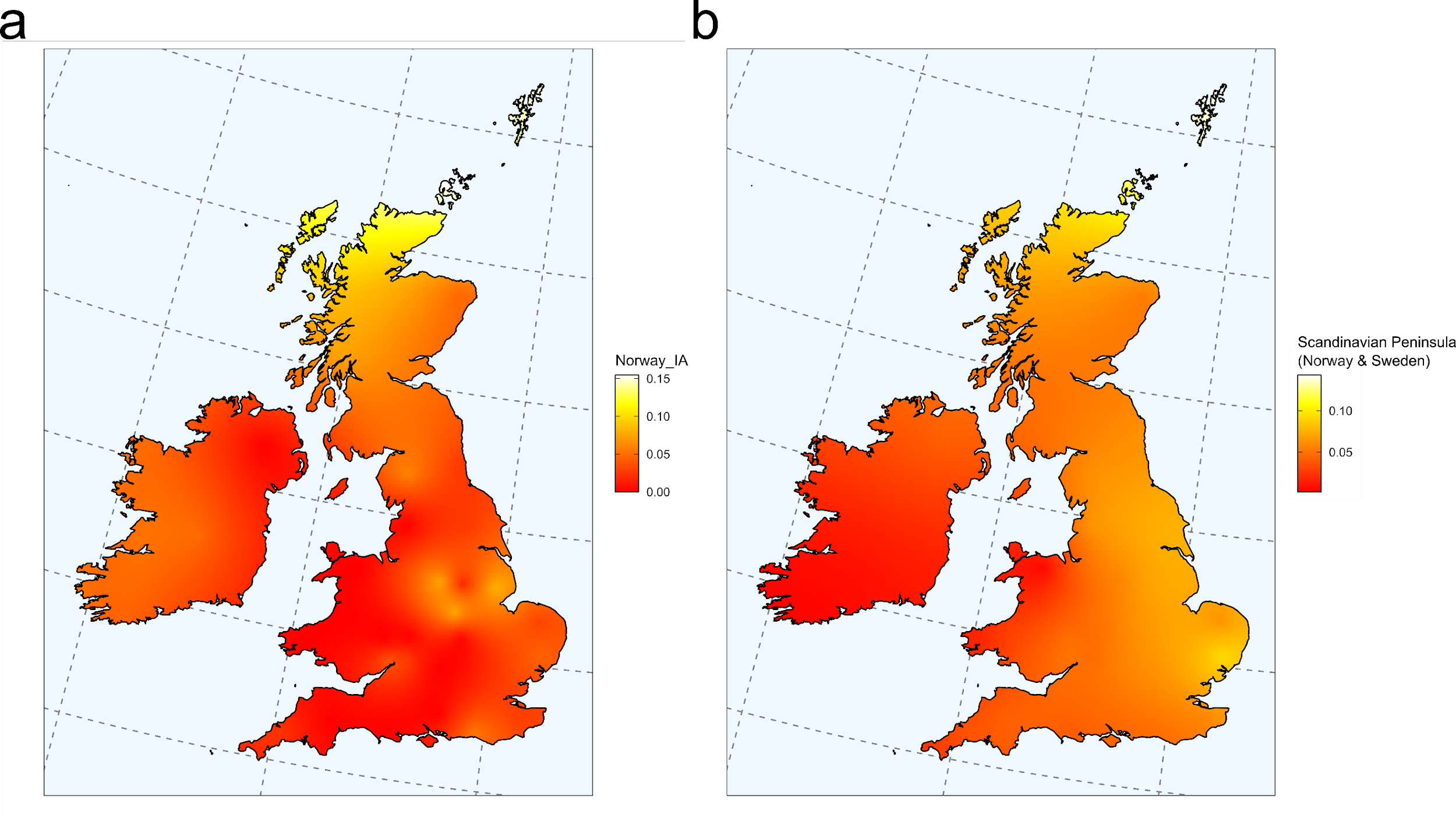


***Supplementary Figure 6.3, Scandinavian Peninsula ancestry in present-day Britain and Ireland.*** *Interpolated maps of Scandinavian Peninsula (Norway IA or present-day Swedish & Norwegian) ancestry across modern populations from Britain and Ireland. a) Using Norway IA and England_EMA_CNE as sources of Norse or CNE ancestry. b) Using NOR (present-day Norway & Sweden) and CNE (present-day Denmark & Northern Germany) as sources of Norse or CNE ancestry.*

#

# Supplementary Note 7 *Association of CNE ancestry with elements of material culture*

Amorim et al. identified distinctive differences in grave furnishings of individuals with predominantly immigrant and local ancestry in the early medieval Langobard-associated cemeteries of Szólád and Collegno in Hungary and Italy. In particular, in both sites, individuals with immigrant ancestry were significantly more often buried with grave goods. We wanted to test if a similar relationships between immigrant ancestry and material culture (Supp. Table 7.1) could be observed in Early Anglo-Saxon Period England.

In order to investigate such a relationship, we applied two different statistical approaches:

1. We classified individuals into either immigrant or local groups based on whether their proportion of CNE ancestry was greater or lower than 50%. We then used this classification in a series of Fisher’s exact tests for their association with material culture.
2. We did not group individuals but included CNE ancestry als continuous variable in a series of (non-parametric) Wilcoxon Rank Sum tests for its association with the presence or absence of material culture.

The rationale behind performing both of these tests, is the fact they assess ancestry from different angles. The Fisher’s test considers ancestry as a proxy for “immigrant” (CNE) vs “local” (WBI) individual family histories. The Wilcoxon Rank test adds nuance and resolution to this view, by using the full spectrum of the ancestry gradient in a continuous fashion, even if the continuous nature of ancestry proportion was arguably less obvious to the lived experience.

Overall, we find consistent results between these two approaches, with notable exception of West Heslerton, where the Wilcoxon test reveals a significant relationship in male graves between CNE ancestry and presence of weapons, which is the only significant such relationship found among sites and regions overall.

## Fisher’s exact test

For approach 1), we categorised all individuals according to their CNE and WBI ancestry estimates into two classes: CNE (>50% CNE ancestry) and WBI (>50% WBI ancestry), and then computed Fisher’s exact tests with respect to the following burial features:

- Presence of grave goods (“Grave goods”)
- For male graves: Presence of grave goods (“Male grave goods”)
- For male graves: Presence of weapons (“Weapons”)
- For female graves: Presence of grave goods (“Female grave goods”)
- For female graves: Presence of brooches (“Brooches”)
- Estimated age (“Age”) (based on three classes <25yo, 25-45yo, >45yo), in which case we used a Fisher-Freeman-Halton test to account for more than two age categories
- Genetic Sex (“Sex”)
- North / South as a broad regional category (“North/South”)

The numbers reported are *p*-values.

### For England as a whole

Grave Goods: .016 significant

Weapons: = 1

Brooches = .012 significant

Age = 0.4

Sex = 0.86

Men grave goods = 0.57

Female Grave goods = 0.001

North/South = 0.01 significant

Each County, Fisher-Freeman-Halton = 0.007 significant

### Regional Patterns

North

Weapons = 0.7

Brooches = 0.9

Grave goods = 0.1

Sex = 0.38

Men grave goods = 0.25

Women Grave goods = 0.004 significant

South

Weapons = 0.53

Brooches = 0.15

Grave goods = 0.44

Sex = 0.65

Men grave goods = 0.74

Women Grave goods = 0.96

East Anglia

Weapons = 1

Brooches = 0.197

Grave goods, Fishers = 0.014 significant

Sex, Fishers = 0.5

Men grave goods, Fishers = 0.34

Women Grave goods, Fisher’s = 0.025 significant

Kent

Weapons, Fisher test = 1

Brooches, Fishers = 0.25

Grave goods, Fishers = 0.283

Sex, Fishers = 0.48

Men grave goods, Fishers = 1

Women Grave goods, Fisher’s = 0.68

### Cemetery Patterns

*Apple Down*

Configuration, Fisher = .048 significant

Weapons, Fisher test = 1

Brooches , Fishers = 0.33

*Dover Buckland*

Weapons Fishers = 0.6

Brooches Fishers = 0.65

Grave goods, Fishers = 1

Genetic Sex, Fishers = 1

Men Grave Goods, Fishers = 1

Women Grave Goods, Fishers = 1

*Hatherdene Close*

Weapons Fishers test = 1

Brooches Fishers = 0.1

Grave goods, Fishers = 0.015 significant

Genetic Sex, Fishers = 1

Men Grave Goods, Fishers = 0.33

Women Grave Goods, Fishers = 0.036 significant

*Oakington*

Weapons Fishers = 0.2

Brooches Fishers = 0.6

Grave goods, Fishers = 1

Genetic Sex, Fishers = 0.6

Men Grave Goods, Fishers = 1

Women Grave Goods, Fishers = 0.45

*West Heslerton*

Weapons Fishers = 0.53

Brooches Fishers = 0.26

Grave goods, Fishers = 0.19

Genetic Sex, Fishers = 1

Men Grave Goods, Fishers = 0.33

Women Grave Goods, Fishers = 0.1

## Wilcoxon Rank Sum test

For approach 2), we tested three different manifestations of material culture: i) (observed) presence against absence of grave goods, ii) (observed) presence of brooches and bractes against their absence in female graves, and iii) (observed) presence of weapons against their absence in male graves.

We tested those on the site level (where applicable due to sample size), in two major regions (Kent and East Anglia), in the northern (Ely, Hatherdene, Lakenheath, Oakington, and West Heslerton) and southern (Apple Down, Dover Buckland, Eastry, and Polhill) sampling range, as well as in all individuals. In those tests, CNE estimates were derived from the three-way admixture model WBI+CNE+CWE as described in Supp. Note 5 (Supp. Fig. 7.1).

We find the following twelve significant (*p* < 0.05) results:

1. In England overall, female graves with brooches/bracteates have more CNE ancestry than female graves without brooches/bracteates.
2. At Hatherdene Close, graves with grave goods have more CNE ancestry than graves without grave goods.
3. At Hatherdene Close, female graves with grave goods have more CNE ancestry than female graves without grave goods.
4. At Apple Down, graves with grave configuration A have more CNE ancestry than graves with grave configuration B.
5. In West Heslerton, male graves with grave goods have more CNE ancestry than male graves without grevae goods.
6. In West Heslerton, male graves with weapons have more CNE ancestry than male graves without weapons.
7. In East Anglia, graves with grave goods have more CNE ancestry than graves without grave goods.
8. In East Anglia, female graves with grave goods have more CNE ancestry than female graves without grave goods.
9. In East Anglia, male graves with grave goods have more CNE ancestry than male graves without grave goods.
10. Looking at the North overall, graves with grave goods have more CNE ancestry than graves without grave goods.
11. Looking at the North overall, female graves with grave goods have more CNE ancestry than female graves without grave goods.
12. Looking at the North overall, male graves with grave goods have more CNE ancestry than male graves without grave goods.

Furthermore, there are four borderline *p*-values (*p* < 0.1):

1. At West Heslerton, graves with grave goods have more CNE than graves without grave goods.
2. At Dover Buckland, female graves with brooches/bracteates have more CNE ancestry than female graves without brooches/bracteates.
3. In Kent, female graves with brooches/bracteates have more CNE than female graves without brooches/bracteates.
4. In the South, female graves with brooches/bracteates have more CNE than female graves without brooches/bracteates.

Finally, we also tested using Kolmogorov-Smirnov-Tests whether (in the whole dataset) CNE distributions differ between: i) graves with and without grave goods, ii) female graves with brooches/bracteates and women graves without brooches/bracteates, iii) male graves with weapons and male graves without weapons. This is, however, for none of the comparisons the case (*p* = 0.5252, 0.165, and 1, respectively).


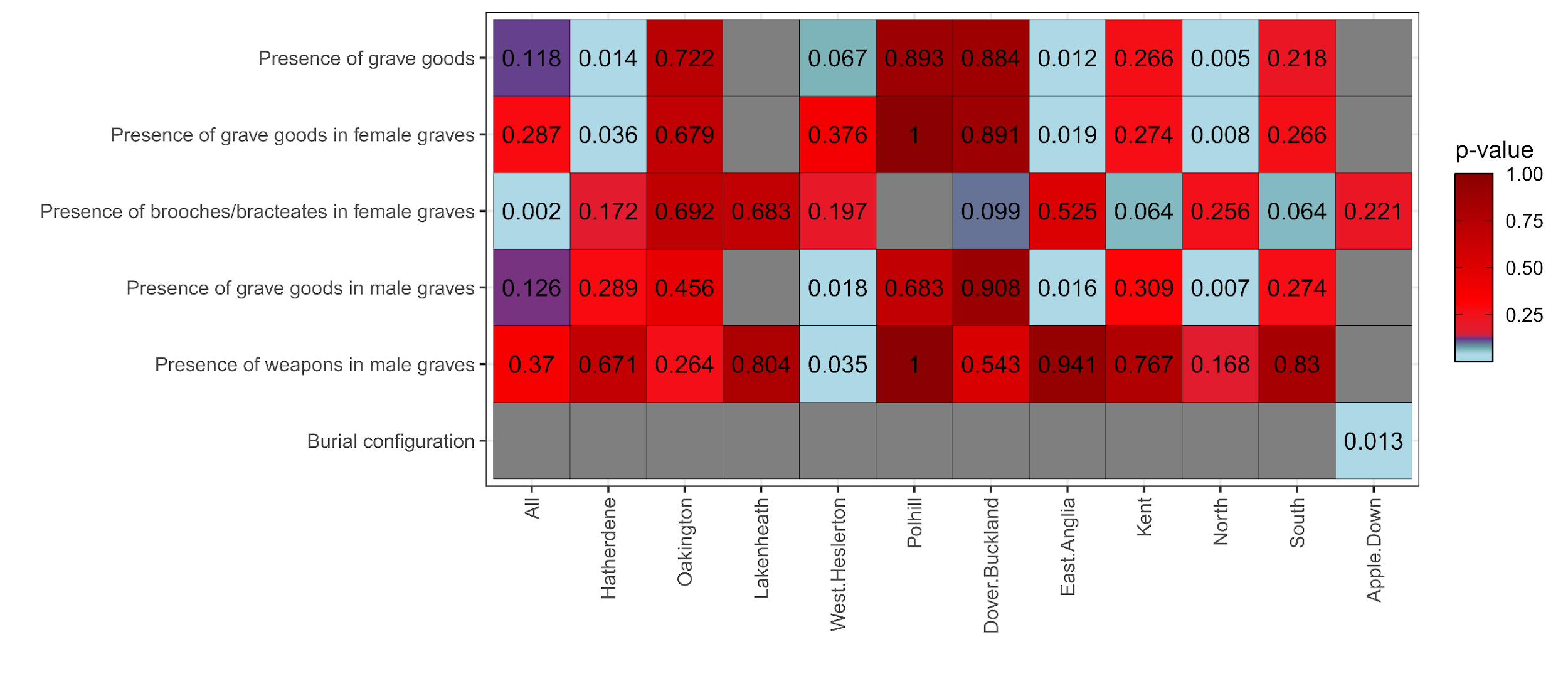


***Supplementary Figure 7.1, Association between material culture and genetic ancestry****. P-values of two-sided Wilcoxon Rank Sum tests across sites and regions are shown.*

# References

1. [Down, A. & Welch, M. Chichester Excavations 7: apple down and the mardens. *Archaeological Journal* **48**, 312–313 (1991).](http://paperpile.com/b/hqnLqQ/ZybRd)

2. [Sayer, D. *Early Anglo-Saxon cemeteries*. (Manchester University Press, 2020).](http://paperpile.com/b/hqnLqQ/G8IaM)

3. [Evison, V. I. *Dover: The Buckland Anglo-Saxon Cemetery*. (Liverpool University Press, 1987).](http://paperpile.com/b/hqnLqQ/Gu1L)

4. [Parfitt, Keith., Anderson, Trevor. & Canterbury Archaeological Trust. *Buckland Anglo-Saxon Cemetery, Dover : excavations 1994*. (Canterbury Archaeological Trust, 2012).](http://paperpile.com/b/hqnLqQ/1dxS)

5. [Stoodley, N. *The spindle and the spear : a critical enquiry into the construction of meaning of gender in the early Anglo-Saxon inhumation burial rite*. vol. 288 (British Archaeology Reports, 1997).](http://paperpile.com/b/hqnLqQ/PzhH)

6. [Lucy, S. Housewives, warriors and slaves? Sex and gender in Anglo-Saxon burials. in *Invisible People and Processes: Writing Gender and Childhood into European Archaeology* (eds. Moore, J. S. E. & Scott, E.) 150–168 (Leicester University Press, 1997).](http://paperpile.com/b/hqnLqQ/rmlm)

7. [Brodrick, H. Fox Holes, Clapdale - a rock shelter. *Yorkshire Ramblers Club Journal* **5**, 112–116 (1924).](http://paperpile.com/b/hqnLqQ/erQAB)

8. [Leach, S. *Going Underground: An anthropological and taphonomic study of human remains from caves and rock shelters in Yorkshire*. (University of Winchester).](http://paperpile.com/b/hqnLqQ/IJ2Jc)

9. [Welch, M. G. Report on excavations of the Anglo-Saxon cemetery at Updown, Eastry, Kent. *Anglo-Saxon Studies in Archaeology and History* **15**, 1–146 (2008).](http://paperpile.com/b/hqnLqQ/bwBUZ)

10. [Ladd, S. & Martin, T. *Roman and Early Anglo-Saxon Cemeteries at Cherry Hinton*. (East Anglian Archaeology).](http://paperpile.com/b/hqnLqQ/s5oNd)

11. [Turk, F. A. Human Remains from Crantock. *Cornish Archaeology* **8**, 98–99 (1969).](http://paperpile.com/b/hqnLqQ/Gy9n6)

12. [Turk, F. A. *On some Human Remains from Early Christian Date from Crantock, Cornwall,*. (1986).](http://paperpile.com/b/hqnLqQ/Lyhrp)

13. [Taylor, A., Duhig, C. & Hines, J. An Anglo-Saxon cemetery at Oakington, Cambridgeshire. *Proceedings of the Cambridge Antiquarian Society* **86**, 57–90 (1997).](http://paperpile.com/b/hqnLqQ/skTi8)

14. [Mortimer, R., Sayer, D. & Wiseman, R. Anglo-Saxon Oakington: A central place on the edge of the Cambridgeshire Fen. in *Life on the Edge: Social, Religious and Political Frontiers in Early Medieval Europe* (eds. Semple, S., Orsini, C. & Mui, S.) vol. 6 305–3016 (Verlag Uwe Krebs, 2017).](http://paperpile.com/b/hqnLqQ/X64wa)

15. [Philp, Brian. & Kent Archaeological Rescue Unit. *The Anglo-Saxon cemetery at Polhill near Sevenoaks, Kent 1964-1986*. (Kent Archaeological Rescue Unit, 2002).](http://paperpile.com/b/hqnLqQ/uGHi7)

16. [Bell, M. Excavations at Bishopstone. *Sussex Archaeological Collections* **115**, (1977).](http://paperpile.com/b/hqnLqQ/ZKupT)

17. [Baldry, R. *et al.* The People of Anglo-Saxon Sedgeford. in *.The Sedgeford Historical and Archaeological Research Project (SHARP) Team. Digging Sedgeford: A people’s archaeology* (eds. Faulkner, N., Rossin, G. & Robinson, K.) 137–166 (Poppyland Publishing, 2014).](http://paperpile.com/b/hqnLqQ/tn7C8)

18. [Kruz, A. Radiocarbon Dating and Bayesian Modelling of the Post-Roman Cemetery. in *Multi-Period Occupation at Football Field, Worth Matravers, Dorset 2006-2011* (ed. Ladle, L.) vol. 643 (2018).](http://paperpile.com/b/hqnLqQ/tBFMI)

19. [Metcalf, D. M. Some further reflections on the volume of the German coinage in the Salian Period (1024-1125). in *Fernhandel und Geldwirschaft. Beiträge zum deutschen Münzwesen in sächsischer Zeit. Ergebnisse des Dannenber-Kolloquiums 1990* (ed. Kluge, B.) (Thorbecke, 1993).](http://paperpile.com/b/hqnLqQ/ZQhag)

20. [Tuin, B. P. Historie. in *Opgravingen aan de Grote Markt Oostzijde, gemeente Groningen Archeologisch onderzoek-deel 1: Algemeen* (ed. Huis in ’t Veld, J. Y.) vols 3300-1 (Raap-Rapport).](http://paperpile.com/b/hqnLqQ/L0Ztk)

21. [Tuin, B. P. Geschiedenis van het Martini-kerhof als begraafplaats. in *Opgravingen aan de Grote Markt Oostzijde, gemeente Groningen Archeologisch onderzoek-deel 4: Martinikerkhof* (ed. Tuin, B. P.) vols 3300-4.](http://paperpile.com/b/hqnLqQ/1G4IX)

22. [Baetsen. Fysisch-antropologisch onderzoek. in *Opgravingen aan de Grote Markt Oostzijde, gemeente Groningen Archeologisch onderzoek-deel 4: Martinikerkhof* (ed. Tuin, B. P.) vols 3300-4 (2018).](http://paperpile.com/b/hqnLqQ/4RbPH)

23. [Kootker, L. M. Isotopenonderzoek menselijke resten Martinikerkhof. in *Opgravingen aan de Grote Markt Oostzijde, gemeente Groningen Archeologisch onderzoek-deel 4: Martinikerkhof* (ed. Tuin, B. P.) vols 3300-4 (2018).](http://paperpile.com/b/hqnLqQ/e8UES)

24. [Hielkema, J. B. Synthese en conclusies. in *‘Dames van Midlum’. Opgraving terprestant Midlum-zuid; N31 Traverse te Harlingen, gemeente Harlingen; archeologisch onderzoek: een opgraving* (ed. Hielkema, J. B.) vol. 3299 (2017).](http://paperpile.com/b/hqnLqQ/hRn6w)

25. [Baetsen, S. Menselijk Botmateriaal. in *‘Dames van Midlum’. Opgraving terprestant Midlum-zuid; N31 Traverse te Harlingen, gemeente Harlingen; archeologisch onderzoek: een opgraving* (ed. Hielkema, J. B.) vol. 3299 (2017).](http://paperpile.com/b/hqnLqQ/13R3k)

26. [Hielkema, J. B. Grondsporen en structuren. in *‘Dames van Midlum’. Opgraving terprestant Midlum-zuid; N31 Traverse te Harlingen, gemeente Harlingen; archeologisch onderzoek: een opgraving* (ed. Hielkema, J. B.) vol. 3299 (2017).](http://paperpile.com/b/hqnLqQ/2b9vP)

27. [Kootker, L. M. Isotopenonderzoek naar de herkomst van zes individuen uit Harlingen. in *‘Dames van Midlum’. Opgraving terprestant Midlum-zuid; N31 Traverse te Harlingen, gemeente Harlingen; archeologisch onderzoek: een opgraving* (ed. Hielkema, J. B.) vol. 3299 (2017).](http://paperpile.com/b/hqnLqQ/XG0p9)

28. [Schmid, P. Das frühmittelalterliche Gräberfeld von Dunum, Kreis Wittmund (Ostfriesland). *Nachrichten aus Niedersachsens Urgeschichte* **36**, 39–74 (1967).](http://paperpile.com/b/hqnLqQ/FlXoC)

29. [Schmid, P. Zum heidnischen und frühchristlichen Bestattungsbrauch auf dem frühmittelalterlichen Gräberfeld von Dunum / Ostfriesland. *Frühmittelalterliche Studien* **3**, 257–275 (1969).](http://paperpile.com/b/hqnLqQ/1kMDv)

30. [Schmid, P. Die Keramik aus dem frühmittelalterlichen Gräberfeld von Dunum, Kreis Wittmund. *Probleme der Küstenforschung im südlichen Nordseegebiet* **9**, 59–76 (1970).](http://paperpile.com/b/hqnLqQ/bjvX4)

31. [Schmid, P. Das frühmittelalterliche Gräberfeld von Dunum, Kreis Wittmund (Ostfriesland) (Grabung 1967-1968). *Neue Ausgrabungen und Forschungen in Niedersachsen* **5**, 40–62 (1970).](http://paperpile.com/b/hqnLqQ/dbi4A)

32. [Schmid, P. Zur Datierung und Gliederung der Grabanlagen von Dunum. *Neue Ausgrabungen und Forschungen in Niedersachsen* **7**, 211–240 (1972).](http://paperpile.com/b/hqnLqQ/eB8OS)

33. [Schmid, P. Friesische Gräberfelder und das Verhältnis ihrer Funde zur Sachkultur im Karolingerreich und in Skandinavien. in *Archäologische und naturwissenschaftliche Untersuchungen an Siedlungen im deutschen Küstengebiet vom 5. Jahrhundert v. Chr. bis zum 11. Jahrhundert n. Chr.* (eds. Kossack, G., Behre, K. E. & Schmid, P.) vol. 1 361–377 (1984).](http://paperpile.com/b/hqnLqQ/mzebk)

34. [Fischer, A., Peek, C. & Siegmüller, A. Feinstrategraphische Untersuchungen an Eisenobjekten des frühmittelalterlichen Gräberfelds von Dunum, Ldkr. Wittmund (Ostfriesland) – Zur Funktion und Deutung organischer Funde und Befunde. *Siedlungs- und Küstenforschung im südlichen Nordseegebiet* **35**, 317–351 (2012).](http://paperpile.com/b/hqnLqQ/7mnhI)

35. [Siegmüller, A. Leichentücher und Federstreuungen. Das frühmittelalterliche Gräberfeld von Dunum, Ostfriesland, als Spiegel politisch-religiöser Wandlungen des 7.-10. Jahrhunderts im Küstenraum. in *Transformations in North-Western Europe (AD 300-1000), Proceedings of the 60th International Sachsen-Symposion 12.-23. September 2009 Maastricht.* (ed. Panhuysen, T. A. S. M.) vol. 3 239–250 (2011).](http://paperpile.com/b/hqnLqQ/zChYj)

36. [Siegmüller, A. & Peel, C. Kleinod und Gebrauchsgegenstand – Nadelröhrchen als Bestandteil des karolingischen Gürtelgehänges. *Archäologisches Korrespondenzblatt* **36**, 445–453 (2006).](http://paperpile.com/b/hqnLqQ/6aRU5)

37. [Siegmüller, A. & Peek, C. Das Bootsgrab aus dem frühmittelalterlichen Gräberfeld von Dunum, Ldkr. Wittmund. *Nachrichten des Marschenrates* **52**, 46–48 (2015).](http://paperpile.com/b/hqnLqQ/q00ad)

38. [Theune, C. Die Perlen von Dunum – Neue Forschungen zur Chonologie des karolingerzeitlichen Gräberfeldes von Dunum, Ldkr. Wittmund. *Probleme der Küstenforschung im südlichen Nordseegebiet* **32**, 243–285 (2008).](http://paperpile.com/b/hqnLqQ/RgQj9)

39. [Tidow, K. & Schmid, P. Frühmittelalterliche Textilfunde aus der Wurt Hessens (Stadt Wilhelmshaven) und dem Gräberfeld von Dunum (Kreis Friesland) und ihre archäologische Bedeutung. *Probleme der Küstenforschung im südlichen Nordseegebiet* **13**, 123–153 (1979).](http://paperpile.com/b/hqnLqQ/9nG37)

40. [Grefen-Peters, S., Peek, C. & Siegmüller, A. Neue Ergebnisse der anthropologischen und archäologischen Forschungen am Gräberfeld von Dunum (Landkreis Wittmund). in *Ostfriesland I Niedersachsenweit. Festschrift für Rolf Bärenfänger.* (ed. Kegler, J.) vol. 87 121–126 (2020).](http://paperpile.com/b/hqnLqQ/hAGdN)

41. [Wahl, J. Beobachtung zur Verbrennung menschlicher Leichname. *Archäologisches Korrespondenzblatt* **11**, 271–279 (1981).](http://paperpile.com/b/hqnLqQ/1hpVp)

42. [Grefen-Peters, S. Die anthropologische Untersuchung ausgewählter Leichenbrände aus dem frühmittelalterlichen Gräberfeld von Dunum, Ldkr. Wittmund. *Siedlungs- und Küstenforschung im südlichen Nordseegebiet* **39**, 273–285 (2016).](http://paperpile.com/b/hqnLqQ/PMaX0)

43. [Grefen-Peters, S. & Hüser, A. Nicht Klunker, sondern Clinker. Kleine Tropfen geben Aufschluss über Leichenverbrennung. *Archäologie in Niedersachsen* **20**, 101–104 (2017).](http://paperpile.com/b/hqnLqQ/8BEum)

44. [Bartelt, U. & Brückner, M. Hiddestorf FStNr. 7. *Fundchronik Niedersachsen 2012. Nachrichten aus Niedersachsens Urgeschichte Beiheft* **17**, 60–61 (2014).](http://paperpile.com/b/hqnLqQ/Lkwvg)

45. [Winger, D., Gerling, C., Bartelt, U. & Ludowici, B. Zwischen Beckum und Alach – das Prunkgrab mit Pferdebestattung von Hemmingen-Hiddestorf am Hellweg. in *Tiere und Tierdarstellungen in der Archäologie. Beiträge zum Kolloquium in Gedenken an Torsten Capelle, 30.–31. Oktober 2015 in Herne.* (eds. Brieske, V., Dickers, A. & Rind, M.) vol. 22 213–232 (2017).](http://paperpile.com/b/hqnLqQ/9TLSP)

46. [Winger, D. Gemeinsam in den Tod. Der Krieger von Hiddestorf und seine Begleiter. in *Saxones. Eine neue Geschichte der alten Sachsen. Begleitband zur Niedersächsischen Landesaustellung 2019. Landesmuseum Hannover 5.4.-18.8. 2019, Braunschweigisches Landesmuseum 22.9.2019 - 2.2.2020* (ed. Ludowici, B.) vol. 7 203–207 (2019).](http://paperpile.com/b/hqnLqQ/n9Uaz)

47. [Ludowici, B. & Winger, D. Fernbeziehung und Selbstzuordnung im Bestattungsbefund: Ein neues Prunkgrab der merowingerzeitlichen Elite am Hellweg von Hemmingen-Hiddestorf, Region Hannover. in *Interacting Barbarians. Contacts, Exchange and Migrations in the First Millennium AD* (eds. Cieslinski, B. & Kontny, B.) vol. 9 (2019).](http://paperpile.com/b/hqnLqQ/bGUNw)

48. [Vogel, V. *Kirche und Gräberfeld des 11. - 13 Jahrhunderts unter dem Rathausmarkt von Schleswig*. (Wachholtz, 1997).](http://paperpile.com/b/hqnLqQ/VgMMY)

49. [Boldsen, J. L., Rasmussen, K. L., Riis, T., Dittmar, M. & Weise, S. Schleswig: Medieval leprosy on the boundary between Germany and Denmark. *Anthropol. Anz.* **70**, 273–287 (2013).](http://paperpile.com/b/hqnLqQ/gnf6T)

50. [Müller, U., Rösch, F. & Schimmer, M. Von Haithabu nach Schleswig. Aktuelle Forschungen zur Gründung einer Metropole zwischen Wikinger- und Hansezeit. *mitt-dgamn* **27**, 26–36 (2014).](http://paperpile.com/b/hqnLqQ/C8xjE)

51. [Carnap-Bornheim, C. von. Die Schleiregion – eine archäologische Landschaft zwischen Nord- und Ostsee. in *Die Schleiregion: Land – Wasser – Geschichte* (eds. Carnap-Bornheim, C. von & Abegg-Wigg, A.) 14–17 (Theiss, 2007).](http://paperpile.com/b/hqnLqQ/IbNtM)

52. [Maixner, B. *Haithabu Fernhandelszentrum zwischen den Welten. Begleitband zur Ausstellung im Wikinger-Museum Haithabu*. (Archäologisches Landesmuseum Schleswig, 2010).](http://paperpile.com/b/hqnLqQ/zSSa7)

53. [Jonsson, K. Burial Rods and Charcoal Graves: New Light on Old Burial Practices. in *Viking and Medieval Scandinavia* (eds. Quinn, J., Brink, S. & Hines, J.) vol. 3 43–73 (2007).](http://paperpile.com/b/hqnLqQ/0JrfH)

54. [Genetic Analysis of Psoriasis Consortium & the Wellcome Trust Case Control Consortium 2 *et al.* A genome-wide association study identifies new psoriasis susceptibility loci and an interaction between HLA-C and ERAP1. *Nat. Genet.* **42**, 985–990 (2010).](http://paperpile.com/b/hqnLqQ/MxYPa)

55. [International Multiple Sclerosis Genetics Consortium *et al.* Genetic risk and a primary role for cell-mediated immune mechanisms in multiple sclerosis. *Nature* **476**, 214–219 (2011).](http://paperpile.com/b/hqnLqQ/XdvpV)

56. [Leslie, S. *et al.* The fine-scale genetic structure of the British population. *Nature* **519**, 309–314 (2015).](http://paperpile.com/b/hqnLqQ/eLLr1)

57. [Price, A. L. *et al.* Long-range LD can confound genome scans in admixed populations. *American journal of human genetics* vol. 83 132–5; author reply 135–9 (2008).](http://paperpile.com/b/hqnLqQ/0fg8B)

58. [Salm, M. P. A. *et al.* The origin, global distribution, and functional impact of the human 8p23 inversion polymorphism. *Genome Res.* **22**, 1144–1153 (2012).](http://paperpile.com/b/hqnLqQ/jJsdH)

59. [Galinsky, K. J., Loh, P.-R., Mallick, S., Patterson, N. J. & Price, A. L. Population Structure of UK Biobank and Ancient Eurasians Reveals Adaptation at Genes Influencing Blood Pressure. *Am. J. Hum. Genet.* **99**, 1130–1139 (2016).](http://paperpile.com/b/hqnLqQ/P78rj)

60. [Anderson, C. A. *et al.* Data quality control in genetic case-control association studies. *Nat. Protoc.* **5**, 1564–1573 (2010).](http://paperpile.com/b/hqnLqQ/tbeMT)

61. [Purcell, S. *et al.* PLINK: a tool set for whole-genome association and population-based linkage analyses. *Am. J. Hum. Genet.* **81**, 559–575 (2007).](http://paperpile.com/b/hqnLqQ/BiJCM)

62. [Behar, D. M. *et al.* The genome-wide structure of the Jewish people. *Nature* **466**, 238–242 (2010).](http://paperpile.com/b/hqnLqQ/6zgL3)

63. [Yunusbayev, B. *et al.* The Caucasus as an asymmetric semipermeable barrier to ancient human migrations. *Mol. Biol. Evol.* **29**, 359–365 (2012).](http://paperpile.com/b/hqnLqQ/tJLVT)

64. [Behar, D. M. *et al.* No evidence from genome-wide data of a Khazar origin for the Ashkenazi Jews. *Hum. Biol.* **85**, 859–900 (2013).](http://paperpile.com/b/hqnLqQ/eNteK)

65. [Genome of the Netherlands Consortium. Whole-genome sequence variation, population structure and demographic history of the Dutch population. *Nat. Genet.* **46**, 818–825 (2014).](http://paperpile.com/b/hqnLqQ/3aPPV)

66. [Kovacevic, L. *et al.* Standing at the gateway to Europe--the genetic structure of Western balkan populations based on autosomal and haploid markers. *PLoS One* **9**, e105090 (2014).](http://paperpile.com/b/hqnLqQ/boRxi)

67. [Kushniarevich, A. *et al.* Genetic Heritage of the Balto-Slavic Speaking Populations: A Synthesis of Autosomal, Mitochondrial and Y-Chromosomal Data. *PLoS One* **10**, e0135820 (2015).](http://paperpile.com/b/hqnLqQ/j8W3L)

68. [Sudmant, P. H. *et al.* An integrated map of structural variation in 2,504 human genomes. *Nature* **526**, 75–81 (2015).](http://paperpile.com/b/hqnLqQ/D6a2V)

69. [1000 Genomes Project Consortium *et al.* A global reference for human genetic variation. *Nature* **526**, 68–74 (2015).](http://paperpile.com/b/hqnLqQ/oRczI)

70. [Pagani, L. *et al.* Genomic analyses inform on migration events during the peopling of Eurasia. *Nature* **538**, 238–242 (2016).](http://paperpile.com/b/hqnLqQ/E47th)

71. [Mallick, S. *et al.* The Simons Genome Diversity Project: 300 genomes from 142 diverse populations. *Nature* **538**, 201–206 (2016).](http://paperpile.com/b/hqnLqQ/fu8UO)

72. [Tamm, E. *et al.* Genome-wide analysis of Corsican population reveals a close affinity with Northern and Central Italy. *Sci. Rep.* **9**, 13581 (2019).](http://paperpile.com/b/hqnLqQ/QtLQE)

73. [Urnikyte, A. *et al.* Patterns of genetic structure and adaptive positive selection in the Lithuanian population from high-density SNP data. *Sci. Rep.* **9**, 9163 (2019).](http://paperpile.com/b/hqnLqQ/RcjO)

74. [Kayser, M. *et al.* Significant genetic differentiation between Poland and Germany follows present-day political borders, as revealed by Y-chromosome analysis. *Hum. Genet.* **117**, 428–443 (2005).](http://paperpile.com/b/hqnLqQ/D9KYI)

75. [Rębała, K. *et al.* Contemporary paternal genetic landscape of Polish and German populations: from early medieval Slavic expansion to post-World War II resettlements. *Eur. J. Hum. Genet.* **21**, 415–422 (2012).](http://paperpile.com/b/hqnLqQ/gWBuy)

76. [Rühlemann, M. C. *et al.* Genome-wide association study in 8,956 German individuals identifies influence of ABO histo-blood groups on gut microbiome. *Nat. Genet.* **53**, 147–155 (2021).](http://paperpile.com/b/hqnLqQ/owf11)

77. [McInnes, L., Healy, J. & Melville, J. UMAP: Uniform Manifold Approximation and Projection for Dimension Reduction. *arXiv [stat.ML]* (2018).](http://paperpile.com/b/hqnLqQ/GhEK2)

78. [Patterson, N., Price, A. L. & Reich, D. Population structure and eigenanalysis. *PLoS Genet.* **2**, e190 (2006).](http://paperpile.com/b/hqnLqQ/LPCGB)

79. [Lao, O. *et al.* Correlation between genetic and geographic structure in Europe. *Curr. Biol.* **18**, 1241–1248 (2008).](http://paperpile.com/b/hqnLqQ/lvyLC)

80. [Novembre, J. *et al.* Genes mirror geography within Europe. *Nature* **456**, 274–274 (2008).](http://paperpile.com/b/hqnLqQ/e38C6)

81. [Patterson, N. *et al.* Large-scale migration into Britain during the Middle to Late Bronze Age. *Nature* **601**, 588–594 (2021).](http://paperpile.com/b/hqnLqQ/3Th9)

82. [Allentoft, M. E. *et al.* Population genomics of Bronze Age Eurasia. *Nature* **522**, 167–172 (2015).](http://paperpile.com/b/hqnLqQ/L1lCW)

83. [Amorim, C. E. G. *et al.* Understanding 6th-century barbarian social organization and migration through paleogenomics. *Nat. Commun.* **9**, 3547 (2018).](http://paperpile.com/b/hqnLqQ/X7M1r)

84. [Antonio, M. L. *et al.* Ancient Rome: A genetic crossroads of Europe and the Mediterranean. *Science* **366**, 708–714 (2019).](http://paperpile.com/b/hqnLqQ/0aHzs)

85. [Brace, S. *et al.* Ancient genomes indicate population replacement in Early Neolithic Britain. *Nature Ecology & Evolution* **3**, 765–771 (2019).](http://paperpile.com/b/hqnLqQ/VTvWq)

86. [Brunel, S. *et al.* Ancient genomes from present-day France unveil 7,000 years of its demographic history. *Proc. Natl. Acad. Sci. U. S. A.* **117**, 12791–12798 (2020).](http://paperpile.com/b/hqnLqQ/CnCIo)

87. [Cassidy, L. M. *et al.* Neolithic and Bronze Age migration to Ireland and establishment of the insular Atlantic genome. *Proc. Natl. Acad. Sci. U. S. A.* **113**, 368–373 (2016).](http://paperpile.com/b/hqnLqQ/r5iW6)

88. [Cassidy, L. M. *et al.* A dynastic elite in monumental Neolithic society. *Nature* **582**, 384–388 (2020).](http://paperpile.com/b/hqnLqQ/VGqUa)

89. [Coutinho, A. *et al.* The Neolithic Pitted Ware culture foragers were culturally but not genetically influenced by the Battle Axe culture herders. *Am. J. Phys. Anthropol.* **172**, 638–649 (2020).](http://paperpile.com/b/hqnLqQ/Iw8qF)

90. [Damgaard, P. de B. *et al.* 137 ancient human genomes from across the Eurasian steppes. *Nature* **557**, 369–374 (2018).](http://paperpile.com/b/hqnLqQ/CxUY7)

91. [de Barros Damgaard, P. *et al.* The first horse herders and the impact of early Bronze Age steppe expansions into Asia. *Science* **360**, (2018).](http://paperpile.com/b/hqnLqQ/MyIFY)

92. [Ebenesersdóttir, S. S. *et al.* Ancient genomes from Iceland reveal the making of a human population. *Science* **360**, 1028–1032 (2018).](http://paperpile.com/b/hqnLqQ/9O9CJ)

93. [Fernandes, D. M. *et al.* The spread of steppe and Iranian-related ancestry in the islands of the western Mediterranean. *Nature Ecology & Evolution* **4**, 334–345 (2020).](http://paperpile.com/b/hqnLqQ/I4Koe)

94. [Fernandes, D. M. *et al.* A genomic Neolithic time transect of hunter-farmer admixture in central Poland. *Sci. Rep.* **8**, 1–11 (2018).](http://paperpile.com/b/hqnLqQ/eGMso)

95. [Fu, Q. *et al.* The genetic history of Ice Age Europe. *Nature* **534**, 200–205 (2016).](http://paperpile.com/b/hqnLqQ/6OVBv)

96. [Furtwängler, A. *et al.* Ancient genomes reveal social and genetic structure of Late Neolithic Switzerland. *Nat. Commun.* **11**, 1–11 (2020).](http://paperpile.com/b/hqnLqQ/TA8vQ)

97. [Gamba, C. *et al.* Genome flux and stasis in a five millennium transect of European prehistory. *Nat. Commun.* **5**, 1–9 (2014).](http://paperpile.com/b/hqnLqQ/gpuQm)

98. [González-Fortes, G. *et al.* Paleogenomic Evidence for Multi-generational Mixing between Neolithic Farmers and Mesolithic Hunter-Gatherers in the Lower Danube Basin. *Curr. Biol.* **27**, 1801–1810.e10 (2017).](http://paperpile.com/b/hqnLqQ/whx7F)

99. [González Fortes, G. & Paijmans, J. L. A. Whole-Genome Capture of Ancient DNA Using Homemade Baits. *Methods Mol. Biol.* **1963**, 93–105 (2019).](http://paperpile.com/b/hqnLqQ/rN0Dy)

100. [Günther, T. *et al.* Population genomics of Mesolithic Scandinavia: Investigating early postglacial migration routes and high-latitude adaptation. *PLoS Biol.* **16**, e2003703 (2018).](http://paperpile.com/b/hqnLqQ/B5zdX)

101. [Günther, T. *et al.* Ancient genomes link early farmers from Atapuerca in Spain to modern-day Basques. *Proc. Natl. Acad. Sci. U. S. A.* **112**, 11917–11922 (2015).](http://paperpile.com/b/hqnLqQ/n3TTx)

102. [Haber, M. *et al.* A Transient Pulse of Genetic Admixture from the Crusaders in the Near East Identified from Ancient Genome Sequences. *Am. J. Hum. Genet.* **104**, 977–984 (2019).](http://paperpile.com/b/hqnLqQ/5qba3)

103. [Haber, M. *et al.* A Genetic History of the Near East from an aDNA Time Course Sampling Eight Points in the Past 4,000 Years. *Am. J. Hum. Genet.* **107**, 149–157 (2020).](http://paperpile.com/b/hqnLqQ/1MKZc)

104. [Harney, É. *et al.* A minimally destructive protocol for DNA extraction from ancient teeth. *Genome Res.* (2021) doi:](http://paperpile.com/b/hqnLqQ/HE65i)[10.1101/gr.267534.120](http://dx.doi.org/10.1101/gr.267534.120)[.](http://paperpile.com/b/hqnLqQ/HE65i)

105. [Hofmanová, Z. *et al.* Early farmers from across Europe directly descended from Neolithic Aegeans. *Proc. Natl. Acad. Sci. U. S. A.* **113**, 6886–6891 (2016).](http://paperpile.com/b/hqnLqQ/RRCNM)

106. [Järve, M. *et al.* Shifts in the Genetic Landscape of the Western Eurasian Steppe Associated with the Beginning and End of the Scythian Dominance. *Curr. Biol.* **29**, 2430–2441.e10 (2019).](http://paperpile.com/b/hqnLqQ/zR5Kn)

107. [Jones, E. R. *et al.* Upper Palaeolithic genomes reveal deep roots of modern Eurasians. *Nat. Commun.* **6**, 1–8 (2015).](http://paperpile.com/b/hqnLqQ/Ik8SB)

108. [Jones, E. R. *et al.* The Neolithic Transition in the Baltic Was Not Driven by Admixture with Early European Farmers. *Curr. Biol.* **27**, 576–582 (2017).](http://paperpile.com/b/hqnLqQ/yqoEm)

109. [Kılınç, G. M. *et al.* The Demographic Development of the First Farmers in Anatolia. *Curr. Biol.* **26**, 2659–2666 (2016).](http://paperpile.com/b/hqnLqQ/cbdfo)

110. [Krzewińska, M. *et al.* Genomic and Strontium Isotope Variation Reveal Immigration Patterns in a Viking Age Town. *Curr. Biol.* **28**, 2730–2738.e10 (2018).](http://paperpile.com/b/hqnLqQ/knSCH)

111. [Krzewińska, M. *et al.* Ancient genomes suggest the eastern Pontic-Caspian steppe as the source of western Iron Age nomads. *Science Advances* **4**, eaat4457 (2018).](http://paperpile.com/b/hqnLqQ/55V1D)

112. [Lamnidis, T. C. *et al.* Ancient Fennoscandian genomes reveal origin and spread of Siberian ancestry in Europe. *Nat. Commun.* **9**, 5018 (2018).](http://paperpile.com/b/hqnLqQ/L7WJT)

113. [Lazaridis, I. *et al.* Ancient human genomes suggest three ancestral populations for present-day Europeans. *Nature* **513**, 409–413 (2014).](http://paperpile.com/b/hqnLqQ/DEho3)

114. [Lazaridis, I. *et al.* Genomic insights into the origin of farming in the ancient Near East. *Nature* **536**, 419–424 (2016).](http://paperpile.com/b/hqnLqQ/AdxKv)

115. [Lazaridis, I. *et al.* Genetic origins of the Minoans and Mycenaeans. *Nature* **548**, 214–218 (2017).](http://paperpile.com/b/hqnLqQ/4qN2w)

116. [Linderholm, A. *et al.* Corded Ware cultural complexity uncovered using genomic and isotopic analysis from south-eastern Poland. *Sci. Rep.* **10**, 1–13 (2020).](http://paperpile.com/b/hqnLqQ/gMorQ)

117. [Lipson, M. *et al.* Parallel palaeogenomic transects reveal complex genetic history of early European farmers. *Nature* **551**, 368–372 (2017).](http://paperpile.com/b/hqnLqQ/7F4ac)

118. [Malmström, H. *et al.* The genomic ancestry of the Scandinavian Battle Axe Culture people and their relation to the broader Corded Ware horizon. *Proceedings of the Royal Society B: Biological Sciences* **286**, 20191528 (2019).](http://paperpile.com/b/hqnLqQ/IJm1H)

119. [Marcus, J. H. *et al.* Genetic history from the Middle Neolithic to present on the Mediterranean island of Sardinia. *Nat. Commun.* **11**, 1–14 (2020).](http://paperpile.com/b/hqnLqQ/aElE1)

120. [Margaryan, A. *et al.* Population genomics of the Viking world. *Nature* **585**, 390–396 (2020).](http://paperpile.com/b/hqnLqQ/spmY)

121. [Martiniano, R. *et al.* Genomic signals of migration and continuity in Britain before the Anglo-Saxons. *Nat. Commun.* **7**, 10326 (2016).](http://paperpile.com/b/hqnLqQ/jnKBm)

122. [Martiniano, R. *et al.* The population genomics of archaeological transition in west Iberia: Investigation of ancient substructure using imputation and haplotype-based methods. *PLoS Genet.* **13**, e1006852 (2017).](http://paperpile.com/b/hqnLqQ/ZjbXz)

123. [Mathieson, I. *et al.* Genome-wide patterns of selection in 230 ancient Eurasians. *Nature* **528**, 499–503 (2015).](http://paperpile.com/b/hqnLqQ/8E2G5)

124. [Mathieson, I. *et al.* The genomic history of southeastern Europe. *Nature* **555**, 197–203 (2018).](http://paperpile.com/b/hqnLqQ/2oozR)

125. [Mittnik, A. *et al.* The genetic prehistory of the Baltic Sea region. *Nat. Commun.* **9**, 442 (2018).](http://paperpile.com/b/hqnLqQ/1SkHO)

126. [Mittnik, A. *et al.* Kinship-based social inequality in Bronze Age Europe. *Science* **366**, 731–734 (2019).](http://paperpile.com/b/hqnLqQ/e10z3)

127. [Narasimhan, V. M. *et al.* The formation of human populations in South and Central Asia. *Science* **365**, (2019).](http://paperpile.com/b/hqnLqQ/WPSxx)

128. [Nikitin, A. G. *et al.* Interactions between earliest Linearbandkeramik farmers and central European hunter gatherers at the dawn of European Neolithization. *Sci. Rep.* **9**, 1–10 (2019).](http://paperpile.com/b/hqnLqQ/QVLVp)

129. [Novak, M. *et al.* Genome-wide analysis of nearly all the victims of a 6200 year old massacre. *PLoS One* **16**, e0247332 (2021).](http://paperpile.com/b/hqnLqQ/WGuuL)

130. [Olalde, I. *et al.* The Beaker phenomenon and the genomic transformation of northwest Europe. *Nature* **555**, 190–196 (2018).](http://paperpile.com/b/hqnLqQ/5danp)

131. [Olalde, I. *et al.* The genomic history of the Iberian Peninsula over the past 8000 years. *Science* **363**, 1230–1234 (2019).](http://paperpile.com/b/hqnLqQ/tfw6T)

132. [Raghavan, M. *et al.* Upper Palaeolithic Siberian genome reveals dual ancestry of Native Americans. *Nature* **505**, 87–91 (2014).](http://paperpile.com/b/hqnLqQ/veAaL)

133. [Rivollat, M. *et al.* Ancient genome-wide DNA from France highlights the complexity of interactions between Mesolithic hunter-gatherers and Neolithic farmers. *Science Advances* **6**, eaaz5344 (2020).](http://paperpile.com/b/hqnLqQ/eyTW6)

134. [Saag, L. *et al.* Extensive Farming in Estonia Started through a Sex-Biased Migration from the Steppe. *Curr. Biol.* **27**, 2185–2193.e6 (2017).](http://paperpile.com/b/hqnLqQ/0gmAb)

135. [Saag, L. *et al.* The Arrival of Siberian Ancestry Connecting the Eastern Baltic to Uralic Speakers further East. *Curr. Biol.* **29**, 1701–1711.e16 (2019).](http://paperpile.com/b/hqnLqQ/3Ms3X)

136. [Sánchez-Quinto, F. *et al.* Megalithic tombs in western and northern Neolithic Europe were linked to a kindred society. *Proc. Natl. Acad. Sci. U. S. A.* **116**, 9469–9474 (2019).](http://paperpile.com/b/hqnLqQ/kmNjU)

137. [Schiffels, S. *et al.* Iron Age and Anglo-Saxon genomes from East England reveal British migration history. *Nat. Commun.* **7**, 10408 (2016).](http://paperpile.com/b/hqnLqQ/I6Awn)

138. [Schroeder, H. *et al.* Unraveling ancestry, kinship, and violence in a Late Neolithic mass grave. *Proc. Natl. Acad. Sci. U. S. A.* **116**, 10705–10710 (2019).](http://paperpile.com/b/hqnLqQ/2YcIe)

139. [Sikora, M. *et al.* The population history of northeastern Siberia since the Pleistocene. *Nature* **570**, 182–188 (2019).](http://paperpile.com/b/hqnLqQ/ZNX1g)

140. [Skoglund, P. *et al.* Separating endogenous ancient DNA from modern day contamination in a Siberian Neandertal. *Proc. Natl. Acad. Sci. U. S. A.* **111**, 2229–2234 (2014).](http://paperpile.com/b/hqnLqQ/cDAYL)

141. [O’Sullivan, N. *et al.* Ancient genome-wide analyses infer kinship structure in an Early Medieval Alemannic graveyard. *Sci Adv* **4**, eaao1262 (2018).](http://paperpile.com/b/hqnLqQ/Ag6wX)

142. [Valdiosera, C. *et al.* Four millennia of Iberian biomolecular prehistory illustrate the impact of prehistoric migrations at the far end of Eurasia. *Proc. Natl. Acad. Sci. U. S. A.* **115**, 3428–3433 (2018).](http://paperpile.com/b/hqnLqQ/HNlDt)

143. [Veeramah, K. R. *et al.* Population genomic analysis of elongated skulls reveals extensive female-biased immigration in Early Medieval Bavaria. *Proc. Natl. Acad. Sci. U. S. A.* **115**, 3494–3499 (2018).](http://paperpile.com/b/hqnLqQ/Gwgbe)

144. [Villalba-Mouco, V. *et al.* Survival of Late Pleistocene Hunter-Gatherer Ancestry in the Iberian Peninsula. *Curr. Biol.* **29**, 1169–1177.e7 (2019).](http://paperpile.com/b/hqnLqQ/pPRlO)

145. [Wang, C.-C. *et al.* Ancient human genome-wide data from a 3000-year interval in the Caucasus corresponds with eco-geographic regions. *Nat. Commun.* **10**, 1–13 (2019).](http://paperpile.com/b/hqnLqQ/2lYj1)

146. [Semino, O. *et al.* Origin, diffusion, and differentiation of Y-chromosome haplogroups E and J: inferences on the neolithization of Europe and later migratory events in the Mediterranean area. *Am. J. Hum. Genet.* **74**, 1023–1034 (2004).](http://paperpile.com/b/hqnLqQ/jWTHJ)

147. [Lipatov, M., Sanjeev, K., Patro, R. & Veeramah, K. R. Maximum Likelihood Estimation of Biological Relatedness from Low Coverage Sequencing Data. *bioRxiv* 023374 (2015) doi:](http://paperpile.com/b/hqnLqQ/h5S9S)[10.1101/023374](http://dx.doi.org/10.1101/023374)[.](http://paperpile.com/b/hqnLqQ/h5S9S)

148. [Lall, G. M. *et al.* Subdividing Y-chromosome haplogroup R1a1 reveals Norse Viking dispersal lineages in Britain. *Eur. J. Hum. Genet.* **29**, 512–523 (2020).](http://paperpile.com/b/hqnLqQ/6hnh9)

149. [Ingman, M. & Gyllensten, U. A recent genetic link between Sami and the Volga-Ural region of Russia. *Eur. J. Hum. Genet.* **15**, 115–120 (2006).](http://paperpile.com/b/hqnLqQ/n85ZY)

150. [Rootsi, S. *et al.* Phylogeography of Y-chromosome haplogroup I reveals distinct domains of prehistoric gene flow in europe. *Am. J. Hum. Genet.* **75**, 128–137 (2004).](http://paperpile.com/b/hqnLqQ/gzbxt)

151. [Myres, N. M. *et al.* A major Y-chromosome haplogroup R1b Holocene era founder effect in Central and Western Europe. *Eur. J. Hum. Genet.* **19**, 95–101 (2010).](http://paperpile.com/b/hqnLqQ/ewRM1)

152. [Moore, L. T., McEvoy, B., Cape, E., Simms, K. & Bradley, D. G. A Y-chromosome signature of hegemony in Gaelic Ireland. *Am. J. Hum. Genet.* **78**, 334–338 (2006).](http://paperpile.com/b/hqnLqQ/71w4f)

153. [Busby, G. B. J. *et al.* The peopling of Europe and the cautionary tale of Y chromosome lineage R-M269. *Proceedings of the Royal Society B: Biological Sciences* **279**, 884–892 (2012).](http://paperpile.com/b/hqnLqQ/oRaH6)

154. [Altena, E. *et al.* The Dutch Y-chromosomal landscape. *Eur. J. Hum. Genet.* **28**, 287–299 (2019).](http://paperpile.com/b/hqnLqQ/Ok2Cw)

155. [Cruciani, F. *et al.* Strong intra- and inter-continental differentiation revealed by Y chromosome SNPs M269, U106 and U152. *Forensic Sci. Int. Genet.* **5**, e49–52 (2011).](http://paperpile.com/b/hqnLqQ/PsVeU)

156. [Weale, M. E., Weiss, D. A., Jager, R. F., Bradman, N. & Thomas, M. G. Y chromosome evidence for Anglo-Saxon mass migration. *Mol. Biol. Evol.* **19**, 1008–1021 (2002).](http://paperpile.com/b/hqnLqQ/9PRMH)

157. [Capelli, C. *et al.* A Y chromosome census of the British Isles. *Curr. Biol.* **13**, 979–984 (2003).](http://paperpile.com/b/hqnLqQ/wOVd7)

158. [Karlsson, A. O., Wallerström, T., Götherström, A. & Holmlund, G. Y-chromosome diversity in Sweden - a long-time perspective. *Eur. J. Hum. Genet.* **14**, 963–970 (2006).](http://paperpile.com/b/hqnLqQ/oGgkh)

159. [Lappalainen, T. *et al.* Population structure in contemporary Sweden--a Y-chromosomal and mitochondrial DNA analysis. *Ann. Hum. Genet.* **73**, 61–73 (2009).](http://paperpile.com/b/hqnLqQ/FqI9e)

160. [Dulias, K. *et al.* Ancient DNA at the edge of the world: Continental immigration and the persistence of Neolithic male lineages in Bronze Age Orkney. *Proceedings of the National Academy of Sciences* **119**, e2108001119 (2022).](http://paperpile.com/b/hqnLqQ/DlY7)

161. [Underhill, P. A. *et al.* The phylogenetic and geographic structure of Y-chromosome haplogroup R1a. *Eur. J. Hum. Genet.* **23**, 124–131 (2014).](http://paperpile.com/b/hqnLqQ/MNI8E)

162. [Wilson, J. F. *et al.* Genetic evidence for different male and female roles during cultural transitions in the British Isles. *Proc. Natl. Acad. Sci. U. S. A.* **98**, 5078–5083 (2001).](http://paperpile.com/b/hqnLqQ/f2Rv7)

163. [Battaglia, V. *et al.* Y-chromosomal evidence of the cultural diffusion of agriculture in Southeast Europe. *Eur. J. Hum. Genet.* **17**, 820–830 (2009).](http://paperpile.com/b/hqnLqQ/eVyLN)

164. [Mendez, F. L. *et al.* Increased resolution of Y chromosome haplogroup T defines relationships among populations of the Near East, Europe, and Africa. *Hum. Biol.* **83**, 39–53 (2011).](http://paperpile.com/b/hqnLqQ/mViEN)

165. [Sanchez, J. J., Børsting, C., Hernandez, A., Mengel-Jørgensen, J. & Morling, N. Y chromosome SNP haplogroups in Danes, Greenlanders and Somalis. *Int. Congr. Ser.* **1261**, 347–349 (2004).](http://paperpile.com/b/hqnLqQ/wv8hC)

166. [Brown, L. D., Tony Cai, T. & DasGupta, A. Interval Estimation for a Binomial Proportion. *SSO Schweiz. Monatsschr. Zahnheilkd.* **16**, 101–133 (2001).](http://paperpile.com/b/hqnLqQ/9rSCq)

167. [Agresti, A. & Coull, B. A. Approximate Is Better than ‘Exact’ for Interval Estimation of Binomial Proportions. *Am. Stat.* **52**, 119–126 (1998).](http://paperpile.com/b/hqnLqQ/zzrMr)

168. [Goldberg, A., Günther, T., Rosenberg, N. A. & Jakobsson, M. Ancient X chromosomes reveal contrasting sex bias in Neolithic and Bronze Age Eurasian migrations. *Proc. Natl. Acad. Sci. U. S. A.* **114**, 2657–2662 (2017).](http://paperpile.com/b/hqnLqQ/AnR16)

169. [Keinan, A., Mullikin, J. C., Patterson, N. & Reich, D. Accelerated genetic drift on chromosome X during the human dispersal out of Africa. *Nat. Genet.* **41**, 66–70 (2009).](http://paperpile.com/b/hqnLqQ/m3c0y)

170. [Waldman, Y. Y. *et al.* The Genetics of Bene Israel from India Reveals Both Substantial Jewish and Indian Ancestry. *PLoS One* **11**, e0152056 (2016).](http://paperpile.com/b/hqnLqQ/0uPIW)

171. [Haak, W. *et al.* Massive migration from the steppe was a source for Indo-European languages in Europe. *Nature* **522**, 207–211 (2015).](http://paperpile.com/b/hqnLqQ/eK1xv)

172. [Alexander, D. H., Novembre, J. & Lange, K. Fast model-based estimation of ancestry in unrelated individuals. *Genome Res.* **19**, 1655–1664 (2009).](http://paperpile.com/b/hqnLqQ/TNMqU)

173. [Shringarpure, S. S., Bustamante, C. D., Lange, K. & Alexander, D. H. Efficient analysis of large datasets and sex bias with ADMIXTURE. *BMC Bioinformatics* **17**, 218 (2016).](http://paperpile.com/b/hqnLqQ/VgyN1)

174. [Patterson, N. *et al.* Ancient admixture in human history. *Genetics* **192**, 1065–1093 (2012).](http://paperpile.com/b/hqnLqQ/5aYUV)

175. [Reich, D. *et al.* Reconstructing Native American population history. *Nature* **488**, 370–374 (2012).](http://paperpile.com/b/hqnLqQ/egNye)

176. [Benjamini, Y. & Hochberg, Y. Controlling the False Discovery Rate: A Practical and Powerful Approach to Multiple Testing. *J. R. Stat. Soc. Series B Stat. Methodol.* **57**, 289–300 (1995).](http://paperpile.com/b/hqnLqQ/g6Vrb)

177. [Athanasiadis, G. *et al.* Nationwide Genomic Study in Denmark Reveals Remarkable Population Homogeneity. *Genetics* **204**, 711–722 (2016).](http://paperpile.com/b/hqnLqQ/4dRfU)

178. [Byrne, R. P. *et al.* Insular Celtic population structure and genomic footprints of migration. *PLoS Genet.* **14**, e1007152 (2018).](http://paperpile.com/b/hqnLqQ/2b2nD)

179. [Gilbert, E. *et al.* The Irish DNA Atlas: Revealing Fine-Scale Population Structure and History within Ireland. *Sci. Rep.* **7**, 17199 (2017).](http://paperpile.com/b/hqnLqQ/0Jl9L)

180. [Gilbert, E. *et al.* The genetic landscape of Scotland and the Isles. *Proc. Natl. Acad. Sci. U. S. A.* **116**, 19064–19070 (2019).](http://paperpile.com/b/hqnLqQ/qyDmA)

181. [Töpf, A. L., Gilbert, M. T. P., Dumbacher, J. P. & Hoelzel, A. R. Tracing the phylogeography of human populations in Britain based on 4th-11th century mtDNA genotypes. *Mol. Biol. Evol.* **23**, 152–161 (2006).](http://paperpile.com/b/hqnLqQ/vtUXz)

182. [Härke, H. Anglo-Saxon Immigration and Ethnogenesis. *Mediev. Archaeol.* **55**, 1–28 (2011).](http://paperpile.com/b/hqnLqQ/HD1jA)
