# Supplementary material for: The Anglo-Saxon migration and the formation of the early English gene pool
Source: Nature. 2022 Sep 21;610(7930):112–9. doi: 10.1038/s41586-022-05247-2 (PMC9534755; doi:10.1038/s41586-022-05247-2)
Supplement: Supplementary file 2 — Reporting Summary [file 41586_2022_5247_MOESM2_ESM.pdf]

nature portfolio

Corresponding author(s): Stephan Schiffels

Last updated by author(s): Jul 22, 2022

Reporting Summary

Nature Portfolio wishes to improve the reproducibility of the work that we publish. This form provides structure for consistency and transparency in reporting. For further information on Nature Portfolio policies, see our Editorial Policies and the Editorial Policy Checklist.

Statistics

For all statistical analyses, confirm that the following items are present in the figure legend, table legend, main text, or Methods section.

n/a	Confirmed	
☐	☒	The exact sample size (*n*) for each experimental group/condition, given as a discrete number and unit of measurement
☒	☐	A statement on whether measurements were taken from distinct samples or whether the same sample was measured repeatedly
☐	☒	The statistical test(s) used AND whether they are one- or two-sided<br>*Only common tests should be described solely by name; describe more complex techniques in the Methods section.*
☒	☐	A description of all covariates tested
☐	☒	A description of any assumptions or corrections, such as tests of normality and adjustment for multiple comparisons
☐	☒	A full description of the statistical parameters including central tendency (e.g. means) or other basic estimates (e.g. regression coefficient) AND variation (e.g. standard deviation) or associated estimates of uncertainty (e.g. confidence intervals)
☐	☒	For null hypothesis testing, the test statistic (e.g. F, t, r) with confidence intervals, effect sizes, degrees of freedom and P value noted<br>*Give P values as exact values whenever suitable.*
☐	☒	For Bayesian analysis, information on the choice of priors and Markov chain Monte Carlo settings
☐	☒	For hierarchical and complex designs, identification of the appropriate level for tests and full reporting of outcomes
☐	☒	Estimates of effect sizes (e.g. Cohen's *d*, Pearson's *r*), indicating how they were calculated

Our web collection on statistics for biologists contains articles on many of the points above.

Software and code

Policy information about availability of computer code

Data collection	No specific software was used for sample collection. Genotype data were generated from sequencing reads. All software used in this study is listed below.
Data analysis	All software used in this work is publicly available. Corresponding publications are cited in the main text and supplementary material. List of software and respective versions: AdapterRemoval (v2.3.1), Burrows-Wheeler Aligner (v0.7.12), DeDup (v0.12.2), mapDamage (v2.0.6), BamUtil (v1.0.14), EAGER (v1), Sex.DetERRmine (v1.1.2) (https://github.com/TCLamnidis/Sex.DetERRmine), ANGSD (v0.915), Schmutzi (v1.5.4), contamMix (v1.0-12), PMDtools (v0.50), pileupCaller (v1.4.0.2), samtools (v1.3.1), Geneious R9.8.1, HaploGrep 2 (v2.4.0), READ (https://bitbucket.org/tguenther/read) (vf541d55), lcMLkin (https://github.com/COMBINE-lab/maximum-likelihood-relatedness-estimation) (v0.5.0), PLINK (v1.90b3.29), Picard tools (v2.27.3), ADNA-Tools (https://github.com/DReichLab/ADNA-Tools) (v3b4357d), smartpca (v16000; EIGENSOFT v6.0.1), qp3Pop (v.435; ADMIXTOOLS v3.0), qpDstat (v.755; ADMIXTOOLS v3.0), Treemix (v1.12), qpWave (v410), qpAdm (v.810), LOCATOR (v1.2), ADMIXTURE (v1.3). The code used in Supplementary Note 2 ("Estimating sex-biased ancestry from uniparental markers in the presence of variable admixture proportions") can be found at https://github.com/stschiff/AngloSaxon_Y-chrom_sex-bias. Data visualisation and descriptive statistical tests were performed in R (v4.1.1). The following R packages were used: Rsamtools (v2.12.0), binom (v1.1-1.1), ape (v.5.6-2), phytools (v1.0-3), psych (v2.2.5), vegan (v2.6-2), factoextra (v1.0.7), ggplot2 (v3.3.6), ggExtra (v0.10.0), ggforce (v0.3.3), rnaturalearth (v0.1.0), sf (v1.0.-8), raster (v3.5-21), elevatr (v0.4.2), rgdal (v1.5-32), spatstat (v2.3-4), maptools (v1.1-4), gstat (v2.0-9), sp (v1.5-0), labdsv (v2.0-1), igraph (v1.3.4), magrittr (v2.0.3), dplyr (v1.0.9), reshape 2 (v1.4.4), and tidyverse (v.1.3.2). Y-chromosome and mtDNA haplogroups were determined using the ISOGG SNP index (v15.73) and PhyloTree (v17-FU1) reference databases, respectively.

For manuscripts utilizing custom algorithms or software that are central to the research but not yet described in published literature, software must be made available to editors and reviewers. We strongly encourage code deposition in a community repository (e.g. GitHub). See the Nature Portfolio guidelines for submitting code & software for further information.

Data

Policy information about availability of data

All manuscripts must include a data availability statement. This statement should provide the following information, where applicable:

- Accession codes, unique identifiers, or web links for publicly available datasets
- A description of any restrictions on data availability
- For clinical datasets or third party data, please ensure that the statement adheres to our policy

Raw sequence data (bam files) from the 479 newly reported ancient individuals will be available prior publication from the European Nucleotide Archive under accession number PRJEB54899. Published genotype data for the present-day British sample are available from the WTCCC via the European Genotype Archive (https://www.ebi.ac.uk/ega/) under accession number EGAD00010000634. Published genotype data for the present-day Irish sample are available from the WTCCC via the European Genotype Archive under accession number EGAD00010000124. Published genotype data for the rest of the present-day European samples are available from the WTCCC via the European Genotype Archive under accession number EGAD00000000120. Published genotype data for the Dutch samples are available by the GoNL request process from The Genome of the Netherlands Data Access Committee (DAC) (https://www.nlgenome.nl). The Genome Reference Consortium Human Build 37 (GRCh37) is available via the National Center for Biotechnology Information under accession number PRJNA31257. The revised Cambridge reference sequence is available via the National Center for Biotechnology Information under NCBI Reference Sequence NC_012920.1. Previous published genotype data for ancient individuals was reported by the Reich Lab in the Allen Ancient DNA Resource v.50.0 (https://reich.hms.harvard.edu/allen-ancient-dna-resource-aadr-downloadable-genotypes-present-day-and-ancient-dna-data).

Field-specific reporting

Please select the one below that is the best fit for your research. If you are not sure, read the appropriate sections before making your selection.

☒ Life sciences ☐ Behavioural & social sciences ☐ Ecological, evolutionary & environmental sciences

For a reference copy of the document with all sections, see nature.com/documents/nr-reporting-summary-flat.pdf

Life sciences study design

All studies must disclose on these points even when the disclosure is negative.

Sample size	We did not rely on statistical methods to predetermine sample sizes. Sample sizes for ancient populations depended solely on the availability of archaeological material and on ancient DNA preservation.
Data exclusions	We selected 379 samples for genome-wide analyses, out of all (n=494) sequenced samples, based on their endogenous content and low contamination estimates. The exclusion criteria mentioned above were pre-established. For analyses including present-day Europeans, individuals with noticeable non-European ancestry were excluded, as described in the Supplementary (Supplementary Note 2; Reference data). Furthermore, closely related individuals were excluded from analyses requiring population allele frequencies.
Replication	We studied unique entities (past and present populations) and did not perform experiments or study various treatments, so replication is not applicable. But we note that samples from the same population carry similar genetic signatures. Moreover, genome-wide data allows for the analysis of multiple realisations of the sample history, by studying hundreds of thousands of SNP sites.
Randomization	We studied unique entities (past and present populations) and did not perform experiments or study various treatments, so randomization is not applicable.
Blinding	We studied unique entities (past and present populations) and did not perform experiments or study various treatments, so blinding is not applicable.

Reporting for specific materials, systems and methods

We require information from authors about some types of materials, experimental systems and methods used in many studies. Here, indicate whether each material, system or method listed is relevant to your study. If you are not sure if a list item applies to your research, read the appropriate section before selecting a response.

Materials & experimental systems

n/a	Involved in the study	
☒	☐	Antibodies
☒	☐	Eukaryotic cell lines
☐	☒	Palaeontology and archaeology
☒	☐	Animals and other organisms
☒	☐	Human research participants
☒	☐	Clinical data
☒	☐	Dual use research of concern

Methods

n/a	Involved in the study	
☒	☐	ChIP-seq
☒	☐	Flow cytometry
☒	☐	MRI-based neuroimaging

Palaeontology and Archaeology

Specimen provenance	Provenance of all samples is outlined in Supplementary Section 1. In summary:<br>Samples from Apple Down were provided by the Novium Museum, Chichester; Permission to analyze was granted to coauthor Ceiridwen Edwards in 2012.<br>Samples from Dover Buckland were provided by the Canterbury Archaeological Trust. Permission to analyse was granted to coauthor Duncan Sayer in 2019.<br>Samples from Widemouth Bay and Newquay, Crantock were provided by the Royal Cornwall Museum. Permission to analyse was granted to coauthor Tom Booth in 2019.<br>Samples from Fox Holes Cave were provided by the Tom Lord's collection at Lower Winskill Farm. Permission to analyse was granted to coauthor Tom Booth in 2015.<br>Samples from Oakington, Polhill and Eastry Updown were provided by the University of Central Lancashire. Permission to analyse was granted to coauthor Duncan Sayer in 2017.<br>Samples from Ely and Hatherdene Close were provided by Oxford Archaeology East. Permission to analyse was granted to coauthor Duncan Sayer in 2017.<br>Samples from Hartlepool, Norton Bishopsmill and Norton East Mill were provided by Tees Archaeology. Permission to analyse was granted to coauthor Tom Booth in 2019.<br>Samples from Dover Hill were provided by the Folkestone Museum. Permission to analyse was granted to coauthor Tom Booth in 2016.<br>Samples from RAF Lakenheath where provided by Cotswold Archaeology Suffolk. Permission to analyse was granted to coauthor Duncan Sayer in 2017.<br>Samples from Lincoln Castle where provided by FAS Heritage. Permission to analyse was granted to coauthor Tom Booth in 2015.<br>Samples from Rookery Hill were provided by the Brighton Museum and Art Gallery. Permission to analyse was granted to coauthor Tom Booth in 2019.<br>Samples from Sedgeford were provided by the Sedgeford Historical and Archaeological Research Project (SHARP). Permission to analyse was granted to coauthor Stephan Schiffels in 2017.<br>Samples from West Heslerton were provided by the Landscape Research Centre. Permission to analyse was granted to coauthors Martin Richards and Ceiridwen Edwards in 2016.<br>Samples from Wolverton were provided by the Milton Keynes Museum. Permission to analyse was granted to coauthor Tom Booth in 2015.<br>Samples from Worth Matravers where provided by the East Dorset Antiquarian Society. Permission to analyse was granted to coauthors Martin Richards and Ceiridwen Edwards in 2012.<br>Samples from Kilteasheen were provided by the Institute of Technology Sligo. Permission to analyse was granted to coauthor Kirsten Bos in 2014.<br>Samples from Groningen were provided by the Gemeente Groningen. Permission to analyse was granted to coauthor Eveline Altena in 2017.<br>Samples from Midlum were provided by the Noordelijk Archeologisch Depot. Permission to analyse was granted to coauthor Eveline Altena in 2017.<br>Samples from Copenhagen were provided by the Museum of Copenhagen. Permission to analyse was granted to coauthor Hannes Schroeder in 2019.<br>Samples from Alt-Inden were provided by the LVR-Amt für Bodendenkmalpflege im Rheinland. Permission to analyse was granted to coauthor Stephan Schiffels in 2021.<br>Samples from Hannover-Anderten, Liebenau, Hiddestorf and Issendorf were provided by the Landesmuseum Hannover. Permission to analyse was granted to coauthor Stephan Schiffels in 2020.<br>Samples from Dunum were provided by the Niedersächsisches Institut für historische Küstenforschung (NIhK). Permission to analyse was granted to coauthor Stephan Schiffels in 2020.<br>Samples from Drantum, Schortens and Zetel were provided by the Landesmuseum Natur und Mensch Oldenburg. Permission to analyse was granted to coauthor Stephan Schiffels in 2020.<br>Samples from Häven were provided by the University of Rostock. Permission to analyse was granted to coauthor Joscha Gretzinger in 2021<br>Samples from Schleswig were provided by the Stiftung Schleswig-Holsteinische Landesmuseen Schloss Gottorf. Permission to analyse was granted to coauthor Ben Krause-Kyora in 2017.
Specimen deposition	Specimens were returned to the owning institutions after laboratory analyses.
Dating methods	A subset of bone samples were sent to the Curt-Engelhorn-Center Archaeometry gGmbH (CECA) to be dated by 14C. Collagen was extracted from the bone samples (modified Longin method), purified by ultrafiltration (fraction >30kD) and freeze-dried. The analysis was performed on a MICADAS-type AMS system at CECA. The isotopic ratios 14C/12C and 13C/12C of samples, calibration standard (Oxalic Acid-II), blanks and control standards were measured simultaneously in the AMS. 14C-ages are normalized to delta 13C=-25‰ and calibrated using the dataset IntCal20 and software SwissCal (L.Wacker, ETH-Zürich).

☒ Tick this box to confirm that the raw and calibrated dates are available in the paper or in Supplementary Information.

Ethics oversight	No ethical oversight was required strictly. However, we confirm that all analyses followed established ethical guidelines for archaeogenetic research, as detailed in Wagner et al., AJHG, 2020 and Alpaslan-Roodenberg, Nature, 2021.

Note that full information on the approval of the study protocol must also be provided in the manuscript.
